# Biochemical and Transcriptional Regulation of Membrane Lipid Metabolism in Maize Leaves under Low Temperature

**DOI:** 10.3389/fpls.2017.02053

**Published:** 2017-11-30

**Authors:** Yingnan Gu, Lin He, Changjiang Zhao, Feng Wang, Bowei Yan, Yuqiao Gao, Zuotong Li, Kejun Yang, Jingyu Xu

**Affiliations:** ^1^Key Laboratory of Modern Agricultural Cultivation and Crop Germplasm Improvement of Heilongjiang Province, College of Agriculture, Heilongjiang Bayi Agricultural University, Daqing, China; ^2^Remote Sensing Technique Center of Heilongjiang Academy of Agricultural Sciences, Harbin, China

**Keywords:** lipid metabolism, lipidome profiling, RNA-Seq, low temperature, maize

## Abstract

Membrane lipid modulation is one of the major strategies plants have developed for cold acclimation. In this study, a combined lipidomic and transcriptomic analysis was conducted, and the changes in glycerolipids contents and species, and transcriptional regulation of lipid metabolism in maize leaves under low temperature treatment (5°C) were investigated. The lipidomic analysis showed an increase in the phospholipid phosphatidic acid (PA) and a decrease in phosphatidylcholine (PC). And an increase in digalactosyldiacylglycerol and a decrease in monogalactosyldiacylglycerol of the galactolipid class. The results implied an enhanced turnover of PC to PA to serve as precursors for galactolipid synthesis under following low temperature treatment. The analysis of changes in abundance of various lipid molecular species suggested major alterations of different pathways of plastidic lipids synthesis in maize under cold treatment. The synchronous transcriptomic analysis revealed that genes involved in phospholipid and galactolipid synthesis pathways were significantly up-regulated, and a comprehensive gene-metabolite network was generated illustrating activated membrane lipids adjustment in maize leaves following cold treatment. This study will help to understand the regulation of glycerolipids metabolism at both biochemical and molecular biological levels in 18:3 plants and to decipher the roles played by lipid remodeling in cold response in major field crop maize.

## Introduction

Low temperature is a major abiotic stress plants are frequently exposed to, which severely affects plant growth and productivity (Mahfoozia et al., 2006; Sandve et al., 2011). Most temperate plant species have evolved specialized mechanisms to survive cold at physiological, molecular and biochemical levels (Janmohammadi, 2012). A major strategy plants have developed to adapt to temperature stress was the membrane fluidity modulation, which is affected by the relative proportions of various lipid classes in the lipid bilayer and the degree of unsaturation in the fatty acyl groups of glycerolipid molecules (Harwood, 1991; Murata et al., 1992; Murakami et al., 2000; Li Q. et al., 2016).

Glycerolipids are the main building-blocks for plant membranes, and there are big differences in glycerolipid proportion and fatty acyl chains composition among different membrane systems (Li N. et al., 2016). The plasma membrane of plant cells consists mainly of phospholipids PC and PE; whereas chloroplast (and thylakoid) membrane is mainly composed of galactolipids MGDG and DGDG, which are closely related to thylakoid membrane structure and photosynthetic properties of plants (Benning, 2009). In plant cells, fatty acids are synthesized exclusively in the chloroplast, while the synthesis of glycerolipids has two completely independent pathways, one in the plastid/chloroplast and the other in the endoplasmic reticulum, generally referred to as the prokaryotic and eukaryotic pathways, respectively (Browse et al., 1986; Ohlrogge and Browse, 1995). Although the two pathways are separated from each other in space, they are synergistically regulated by various factors during the process of lipid synthesis, and there are big differences among different plant species (Zheng et al., 2011; Li Q. et al., 2016). Due to the specificity of the acyltransferases, the galactolipids produced by the prokaryotic pathway in the chloroplast carry C16 fatty acids at the second acyl (sn-2) position, whereas the eukaryotic pathway produces galactolipids with only C18 fatty acid at the second acyl (sn-2) position (Ohlrogge and Browse, 1995). In some plants such as *Arabidopsis thaliana* and spinach, the main chloroplast lipids (glyceroglycolipid MGDG and DGDG) are produced by the prokaryotic pathway and eukaryotic pathway together. The symbolic phenomenon is that the two lipids contain high levels of C16 and have almost equal proportion of C34:6 and C36:6, thus such plants are called 16:3 plants. While in other category, such as peas, wheat, and maize, the synthesis of plastidic lipids almost completely depends on the eukaryotic pathway, and the product of this process contains a large amount of 18:3 fatty acids, resulting dominating C36:6 in their MGDG and DGDG, thus they are called 18:3 plants (Heinz and Roughan, 1983; Browse et al., 1986; Lohden and Frentzen, 1988; Ohlrogge and Browse, 1995).

There have been a number of studies in *Arabidopsis* (16:3 plant) that have revealed the balance of prokaryotic and eukaryotic pathways of lipid synthesis is associated with abiotic stresses, and an enhanced eukaryotic pathway contribution has been observed in various stress conditions (Nakamura et al., 2009; Moellering and Benning, 2011; Higashi et al., 2015; Li et al., 2015). Though a few recent studies have also suggested the adjustment of two pathways and a flow of eukaryotic pathway derived precursors to chloroplast in wheat (18:3 plant) under temperature stress, yet the information for explicating the mechanism underlying the lipid regulation in18:3 plants is still lacking (Li et al., 2015; Narayanan et al., 2016a,b). In addition to the strong biochemical evidences on the glycerolipid pathways adjustment, some molecular studies have demonstrated that enzymes associated with the two pathways are affected at transcriptional level synergistically (Shen et al., 2010). In *Arabidopsis*, the coordinated gene expression in parallel with changes in glycerolipid species was revealed in response to light and temperature stimulus (Burgos et al., 2011; Szymanski et al., 2014; Li et al., 2015). Nonetheless, comprehensive information on the biological and biochemical mechanisms of the lipids metabolism in both 16:3 and 18:3 plants has been lacking, and the precise factors involved in the respective processes remain largely unknown (Li Q. et al., 2016).

In this study, an integrated lipidomic and transcriptomic strategy was carried out to investigate the changes in glycerolipid contents and species as well as transcriptional regulation of lipid metabolism in maize leaves under low temperature treatment, in an attempt to obtain better understanding on the regulation of glycerolipid pathway adjustment in response to cold in18:3 plants. Originated in the temperate zone, maize has frequently been challenged by low temperature stress in cold regions, which causes an increase in molecular disorder and disintegration of lipid bilayers. This study will help to decipher the roles played by lipid metabolism in acclimatization responses of maize suffering from cold stress.

## Materials and Methods

### Plant Growth, Treatments, and Sampling

Seeds of maize (He 344) were immersed in 1% (v/v) bleach for 30 min and then rinsed with sterilized water. Maize seeds were planted in twelve 6-in pots with vermiculite: soil (1:1, v/v), and the growth chamber was set at 22°C, with 16 h/8 h (light/dark) daily photoperiodic cycle. At the 2-week old stage, six pots of maize seedlings were removed to another growth chamber set at 5°C (all other settings remained the same) for low temperature treatment, and the ones left at 22°C were used as a control. Maize leaf samples were collected at 3 days after each treatment. All samples had at least three replicates, and all replicates were sampled at the same part of maize seedlings at the same time. The collected samples were wrapped with tin foil rapidly, and frozen immediately with liquid nitrogen and stored at -80°C.

### Maize Leaf RNA-seq Analysis and RT-PCR Validation

Total RNA was prepared from maize leaf samples collected from 2-week old seedlings after 3 days of treatment under 5°C (treatment)and 22°C (control) using TRIzol reagent (Invitrogen). After the quality inspection, a total amount of 20 μg RNA from each sample was used to build the library. The process was started with the synthesis of two cDNA strands, followed by the purification of the double-stranded cDNAs, and finally obtained cDNA library by PCR enrichment. The library integrity was assessed using Qubit 2.0. The insert size was purified (AMPure XP system) and quantified using the Agilent high sensitivity DNA assay on the Agilent Bioanalyzer 2100 system. The high throughput sequencing was done using Illumina HiSeq system, and was conducted in triplicate for each treatment. The raw data (raw reads) were filtered with FASTQ_Quality_Filter tool from the FASTX-toolkit. The clean data were used for further analysis. After preprocessing the RNA-seq data, the reads were mapped to the maize reference genome version 3 (B73 RefGen_v3). The Sequence Alignment generated by Tophat was then processed by the software Cufflinks to assemble the alignments in the Sequence Alignment/Map file into transcript fragments (transfrags). FPKM was used as the unit of measurement to estimate transcript abundance. Differential expression analysis of all samples was performed using the Cuffdiff program. Candidate DEGs were submitted to GO, COA, KEGG, Swissprot, and NR databases, respectively. Enriched GO terms were selected using Singular Enrichment Analysis (SEA) with the maize reference genome B73 as background. This RNA-seq data has been submitted to the online SRA (Sequence Reads Archive) database with the accession number SRX2672484.

The real-time RT-PCR was performed to validate the RNA-seq results. Gene-specific primers were designed based on the selected genes sequence (see Supplementary Table S1 for primers used in this study). The maize 18s rRNA gene was used as the internal control. Each10 μL reaction system contained 3.4 μL ddH_2_O, 5.0 μL SYBR^®^ Green Master Mix, 0.3 μL of each primer, and 1.0 μL cDNA template. The PCR reaction was initiated with a starting step of 95°C/60 s; followed by 40 cycles of 95°C/15 s, and 55°C/30 s; and terminated at 72°C for 60 s.

### Lipid Extraction and Analysis

After 3 days of low temperature (5°C) treatment, leaf samples from 2-week old maize seedlings were collected from five plants in different pots (the samples from plants growing in 22°C were used as control), and the lipidomic analysis was done with five replicates. The extraction of total lipid was conducted as previously reported (Narayanan et al., 2016a). About 200 mg leaves were cut and quickly immersed in75°C isopropanol (3.0 ml with 0.01% BHT for 15 min) in a 50 ml glass tubes (Teflon-lined with screw-cap). After cooling to room temperature, chloroform (1.5 ml) and water (0.6 ml) were added, vortexed and then shook for 1 h, and then lipid extracts were transferred to new glass tubes. Repeated extraction procedure was performed with chloroform/methanol (2:1), and then the lipid extracts were combined and washed with 1 M KCl (1.0 ml). The upper phase was discarded, and samples were evaporated completely and stored at -80°C. For fatty acid compositional analyses, the extracts were dissolved in 1 mL of chloroform, and precise amounts of internal standards, obtained and quantified as previously described (Welti et al., 2002). Unfractionated lipid extracts were introduced by continuous infusion into the ESI source on a triple quadrupole MS/MS (API4000, AB Sciex, Framingham, MA, United States). Samples were introduced using an autosampler (LC Mini PAL, CTC Analytics AG, Zwingen, Switzerland) fitted with the required injection loop for the acquisition time and presented to the ESI needle at 30 μL min^-1^ (Narayanan et al., 2016a). The mass spectrometry lipid profiling was conducted at Kansas Lipidomics Research Center (KLRC, United States). The mass spectral parameters were provided in Supplementary Table S4. The spectral specific scan of the identified lipid species were shown in Supplementary Figure S4. The lipidomic results were presented as values (mol %) = means 5 ± standard deviation (SD) (*n* = 5), and the statistical analysis was conducted by the SPSS statistics 21.0 with the significant level set at α = 0.05. The raw data could be find in Supplementary Tables S5–S7.

### Analysis of the Expression Correlation of Lipid Related DEGs and Construction of Co-expression Networks

Spearman correlation coefficient analysis was conducted on the RNA-seq data of the lipid-related DEGs (differentially expressed genes, Log2FC ≥1.5). The pairwise correlation coefficients were computed based on the expression data of the DEGs. After removing the self-pairing and repetition, a correlation cutoff of 0.9 was applied and the retained gene pairs were used for the construction of the co-expression network. The co-expression network was constructed and visualized using the Cytoscape software, and the genes within different categories were differently colored.

## Results

### Metabolism of Membrane Glycerolipids in Maize Leaves under Low Temperature

**FIGURE 1:**
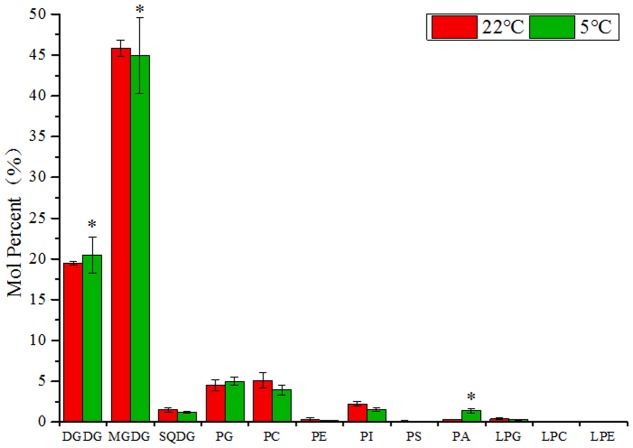
Changes of glycerolipids species in maize leaves under low temperature (5°C) in comparison to room temperature (22°C). Values (mol %) are means 5 ± standard deviation (SD) (*n* = 5). MGDG, monogalactosyldiacylglycerol; DGDG, digalactosyldiacylglycerol; SQDG, sulfoquinovosyldiacylglycerol; PG, phosphatidylglycerol; PC, phosphatidylcholine; PE, phosphatidylethanolamine; LPG, Lyso-PC; LPC, Lyso-PC; LPA, Lyso-PC. “^∗^” indicated that the value was significantly different from the control (*P* < 0.05).

To investigate the membrane lipids metabolism in maize leaves under low temperature, a lipidomic approach was carried out to measure the changes in contents and fatty acid compositions of the major glycerolipids (Welti et al., 2002). The total lipid extraction from leaf samples of 2-week old maize seedlings after 3 days treatment at 5°C (low temperature) and 22°C (room temperature control) were probed for individual lipid molecular species within each subset of glycerolipids. A total of eleven headgroup classes of lipids were detected by ESI-MS/MS, including two classes of galactolipids (MGDG and DGDG), six of phospholipids (PG, PC, PE, PI, PS, and PA), one of sulfolipid (SQDG), and three of lyso-phospholipids (LPG, LPC, and LPE) (**Figure 1**).

As shown in **Figure 1**, in the lipid samples from maize leaves, the two types of galactolipids MGDG and DGDG were the most abundant lipid species, accounting for approximately 75% of the total components. The major phospholipids PC, PG, PI, and PA accounted for about 15% of the total lipids and followed by a small proportion of SQDG (2–3%). The levels of phospholipids PE and PS, and three types of lyso-phospholipids were relatively low. Under low temperature (5°C), the level of DGDG increased, whereas the level of MGDG and SQDG declined in comparison to control (22°C). In the phospholipids group, the accumulation of PA was significantly induced by cold (4.9-fold increase), and the level of PG was also increased. However, the content of other phospholipids were lowered, including PC, PI, PE, and PS. Although the signals were low for lyso-phospholipids, an increase in the level of LPC and LPE and a decrease in LPG were observed.

### The Analysis of Lipid Molecular Species Revealed Contribution of Different Pathways to Plastidic Lipids Synthesis in Maize

**FIGURE 2:**
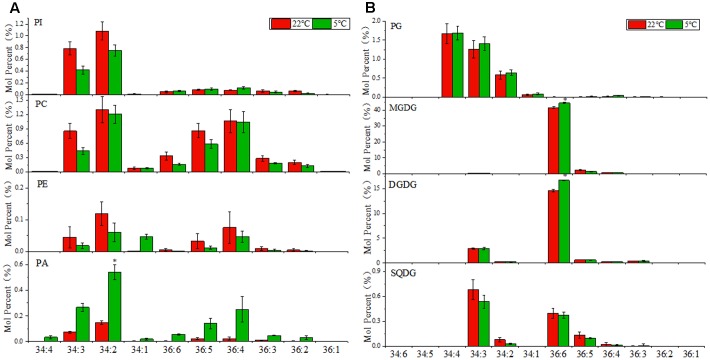
Changes in diacyl lipid molecular species of major phospholipids and plastidic glycerolipids in maize leaves under low temperature (5°C) in comparison to room temperature (22°C). **(A)** Major phospholipids, PI, phosphatidylinositol; PC, phosphatidylcholine; PE, phosphatidylethanolamine; PA, phosphatidic acid. **(B)** Major plastidic glycerolipids, PG, phosphatidylglycerol; MGDG, monogalactosyldiacylglycerol; DGDG, digalactosyldiacylglycerol; SQDG, sulfoquinovosyldiacylglycerol. Values (mol %) are means 5 ± standard deviation (SD) (*n* = 5). “^∗^” indicated that the value was significantly different from the control (*P* < 0.05). More detailed MGDG and DGDG molecular species as shown in Supplementary Figure S3.

The fatty acid composition of glycerolipids was profiled via the lipidomic approach. As shown in **Figure 2A**, the phospholipids PI, PC, PE, and PA were comprised of a diverse molecular species ranging from C34:1 to C36:6 (total number of acyl carbon atoms: number of double bounds). The C34 molecules were dominating in PI, and accounted for a large proportion in PA, while PC and PE were comprised of the similar proportion of C36 and C34 molecular species. As mentioned earlier, 16:3 and 18:3 plants could be distinguished by their fatty acid profiles of glycerolipids. As shown in **Figure 2B**, the predominant C36:6 molecules in MGDG and DGDG reflected a dependence of the eukaryotic/ER pathway for galactolipids synthesis in maize, which was in accord with the characteristics of a typical 18:3 plant. A small proportion of C34 molecules was found in DGDG, mainly in the form of C34:3, which might resulted from the reduction of C34PC from eukaryotic/ER pathway or a contribution from the chloroplast pathway. In sulfolipid SQDG, almost equal amount of C34 and C36 molecular species were detected, leading by C34:3 and C36:6, respectively, which suggested that both the prokaryotic/chloroplast pathway and the eukaryotic/ER pathway contributed to SQDG production. As for the only chloroplast compartmentalized phospholipids PG, the C34 molecules were dominate, indicating that PG was derived almost entirely from the prokaryotic/chloroplast pathway. Under (5°C) low temperature treatment, the level of fully desaturated C36:6 DGDG and MGDG species was raised, which might be beneficial for the chloroplast membrane to cope with cold stress.

### Transcriptomic Analysis of Maize Leaves under Low-Temperature

In an attempt to investigate the molecular biological regulation of the membrane lipid metabolism in maize. A global transcriptional analysis was carried out using the Illumina RNA-seq approach. The RNA was prepared from maize leaf samples collected from 2-week old seedlings after 3 days of treatment under 5°C (treatment) and 22°C (control). A total of 53.03 Gb clean data was generated for nine runs (three replicates for each treatment), and at least 4.10 Gb clean data was yield for each single run. Approximately 20–28 million clean reads were generated for each sample, and 65–70% of them were mapped to the reference genome. The clean reads were further annotated with the GO, COA, KEGG, Swissprot, and NR databases, respectively. A total number of 40,940 unigenes were annotated and 18,813 of them had a length >1 kb. The real-time RT-PCR analyses were performed on a number of differentially expressed genes to validate the RNA-seq data, and the result demonstrated a high correlation (R2 = 0.9399, *P* < 0.05) (Supplementary Figure S1). This RNA-seq data has been submitted to the online SRA (Sequence Reads Archive) database with the accession number SRX2672484.

**FIGURE 3:**
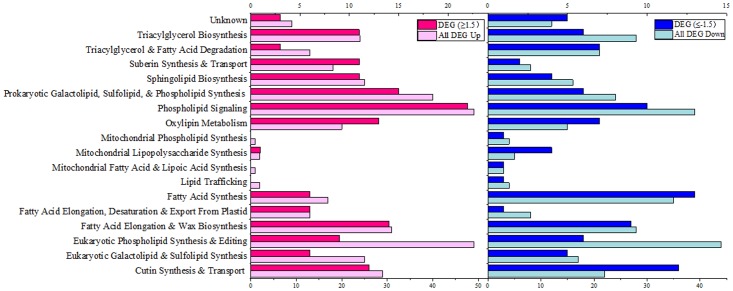
Functional categorization of lipid-related genes from transcriptome of maize leaves (5°C vs. 22°C). Genes involved in lipid metabolism were compiled from the *Arabidopsis* lipid gene database (Beisson et al., 2003) and categorized based on different pathways. Pink columns represent down-regulated genes, and blue columns represent up-regulated genes. In each category, the light colored column represents the total differentially expressed genes (DEG), and the dark colored column represents the significantly differentially expressed genes (DEG, Log2FC ≥1.5 or ≤–1.5). The number of the genes in each category were displayed in the *x*-axis (the ones on the upper *x*-axis indicate the DEG, Log2FC ≥1.5 or ≤–1.5).

The genome-wide screening of maize lipid related genes was carried out according to the GO and KEGG annotation and also based on the previously released *Arabidopsis* lipid gene database and some recent supplementaries (Beisson et al., 2003; Troncoso-Ponce et al., 2013; Botella et al., 2017). A total of 556 genes was identified as lipid-related from the 22°C vs. 5°C (22°C was control) transcriptome. The differentially expressed genes (DEGs) were further screened out based on the criteria of Log2FC ≥1.5 or ≤-1.5 (FDR ≤0.01), which resulted in a total of 212 DEGs including 117 up-regulated and 95 down-regulated. These lipid-related genes were further categorized into specific pathways, and the locus number of genes involved in each category of lipid metabolism was shown in **Figure 3**. Under 5°C low temperature treatment, a wide range of lipid-related pathways were perturbed in comparison to 22°C. Genes involved in “Phospholipid Signaling,” “Eukaryotic Phospholipid Synthesis and Editing” and “Prokaryotic Galactolipid, Sulfolipid, and Phospholipid Synthesis” were significantly up-regulated, and the ratio of DEGs was 22-up/10-down, 9-up/6-down and 15-up/6-down (Log2FC ≥1.5 or ≤-1.5), respectively. Genes involved in “Eukaryotic Galactolipid and Sulfolipid Synthesis” were 6-up/5-down regulated (Log2FC ≥1.5 or ≤-1.5). Genes involved in “Fatty Acid Synthesis” were significantly down-regulated as 6-up/13-down (Log2FC ≥1.5 or ≤-1.5), and genes involved in “Mitochondrial Lipid Metabolism” were mostly down-regulated. We also observed the up-regulation of a large number of genes involved in “Fatty Acid Elongation and Wax Biosynthesis” under 5°C (**Figure 3**). These results indicated that both phospholipid and galactolipid pathways were activated under low temperature treatment at the transcriptional level.

The expression correlation analysis was conducted on the 212 lipid-related DEGs (differentially expressed genes, Log2FC ≥1.5), and a correlation coefficient greater than 0.9 was applied and 170 genes were retained for the construction of co-expression network (Supplementary Figure S2A). The lipid-related DEGs were separated into two subnetworks based on their expression correlation, The major co-expression cluster consisted of a large number of up-regulated genes mainly involved in lipid metabolic pathways, such as phospholipids synthesis and editing (*GPAT7*, *LPAT5*, *CCT*, *PAH*, *FAD2,* and *PLD*), implying a modular regulation of these pathways.

### Analysis of Lipid Related DEGs Involved in Major Lipid Metabolism Processes

The differentially expressed genes (DEGs, Log2FC ≥1.5 and FDR ≤0.01) involved in the major membrane lipids metabolism processes were selected and listed in **Table 1** (for the full list and detailed info see Supplementary Table S2). As shown in **Table 1** (and Supplementary Table S2), under low temperature, genes involved in the early steps of *de novo* synthesis of storage lipids TAG were up-regulated including GPAT (glycerol-3-phosphate acyltransferase) and LPAT (lysophosphatidyl acyltransferase) isoforms. Genes involved in the *de novo* synthesis of phospholipid PC were induced as well, including 2 CEKs (choline kinase) and CCT (choline phosphate cytidylyltransferase). Whereas one DGAT (diacylglycerol acyltransferase) and two PDAT (phospholipid:diacylglycerol acyltransferase) isoforms for TAG production were identified as being down-regulated. The findings together might suggest that the synthesis of phospholipids PC was enhanced by the *de novo* assembly steps.

**Table 1:** The differentially expressed genes (DEGs) involved in the major lipids metabolism processes in maize leaves under low temperature.

Name Abbreviations	Maize ID	Putative function	Log2 fold change (5/22°C)
**TAG/PC *de novo* synthesis**			
GPAT7	GRMZM2G059637	Glycerol-3-phosphate acyltransferase 7	+3.16
LPAT5	GRMZM2G135027	Lysophosphatidyl acyltransferase 5	+2.27
DGAT2	GRMZM2G042356	Diacylglycerol acyltransferase	-3.04
PDAT1	GRMZM2G088291	Phospholipid:diacylglycerol acyltransferase	-2.33
CEK4	GRMZM2G469409	Choline/ethanolamine kinase (P)	+2.88
CCT2	GRMZM2G132898	CTP : phosphocholine cytidyltransferaseaaa	+4.50
**PC turnover and DAG formation**			
PLDα	GRMZM2G054559	Phospholipase D	+3.31
PLDα	GRMZM2G019029	Phospholipase D	+1.67
PLDα	GRMZM2G179792	Phospholipase D	+2.45
NPC1	GRMZM2G116876	Non-specific phospholipase C	-2.21
NPC3	GRMZM2G422670	Non-specific phospholipase C	-3.24
NPC4	GRMZM2G081719	Non-specific phospholipase C	-4.16
PAH1	GRMZM2G099481	Phosphatidic acid phosphatase (E	+3.08
PAP1/LPP1	GRMZM2G024144	Phosphatidic acid phosphatase	+6.24
PAP2/LPP2	GRMZM2G447433	Phosphatidic acid phosphatase	-2.14
LPPa3	GRMZM2G077187	Phosphatidic acid phosphatase (E	+2.72
**Galactolipid synthesis**			
MGD1	GRMZM2G142873	Monogalactosyl diacylglycerol synthase 1	+1.41
MGD2	GRMZM2G141320	Monogalactosyl diacylglycerol synthase 2	+4.49
MGD3	GRMZM2G178892	Monogalactosyl diacylglycerol synthase 3	+1
DGD1	Maize_newGene_1953	Digalactosyl diacylglycerol Synthase1	+0.84
DGD2	GRMZM2G092588	Digalactosyl diacylglycerol Synthase1	+1.4
SQD2	GRMZM2G117153	Sulfoquinovosyl diacylglycerol Synthase1	+1.33
**Fatty Acid desaturation and formation**			
FAD2	GRMZM2G056252	Fatty acid desaturase	+2.75
FAD3	GRMZM2G354558	Fatty acid desaturase	+2.22
FAD8	GRMZM2G128971	Fatty acid desaturase	+2.22
FATB	GRMZM2G007489	Fatty acyl-acyl carrier thioesterase B	2.73
LACS3	GRMZM5G812228	Acyl-CoA synthetaseaaa	+2.67
DGL5	GRMZM2G359904	Plastic acylase (E)	+5.09

Phosphatidylcholine is the most abundant and important phospholipid, which works not only as the major building blocks for plasma membrane but also as an active site for acyl-editing and as the precursor for the regeneration of PA, DAG, and acyl-CoA pools, through degradation catalyzed by different lipins. The regenerated PA, DAG and acyl-CoA pools with distinct acyl profiles could be used for multiple purposes, including the synthesis of storage lipids TAG and plastidic galactolipids. Phospholipase PLD and PLC both involved in the hydrolization of phospholipids PC and PE to produce PA and DAG. In this study, 5 out of 6 PLD isoforms were up-regulated, and 4 of them were PLDα and 1 was PLDβ2; whereas all 3 NPCs (non-specific PLC) identified were down-regulated, implying that the PLD pathway was the major one in hydrolyzing PC to generate DAG under cold stress (**Table 1** and Supplementary Table S2). Multiple genes involved in the PA to DAG conversion have been identified: a soluble PAH ortholog (phosphatidate phosphohydrolase), which was supposed to be involved in ER pathway, was up-regulated (Log2FC 3.08); and 5 out of 6 PAP (PA phosphatase)/LPP (lipid phosphate phosphatase) orthologs, representing the membrane bound and putative plastidic localized PAP (see Supplementary Table S3 for the prediction of transmembrane domains and subcellular localizations), were induced by cold as well. The most highly induced PAP1/LPP1 was an ortholog of AtLPP1 (At2g01180), which had a Log2FC of 6.24.

In 18:3 plant maize, the galactolipids synthesis was supposed to rely entirely on the eukaryotic pathway. However, the functions and regulation mechanism of genes involved in galactolipid synthesis pathways has remained unknown. In this study, we took a closer look at the putative genes involved in galactolipids pathway in our RNA-seq data and identified a total of 6 genes encoding MGD (monogalactosyl diacylglycerol synthase), DGD (digalactosyl diacylglycerol synthase), and SQD (SQDG synthase), respectively. The genes involved in galactolipid synthesis pathways were all up-regulated under cold stress, and the most upregulated was MGD2 having a Log2FC of 4.49 (**Table 1**).

A set of FADs (fatty acid desaturase) involve in the fatty acid desaturation in both phospholipid and galactolipid pathways in plants. In our RNA-seq database, all detected FADs were up-regulated, including FAD2 and FAD3 for PC-mediated acyl-editing and FAD7 and FAD8 for the complete desaturation of galactolipids. A set of PLA-I family lipases DONGLE–DGLs, which catalyze the hydrolysis of phospholipids and galactolipids to release free fatty acid, were also found up-regulated (**Table 1**).

### The Gene-Metabolite Network of Membrane Glycerolipids Metabolism in Maize under Low Temperature

**FIGURE 4:**
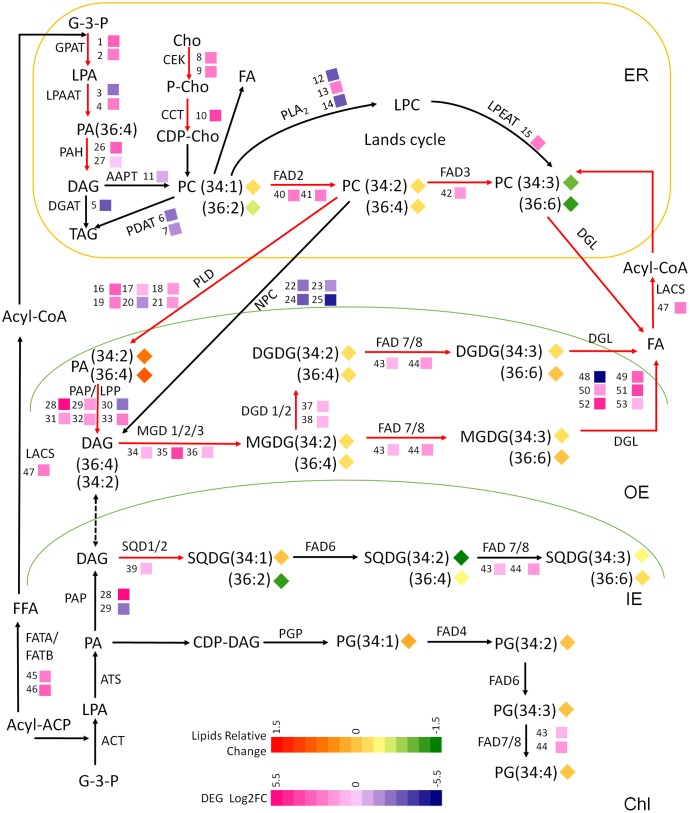
A schematic diagram of gene-metabolite network demonstrates lipid metabolism in maize under low temperature stress. The glycerolipids synthesis pathways were depicted and the involving genes and lipid melatolites were symbolized. The relative change of lipid molecular species [(5°C mol % –22°C mol %)/22°C mol %] and the relative expression levels of selected genes [Log2FC ≥1.5 or ≤–1.5, (5°C vs. 22°C)] were marked as heat-map icons. The red arrows represents activated steps by cold. ER, endoplasmic reticulum; OE, outer envelope; IE, inner envelope; Chl, chloroplast. The map is based on (Ohlrogge and Browse, 1995; Benning, 2009; DeBolt et al., 2009; Shen et al., 2010; Narayanan et al., 2016a).

Based on the results of the transcriptomic and lipidomic studies, a schematic diagram was drawn to illustrate the gene-metabolite network of maize leaf lipid under cold stress (**Figure 4**). The metabolic pathways pertaining to glycerolipids metabolism were depicted and the transcript levels and fatty acid profiles were marked with colored heatmap icons. As illustrated in **Figure 4**, the transcriptionally activated steps/pathways under cold were indicated by red arrows, which mainly involved the synthesis and turnover of PC, generation and degradation of galactolipids MGDG and DGDG, and fatty acids desaturation.

In plant, the generation of DAG via PC degradation was catalyzed by two types of phospholipases PLD and PLC, while the up-regulated transcripts of PLDs and PAH/PAP/LPP suggested that the PC-PA-DAG route was the major contributor to the galactolipids synthesis under cold. The significantly up-regulated PAP/LPPs implicated their possible involvement in dephosphorylation of PA channeled from ER to chloroplast in18:3 plants. The lipidomic analysis revealed that major phospholipids PC was decreased accompanied by an increased PA, which also implied there might be an intensive degradation of PC after the *de novo* synthesis processes.

All genes identified as being involved in the synthesis of galactolipids MGDG and DGDG, and the sulfolipids SQDG were transcriptionally up-regulated. The biochemical evidence was observed by the lipidomic data showing an enhanced accumulation of DGDG—the end product of galactolipids synthesis and the major chloroplast bilayer lipid. The fatty acid profiling showed that C36:6 were the dominating molecular species for both MGDG and DGDG, which suggested that C36:4 DAG produced from C36:4 PC was preferred precursors for MGDG and DGDG production. SQDG had the fatty acid traits of both ER and chloroplast, reflecting a recruitment of DAG precursors from both eukaryotic and prokaryotic pathway. Although the changes at the level of gene transcription were significant, the changes of lipid contents and acyl profiles were not dramatic. It is possibly due to the activation of galactolipid metabolism at the biochemical level lagging behind that of transcriptome, which perhaps would occur after a longer term cold exposure-later than 3 days.

To analyze the modular regulation of DEGs involved in the major lipid processes, the expression correlation of DEGs which appeared in pathways in **Figure 4** (listed in Supplementary Table S2) was measured and the co-expression network was constructed. As revealed in Supplementary Figure S2B, a highly relevant gene expression cluster was constituted by genes involved in phospholipid synthesis and degradation (*GPAT7*, *LPAT5*, *CCT*, and *PLD*, *LPP*), galactolipids synthesis (*MGD1* and *DGD2*), and fatty acid and acyl-CoA production and desaturation (*FATB*, *DGL*, and *FAD2*), which was in agreement with the above finding of the activation of these pathways, within which most of the involved genes were up-regulated.

### Lipid Transporter Genes and Lipids Related Transcriptional Factors Responses to Low Temperature

A screening of maize lipid related transporters and transcriptional factors was conducted based on the previous released *Arabidopsis* lipid gene database and some recent findings (Beisson et al., 2003; Manan et al., 2016). And the differentially expressed candidates were listed in **Table 2**. In plant, although the synthesis of fatty acid takes place in chloroplast, the synthesis of different kinds of lipids occurs in various compartments, thus the lipids transportation among different organelles are needed (Li N. et al., 2016). A complex of TGD (trigalatosyldiacylglycerol) proteins have been characterized from *Arabidopsis* which transport PA from ER to chloroplast (Wang et al., 2012; Manan et al., 2016). All the TGDs appeared in our maize RNA-seq database were repressed under low temperature. Some ACBGs (ATP-Binding Cassette), which could export lipid precursor out of the plasma membrane, were identified and 4 out of 5 of them were up-regulated. One ACBP5 (Acyl-CoA binding protein) which might involve in FA exporting from chloroplast, and 1 LACS3 (Long-chain Acyl-coenzyme A Synthetase) which might relate to FA and lipid transport, were also found induced by cold.

**Table 2:** The differentially expressed lipid-related transporters and transcriptional factors in maize leaves under low temperature.

Name abbreviations	Maize ID	Putative function	Log2 fold chang (5/22°C)	*Arabidopsis* ID
**Transporters**				
TGD1	GRMZM2G170516	Trigalactosyldiacylglycerol 1	-3.00	At3G06960
TGD2	GRMZM2G138995	Trigalactosyldiacylglycerol 2	-2.34	At3G20320
TGD4	EF517601.1_FG015	Trigalactosyldiacylglycerol 4	-2.68	At1G19800
ABCG15	GRMZM2G157564	ATP-binding cassette G15	-2.00	At3G53510
ABCG20	GRMZM2G099619	ATP-binding cassette G20	+3.35	At5G19410
ABCG23	GRMZM2G036940	ATP-binding cassette G23	+4.31	At3G13220
ABCG26	GRMZM2G076526	ATP-binding cassette G26	+2.16	At5G60740
ABCG28	GRMZM2G064603	ATP-binding cassette G28	+2.07	At3G21090
ACBP5	GRMZM2G085547	Acyl-CoA binding protein 5	+2.08	At5G27630
LACS3	GRMZM5G812228	Long-chain acyl-coenzyme A synthetase	+2.67	At1G64400
**Transcription factors**				
WRI	GRMZM2G131266	WRINKLED 1	-6.87	At3G54320
Val 2	GRMZM2G008356	Transcriptional factor B3	+2.30	At4G32010
bZIP	GRMZM2G448607	Basic leucine zipper proteins	+3.76	At3G62420
Dof	GRMZM2G456452	DNA binding with one finger	+1.74	At4G00940
PKL	Maize_newGene_5126	PICKLE	+3.42	At2G25170
ARF 7	GRMZM2G073750	Auxin-responsive factor 7	-3.40	At5G20730
ARF19	GRMZM2G317900	Auxin-responsive factor 19	+3.10	At1G19220
ARF19	GRMZM2G160005	Auxin-responsive factor 19	+2.08	At1G19220
MYB96	GRMZM2G139284	MYB domain protein 96	-3.27	At3G01140
MYB106	GRMZM2G097636	MYB domain protein 106	+6.97	At3G01140
MYB106	GRMZM2G162709	MYB domain protein 106	-3.74	At5G62470

WRI1 has been identified as an important regulator of oil production and a member of the APETALA2 family of TFs, which regulates the genes involved in FA and TAG biosynthesis (Shen et al., 2010; Pouvreau et al., 2011). As revealed in **Table 2**, the maize WRI1 was significantly reduced under low temperature condition, which was in agreement with our findings that a larger number of genes were found down-regulated in FA and TAG synthesis processes, as shown in **Figure 4**. A bZIP and a Dof, which might associate with either FA or lipid metabolism, were found up-regulated. A number of ARF and MYB exhibited different expression under cold.

## Discussion

The membrane lipid metabolism and remodeling, which modulate the lipid composition, fatty acyl group unsaturation, and membrane fluidity, have been developed as a key strategy for plant to cope with temperature stresses (Li Q. et al., 2016). In plant, there are two pathways responsible for glycerolipids synthesis – the endoplasmic reticulum compartmentalized eukaryotic pathway and the chloroplast/plastid compartmentalized prokaryotic pathway. In the typical 16:3 plant *Arabidopsis*, eukaryotic pathway and prokaryotic pathway contribute almost equally to galactolipids synthesis occurred in chloroplast, whereas the 18:3 plants have been proved to be relying almost entirely on eukaryotic pathway for galactolipids synthesis (Heinz and Roughan, 1983; Browse et al., 1986; Lohden and Frentzen, 1988; Ohlrogge and Browse, 1995). Though there have been a number of studies in 16:3 plants revealing that the adjustment of two pathways and a flow of eukaryotic pathway derived precursors to chloroplast occurred in response to a wide range of stresses (Nakamura et al., 2009; Moellering and Benning, 2011; Higashi et al., 2015; Li et al., 2015), there are only a few reports on the lipid metabolism regulation in 18:3 plants (Li et al., 2015; Narayanan et al., 2016a,b). In this study, a combined lipidomic and transcriptomic analysis was conducted in18:3 plant maize under cold treatment (5°C), and the changes in glycerolipids contents and species and the transcriptional regulation of lipid metabolism related genes were investigated.

In our study, the activation of phospholipids degradation and the concurrent galactolipids synthesis under low temperature were observed at both biochemical and transcriptional levels. The lipidomic analysis revealed an increased PA and a decreased PC in the phospholipid class and an increased DGDG and a decreased MGDG in the galactolipids class, implying an elevated hydrolysis of PC to PA to provide precursors for galactolipids and an enhanced accumulation of DGDG – the end product of galactolipid synthesis pathway. DGDG has been reported to be the major chloroplast bilayer lipid, and plays important roles in maintaining the membrane integrity under stresses (Li et al., 2015; Lin et al., 2016; Zheng et al., 2016). The transcriptomic analysis of the maize leaf RNA-seq data identified over 200 differentially expressed genes (DEGs, Log2FC ≥1.5), and the genes involved in phospholipid and galactolipid synthesis pathways were significantly up-regulated, indicating an activated membrane lipids adjustment in maize leaf under cold. The changes in phospholipid metabolism were significant and confirmed by the results from both lipid content assay and transcriptomic data. The galactolipid metabolism, however, was not dramatically changed, which suggested that the activation of galactolipid metabolism at the biochemical level might lag behind that of transcriptome, which perhaps would occur after a longer term cold exposure-later than 3 days.

**FIGURE 5:**
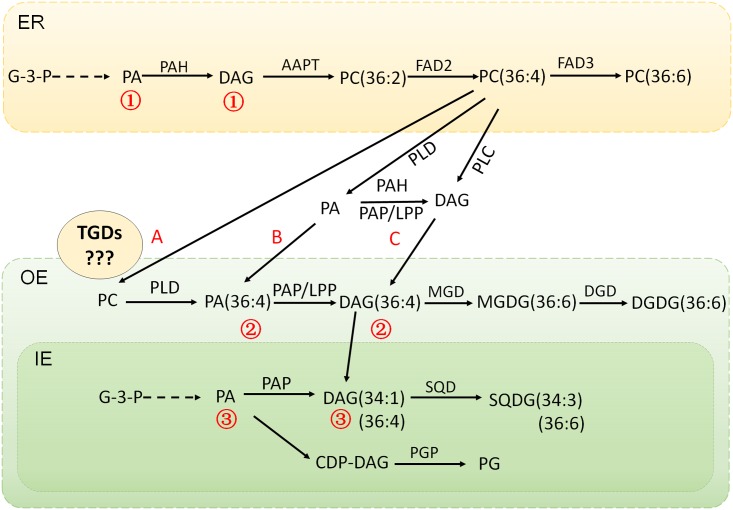
The proposed interaction and intermediates exchange between phospholipids pathway and galactolipids pathway in maize under low temperature stress. The *de novo* assembly of PA and DAG in endoplasmic reticulum and plastid produced different PA and DAG pools (the ER localized were marked as bbb172, and plastid localized were marked as bbb174) and PA and DAG generated from PC hydrolysis by phospholipases form a separated PA and DAG pool (marked as bbb173). A, B, C represents the proposed routes for the lipids trafficking between ER and chloroplast. ER, endoplasmic reticulum; OE, outer envelope; IE, inner envelope.

Based on the existing knowledge on the integrated lipid synthesis pathways compartmentalized in ER and chloroplast, a comprehensive gene-metabolite network has been generated (**Figure 4**). The proposed interactions and intermediates exchange between ER phospholipids pathway and chloroplast galactolipids pathway were illustrated in **Figure 5**. The central lipids intermediates PA and DAG could be generated through multiple pathways in different sub-cellular localizations. The *de novo* assembly of PA and DAG in endoplasmic reticulum and plastid produced different PA and DAG pools (the ER localized were marked as 1, and plastid localized were marked as 3), and PA and DAG generated from PC hydrolysis by phospholipases form a separated PA and DAG pool (marked as 2). PA and DAG could also be generated by phospholipids (PC and PE) degradation, catalyzed by two types of phospholipases PLD and PLC. In plant, the PLD pathway generates PA directly through the hydrolysis of phospholipids PC (and PE), and the NPC (non-specific PLC) pathway generates DAG instead. In this study, the down-regulated NPCs and up-regulated transcripts of PLDs plus PAH/PAP/LPP suggested the PLD-PA pathway was the major contributor for providing DAG was precursor for galactolipids synthesis, and the lipidomic data suggested the C36:4 DAG produced from C36:4 PC by PLD and PAH/PAP/LPP was a preferred precursor for MGDG and DGDG production.

Although the pathways interactions and lipid traffic between different subcellular compartments have been proved in the previous studies, there are still some questions remaining on illustrating the whole picture of the network, and one of the big unknowns is how the intermediates channeled from the ER phospholipid pathway to the chloroplast, and in what forms (Hurlock et al., 2014). As shown in **Figure 5**, the flow of intermediates from ER phospholipids to chloroplast galactolipids pathway could be accomplished through multiple means. In the proposed route A, it was suggested that PC itself was channeled to chloroplast, and the PC-PA-DAG conversion was taking place in chloroplast, which meant that the actions of PLDs and PAP/LPPs would be in the chloroplast. In the proposed route B, it was suggested that PA was channeled to the chloroplast, which meant the PC-PA process was taking place in ER/cytoplasm via the action of PLDs, while the PA-DAG conversion occurred in chloroplast catalyzed by PAP/LPPs. In the proposed route C, while DAG was the one for channeling, the PA-DAG process was taking place in ER/cytoplasm and catalyzed by the ER pathway PAH (or PAP/LPPs).

To clarify these lipid trafficking routes, the determination of the functional properties and subcellular localizations of the involving enzymes is pivotal. A primary prediction of the subcellular localizations and transmembrane domains was conducted on the genes in the proposed pathways (Supplementary Table S3 and **Figure 5**). All the maize PLDs analyzed had no transmembrane domains and did not seem to have distinct subcellular localizations. Two PAHs had no transmembrane domains, and cytoplasmic localization, which reflected the soluble nature of their *Arabidopsis* homologous genes AtPAH1&2 (Nakamura et al., 2007, 2009; Eastmond et al., 2010; Craddock et al., 2015). All the LPP type PAPs were predicted to be membrane-bound proteins harboring 5–7 transmembrane domains, and 3 of them were likely localized on the chloroplast/thylakoid membrane, including 2 PAP1/LPP1 and 1 PAP2/LPP2. In *Arabidopsis*, except the above mentioned soluble AtPAH1&2, there are a set of membrane bound LPPs, and some of them have been suggested to be plastidic PAP (Pierrugues et al., 2001; Nakamura et al., 2009). In18:3 plants, early studies suggested that the dysfunction of prokaryotic pathway was due to the degenerated weak plastid PAP activity (Frentzen et al., 1983; Heinz and Roughan, 1983). The later identification of the PA transporter TGD1, 2, 3 (trigalatosyldiacylglycerol) complex implicated that the PA derived from eukaryotic pathway could be channeled from ER to chloroplast and would have to be converted to DAG by the plastidic PAP (Benning, 2009). In our case, the putative plastidic PAP/LPPs were significantly up-regulated, thus we assumed that the plastid PAP might be able to work on the PA channeled from ER to chloroplast in18:3 plants.

The lipids transportation among different organelles often involves the assistance of transporters, such as the complex of TGD proteins in *Arabidopsis* which transport PA from ER to chloroplast (Wang et al., 2012; Manan et al., 2016). There were three TGDs orthologs annotated in our maize RNA-seq database and all of them were repressed under low temperature, which seemed to be a conflict with the PA transport scenario. In an earlier study, the major activity of MGDG synthesis was found in the outer envelope of chloroplast in the 18:3 plant pea (Cline and Keegstra, 1983), which suggested that the proposed TGD complex might not be necessary for galactolipids synthesis in 18:3 plants, though there was a TGD2 protein being identified in the envelope of plastid in pea (Brautigam et al., 2008; Benning, 2009). In *Arabidopsis*, PI-regulated DGDG accumulation did not seem to require the TGDs mediated ER-to-chloroplast lipid-trafficking, because the tgd1-1mutant shows no apparent defects in this process (Xu et al., 2010). The low-pi induced MGD2/MGD3 and DGD2 enzymes were also related to the outer envelope of chloroplast, which might be the reason for the independent of TGDs under pi deficiency (Shimojima and Ohta, 2011; Kelly et al., 2016). If the speculation was true that in the 18:3 plants, the synthesis of galactolipids MGDG and DGDG was occurring on the outer envelope of chloroplast, which did not involve the transport of lipid precursors across the double membrane system of the chloroplast, then another question would arise — how did the plastidic PAP/LPP get access to the ER-derived PA, as the plastidic PAP was localized on the inner side of the inner envelope of chloroplast (Nakamura et al., 2007). The precise localization of the PAP/LPP isoforms would need to be determined to find out whether the localization of the plastidic PAP/LPPs in 18:3 plant is different from the 16:3 *Arabidopsis* or only non-plastidic PAP/LPPs is involved in this process.

Plant lipid metabolism is a complex process involving multiple metabolic pathways and hundreds of related enzymes/genes, and the transcriptional coexpression of genes may reflect the functional relatedness (Peng and Weselake, 2011). The transcriptional modular regulation and coordinated gene expression have been observed in *Arabidopsis* in response to metabolic perturbations or environmental stress stimulus (Shen et al., 2010; Burgos et al., 2011; Szymanski et al., 2014; Li et al., 2015). In our study on the expression correlation of lipid related DEGs, a highly relevant gene expression cluster was observed harboring genes involved in phospholipid synthesis and degradation, as well as galactolipids synthesis, which was in agreement with the activation of these pathways under low temperature stress (Supplementary Figure S2). The study on transcriptional regulation of lipid metabolism has been scanty and only a few transcriptional regulators have been reported (Manan et al., 2016). The most well documented WRI1 – an important regulator of oil production and a member of the APETALA2 family of TFs, regulates the genes involved in FA biosynthesis through direct binding to AW box in their promoter regions in *Arabidopsis* and maize (Shen et al., 2010; Pouvreau et al., 2011). In this study, the maize WRI1 was found significantly reduced under low temperature, which might associate with the repressed genes expression in FA and TAG synthesis as shown in **Figure 4**.

Due to the lack of information on lipid metabolism regulation in18:3 plants, the current work have to be discussed based on the existing knowledge formulated mainly from studies on *Arabidopsis*, which is a 16:3 plant with a totally different mechanism. Although most of the results observed could be explained and fitted in the known biochemical routes governing different lipid synthesis pathways and their interactions, there are still some questions remaining and new hypothesis could be proposed and further investigations are needed. Further studies in 18:3 plants would concern the functional characterization of the lipid-related enzymes; determination of the localization and membrane topology of the specific proteins; the biological regulation of genes, pathways and networks; the trafficking means of lipids between different organelles; and the membrane lipids adjustment in response to various biotic and abiotic stresses.

## Author Contributions

YnG and LH designed and performed the experiments, and prepared the manuscript. FW, BY, and YqG prepared the samples. CZ and ZL analyzed the data. JX and KY conceived the experiments and revised the manuscript.

## Conflict of Interest Statement

The authors declare that the research was conducted in the absence of any commercial or financial relationships that could be construed as a potential conflict of interest. The reviewer TWTR and handling Editor declared their shared affiliation.

## Abbreviations

DAG: diacylglycerol
DGDG: digalactosyldiacylglycerol
MGDG: monogalactosyldiacylglycerol
PA: phosphatidic acid
PC: phosphatidylcholine
PE: phosphatidylethanolamine
PI: phosphatidylinositol
PLC: phospholipase C
PLD: phospholipase D
PS: phosphatidylserine
SQDG: sulfoquinovosyl diacylglycerol.
