# Supplementary material for: Biochemical and Transcriptional Regulation of Membrane Lipid Metabolism in Maize Leaves under Low Temperature
Source: Front Plant Sci. 2017 Nov 30;8:2053. doi: 10.3389/fpls.2017.02053 (PMC5714865; doi:10.3389/fpls.2017.02053)
Supplement: Supplementary file 1 [file Table_1.DOCX]

# Supplementary Tables

**SupplementalTable 1. Primers used for quantitive real-time PCR**

| Gene ID | Name | Primers |
| --- | --- | --- |
|  | *18s rRNA-F* | 5'-AGTTTGAGGCAATAACAGGTCT-3' |
|  | *18s rRNA-R* | 5'-GATGAAATTTCCCAAGATTACC-3' |
| GRMZM2G155357 | *qZmPECT-F* | 5'-CCTGCCGTTATGTTGATG-3' |
|  | *qZmPECT-R* | 5'-TTGGGCTCCTGATTGTCT-3' |
| GRMZM2G092588 | *qZmDGD1-F* | 5'-GTCAGATGTGGTGGATGG-3' |
|  | *qZmDGD1-R* | 5'-TGTTGAACTTGAGGAGGC-3' |
| GRMZM2G160452 | *qZmDGD2-F* | 5'-TGTGCCAATCACCCGTCAA-3' |
|  | *qZmDGD2-R* | 5'-TTTCAGTCGCAGCGTCCC-3' |
| GRMZM2G178892 | *qZmMGD3-F* | 5'-AAACCCAAATGGAGAAGTG-3' |
|  | *qZmMGD3-R* | 5'-TCTGAATCTGGACCAAACC -3' |
| GRMZM2G124335 | *qZmFAB-F* | 5'-TTCGCCAGAACCCTCAAC-3' |
|  | *qZmFAB-R* | 5'-AACGCCACCTTCACCTCA-3' |
| GRMZM2G128971 | *qZmFAD8-F* | 5'-TTATCTGCGTGGAGGACTGA-3' |
|  | *qZmFAD8-R* | 5'-CGACTTCTTCGGCTCTTTGT-3' |
