# Supplementary material for: Biochemical and Transcriptional Regulation of Membrane Lipid Metabolism in Maize Leaves under Low Temperature
Source: Front Plant Sci. 2017 Nov 30;8:2053. doi: 10.3389/fpls.2017.02053 (PMC5714865; doi:10.3389/fpls.2017.02053)
Supplement: Supplementary file 2 [file Table_2.DOCX]

**Supplemental Table 2. The differentially expressed genes (DEGs) were further screened out from the total lipid related genes**

| **Serial Number** | **Name Abbreviations** | **Maize ID** | **Putative Function** | **Log2 Fold Chang**  **(5/22℃）** | ***Arabidopsis* ID** |
| --- | --- | --- | --- | --- | --- |
|  | **Phospholipid Pathway/DAG synthsis/ PC turnover TAG/ PC de novo synthesis** | | | |  |
| NO.1 | GPAT7 | GRMZM2G059637 | Glycerol-3-Phosphate Acyltransferase 7 | +3.16 | At5G06090 |
| NO.2 | GPAT7 | GRMZM2G169293 | Glycerol-3-Phosphate Acyltransferase 7 | +2.37 | At5G06090 |
| NO.3 | LPAT2 | GRMZM2G037104 | Lysophosphatidyl Acyltransferase2 | -2.28 | At3G57650 |
| NO.4 | LPAT5 | GRMZM2G135027 | Lysophosphatidyl Acyltransferase 5 | +2.27 | At3G18850 |
| NO.5 | DGAT2 | GRMZM2G042356 | Diacylglycerol Acyltransferase | -3.04 | At3G51520 |
| NO.6 | PDAT1 | GRMZM2G088291 | Phospholipid:Diacylglycerol Acyltransferase | -2.33 | At5G13640 |
| NO.7 | PDAT1 | GRMZM2G095763 | Phospholipid:Diacylglycerol Acyltransferase | -1.49 | At5G13640 |
| NO.8 | CEK4 | GRMZM2G469409 | Choline/Ethanolamine Kinase (P) | +2.88 | At2G26830 |
| NO.9 | CEK4 | GRMZM2G100333 | Choline/Ethanolamine | +2.83 | At2G26830 |
| NO.10 | CCT2 | GRMZM2G132898 | CTP : Phosphocholine Cytidyltransferase € | +4.50 | At4G15130 |
| NO.11 | AAPT1 | GRMZM2G701058 | CDP-Choline : Diacylglycerol Phosphocholinetransferase € | -0.12 | At1G13560 |
|  | **PC Turnover & DAG Formation** | | |  |  |
| NO.12 | PLA2a | GRMZM5G865811 | Phospholipase A | -2.56 | At2G26560 |
| NO.13 | PLA2a | GRMZM2G349749 | Phospholipase A | +2.31 | At2G26560 |
| NO.14 | PLA2b | GRMZM2G045294 | Phospholipase A | -2.91 | At2G19690 |
| NO.15 | LPEAT2 | GRMZM2G116243 | Lysophosphatidyl Choline Acyltransferase (E) | +2.50 | At2G45670 |
| NO.16 | PLDα | GRMZM2G054559 | Phospholipase D | +3.31 | At3G15730 |
| NO.17 | PLDα | GRMZM2G061969 | Phospholipase D | +1.03 | At3G15730 |
| NO.18 | PLDα | GRMZM2G019029 | Phospholipase D | +1.67 | At3G15730 |
| NO.19 | PLDα | GRMZM2G179792 | Phospholipase D | +2.45 | At4G00240 |
| NO.20 | PLDβ1 | Maize_newGene_3214 | Phospholipase D | -1.64 | At2G42010 |
| NO.21 | PLDβ2 | GRMZM2G133943 | Phospholipase D | +1.53 | At4G11840 |
| NO.22 | NPC1 | GRMZM2G116876 | Non Specific Phospholipase C | -2.21 | At1G07230 |
| NO.23 | NPC2 | GRMZM2G479112 | Non Specific Phospholipase C | -1.09 | At3G03520 |
| NO.24 | NPC3 | GRMZM2G422670 | Non Specific Phospholipase C | -3.24 | At3G03530 |
| NO.25 | NPC4 | GRMZM2G081719 | Non Specific Phospholipase C | -4.16 | At3G48610 |
| NO.26 | PAH1 | GRMZM2G099481 | Phosphatidic Acid Phosphatase | +3.08 | At3G09560 |
| NO.27 | PAH2 | GRMZM2G154366 | Phosphatidic Acid Phosphatase | +0.33 | At5G42870 |
| NO.28 | PAP1/  LPP1 | GRMZM2G024144 | Phosphatidic Acid Phosphatase | +6.24 | At2G01180 |
| NO.30 | PAP1/  LPP1 | GRMZM2G061568 | Phosphatidic Acid Phosphatase | +1.63 | At2G01180 |
| NO.29 | PAP2/  LPP2 | GRMZM2G447433 | Phosphatidic Acid Phosphatase | -2.14 | At1G15080 |
| NO.31 | PAP2/  LPP2 | GRMZM2G050658 | Phosphatidic Acid Phosphatase | +1.60 | At1G15080 |
| NO.32 | PAP2/  LPP2 | GRMZM2G024615 | Phosphatidic Acid Phosphatase | +1.52 | At1G15080 |
| NO.33 | LPP3 | GRMZM2G077187 | Phosphatidic Acid Phosphatase | +2.72 | At3G02600 |
|  | **Galactolipid Synthesis** | | |  |  |
| NO.34 | MGD1 | GRMZM2G142873 | Monogalactosyl Diacylglycerol Synthase 1 | +1.41 | At4G31780 |
| NO.35 | MGD2 | GRMZM2G141320 | Monogalactosyl Diacylglycerol Synthase 2 | +4.49 | At5G20410 |
| NO.36 | MGD3 | GRMZM2G178892 | Monogalactosyl Diacylglycerol Synthase 3 | +1 | At2G11810 |
| NO.37 | DGD1 | Maize_newGene_1953 | Digalactosyl Diacylglycero Synthase1 | +0.84 | At3G11670 |
| NO.38 | DGD2 | GRMZM2G092588 | Digalactosyl Diacylglycero Synthase1 | +1.4 | At4G00550 |
| NO.39 | SQD2 | GRMZM2G117153 | Sulfoquinovosyl Diacylglycerol Synthase1 | +1.33 | At5G01220 |
|  | **Fatty Acid Desaturation & Formation** | | |  |  |
| NO.40 | FAD2 | GRMZM2G056252 | Fatty Acid Desaturase | +2.75 | At3G12120 |
| NO.41 | FAD2 | GRMZM2G064701 | Fatty Acid Desaturase | +2.98 | At3G12120 |
| NO.42 | FAD3 | GRMZM2G354558 | Fatty Acid Desaturase | +2.22 | At2G29980 |
| NO.43 | FAD7 | GRMZM2G074401 | Fatty Acid Desaturase | +1.1 | At5G05580 |
| NO.44 | FAD8 | GRMZM2G128971 | Fatty Acid Desaturase | +2.22 | At5G05580 |
| NO.45 | FATB | GRMZM2G007489 | Fatty Acyl Acyl Carrier Thioesterase B | 2.73 | At1G08510 |
| NO.46 | FATB | GRMZM2G406603 | Fatty Acyl Acyl Carrier Thioesterase B | 3.65 | At1G08510 |
| NO.47 | LACS3 | GRMZM5G812228 | Acyl-Coa Synthetase€ | +2.67 | At1G64400 |
| NO.48 | DGL1 | GRMZM5G812425 | Plastic Acylase (E) | -5.67 | At1G05800 |
| NO.49 | DGL3 | GRMZM2G174860 | Plastic Acylase (E) | +3.32 | At4G16820 |
| NO.50 | DGL3 | GRMZM2G097704 | Plastic Acylase (E) | +1.80 |  |
| NO.51 | DGL3 | GRMZM2G058149 | Plastic Acylase (E) | +4.29 |  |
| NO.52 | DGL5 | GRMZM2G359904 | Plastic Acylase (E) | +5.09 | At1G06800 |
| NO.53 | DGL5 | GRMZM2G353444 | Plastic Acylase (E) | +1.08 |  |
