# Supplementary material for: Biochemical and Transcriptional Regulation of Membrane Lipid Metabolism in Maize Leaves under Low Temperature
Source: Front Plant Sci. 2017 Nov 30;8:2053. doi: 10.3389/fpls.2017.02053 (PMC5714865; doi:10.3389/fpls.2017.02053)
Supplement: Supplementary file 3 [file Table_3.DOCX]

**Supplemental Table 3.** **The subcellular localization and transmembrane structure prediction of related genes**

| **Name Abbreviations** | **Maize ID** | ***Arabidopsis* ID** | **Log2 Fold Change (5/22℃）** | **Subcellular Location Prediction** | **Predicted TMHs** |
| --- | --- | --- | --- | --- | --- |
| PLDα | GRMZM2G054559 | At3G15730 | +3.31 | Microbody (peroxisome) | 0 |
| PLDα | GRMZM2G061969 | At3G15730 | +1.03 | Microbody (peroxisome) | 0 |
| PLDα | GRMZM2G019029 | At3G15730 | +1.67 | Nucleus、Mitochondrial matrix space | 0 |
| PLDα | GRMZM2G179792 | At4G00240 | +2.45 | Nucleus | 0 |
| PLDβ1 | Maize_newGene_3214 | At2G42010 | -1.64 |  | 0 |
| PLDβ2 | GRMZM2G133943 | At4G11840 | +1.53 | Endoplasmic reticulum (membrane) | 0 |
| NPC1 | GRMZM2G116876 | At1G07230 | -2.21 | Cytoplasmic、nucleus | 0 |
| NPC2 | GRMZM2G479112 | At2G26870 | -1.09 | Cytoplasmic、lysosome (lumen) | 1 |
| NPC4 | GRMZM2G422670 | At3G03530 | -3.24 | Cytoplasmic、cytoplasm | 0 |
| NPC6 | GRMZM2G081719 | At3G48610 | -4.16 | Cytoplasmic、outside | 0 |
| NPC | GRMZM2G479112 | At2G26870 | -1.09 | Cytoplasmic、outside | 1 |
| MGD1 | GRMZM2G142873 | At4G31780 | +1.41 | Membrane bound Chloroplast、microbody (peroxisome) | 0 |
| MGD2 | GRMZM2G141320 | At5G20410 | +4.49 | Membrane bound Chloroplast、endoplasmic reticulum (membrane) | 0 |
| MGD3 | GRMZM2G178892 | At2G11810 | +1 | Membrane bound Chloroplast、mitochondrial matrix space | 0 |
| DGD1 | Maize_newGene_1953 | At3G11670 | +0.84 |  | 0 |
| DGD2 | GRMZM2G092588 | At4G00550 | +1.4 | Extracellular (Secreted)、mitochondrial matrix space | 0 |
| SQD2 | GRMZM2G117153 | At5G01220 | +1.33 | Extracellular (Secreted)、plasma membrane | 0 |
| PAH1 | GRMZM2G099481 | At3G09560 | +3.08 | Cytoplasmic、cytoplasm | 0 |
| PAH2 | GRMZM2G154366 | At5G42870 | +0.33 | Cytoplasmic、cytoplasm | 0 |
| PAP1/LPP1 | GRMZM2G024144 | At2G01180 | +6.24 | Plasma membrane、chloroplast thylakoid membrane | 6 |
| PAP1/LPP1 | GRMZM2G061568 | At2G01180 | +1.63 | chloroplast thylakoid membrane、 Plasma membrane | 6 |
| PAP2/LPP2 | GRMZM2G447433 | At1G15080 | -2.14 | chloroplast thylakoid membrane、 Plasma membrane | 5 |
| PAP2/LPP2 | GRMZM2G050658 | At1G15080 | +1.60 | Plasma membrane | 6 |
| PAP2/LPP2 | GRMZM2G024615 | At1G15080 | +1.52 | Plasma membrane | 7 |
| LPP3 | GRMZM2G077187 | At3G02600 | +2.72 | Plasma membrane | 6 |
