# Supplementary material for: Biochemical and Transcriptional Regulation of Membrane Lipid Metabolism in Maize Leaves under Low Temperature
Source: Front Plant Sci. 2017 Nov 30;8:2053. doi: 10.3389/fpls.2017.02053 (PMC5714865; doi:10.3389/fpls.2017.02053)
Supplement: Supplementary file 8 [file Image_1.PDF]

Supplementary Material

Biochemical and Transcriptional Regulation of Membrane Lipid Metabolism in Maize Leaves under Low Temperature

Yingnan Gu[1 2†], Lin He[1†], Changjiang Zhao[1], Feng Wang[1], Bowei Yan[1], Yuqiao Gao[1], Zuotong Li[1], Kejun Yang[1]*, Jingyu Xu[1]*

***Co-corresponding author:**

Kejun Yang:byndykj@163.com

Jingyu Xu: E-Mail: xujingyu2003@hotmail.com

1 Supplementary Figure

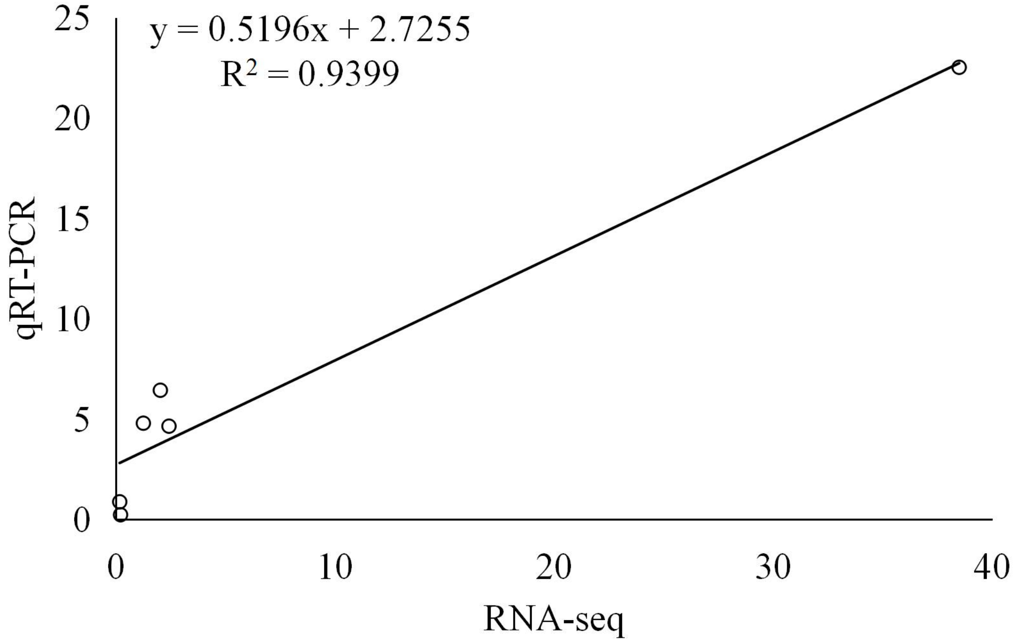

Supplementary Figure 1. Validation of RNA-seq expression through qRT-PCR. The plots demonstrate the expression ratio between 5°C Vs 22°C treatment in 2^. These gene include PECT-GRMZM2G155357; DGD-GRMZM2G092588; MGD2-GRMZM2G141320; NMT-GRMZM2G170400; FAB-GRMZM2G124335; FAD-GRMZM2G128971
