# Supplementary material for: Biochemical and Transcriptional Regulation of Membrane Lipid Metabolism in Maize Leaves under Low Temperature
Source: Front Plant Sci. 2017 Nov 30;8:2053. doi: 10.3389/fpls.2017.02053 (PMC5714865; doi:10.3389/fpls.2017.02053)
Supplement: Supplementary file 9 [file Image_2.PDF]

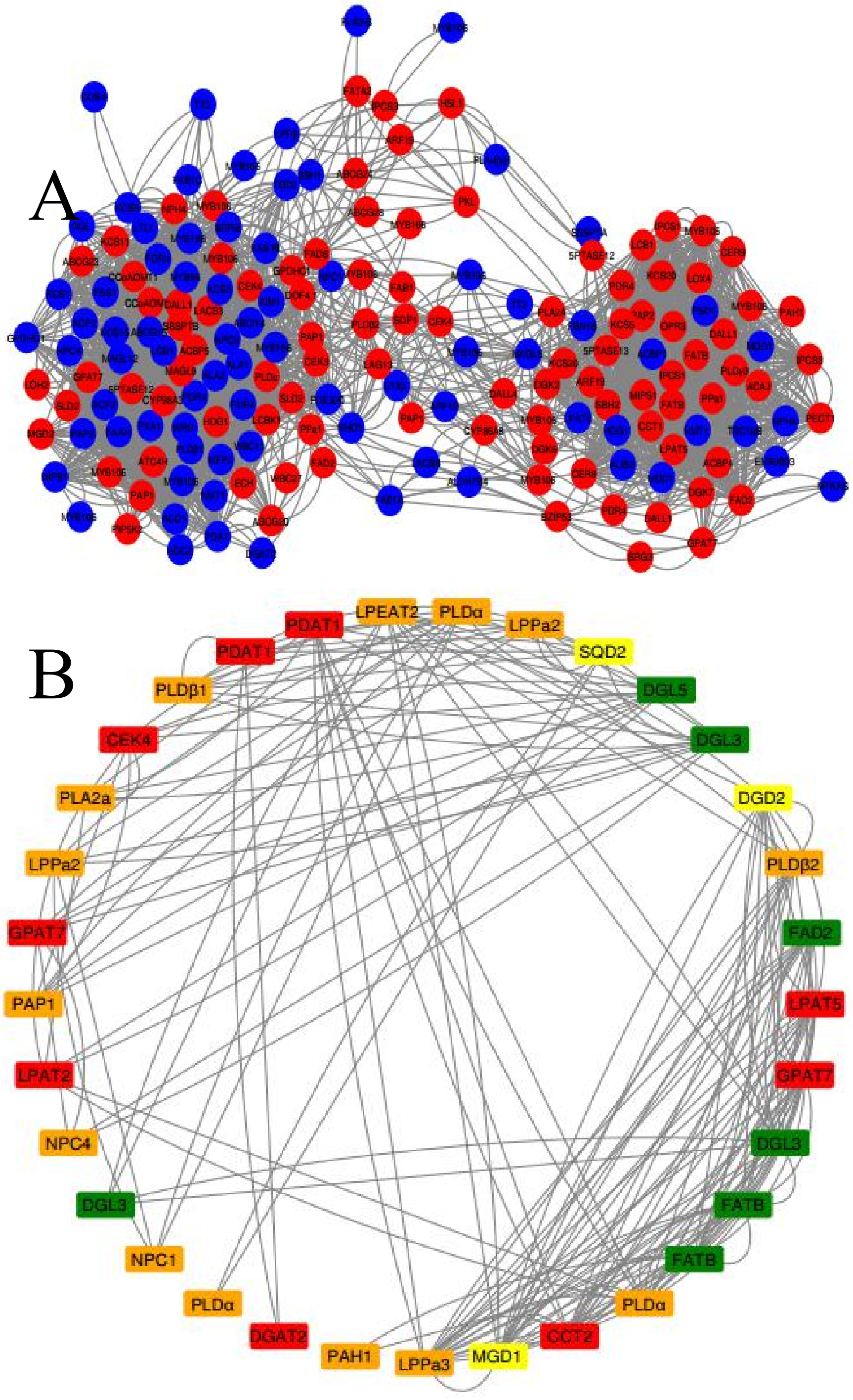

Supplementary Figure 2. Analysis of the expression correlation of lipid related DEGs and construction of co-expression networks in maize under low temperature stress. (A), Analysis of the expression correlation of lipid related DEGs (differentially expressed genes, Log2FC≥1.5 or ≤ -1.5), the red dots represent up-regulated genes and the blue dots represent down-regulated genes; (B), Analysis of the expression correlation of genes

involved in the major glycerolipids pathways,different categories of genes were differently coloured. DEGs the co-expression network was constructed for the data with a correlation greater than 0.9 using the cytoscape sofeware
