# Supplementary material for: Biochemical and Transcriptional Regulation of Membrane Lipid Metabolism in Maize Leaves under Low Temperature
Source: Front Plant Sci. 2017 Nov 30;8:2053. doi: 10.3389/fpls.2017.02053 (PMC5714865; doi:10.3389/fpls.2017.02053)
Supplement: Supplementary file 10 [file Image_3.PDF]

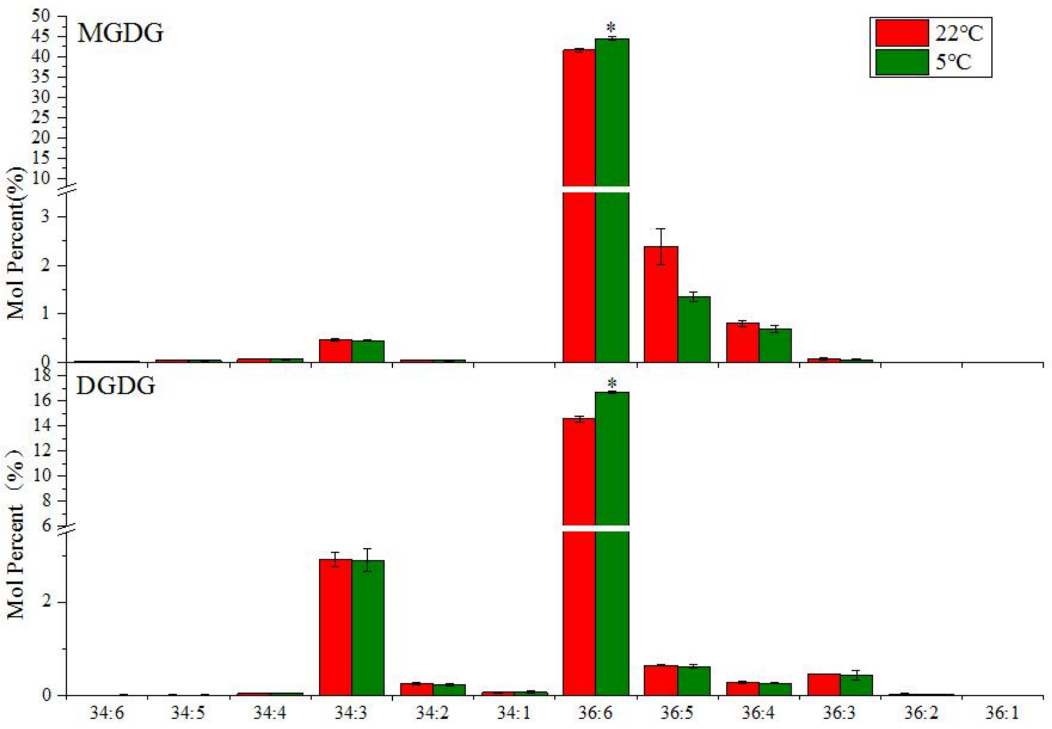

Supplementary Figure 3. Changes in diacyl lipid molecular species of monogalactosyldiacylglycerol (MGDG) and digalactosyldiacylglycerol (DGDG) in maize leaves under low temperature (5°C) in comparison to room temperature (22°C).Values (mol %) are means 5 ± standard deviation (SD)(n = 5). "*" indicated that the value was significantly different from the control. (P<0.05).
