# Supplementary material for: Biochemical and Transcriptional Regulation of Membrane Lipid Metabolism in Maize Leaves under Low Temperature
Source: Front Plant Sci. 2017 Nov 30;8:2053. doi: 10.3389/fpls.2017.02053 (PMC5714865; doi:10.3389/fpls.2017.02053)
Supplement: Supplementary file 11 [file Image_4.PDF]

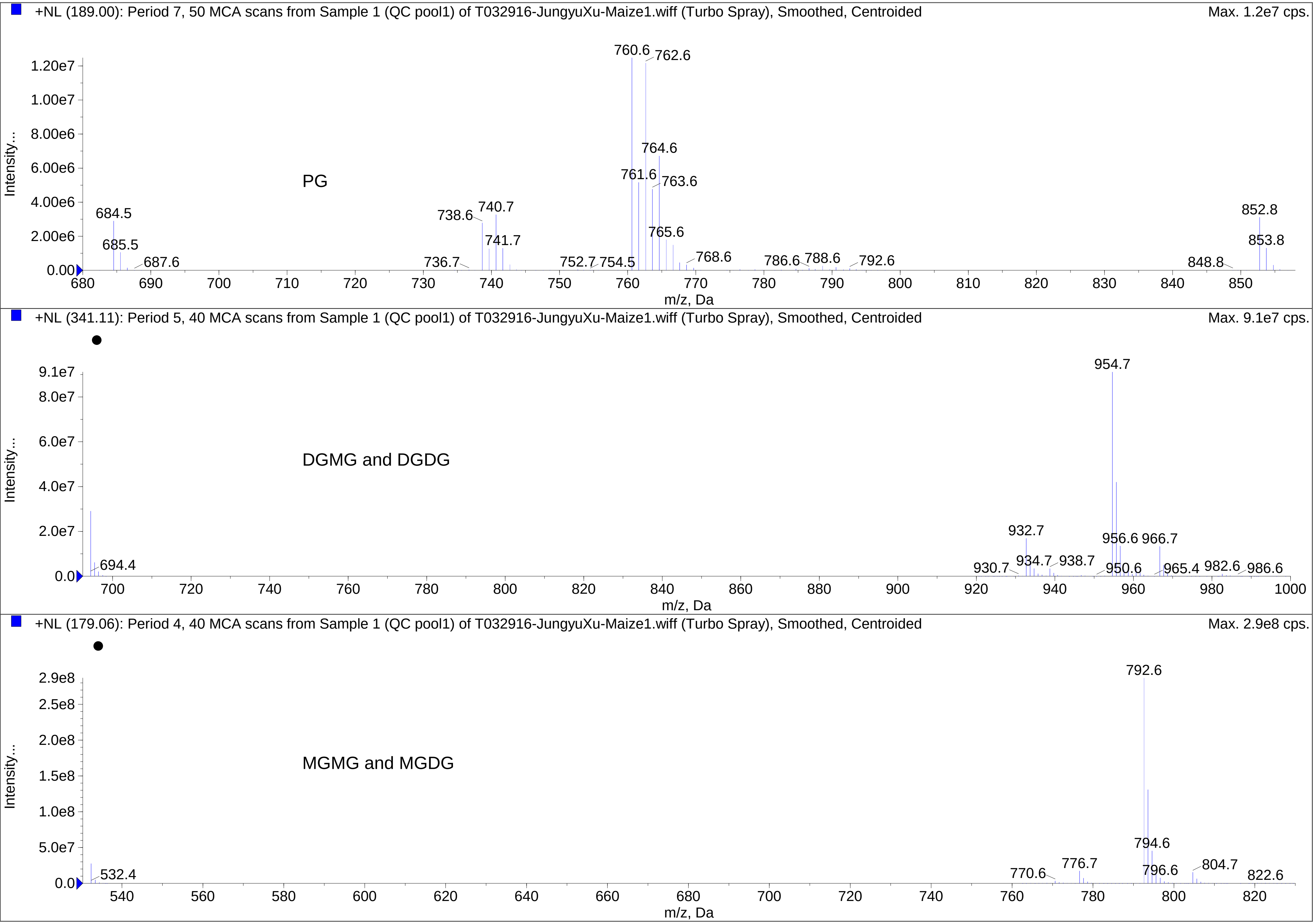
+NL (189.00): Period 7, 50 MCA scans from Sample 1 (QC pool1) of T032916-JungyuXu-Maize1.wiff (Turbo Spray), Smoothed, Centroided
Max. 1.2e7 cps.
PG
Intensity...
m/z, Da
760.6
762.6
764.6
761.6
763.6
765.6
768.6
738.6
740.7
741.7
736.7
752.7
754.5
684.5
685.5
687.6
786.6
788.6
792.6
852.8
853.8
848.8
+NL (341.11): Period 5, 40 MCA scans from Sample 1 (QC pool1) of T032916-JungyuXu-Maize1.wiff (Turbo Spray), Smoothed, Centroided
Max. 9.1e7 cps.
DGMG and DGDG
Intensity...
m/z, Da
954.7
932.7
956.6
966.7
934.7
938.7
930.7
950.6
965.4
982.6
986.6
694.4
+NL (179.06): Period 4, 40 MCA scans from Sample 1 (QC pool1) of T032916-JungyuXu-Maize1.wiff (Turbo Spray), Smoothed, Centroided
Max. 2.9e8 cps.
MGMG and MGDG
Intensity...
m/z, Da
792.6
794.6
776.7
796.6
804.7
770.6
822.6
532.4

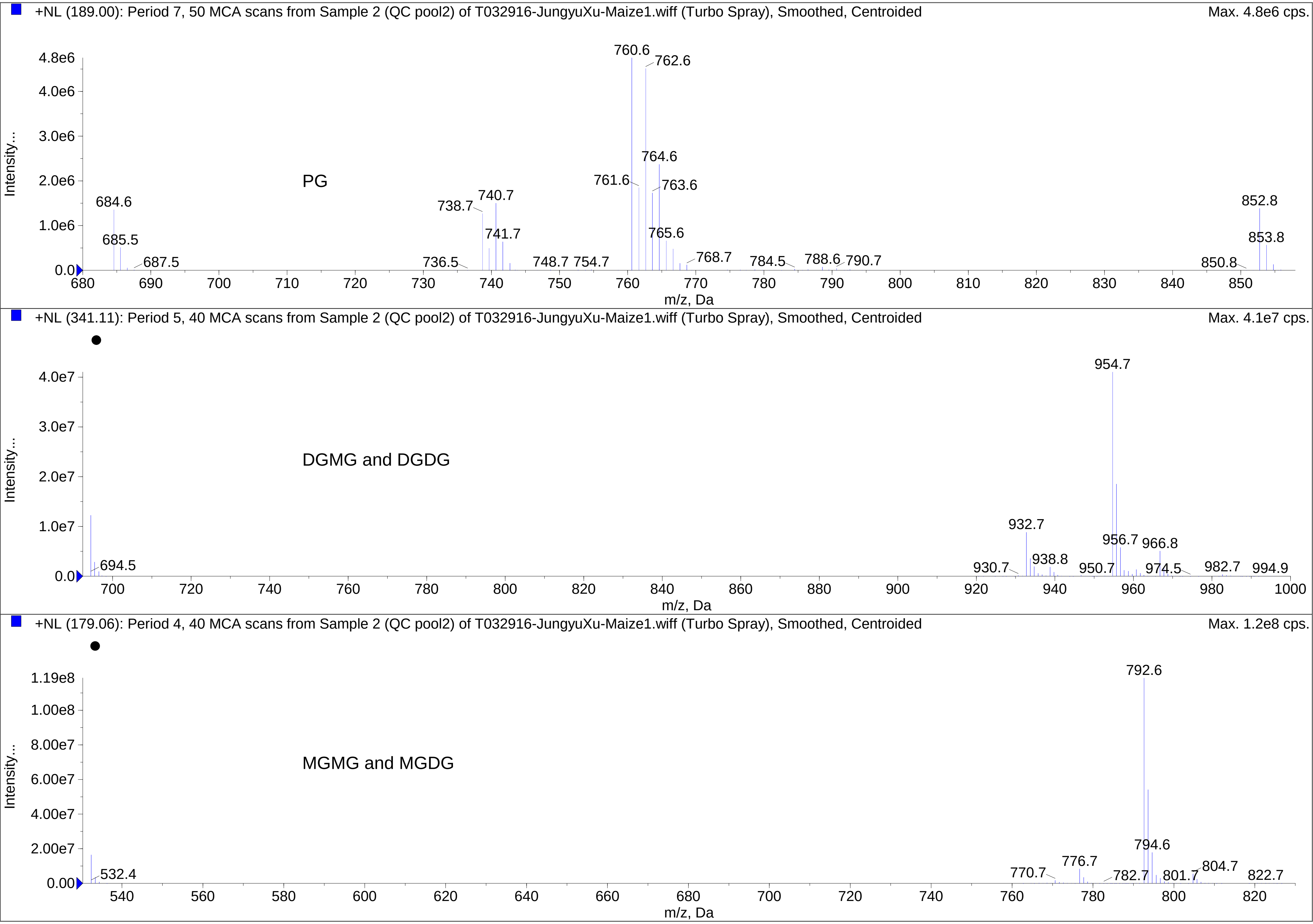

+NL (189.00): Period 7, 50 MCA scans from Sample 2 (QC pool2) of T032916-JungyuXu-Maize1.wiff (Turbo Spray), Smoothed, Centroided
Max. 4.8e6 cps.
PG
Intensity...
m/z, Da
760.6
762.6
764.6
761.6
763.6
740.7
738.7
741.7
765.6
684.6
685.5
687.5
736.5
748.7
754.7
768.7
784.5
788.6
790.7
852.8
853.8
850.8
+NL (341.11): Period 5, 40 MCA scans from Sample 2 (QC pool2) of T032916-JungyuXu-Maize1.wiff (Turbo Spray), Smoothed, Centroided
Max. 4.1e7 cps.
DGMG and DGDG
954.7
932.7
956.7
966.8
938.8
930.7
950.7
974.5
982.7
994.9
694.5
+NL (179.06): Period 4, 40 MCA scans from Sample 2 (QC pool2) of T032916-JungyuXu-Maize1.wiff (Turbo Spray), Smoothed, Centroided
Max. 1.2e8 cps.
MGMG and MGDG
792.6
794.6
776.7
770.7
782.7
801.7
804.7
822.7
532.4

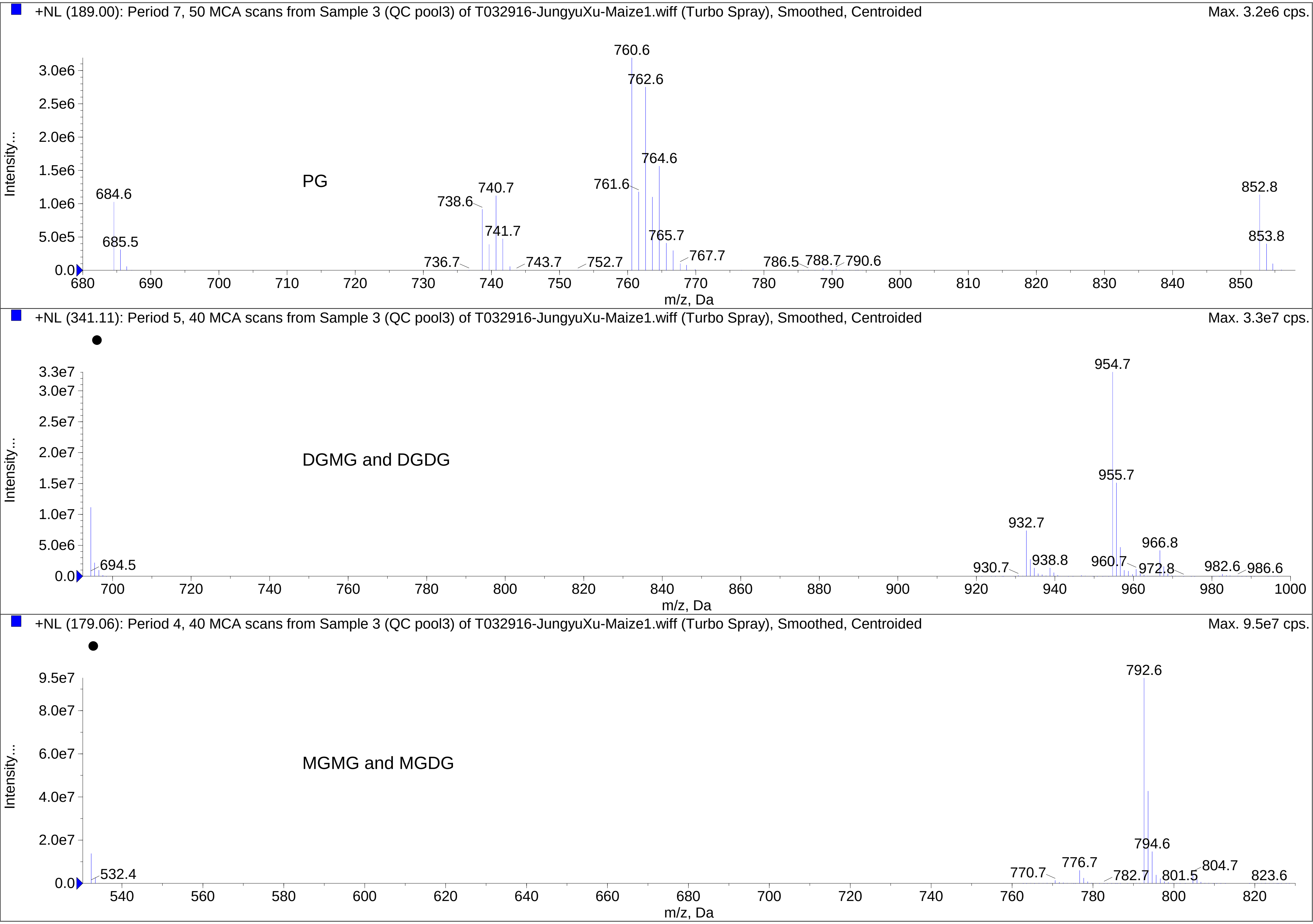
+NL (189.00): Period 7, 50 MCA scans from Sample 3 (QC pool3) of T032916-JungyuXu-Maize1.wiff (Turbo Spray), Smoothed, Centroided
Max. 3.2e6 cps.
PG
Intensity...
m/z, Da
760.6
762.6
764.6
761.6
740.7
738.6
684.6
852.8
685.5
741.7
765.7
853.8
767.7
736.7
743.7
752.7
786.5
788.7
790.6
+NL (341.11): Period 5, 40 MCA scans from Sample 3 (QC pool3) of T032916-JungyuXu-Maize1.wiff (Turbo Spray), Smoothed, Centroided
Max. 3.3e7 cps.
DGMG and DGDG
Intensity...
m/z, Da
954.7
955.7
932.7
966.8
938.8
960.7
972.8
930.7
694.5
982.6
986.6
+NL (179.06): Period 4, 40 MCA scans from Sample 3 (QC pool3) of T032916-JungyuXu-Maize1.wiff (Turbo Spray), Smoothed, Centroided
Max. 9.5e7 cps.
MGMG and MGDG
Intensity...
m/z, Da
792.6
794.6
776.7
804.7
770.7
782.7
801.5
823.6
532.4

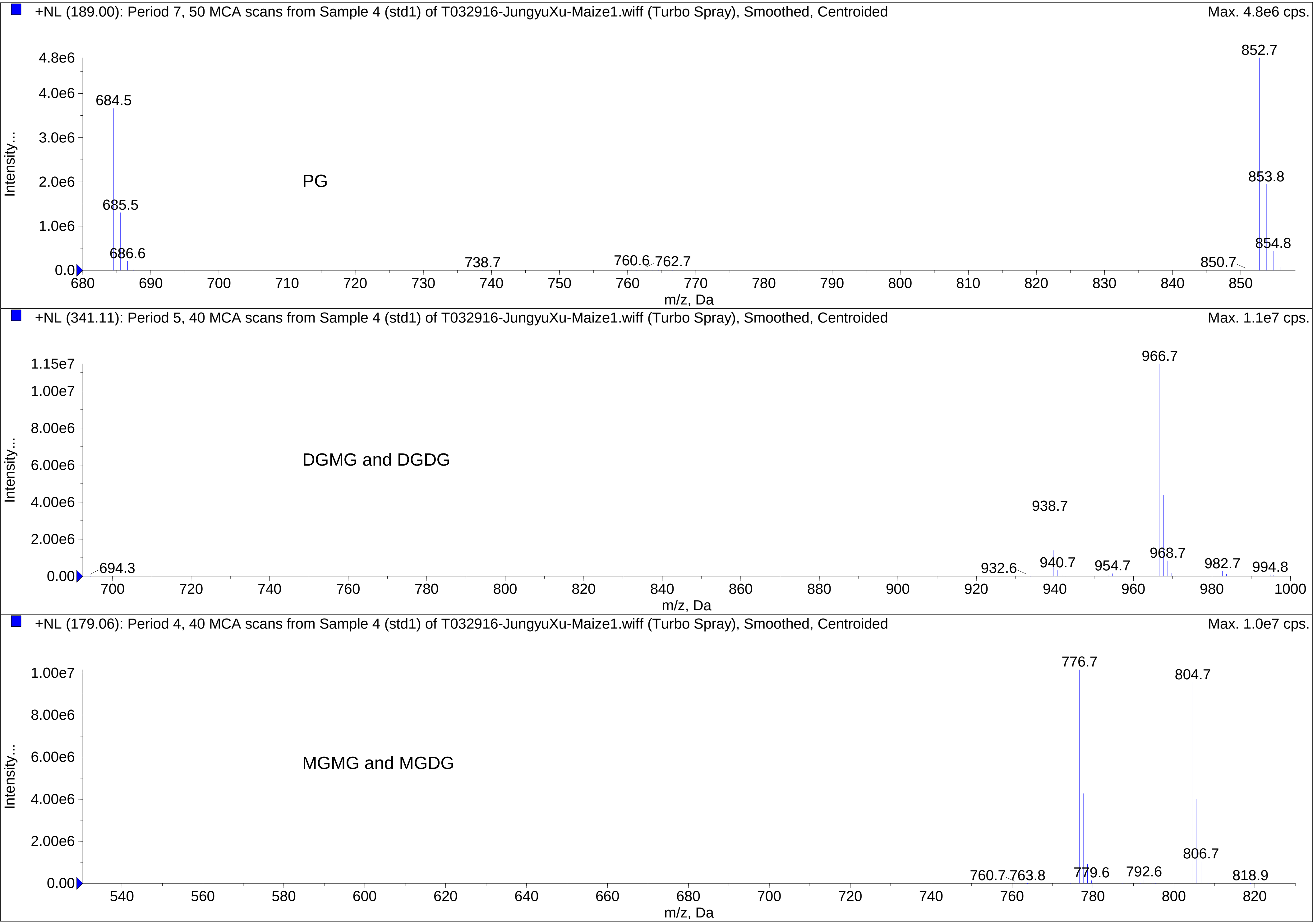
+NL (189.00): Period 7, 50 MCA scans from Sample 4 (std1) of T032916-JungyuXu-Maize1.wiff (Turbo Spray), Smoothed, Centroided
Max. 4.8e6 cps.
PG
684.5
685.5
686.6
738.7
760.6
762.7
850.7
852.7
853.8
854.8
Intensity...
m/z, Da
+NL (341.11): Period 5, 40 MCA scans from Sample 4 (std1) of T032916-JungyuXu-Maize1.wiff (Turbo Spray), Smoothed, Centroided
Max. 1.1e7 cps.
DGMG and DGDG
694.3
932.6
938.7
940.7
954.7
966.7
968.7
982.7
994.8
Intensity...
m/z, Da
+NL (179.06): Period 4, 40 MCA scans from Sample 4 (std1) of T032916-JungyuXu-Maize1.wiff (Turbo Spray), Smoothed, Centroided
Max. 1.0e7 cps.
MGMG and MGDG
760.7
763.8
776.7
779.6
792.6
804.7
806.7
818.9
Intensity...
m/z, Da

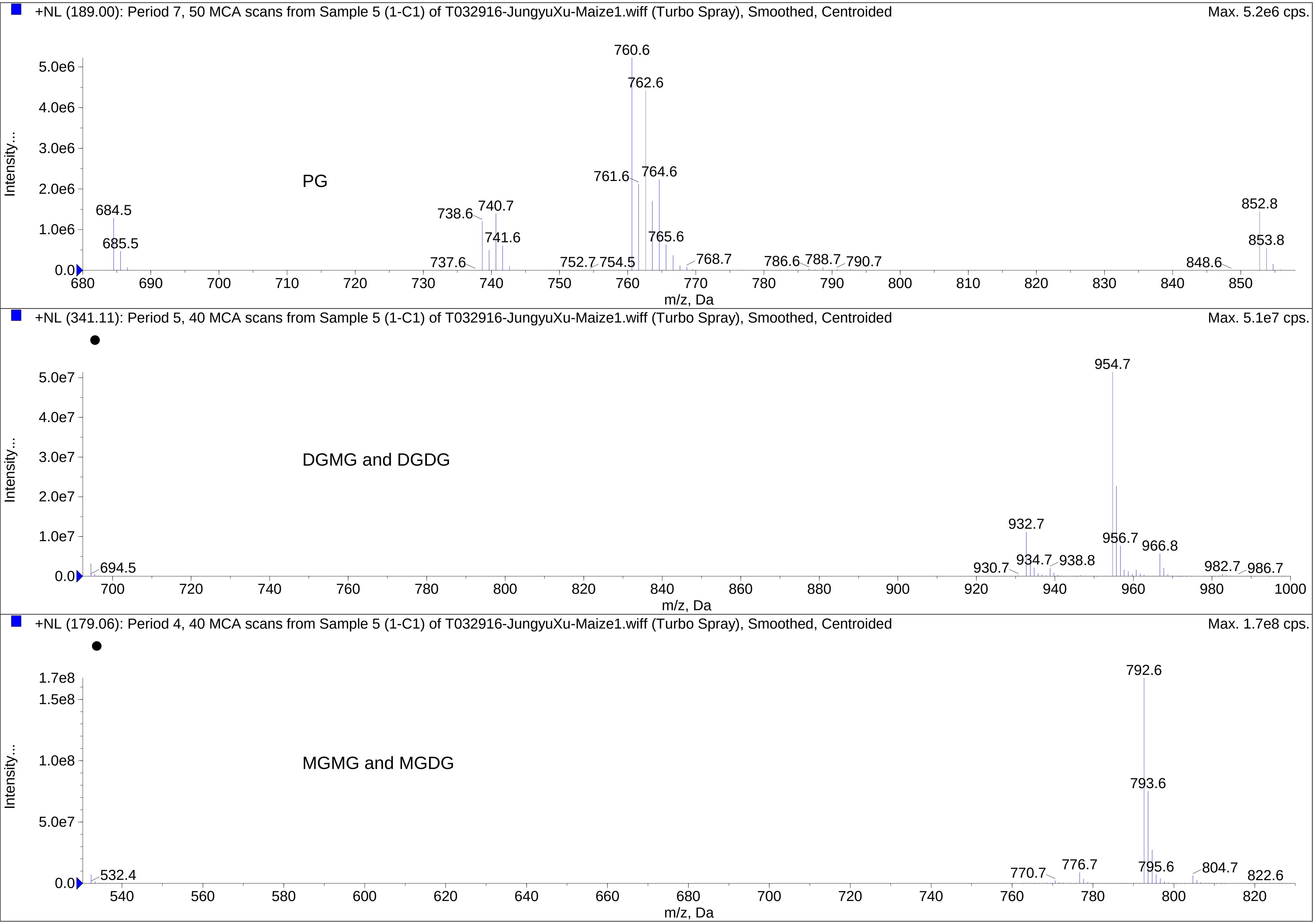

+NL (189.00): Period 7, 50 MCA scans from Sample 5 (1-C1) of T032916-JungyuXu-Maize1.wiff (Turbo Spray), Smoothed, Centroided
Max. 5.2e6 cps.
PG
Intensity...
m/z, Da
760.6
762.6
761.6
764.6
684.5
685.5
738.6
740.7
741.6
765.6
737.6
752.7
754.5
768.7
786.6
788.7
790.7
852.8
853.8
848.6
+NL (341.11): Period 5, 40 MCA scans from Sample 5 (1-C1) of T032916-JungyuXu-Maize1.wiff (Turbo Spray), Smoothed, Centroided
Max. 5.1e7 cps.
DGMG and DGDG
Intensity...
m/z, Da
954.7
932.7
956.7
966.8
934.7
938.8
930.7
694.5
982.7
986.7
+NL (179.06): Period 4, 40 MCA scans from Sample 5 (1-C1) of T032916-JungyuXu-Maize1.wiff (Turbo Spray), Smoothed, Centroided
Max. 1.7e8 cps.
MGMG and MGDG
Intensity...
m/z, Da
792.6
793.6
776.7
770.7
795.6
804.7
822.6
532.4

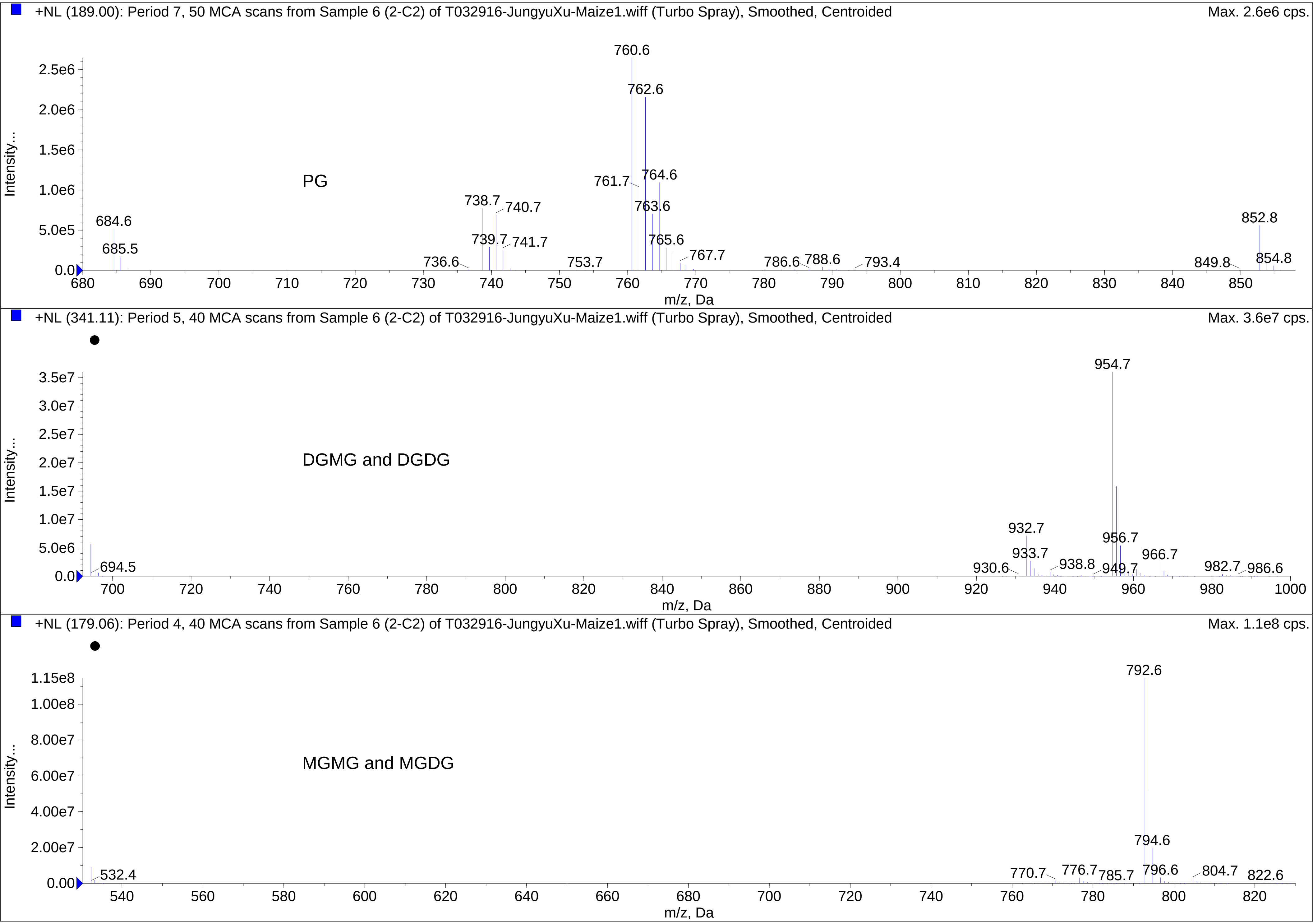
+NL (189.00): Period 7, 50 MCA scans from Sample 6 (2-C2) of T032916-JungyuXu-Maize1.wiff (Turbo Spray), Smoothed, Centroided
Max. 2.6e6 cps.
PG
760.6
762.6
761.7
764.6
763.6
765.6
767.7
738.7
740.7
739.7
741.7
736.6
753.7
684.6
685.5
786.6
788.6
793.4
849.8
852.8
854.8
Intensity...
m/z, Da
+NL (341.11): Period 5, 40 MCA scans from Sample 6 (2-C2) of T032916-JungyuXu-Maize1.wiff (Turbo Spray), Smoothed, Centroided
Max. 3.6e7 cps.
DGMG and DGDG
954.7
932.7
956.7
933.7
966.7
938.8
930.6
949.7
982.7
986.6
694.5
Intensity...
m/z, Da
+NL (179.06): Period 4, 40 MCA scans from Sample 6 (2-C2) of T032916-JungyuXu-Maize1.wiff (Turbo Spray), Smoothed, Centroided
Max. 1.1e8 cps.
MGMG and MGDG
792.6
794.6
796.6
804.7
822.6
770.7
776.7
785.7
532.4
Intensity...
m/z, Da

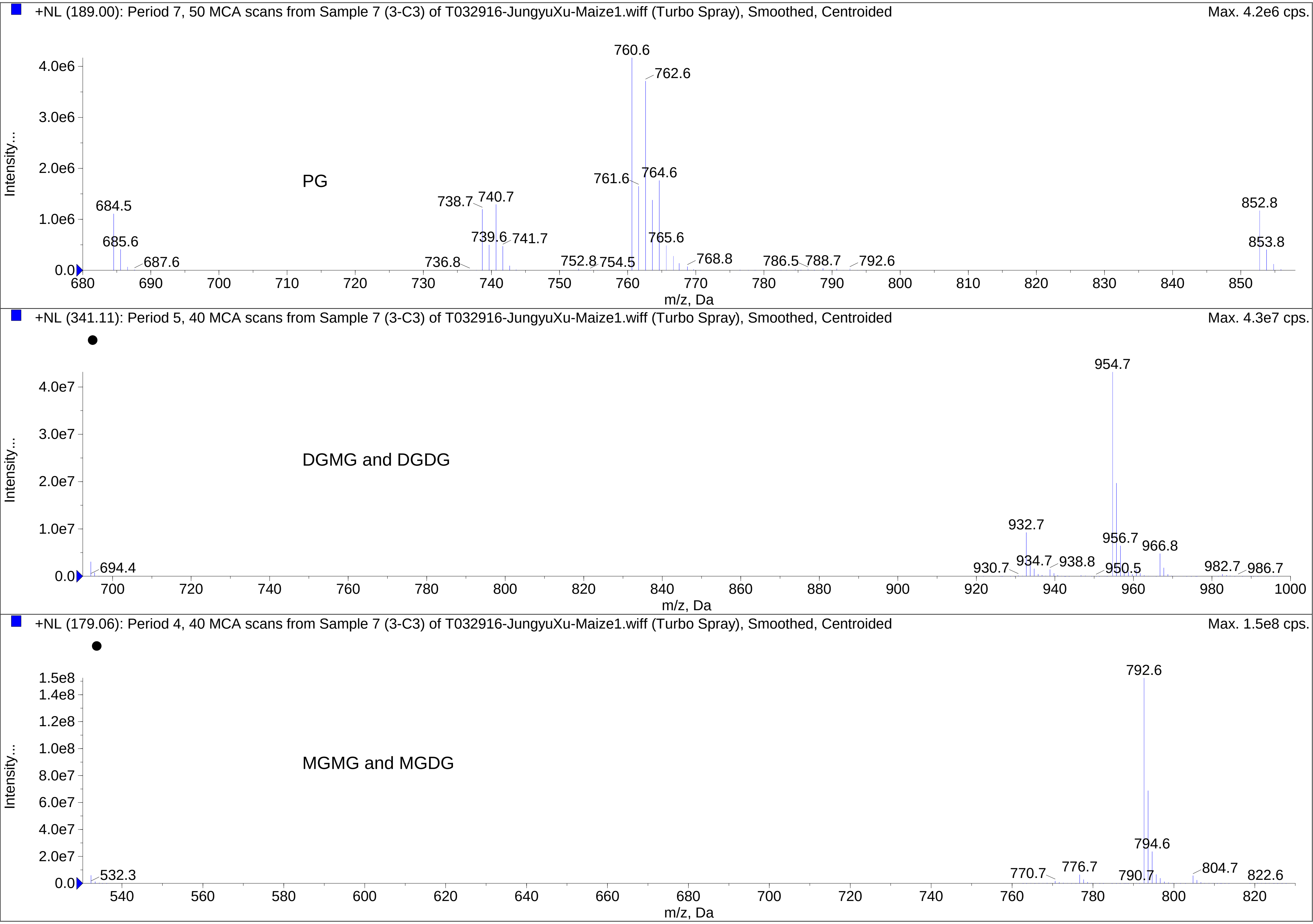

+NL (189.00): Period 7, 50 MCA scans from Sample 7 (3-C3) of T032916-JungyuXu-Maize1.wiff (Turbo Spray), Smoothed, Centroided
Max. 4.2e6 cps.
PG
Intensity...
m/z, Da
760.6
762.6
761.6
764.6
738.7
740.7
684.5
852.8
739.6
741.7
765.6
685.6
853.8
687.6
736.8
752.8
754.5
768.8
786.5
788.7
792.6
+NL (341.11): Period 5, 40 MCA scans from Sample 7 (3-C3) of T032916-JungyuXu-Maize1.wiff (Turbo Spray), Smoothed, Centroided
Max. 4.3e7 cps.
DGMG and DGDG
954.7
932.7
956.7
966.8
934.7
938.8
930.7
950.5
982.7
986.7
694.4
+NL (179.06): Period 4, 40 MCA scans from Sample 7 (3-C3) of T032916-JungyuXu-Maize1.wiff (Turbo Spray), Smoothed, Centroided
Max. 1.5e8 cps.
MGMG and MGDG
792.6
794.6
770.7
776.7
790.7
804.7
822.6
532.3

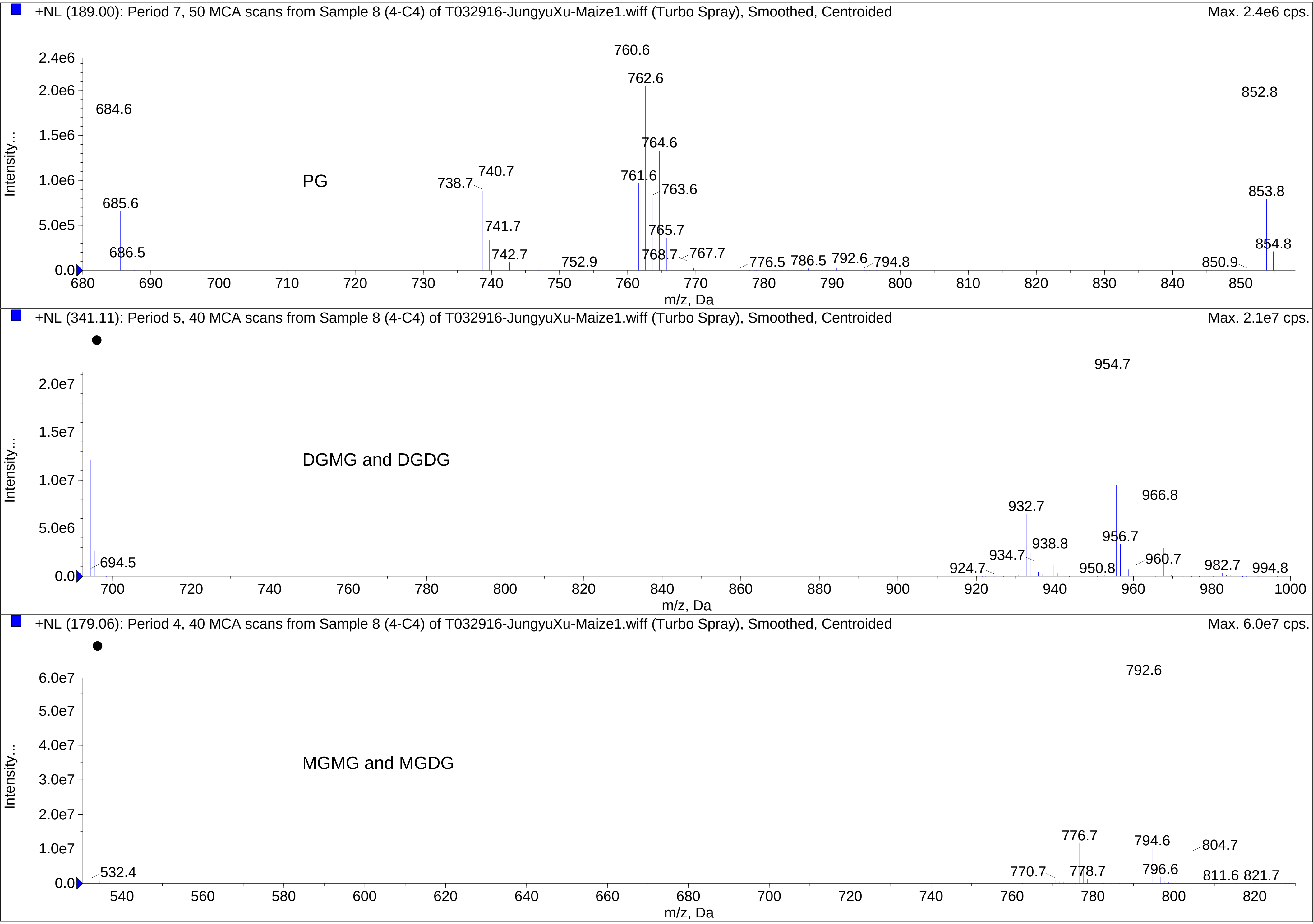

+NL (189.00): Period 7, 50 MCA scans from Sample 8 (4-C4) of T032916-JungyuXu-Maize1.wiff (Turbo Spray), Smoothed, Centroided
Max. 2.4e6 cps.
PG
Intensity...
m/z, Da
684.6
685.6
686.5
738.7
740.7
741.7
742.7
752.9
760.6
761.6
762.6
763.6
764.6
765.7
767.7
768.7
776.5
786.5
792.6
794.8
850.9
852.8
853.8
854.8
+NL (341.11): Period 5, 40 MCA scans from Sample 8 (4-C4) of T032916-JungyuXu-Maize1.wiff (Turbo Spray), Smoothed, Centroided
Max. 2.1e7 cps.
DGMG and DGDG
Intensity...
m/z, Da
694.5
924.7
932.7
934.7
938.8
950.8
954.7
956.7
960.7
966.8
982.7
994.8
+NL (179.06): Period 4, 40 MCA scans from Sample 8 (4-C4) of T032916-JungyuXu-Maize1.wiff (Turbo Spray), Smoothed, Centroided
Max. 6.0e7 cps.
MGMG and MGDG
Intensity...
m/z, Da
532.4
770.7
776.7
778.7
792.6
794.6
796.6
804.7
811.6
821.7

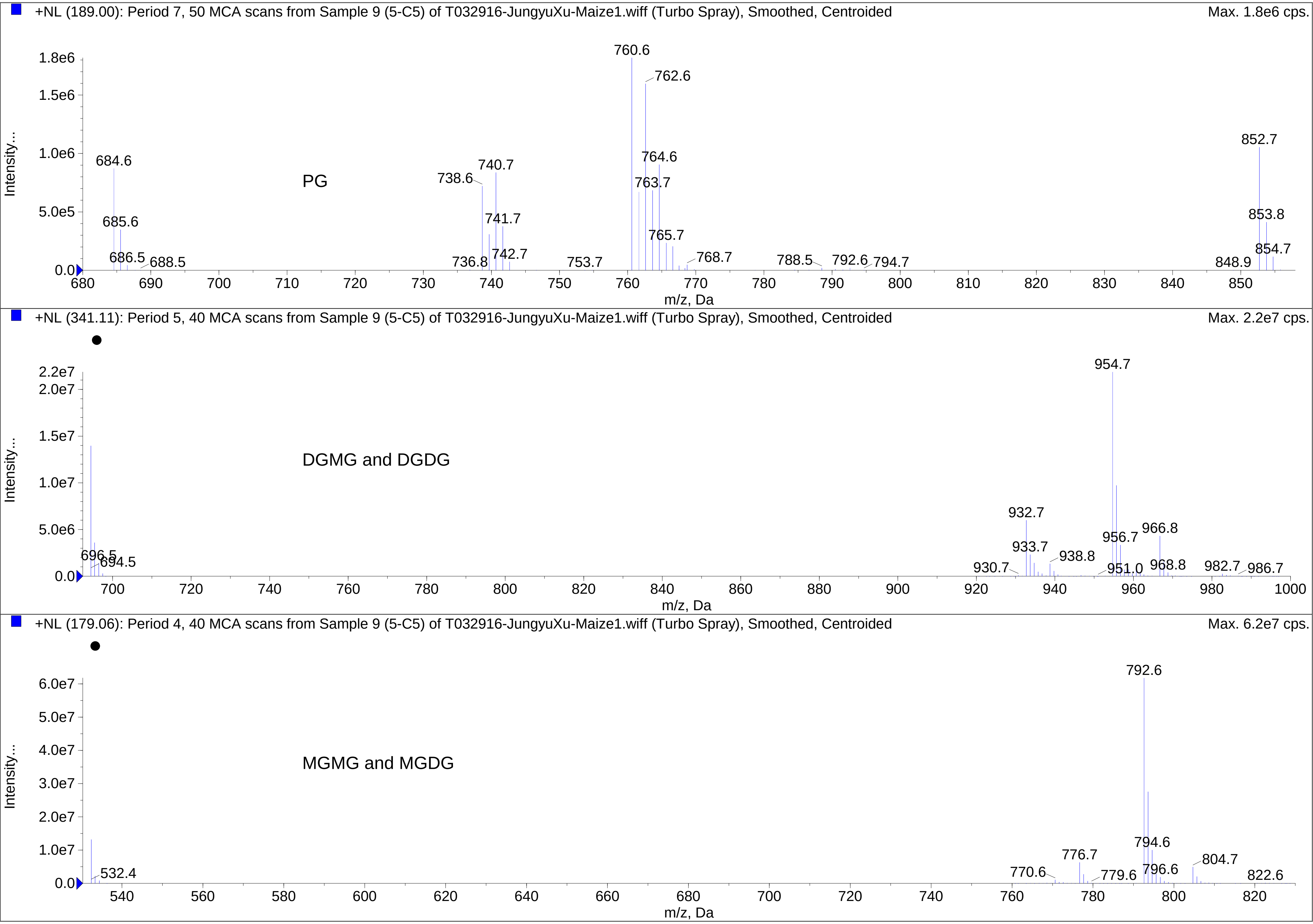

+NL (189.00): Period 7, 50 MCA scans from Sample 9 (5-C5) of T032916-JungyuXu-Maize1.wiff (Turbo Spray), Smoothed, Centroided
Max. 1.8e6 cps.
PG
Intensity...
m/z, Da
+NL (341.11): Period 5, 40 MCA scans from Sample 9 (5-C5) of T032916-JungyuXu-Maize1.wiff (Turbo Spray), Smoothed, Centroided
Max. 2.2e7 cps.
DGMG and DGDG
Intensity...
m/z, Da
+NL (179.06): Period 4, 40 MCA scans from Sample 9 (5-C5) of T032916-JungyuXu-Maize1.wiff (Turbo Spray), Smoothed, Centroided
Max. 6.2e7 cps.
MGMG and MGDG
Intensity...
m/z, Da

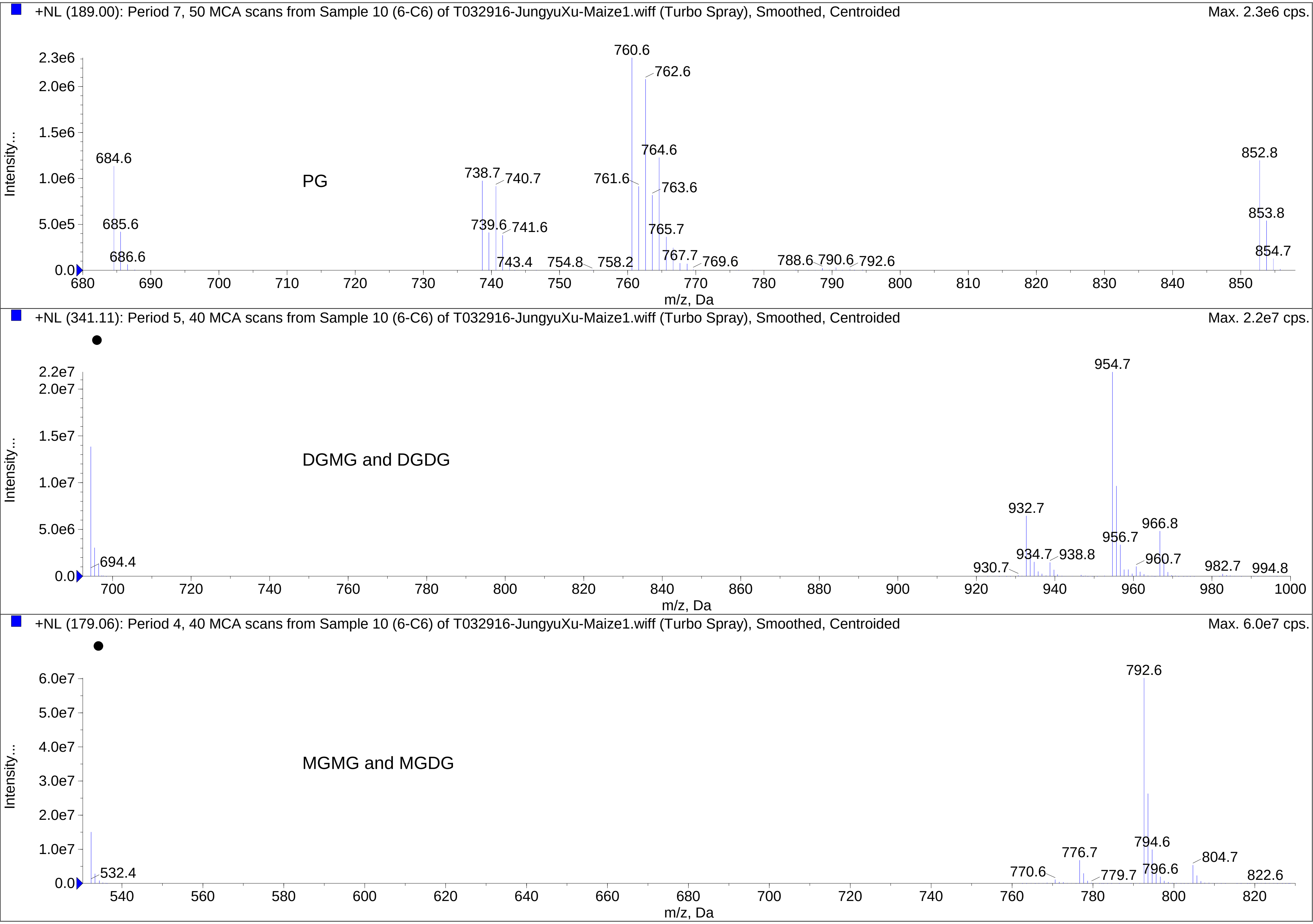

+NL (189.00): Period 7, 50 MCA scans from Sample 10 (6-C6) of T032916-JungyuXu-Maize1.wiff (Turbo Spray), Smoothed, Centroided
Max. 2.3e6 cps.
PG
760.6
762.6
764.6
763.6
761.6
738.7
740.7
739.6
741.6
684.6
685.6
686.6
743.4
754.8
758.2
765.7
767.7
769.6
788.6
790.6
792.6
852.8
853.8
854.7
Intensity...
m/z, Da
+NL (341.11): Period 5, 40 MCA scans from Sample 10 (6-C6) of T032916-JungyuXu-Maize1.wiff (Turbo Spray), Smoothed, Centroided
Max. 2.2e7 cps.
DGMG and DGDG
954.7
932.7
966.8
956.7
934.7
938.8
960.7
930.7
982.7
994.8
694.4
Intensity...
m/z, Da
+NL (179.06): Period 4, 40 MCA scans from Sample 10 (6-C6) of T032916-JungyuXu-Maize1.wiff (Turbo Spray), Smoothed, Centroided
Max. 6.0e7 cps.
MGMG and MGDG
792.6
794.6
776.7
804.7
770.6
779.7
796.6
822.6
532.4
Intensity...
m/z, Da

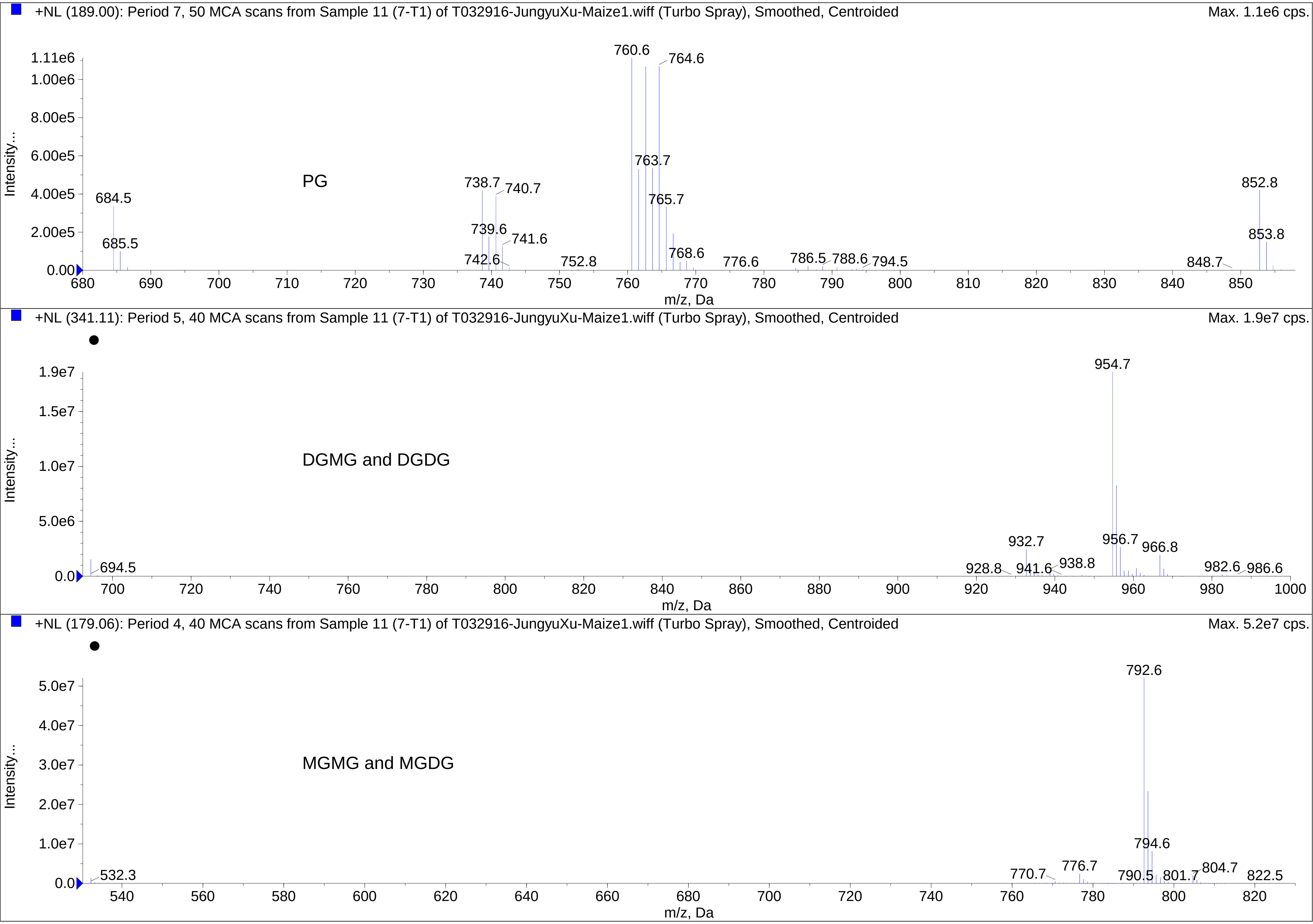

+NL (189.00): Period 7, 50 MCA scans from Sample 11 (7-T1) of T032916-JungyuXu-Maize1.wiff (Turbo Spray), Smoothed, Centroided
Max. 1.1e6 cps.
PG
Intensity...
m/z, Da
760.6
764.6
763.7
765.7
738.7
740.7
739.6
741.6
742.6
752.8
768.6
776.6
786.5
788.6
794.5
684.5
685.5
852.8
853.8
848.7
+NL (341.11): Period 5, 40 MCA scans from Sample 11 (7-T1) of T032916-JungyuXu-Maize1.wiff (Turbo Spray), Smoothed, Centroided
Max. 1.9e7 cps.
DGMG and DGDG
Intensity...
m/z, Da
954.7
932.7
956.7
966.8
928.8
941.6
938.8
982.6
986.6
694.5
+NL (179.06): Period 4, 40 MCA scans from Sample 11 (7-T1) of T032916-JungyuXu-Maize1.wiff (Turbo Spray), Smoothed, Centroided
Max. 5.2e7 cps.
MGMG and MGDG
Intensity...
m/z, Da
792.6
794.6
770.7
776.7
790.5
801.7
804.7
822.5
532.3

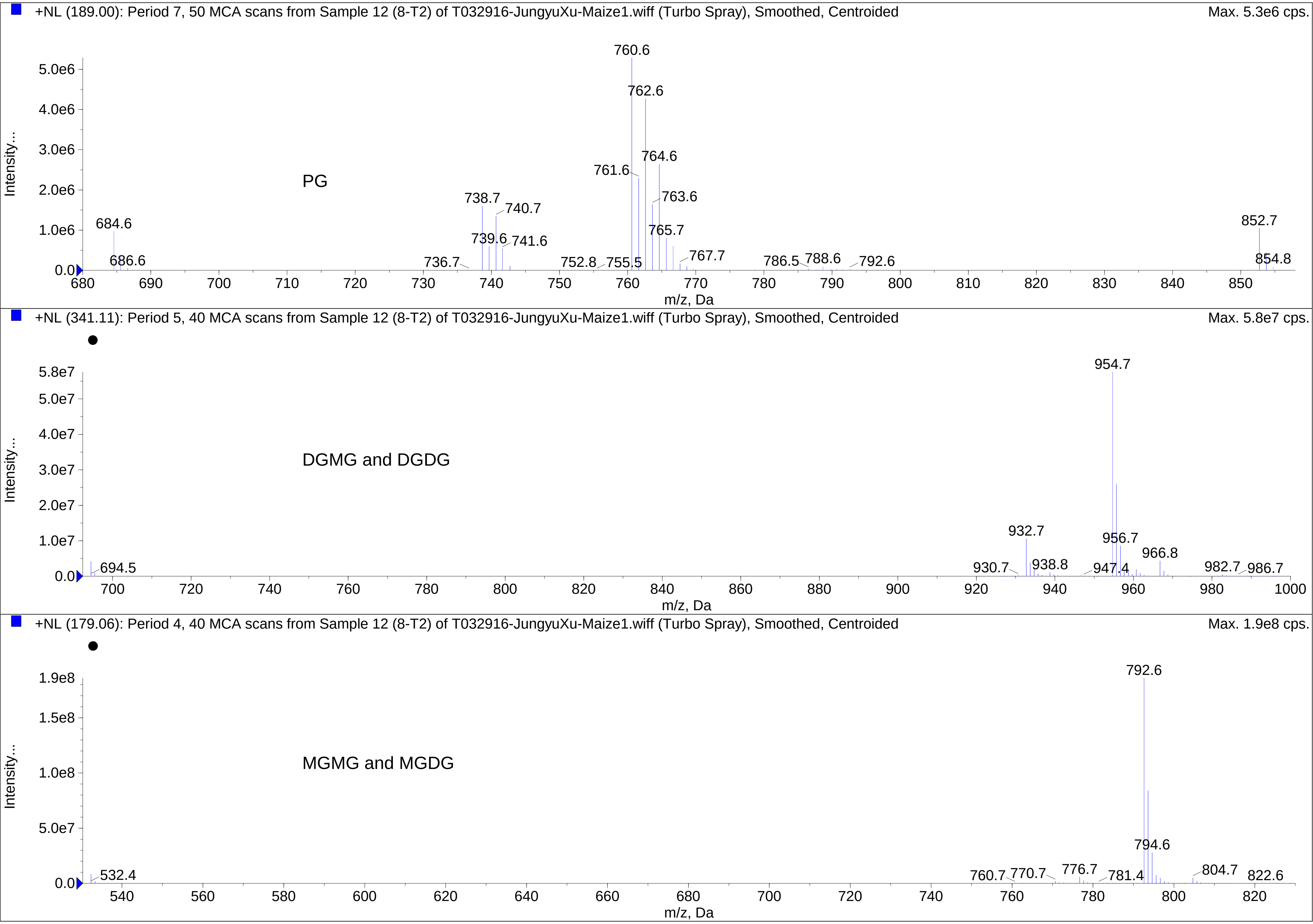

+NL (189.00): Period 7, 50 MCA scans from Sample 12 (8-T2) of T032916-JungyuXu-Maize1.wiff (Turbo Spray), Smoothed, Centroided
Max. 5.3e6 cps.
PG
760.6
762.6
764.6
761.6
763.6
765.7
767.7
738.7
740.7
739.6
741.6
684.6
686.6
736.7
752.8
755.5
786.5
788.6
792.6
852.7
854.8
Intensity...
m/z, Da
+NL (341.11): Period 5, 40 MCA scans from Sample 12 (8-T2) of T032916-JungyuXu-Maize1.wiff (Turbo Spray), Smoothed, Centroided
Max. 5.8e7 cps.
DGMG and DGDG
954.7
932.7
956.7
966.8
930.7
938.8
947.4
982.7
986.7
694.5
Intensity...
m/z, Da
+NL (179.06): Period 4, 40 MCA scans from Sample 12 (8-T2) of T032916-JungyuXu-Maize1.wiff (Turbo Spray), Smoothed, Centroided
Max. 1.9e8 cps.
MGMG and MGDG
792.6
794.6
532.4
760.7
770.7
776.7
781.4
804.7
822.6
Intensity...
m/z, Da

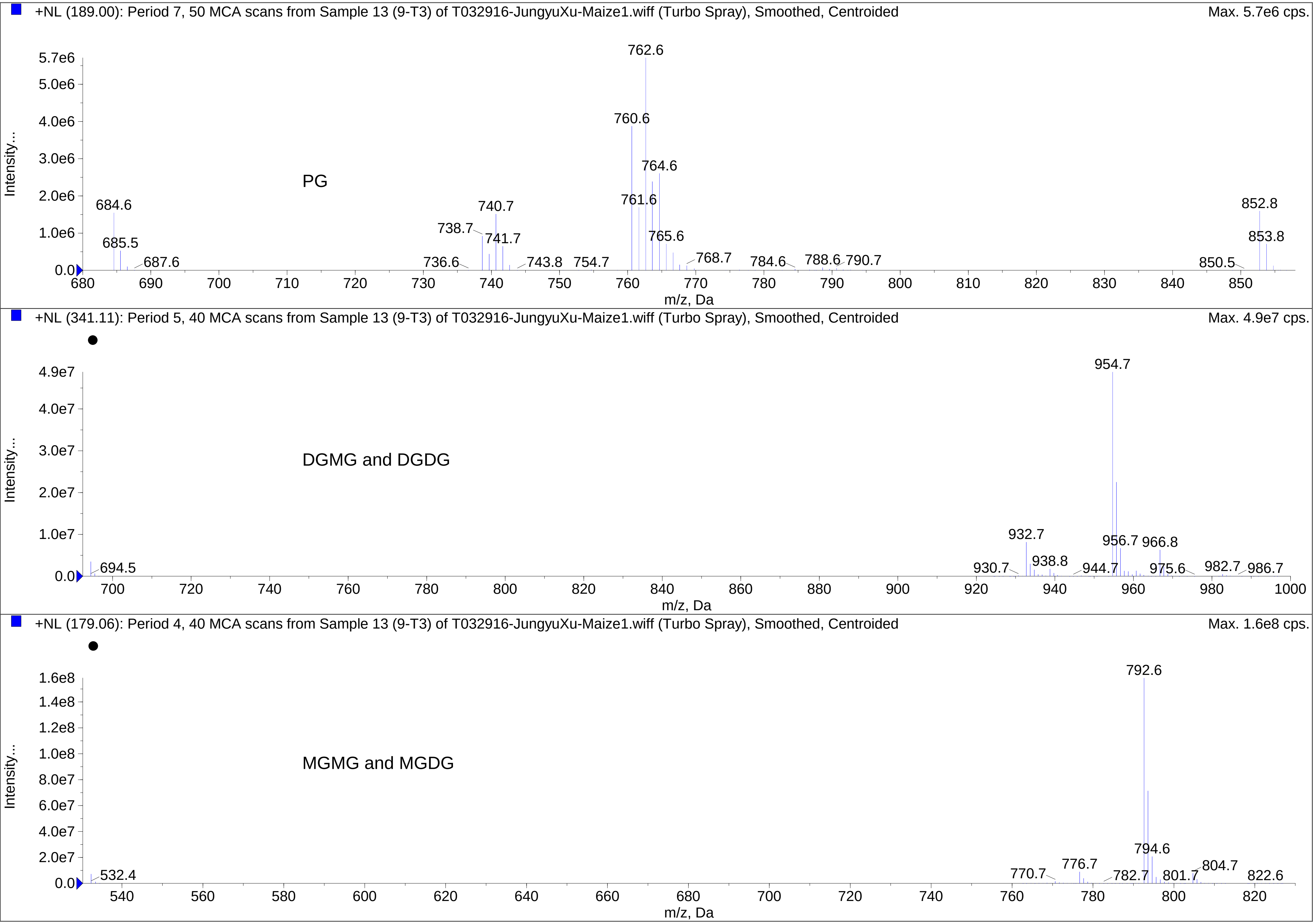
+NL (189.00): Period 7, 50 MCA scans from Sample 13 (9-T3) of T032916-JungyuXu-Maize1.wiff (Turbo Spray), Smoothed, Centroided
Max. 5.7e6 cps.
Intensity...
PG
762.6
760.6
764.6
761.6
684.6
740.7
852.8
738.7
741.7
685.5
765.6
853.8
687.6
736.6
743.8
754.7
768.7
784.6
788.6
790.7
850.5
m/z, Da
+NL (341.11): Period 5, 40 MCA scans from Sample 13 (9-T3) of T032916-JungyuXu-Maize1.wiff (Turbo Spray), Smoothed, Centroided
Max. 4.9e7 cps.
Intensity...
DGMG and DGDG
954.7
932.7
956.7
966.8
938.8
694.5
930.7
944.7
975.6
982.7
986.7
m/z, Da
+NL (179.06): Period 4, 40 MCA scans from Sample 13 (9-T3) of T032916-JungyuXu-Maize1.wiff (Turbo Spray), Smoothed, Centroided
Max. 1.6e8 cps.
Intensity...
MGMG and MGDG
792.6
794.6
776.7
804.7
770.7
782.7
801.7
822.6
532.4
m/z, Da

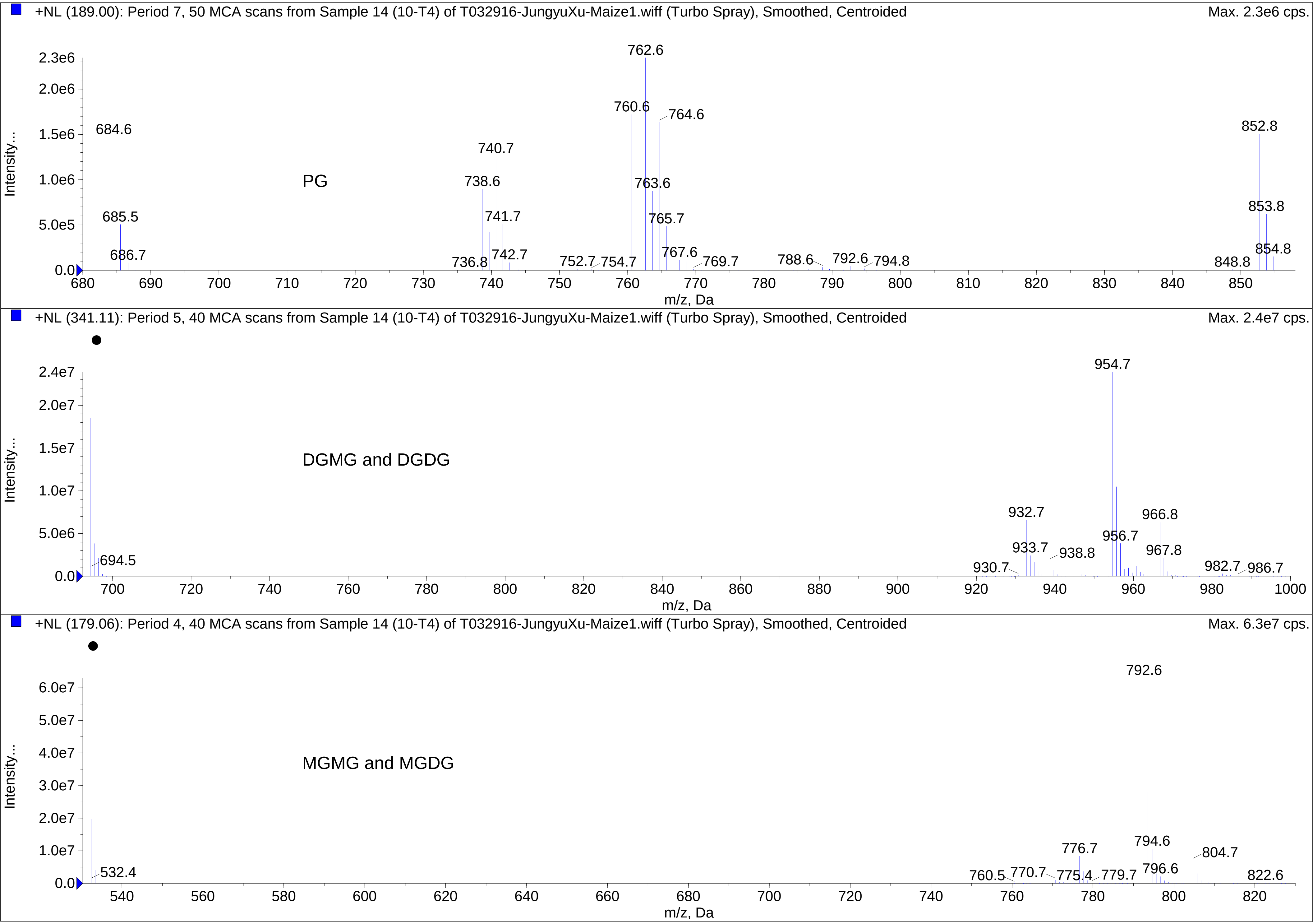
+NL (189.00): Period 7, 50 MCA scans from Sample 14 (10-T4) of T032916-JungyuXu-Maize1.wiff (Turbo Spray), Smoothed, Centroided
Max. 2.3e6 cps.
PG
Intensity...
2.3e6
2.0e6
1.5e6
1.0e6
5.0e5
0.0
684.6
685.5
686.7
736.8
738.6
740.7
741.7
742.7
752.7
754.7
760.6
762.6
763.6
764.6
765.7
767.6
769.7
788.6
792.6
794.8
848.8
852.8
853.8
854.8
680
690
700
710
720
730
740
750
760
770
780
790
800
810
820
830
840
850
m/z, Da
+NL (341.11): Period 5, 40 MCA scans from Sample 14 (10-T4) of T032916-JungyuXu-Maize1.wiff (Turbo Spray), Smoothed, Centroided
Max. 2.4e7 cps.
DGMG and DGDG
Intensity...
2.4e7
2.0e7
1.5e7
1.0e7
5.0e6
0.0
694.5
930.7
932.7
933.7
938.8
954.7
956.7
966.8
967.8
982.7
986.7
700
720
740
760
780
800
820
840
860
880
900
920
940
960
980
1000
m/z, Da
+NL (179.06): Period 4, 40 MCA scans from Sample 14 (10-T4) of T032916-JungyuXu-Maize1.wiff (Turbo Spray), Smoothed, Centroided
Max. 6.3e7 cps.
MGMG and MGDG
Intensity...
6.0e7
5.0e7
4.0e7
3.0e7
2.0e7
1.0e7
0.0
532.4
760.5
770.7
775.4
776.7
779.7
792.6
794.6
796.6
804.7
822.6
540
560
580
600
620
640
660
680
700
720
740
760
780
800
820
m/z, Da

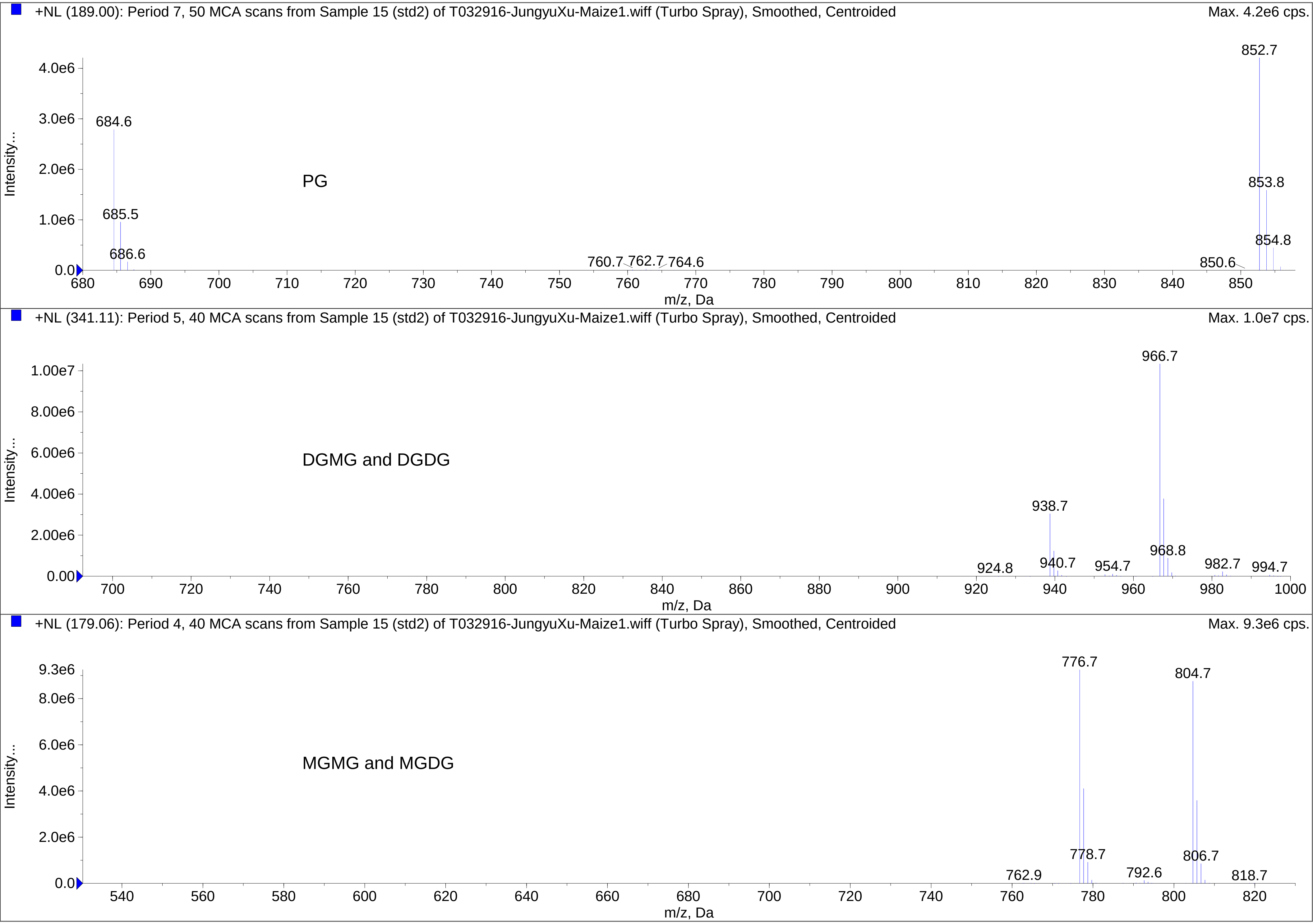
+NL (189.00): Period 7, 50 MCA scans from Sample 15 (std2) of T032916-JungyuXu-Maize1.wiff (Turbo Spray), Smoothed, Centroided
Max. 4.2e6 cps.
PG
Intensity...
m/z, Da
684.6
685.5
686.6
760.7
762.7
764.6
850.6
852.7
853.8
854.8
+NL (341.11): Period 5, 40 MCA scans from Sample 15 (std2) of T032916-JungyuXu-Maize1.wiff (Turbo Spray), Smoothed, Centroided
Max. 1.0e7 cps.
DGMG and DGDG
Intensity...
m/z, Da
924.8
938.7
940.7
954.7
966.7
968.8
982.7
994.7
+NL (179.06): Period 4, 40 MCA scans from Sample 15 (std2) of T032916-JungyuXu-Maize1.wiff (Turbo Spray), Smoothed, Centroided
Max. 9.3e6 cps.
MGMG and MGDG
Intensity...
m/z, Da
762.9
776.7
778.7
792.6
804.7
806.7
818.7

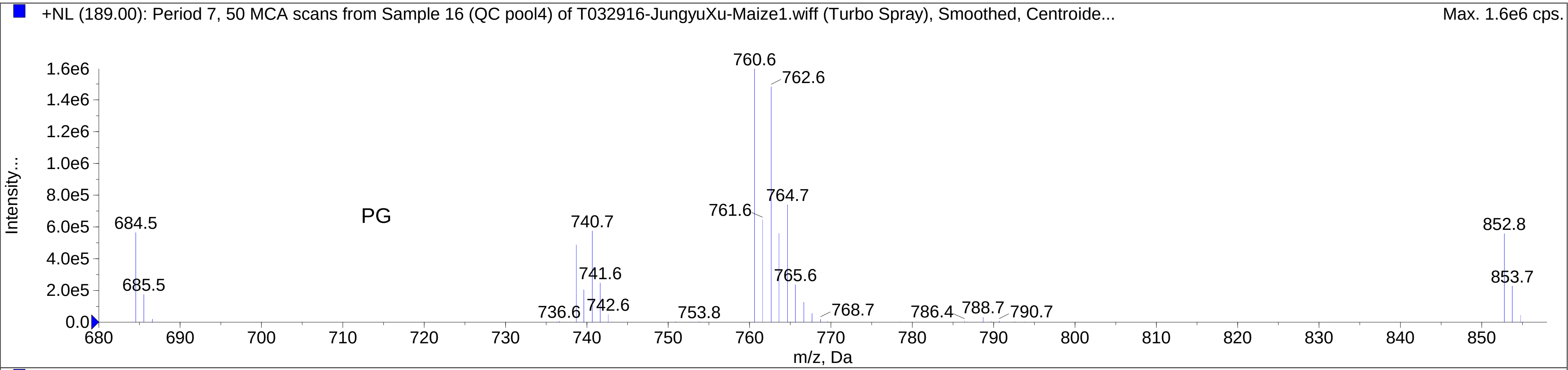
+NL (189.00): Period 7, 50 MCA scans from Sample 16 (QC pool4) of T032916-JungyuXu-Maize1.wiff (Turbo Spray), Smoothed, Centroide...
Max. 1.6e6 cps.
PG
Intensity...
m/z, Da
760.6
762.6
764.7
761.6
740.7
684.5
852.8
741.6
765.6
685.5
853.7
742.6
736.6
753.8
768.7
786.4
788.7
790.7

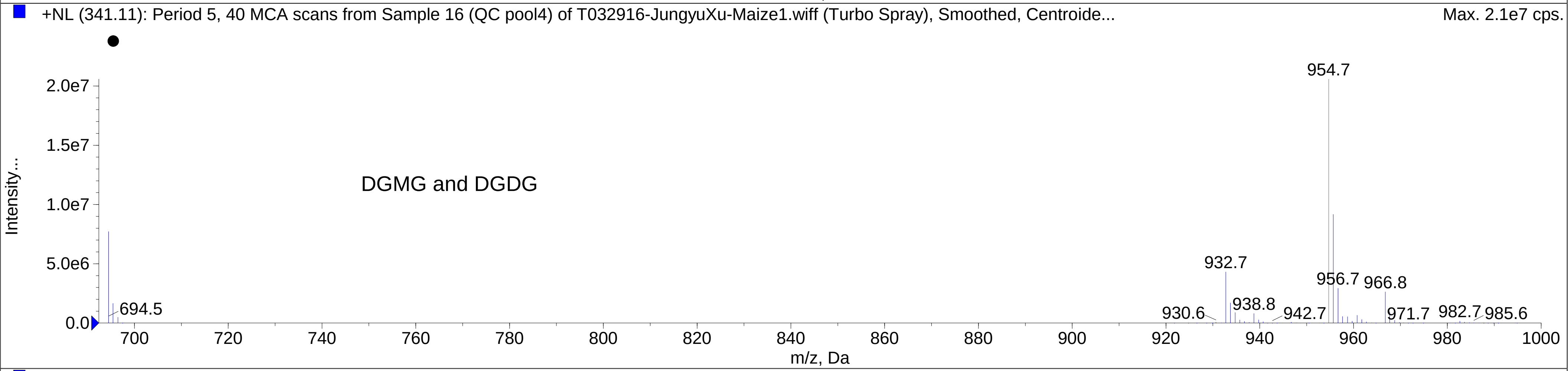
+NL (341.11): Period 5, 40 MCA scans from Sample 16 (QC pool4) of T032916-JungyuXu-Maize1.wiff (Turbo Spray), Smoothed, Centroide...
Max. 2.1e7 cps.
DGMG and DGDG
Intensity...
m/z, Da
954.7
932.7
956.7
966.8
938.8
694.5
930.6
942.7
971.7
982.7
985.6

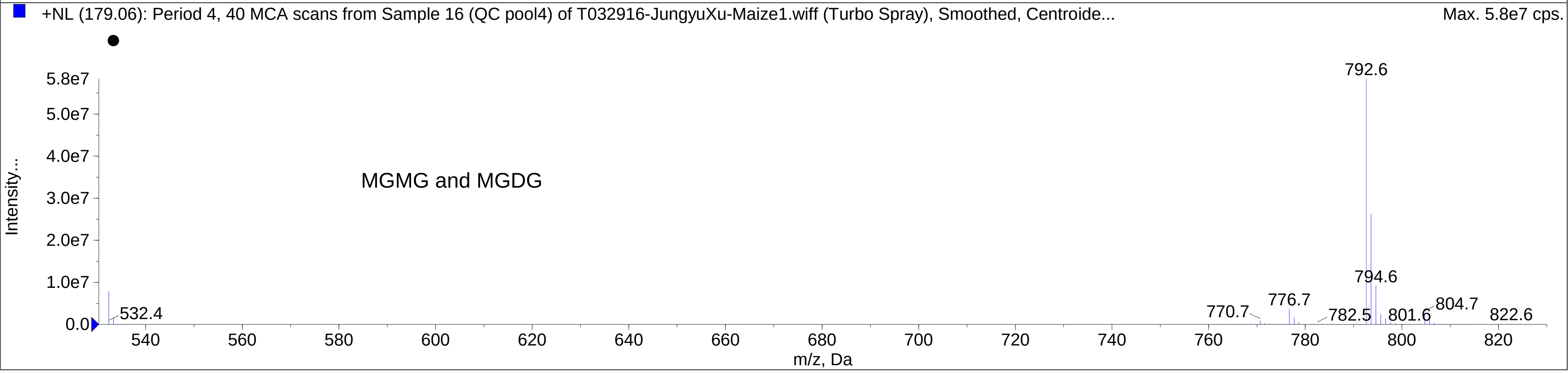
+NL (179.06): Period 4, 40 MCA scans from Sample 16 (QC pool4) of T032916-JungyuXu-Maize1.wiff (Turbo Spray), Smoothed, Centroide...
Max. 5.8e7 cps.
MGMG and MGDG
Intensity...
m/z, Da
792.6
794.6
776.7
770.7
782.5
801.6
804.7
822.6
532.4

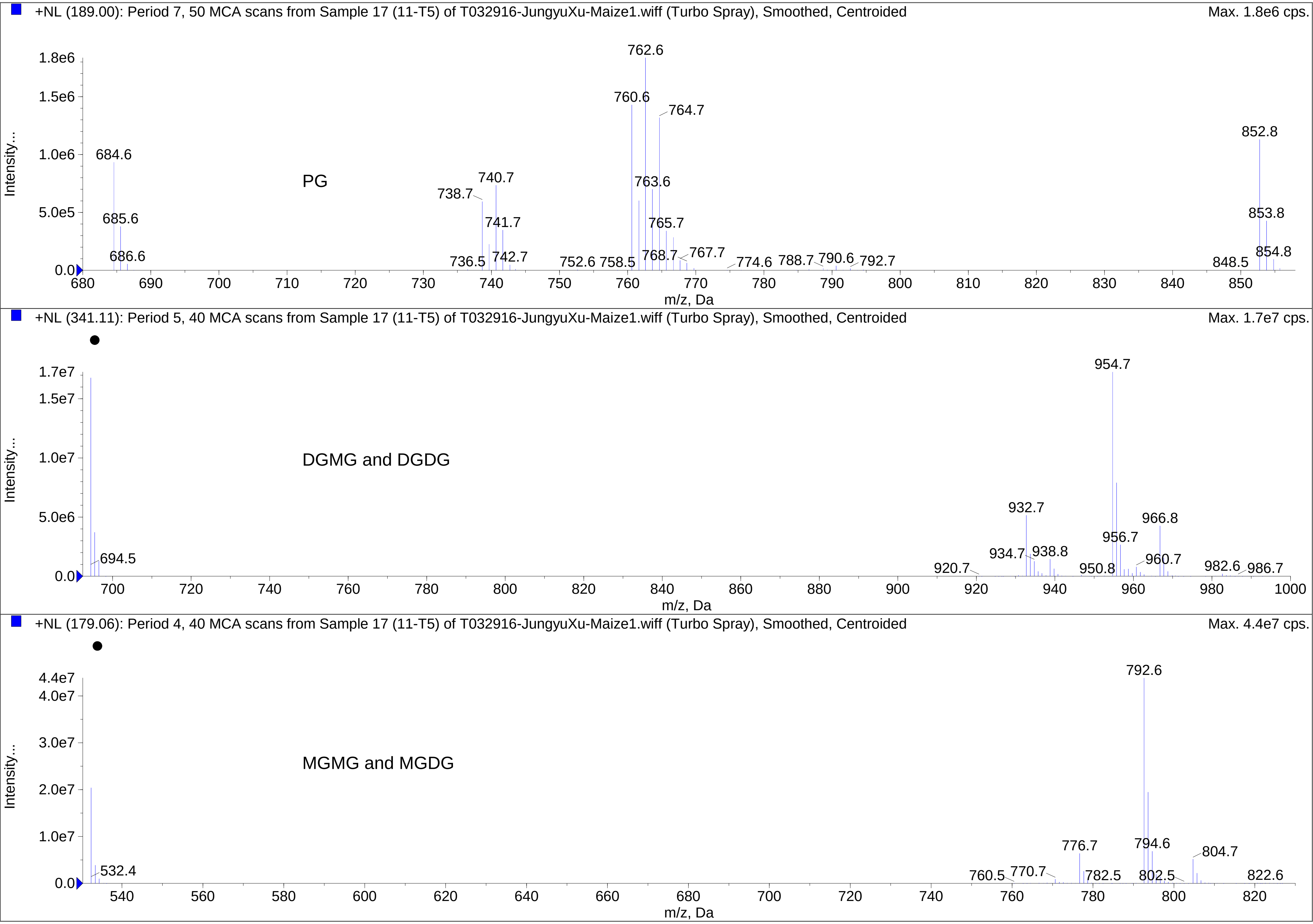

+NL (189.00): Period 7, 50 MCA scans from Sample 17 (11-T5) of T032916-JungyuXu-Maize1.wiff (Turbo Spray), Smoothed, Centroided
Max. 1.8e6 cps.
PG
762.6
760.6
764.7
852.8
684.6
740.7
738.7
763.6
685.6
741.7
765.7
853.8
686.6
742.7
736.5
752.6
758.5
768.7
767.7
774.6
788.7
790.6
792.7
848.5
854.8
Intensity...
m/z, Da
+NL (341.11): Period 5, 40 MCA scans from Sample 17 (11-T5) of T032916-JungyuXu-Maize1.wiff (Turbo Spray), Smoothed, Centroided
Max. 1.7e7 cps.
DGMG and DGDG
954.7
932.7
966.8
956.7
934.7
938.8
960.7
694.5
920.7
950.8
982.6
986.7
Intensity...
m/z, Da
+NL (179.06): Period 4, 40 MCA scans from Sample 17 (11-T5) of T032916-JungyuXu-Maize1.wiff (Turbo Spray), Smoothed, Centroided
Max. 4.4e7 cps.
MGMG and MGDG
792.6
776.7
794.6
804.7
532.4
760.5
770.7
782.5
802.5
822.6
Intensity...
m/z, Da

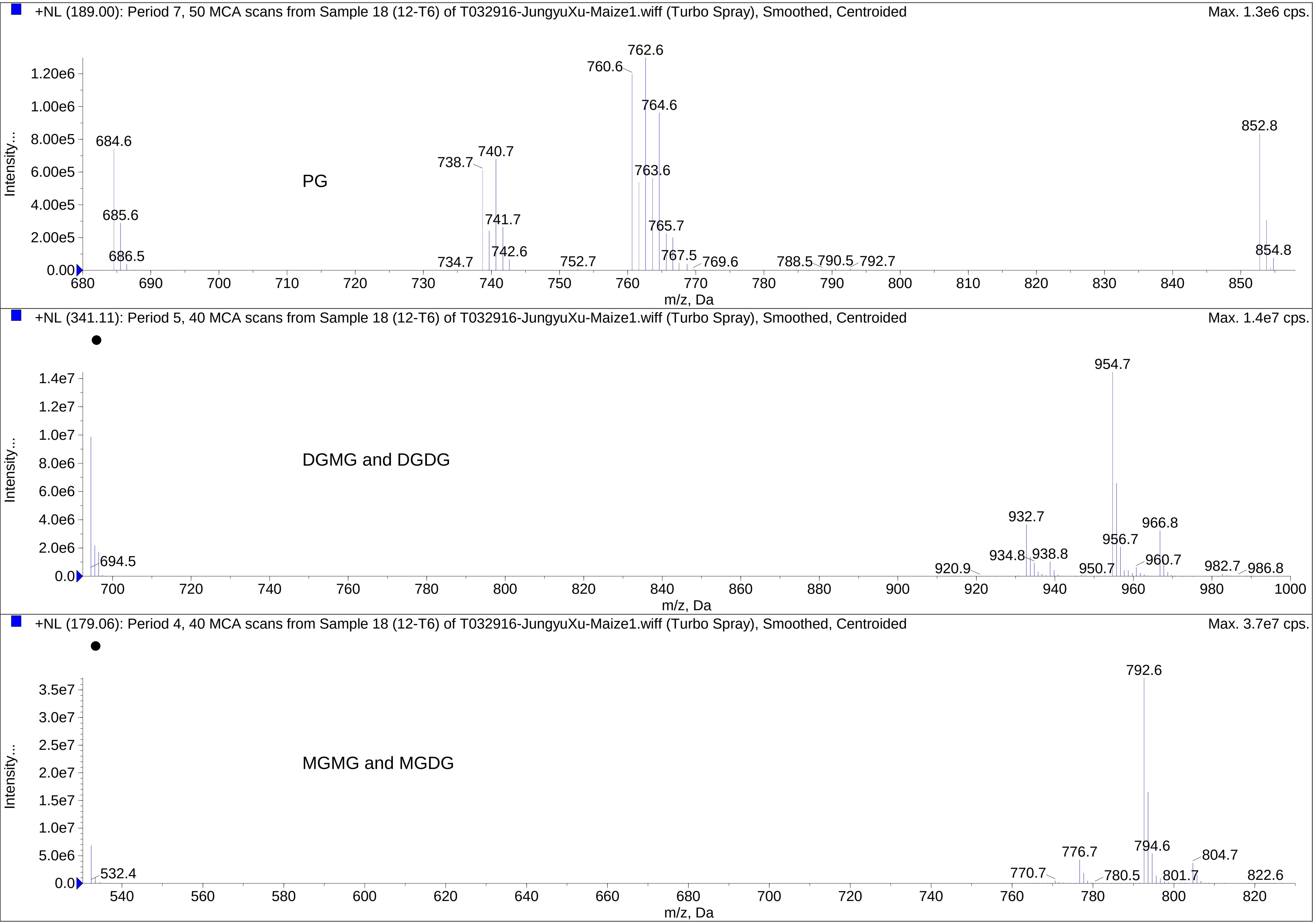
+NL (189.00): Period 7, 50 MCA scans from Sample 18 (12-T6) of T032916-JungyuXu-Maize1.wiff (Turbo Spray), Smoothed, Centroided
Max. 1.3e6 cps.
PG
762.6
760.6
764.6
852.8
684.6
740.7
738.7
763.6
685.6
741.7
765.7
686.5
742.6
854.8
767.5
734.7
752.7
769.6
788.5
790.5
792.7
Intensity...
m/z, Da
+NL (341.11): Period 5, 40 MCA scans from Sample 18 (12-T6) of T032916-JungyuXu-Maize1.wiff (Turbo Spray), Smoothed, Centroided
Max. 1.4e7 cps.
DGMG and DGDG
954.7
932.7
966.8
956.7
934.8
938.8
960.7
694.5
920.9
950.7
982.7
986.8
Intensity...
m/z, Da
+NL (179.06): Period 4, 40 MCA scans from Sample 18 (12-T6) of T032916-JungyuXu-Maize1.wiff (Turbo Spray), Smoothed, Centroided
Max. 3.7e7 cps.
MGMG and MGDG
792.6
794.6
776.7
804.7
532.4
770.7
780.5
801.7
822.6
Intensity...
m/z, Da

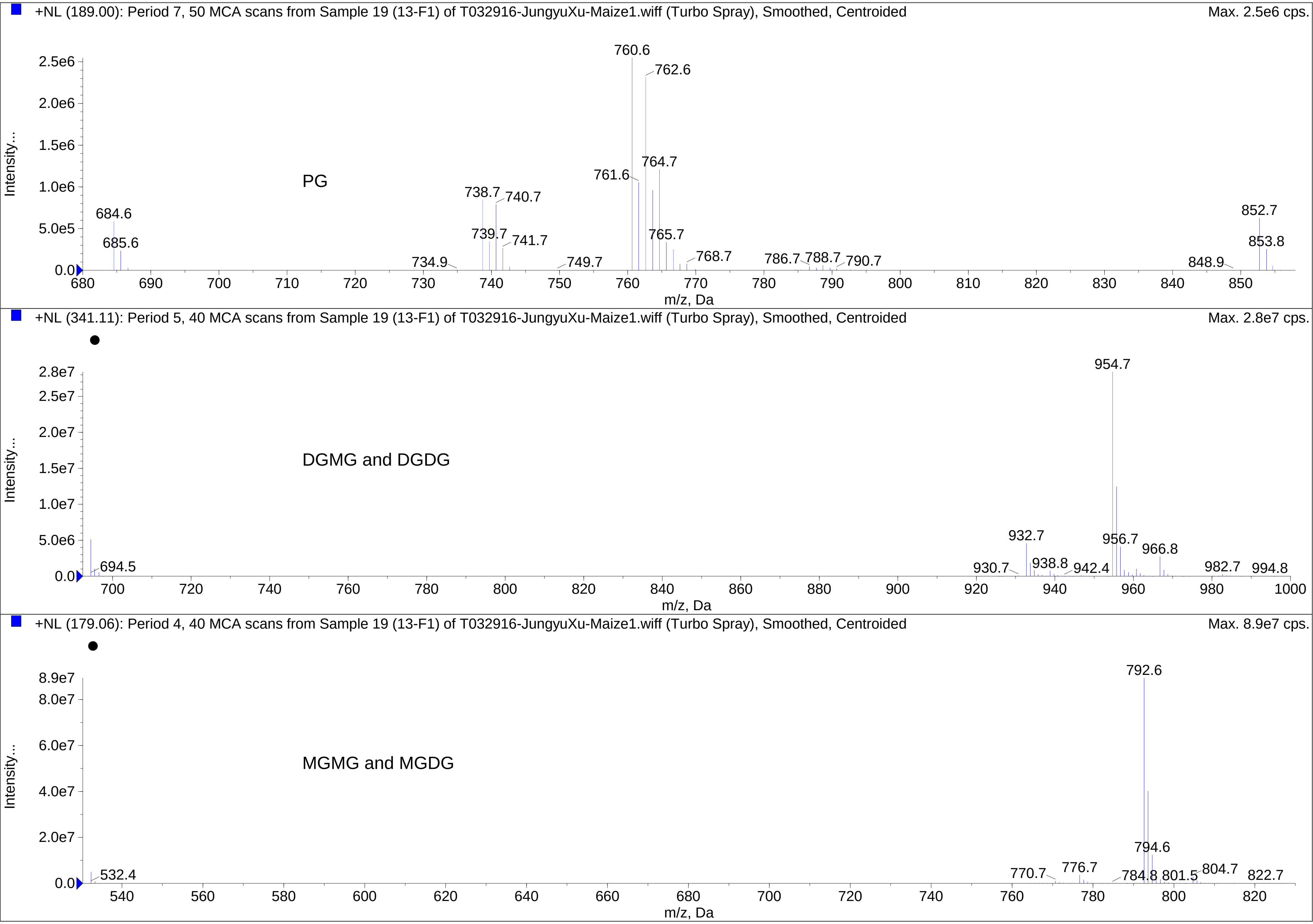
+NL (189.00): Period 7, 50 MCA scans from Sample 19 (13-F1) of T032916-JungyuXu-Maize1.wiff (Turbo Spray), Smoothed, Centroided
Max. 2.5e6 cps.
PG
760.6
762.6
764.7
761.6
738.7
740.7
684.6
852.7
739.7
741.7
765.7
685.6
853.8
768.7
734.9
749.7
786.7
788.7
790.7
848.9
Intensity...
m/z, Da
+NL (341.11): Period 5, 40 MCA scans from Sample 19 (13-F1) of T032916-JungyuXu-Maize1.wiff (Turbo Spray), Smoothed, Centroided
Max. 2.8e7 cps.
DGMG and DGDG
954.7
932.7
956.7
966.8
694.5
930.7
938.8
942.4
982.7
994.8
Intensity...
m/z, Da
+NL (179.06): Period 4, 40 MCA scans from Sample 19 (13-F1) of T032916-JungyuXu-Maize1.wiff (Turbo Spray), Smoothed, Centroided
Max. 8.9e7 cps.
MGMG and MGDG
792.6
794.6
532.4
770.7
776.7
784.8
801.5
804.7
822.7
Intensity...
m/z, Da

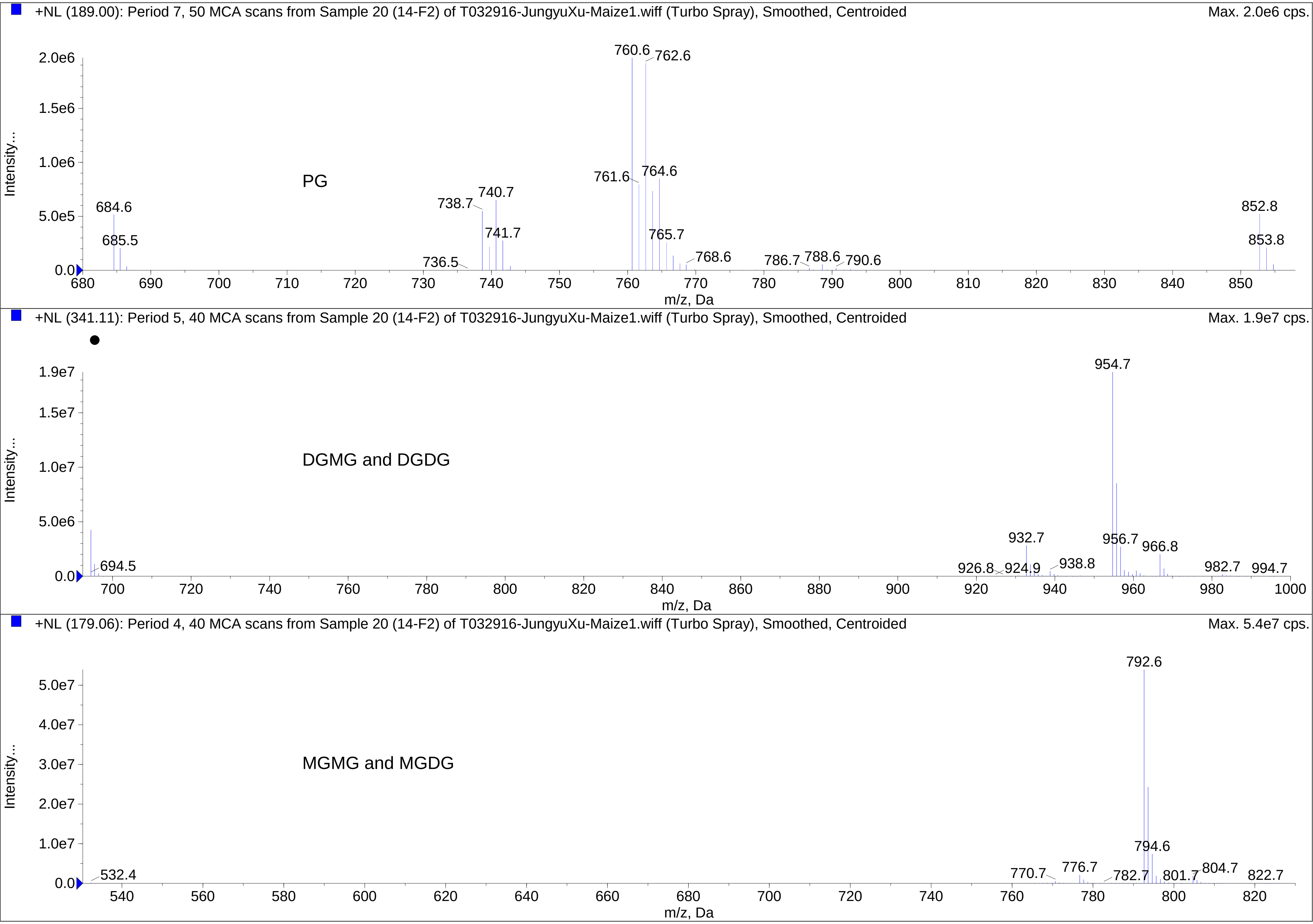

+NL (189.00): Period 7, 50 MCA scans from Sample 20 (14-F2) of T032916-JungyuXu-Maize1.wiff (Turbo Spray), Smoothed, Centroided
Max. 2.0e6 cps.
PG
Intensity...
m/z, Da
684.6
685.5
736.5
738.7
740.7
741.7
760.6
761.6
762.6
764.6
765.7
768.6
786.7
788.6
790.6
852.8
853.8
+NL (341.11): Period 5, 40 MCA scans from Sample 20 (14-F2) of T032916-JungyuXu-Maize1.wiff (Turbo Spray), Smoothed, Centroided
Max. 1.9e7 cps.
DGMG and DGDG
Intensity...
m/z, Da
694.5
926.8
924.9
932.7
938.8
954.7
956.7
966.8
982.7
994.7
+NL (179.06): Period 4, 40 MCA scans from Sample 20 (14-F2) of T032916-JungyuXu-Maize1.wiff (Turbo Spray), Smoothed, Centroided
Max. 5.4e7 cps.
MGMG and MGDG
Intensity...
m/z, Da
532.4
770.7
776.7
782.7
792.6
794.6
801.7
804.7
822.7

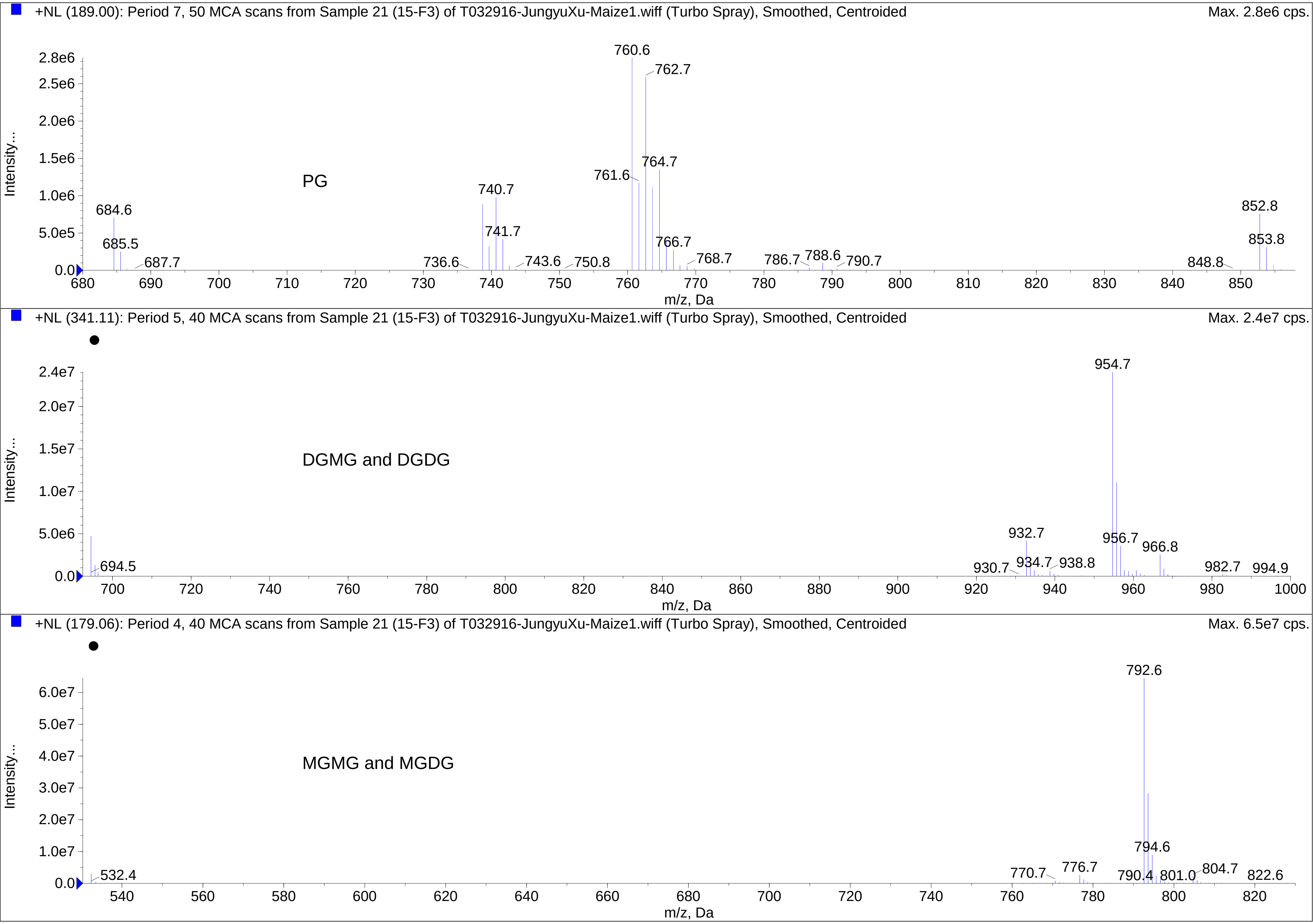

+NL (189.00): Period 7, 50 MCA scans from Sample 21 (15-F3) of T032916-JungyuXu-Maize1.wiff (Turbo Spray), Smoothed, Centroided
Max. 2.8e6 cps.
PG
760.6
762.7
764.7
761.6
740.7
741.7
684.6
685.5
687.7
736.6
743.6
750.8
766.7
768.7
786.7
788.6
790.7
848.8
852.8
853.8
Intensity...
m/z, Da
+NL (341.11): Period 5, 40 MCA scans from Sample 21 (15-F3) of T032916-JungyuXu-Maize1.wiff (Turbo Spray), Smoothed, Centroided
Max. 2.4e7 cps.
DGMG and DGDG
954.7
932.7
956.7
966.8
694.5
930.7
934.7
938.8
982.7
994.9
Intensity...
m/z, Da
+NL (179.06): Period 4, 40 MCA scans from Sample 21 (15-F3) of T032916-JungyuXu-Maize1.wiff (Turbo Spray), Smoothed, Centroided
Max. 6.5e7 cps.
MGMG and MGDG
792.6
794.6
532.4
770.7
776.7
790.4
801.0
804.7
822.6
Intensity...
m/z, Da

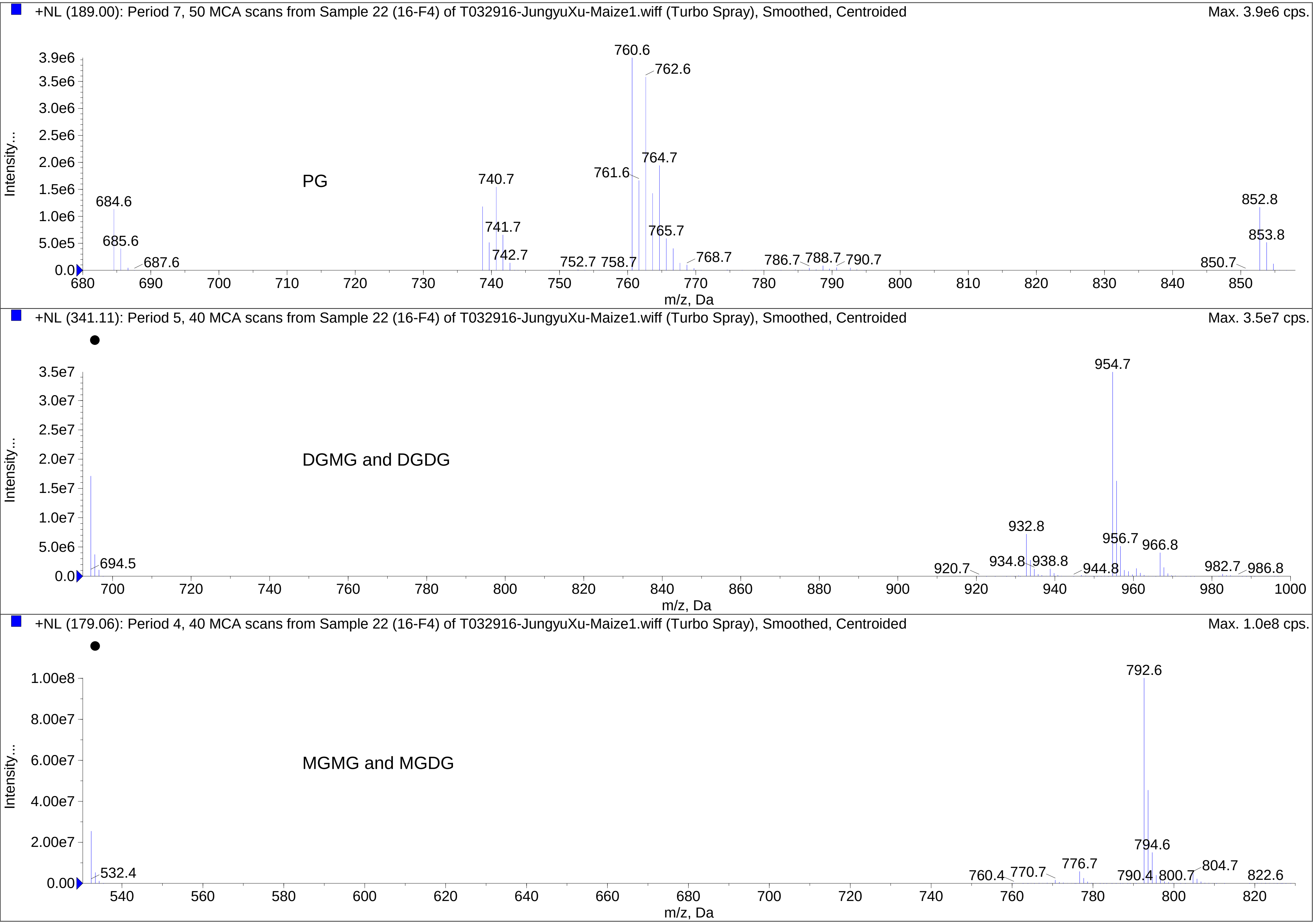

+NL (189.00): Period 7, 50 MCA scans from Sample 22 (16-F4) of T032916-JungyuXu-Maize1.wiff (Turbo Spray), Smoothed, Centroided
Max. 3.9e6 cps.
PG
760.6
762.6
764.7
761.6
740.7
684.6
741.7
765.7
685.6
742.7
687.6
752.7
758.7
768.7
786.7
788.7
790.7
852.8
853.8
850.7
Intensity...
m/z, Da
+NL (341.11): Period 5, 40 MCA scans from Sample 22 (16-F4) of T032916-JungyuXu-Maize1.wiff (Turbo Spray), Smoothed, Centroided
Max. 3.5e7 cps.
DGMG and DGDG
954.7
932.8
956.7
966.8
694.5
920.7
934.8
938.8
944.8
982.7
986.8
Intensity...
m/z, Da
+NL (179.06): Period 4, 40 MCA scans from Sample 22 (16-F4) of T032916-JungyuXu-Maize1.wiff (Turbo Spray), Smoothed, Centroided
Max. 1.0e8 cps.
MGMG and MGDG
792.6
794.6
532.4
760.4
770.7
776.7
790.4
800.7
804.7
822.6
Intensity...
m/z, Da

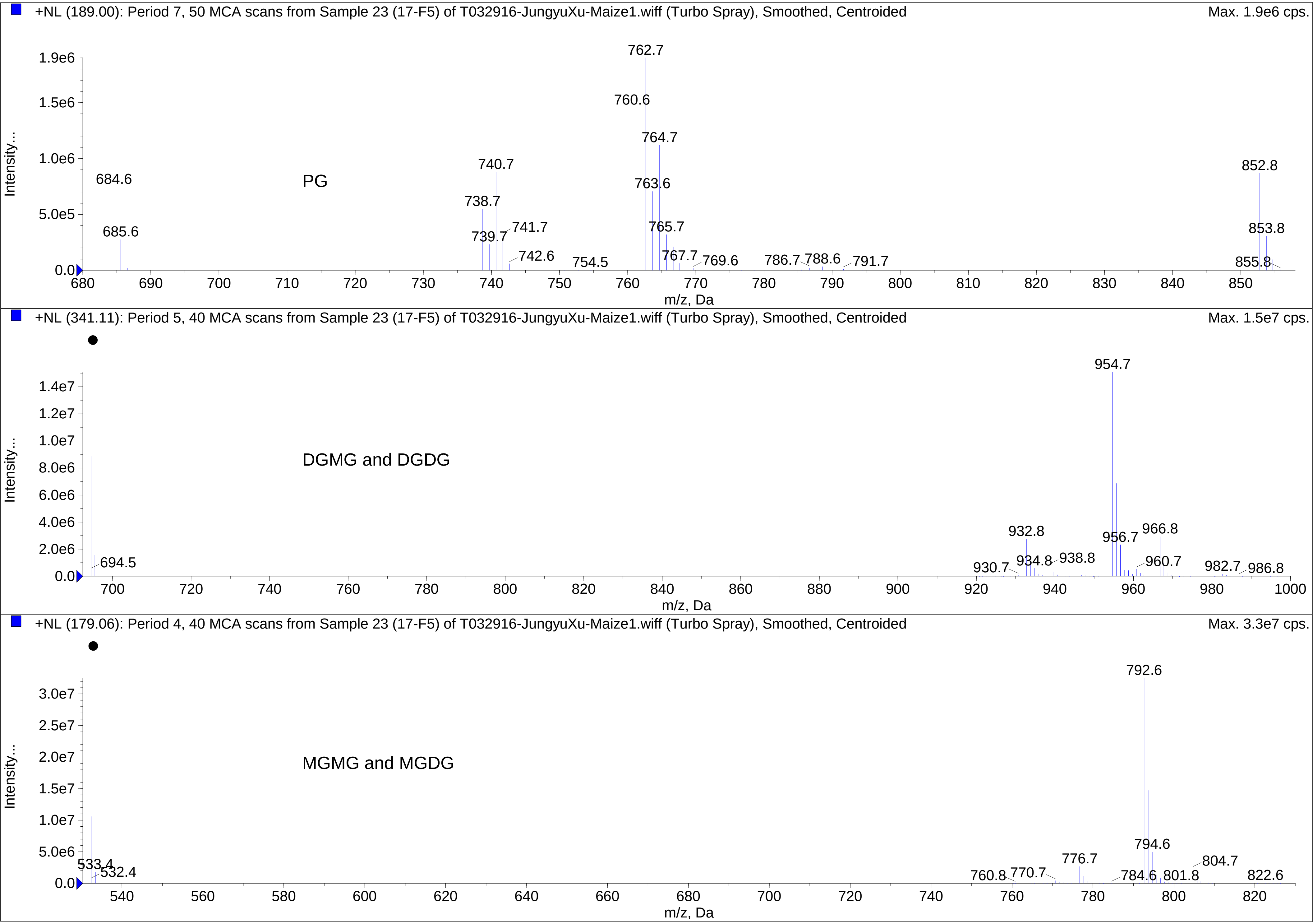

+NL (189.00): Period 7, 50 MCA scans from Sample 23 (17-F5) of T032916-JungyuXu-Maize1.wiff (Turbo Spray), Smoothed, Centroided
Max. 1.9e6 cps.
PG
Intensity...
m/z, Da
762.7
760.6
764.7
740.7
852.8
684.6
763.6
738.7
741.7
685.6
739.7
765.7
853.8
742.6
767.7
754.5
769.6
786.7
788.6
791.7
855.8
+NL (341.11): Period 5, 40 MCA scans from Sample 23 (17-F5) of T032916-JungyuXu-Maize1.wiff (Turbo Spray), Smoothed, Centroided
Max. 1.5e7 cps.
DGMG and DGDG
Intensity...
m/z, Da
954.7
932.8
956.7
966.8
694.5
934.8
938.8
960.7
930.7
982.7
986.8
+NL (179.06): Period 4, 40 MCA scans from Sample 23 (17-F5) of T032916-JungyuXu-Maize1.wiff (Turbo Spray), Smoothed, Centroided
Max. 3.3e7 cps.
MGMG and MGDG
Intensity...
m/z, Da
792.6
794.6
776.7
804.7
533.4
532.4
760.8
770.7
784.6
801.8
822.6

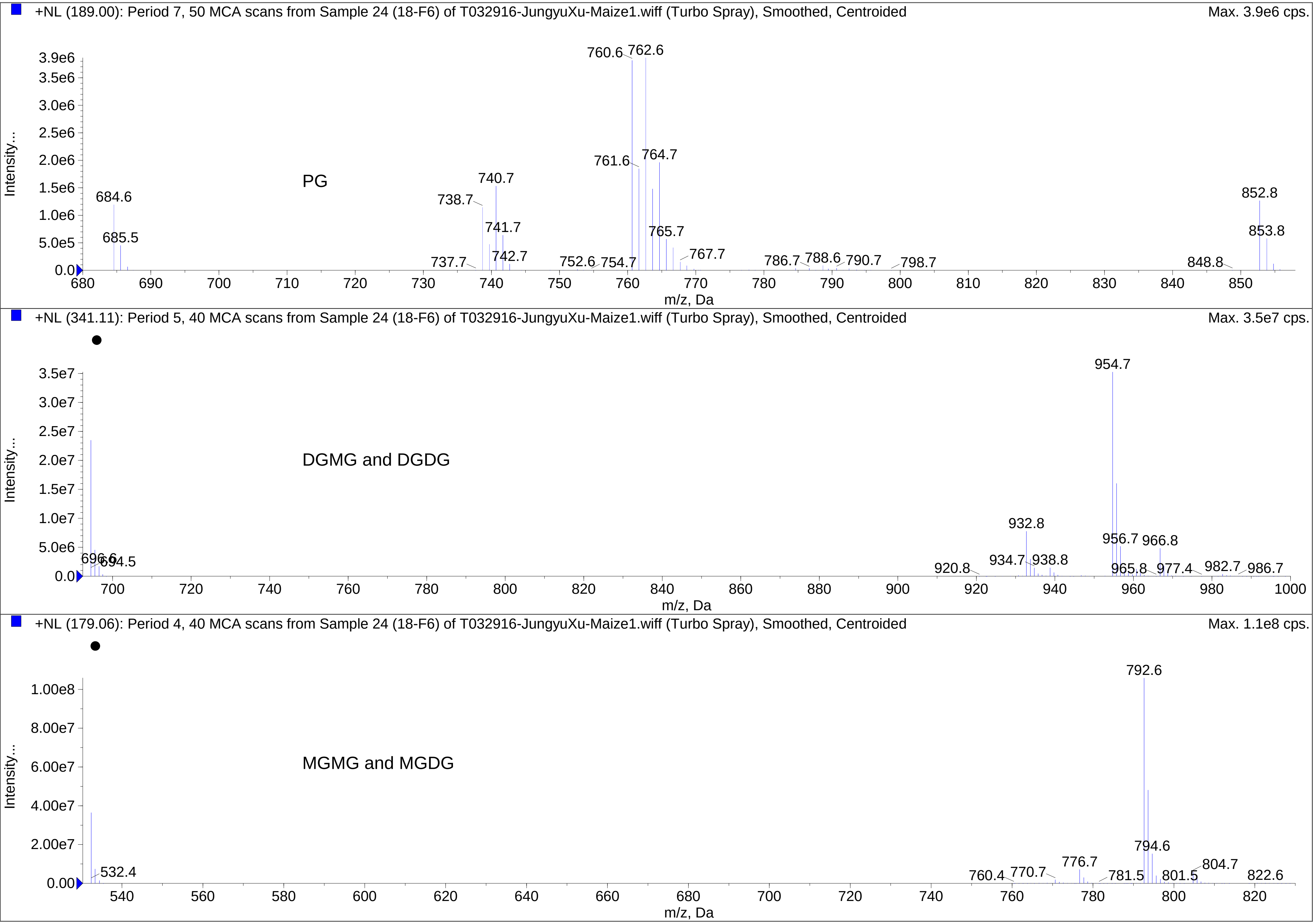
+NL (189.00): Period 7, 50 MCA scans from Sample 24 (18-F6) of T032916-JungyuXu-Maize1.wiff (Turbo Spray), Smoothed, Centroided
Max. 3.9e6 cps.
PG
Intensity...
m/z, Da
+NL (341.11): Period 5, 40 MCA scans from Sample 24 (18-F6) of T032916-JungyuXu-Maize1.wiff (Turbo Spray), Smoothed, Centroided
Max. 3.5e7 cps.
DGMG and DGDG
+NL (179.06): Period 4, 40 MCA scans from Sample 24 (18-F6) of T032916-JungyuXu-Maize1.wiff (Turbo Spray), Smoothed, Centroided
Max. 1.1e8 cps.
MGMG and MGDG

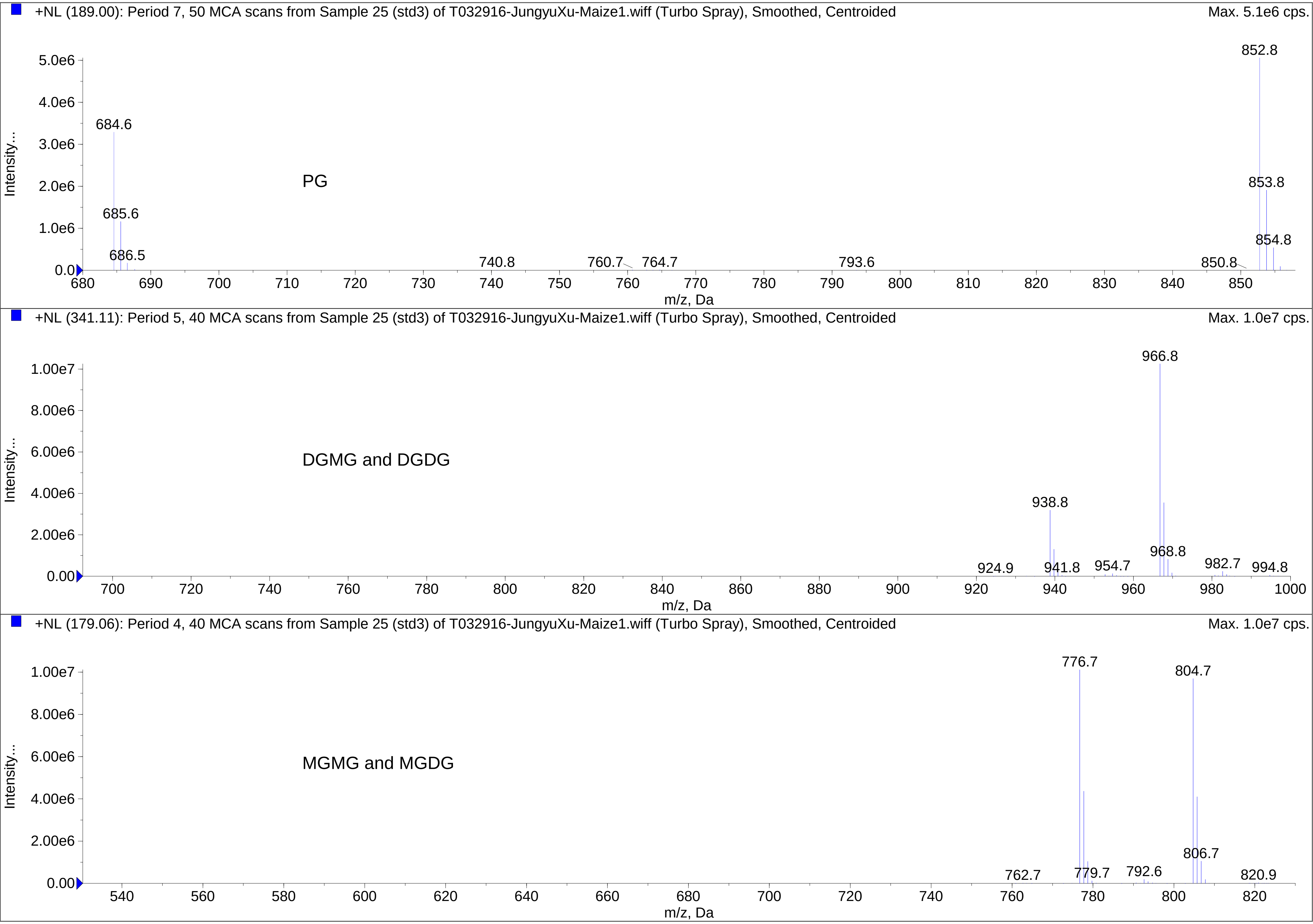

+NL (189.00): Period 7, 50 MCA scans from Sample 25 (std3) of T032916-JungyuXu-Maize1.wiff (Turbo Spray), Smoothed, Centroided
Max. 5.1e6 cps.
PG
684.6
685.6
686.5
740.8
760.7
764.7
793.6
850.8
852.8
853.8
854.8
Intensity...
m/z, Da
+NL (341.11): Period 5, 40 MCA scans from Sample 25 (std3) of T032916-JungyuXu-Maize1.wiff (Turbo Spray), Smoothed, Centroided
Max. 1.0e7 cps.
DGMG and DGDG
924.9
938.8
941.8
954.7
966.8
968.8
982.7
994.8
Intensity...
m/z, Da
+NL (179.06): Period 4, 40 MCA scans from Sample 25 (std3) of T032916-JungyuXu-Maize1.wiff (Turbo Spray), Smoothed, Centroided
Max. 1.0e7 cps.
MGMG and MGDG
762.7
776.7
779.7
792.6
804.7
806.7
820.9
Intensity...
m/z, Da

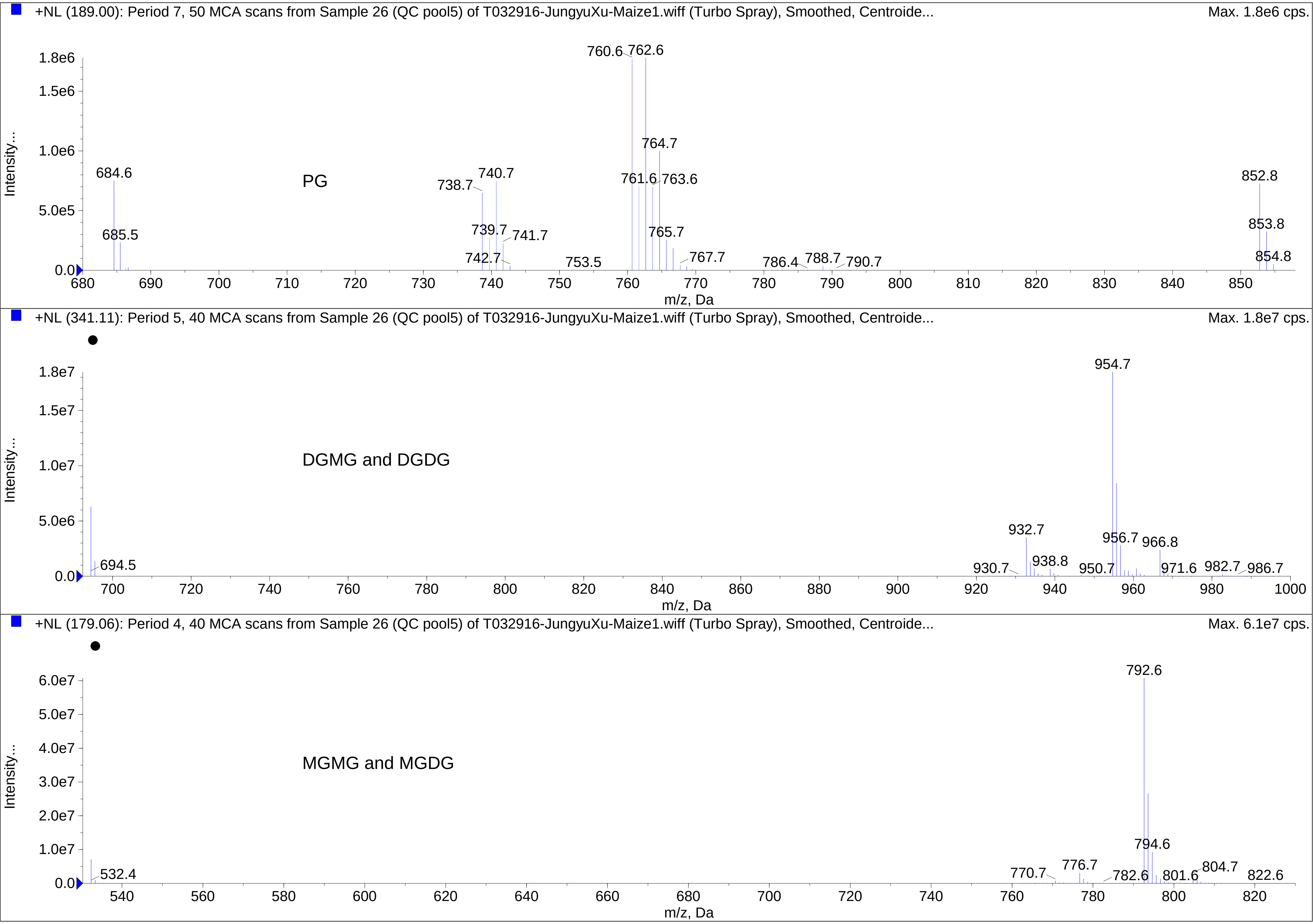
+NL (189.00): Period 7, 50 MCA scans from Sample 26 (QC pool5) of T032916-JungyuXu-Maize1.wiff (Turbo Spray), Smoothed, Centroide...
Max. 1.8e6 cps.
PG
684.6
685.5
738.7
739.7
740.7
741.7
742.7
753.5
760.6
762.6
761.6
763.6
764.7
765.7
767.7
786.4
788.7
790.7
852.8
853.8
854.8
Intensity...
m/z, Da
+NL (341.11): Period 5, 40 MCA scans from Sample 26 (QC pool5) of T032916-JungyuXu-Maize1.wiff (Turbo Spray), Smoothed, Centroide...
Max. 1.8e7 cps.
DGMG and DGDG
694.5
930.7
932.7
938.8
950.7
954.7
956.7
966.8
971.6
982.7
986.7
Intensity...
m/z, Da
+NL (179.06): Period 4, 40 MCA scans from Sample 26 (QC pool5) of T032916-JungyuXu-Maize1.wiff (Turbo Spray), Smoothed, Centroide...
Max. 6.1e7 cps.
MGMG and MGDG
532.4
770.7
776.7
782.6
792.6
794.6
801.6
804.7
822.6
Intensity...
m/z, Da

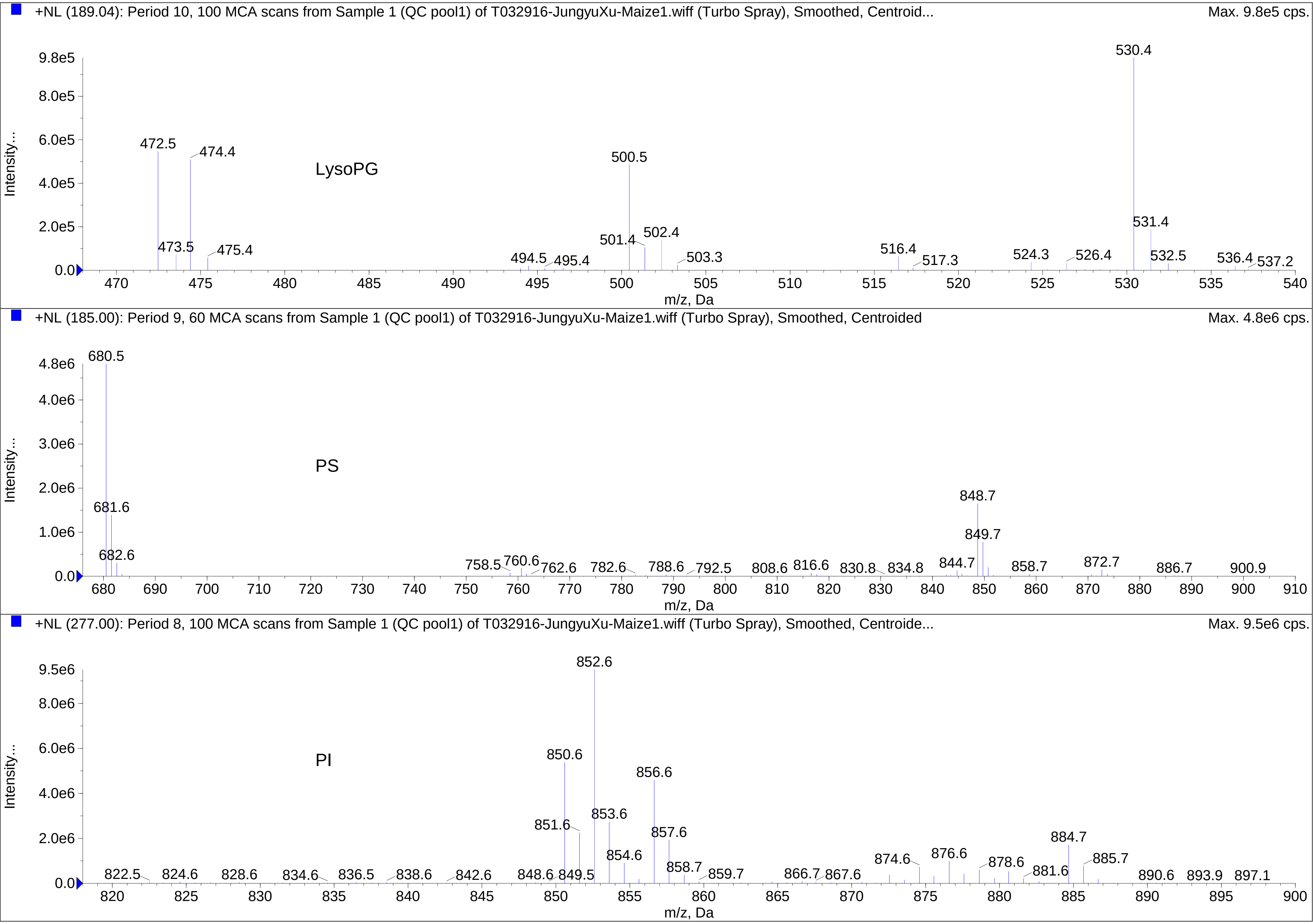

+NL (189.04): Period 10, 100 MCA scans from Sample 1 (QC pool1) of T032916-JungyuXu-Maize1.wiff (Turbo Spray), Smoothed, Centroid...
Max. 9.8e5 cps.
LysoPG
472.5
473.5
474.4
475.4
494.5
495.4
500.5
501.4
502.4
503.3
516.4
517.3
524.3
526.4
530.4
531.4
532.5
536.4
537.2
Intensity...
m/z, Da
+NL (185.00): Period 9, 60 MCA scans from Sample 1 (QC pool1) of T032916-JungyuXu-Maize1.wiff (Turbo Spray), Smoothed, Centroided
Max. 4.8e6 cps.
PS
680.5
681.6
682.6
758.5
760.6
762.6
782.6
788.6
792.5
808.6
816.6
830.8
834.8
844.7
848.7
849.7
858.7
872.7
886.7
900.9
Intensity...
m/z, Da
+NL (277.00): Period 8, 100 MCA scans from Sample 1 (QC pool1) of T032916-JungyuXu-Maize1.wiff (Turbo Spray), Smoothed, Centroide...
Max. 9.5e6 cps.
PI
822.5
824.6
828.6
834.6
836.5
838.6
842.6
848.6
849.5
850.6
851.6
852.6
853.6
854.6
856.6
857.6
858.7
859.7
866.7
867.6
874.6
876.6
878.6
881.6
884.7
885.7
890.6
893.9
897.1
Intensity...
m/z, Da

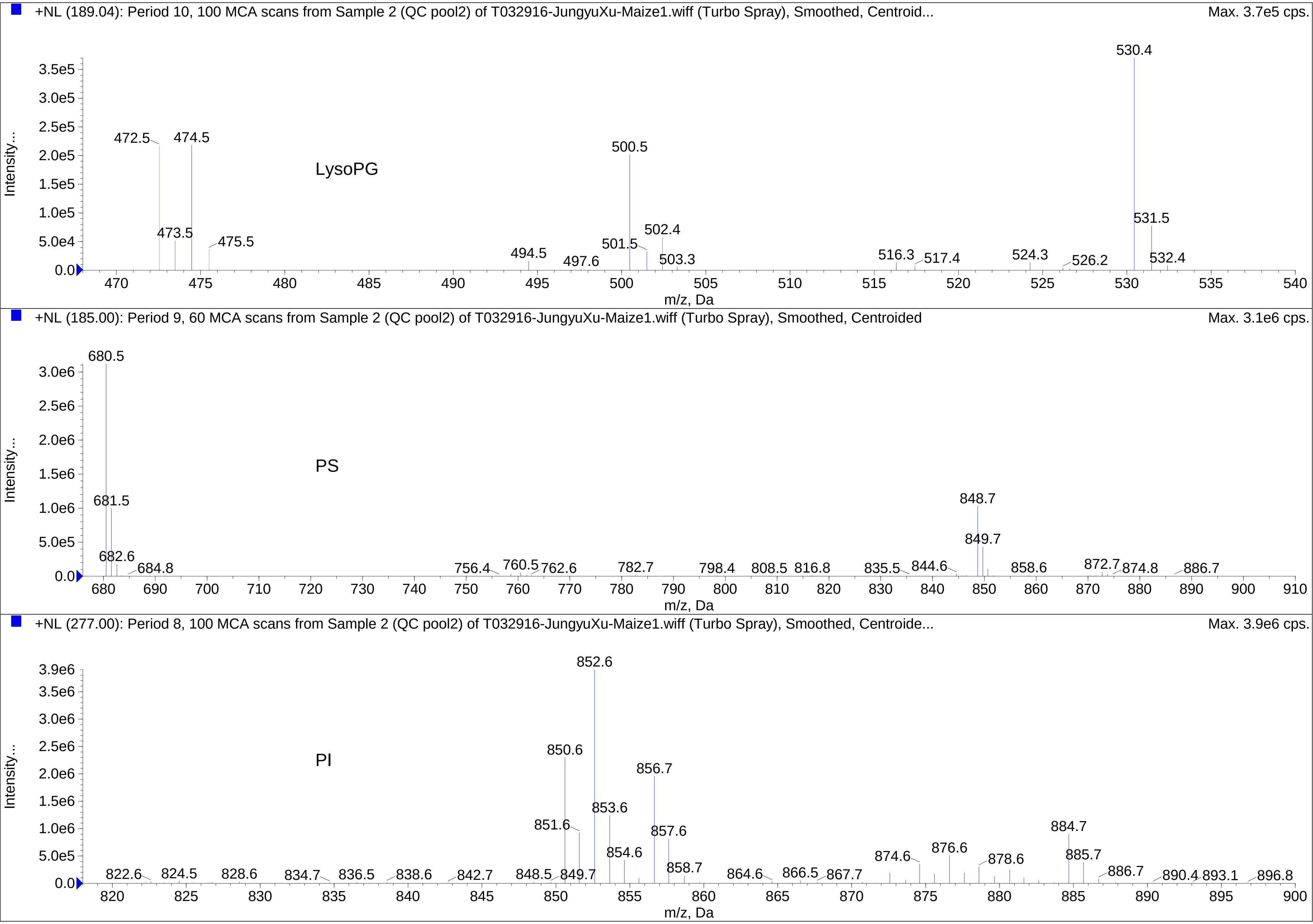
+NL (189.04): Period 10, 100 MCA scans from Sample 2 (QC pool2) of T032916-JungyuXu-Maize1.wiff (Turbo Spray), Smoothed, Centroid...
Max. 3.7e5 cps.
LysoPG
530.4
472.5
474.5
500.5
473.5
475.5
494.5
497.6
501.5
502.4
503.3
516.3
517.4
524.3
526.2
531.5
532.4
Intensity...
m/z, Da
+NL (185.00): Period 9, 60 MCA scans from Sample 2 (QC pool2) of T032916-JungyuXu-Maize1.wiff (Turbo Spray), Smoothed, Centroided
Max. 3.1e6 cps.
PS
680.5
681.5
682.6
684.8
756.4
760.5
762.6
782.7
798.4
808.5
816.8
835.5
844.6
848.7
849.7
858.6
872.7
874.8
886.7
Intensity...
m/z, Da
+NL (277.00): Period 8, 100 MCA scans from Sample 2 (QC pool2) of T032916-JungyuXu-Maize1.wiff (Turbo Spray), Smoothed, Centroide...
Max. 3.9e6 cps.
PI
852.6
850.6
856.7
853.6
851.6
857.6
854.6
858.7
822.6
824.5
828.6
834.7
836.5
838.6
842.7
848.5
849.7
864.6
866.5
867.7
874.6
876.6
878.6
884.7
885.7
886.7
890.4
893.1
896.8
Intensity...
m/z, Da

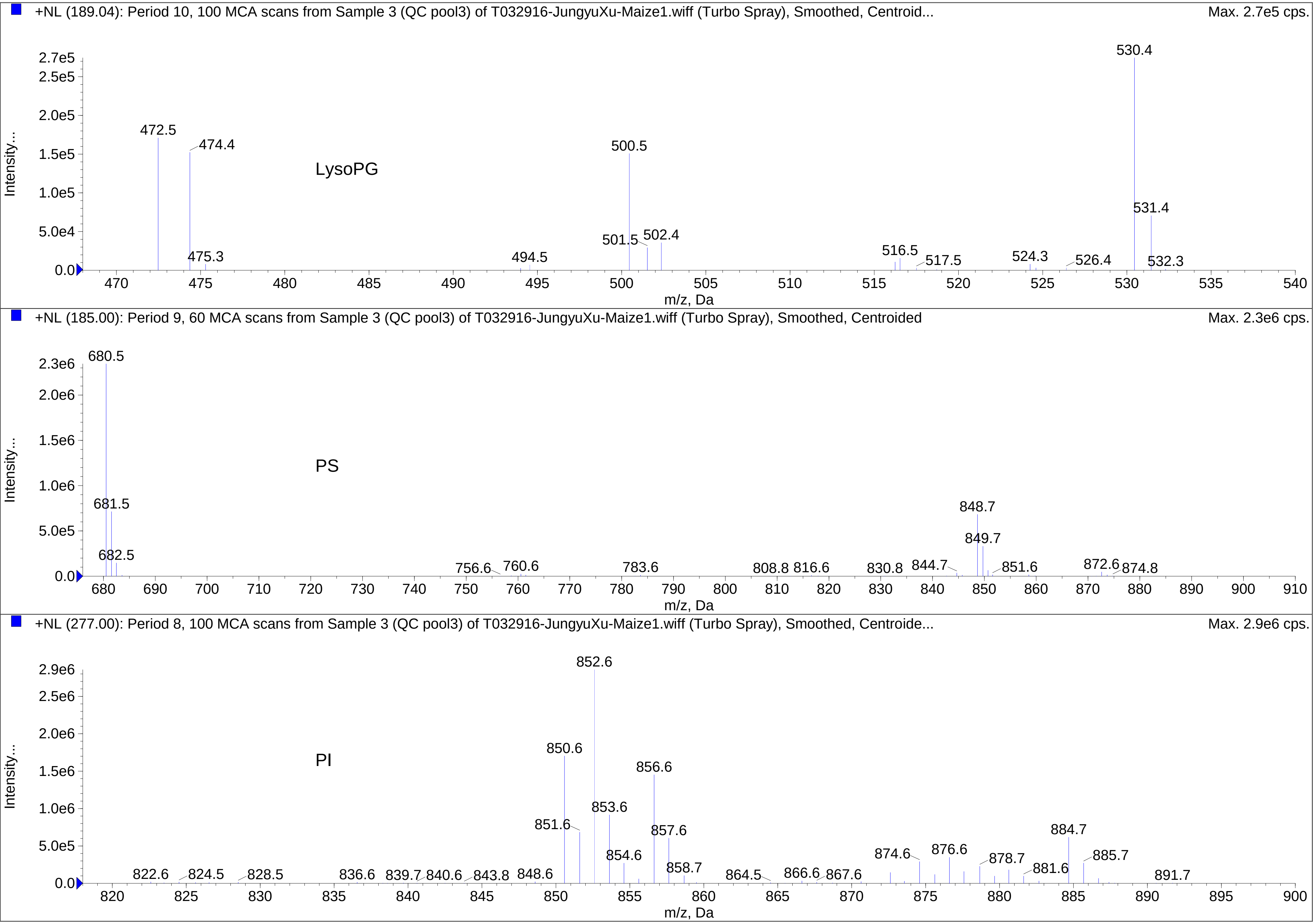

+NL (189.04): Period 10, 100 MCA scans from Sample 3 (QC pool3) of T032916-JungyuXu-Maize1.wiff (Turbo Spray), Smoothed, Centroid...
Max. 2.7e5 cps.
LysoPG
472.5
474.4
475.3
494.5
500.5
501.5
502.4
516.5
517.5
524.3
526.4
530.4
531.4
532.3
Intensity...
m/z, Da
+NL (185.00): Period 9, 60 MCA scans from Sample 3 (QC pool3) of T032916-JungyuXu-Maize1.wiff (Turbo Spray), Smoothed, Centroided
Max. 2.3e6 cps.
PS
680.5
681.5
682.5
756.6
760.6
783.6
808.8
816.6
830.8
844.7
848.7
849.7
851.6
872.6
874.8
Intensity...
m/z, Da
+NL (277.00): Period 8, 100 MCA scans from Sample 3 (QC pool3) of T032916-JungyuXu-Maize1.wiff (Turbo Spray), Smoothed, Centroide...
Max. 2.9e6 cps.
PI
822.6
824.5
828.5
836.6
839.7
840.6
843.8
848.6
850.6
851.6
852.6
853.6
854.6
856.6
857.6
858.7
864.5
866.6
867.6
874.6
876.6
878.7
881.6
884.7
885.7
891.7
Intensity...
m/z, Da

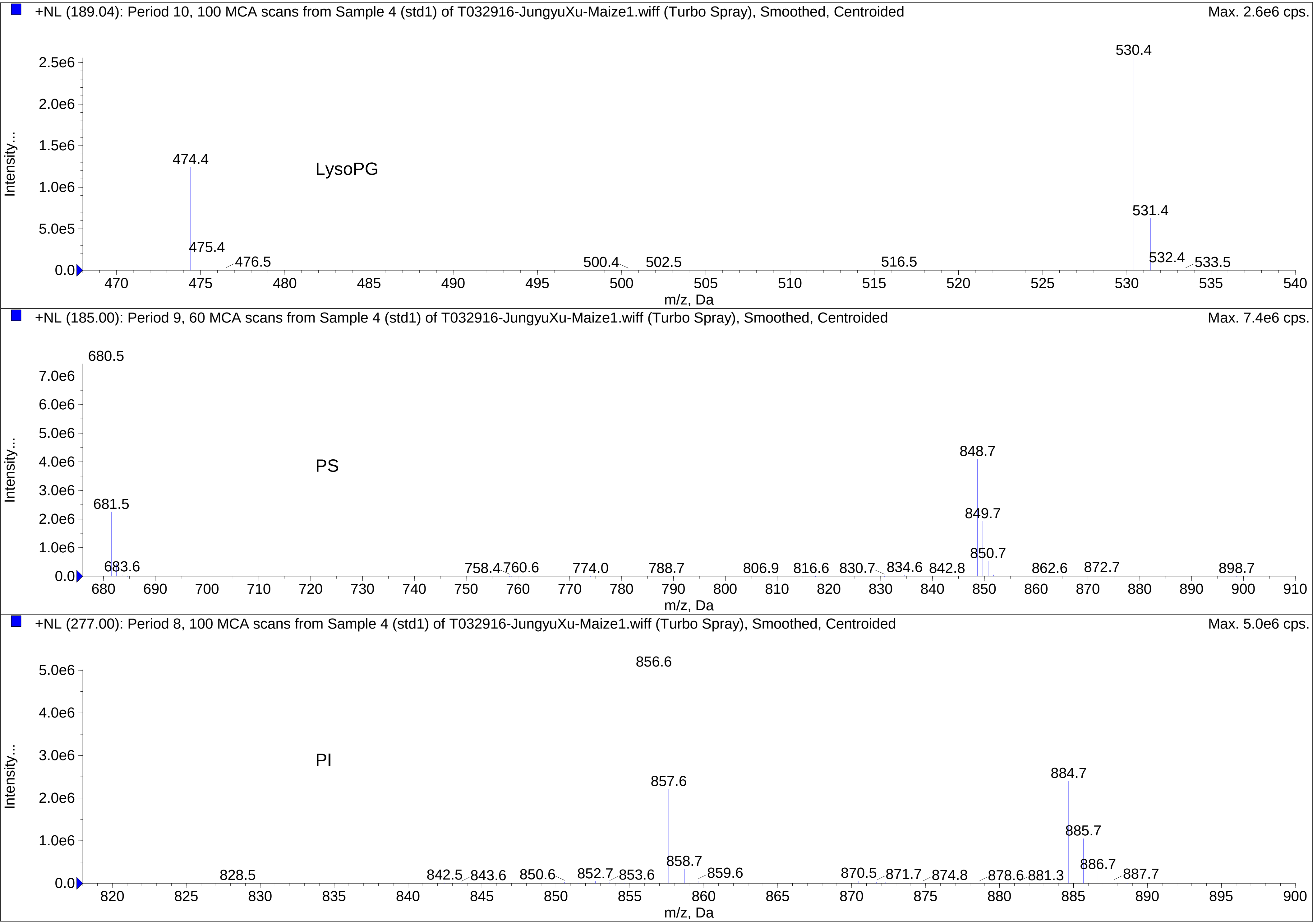

+NL (189.04): Period 10, 100 MCA scans from Sample 4 (std1) of T032916-JungyuXu-Maize1.wiff (Turbo Spray), Smoothed, Centroided
Max. 2.6e6 cps.
LysoPG
474.4
475.4
476.5
500.4
502.5
516.5
530.4
531.4
532.4
533.5
Intensity...
m/z, Da
+NL (185.00): Period 9, 60 MCA scans from Sample 4 (std1) of T032916-JungyuXu-Maize1.wiff (Turbo Spray), Smoothed, Centroided
Max. 7.4e6 cps.
PS
680.5
681.5
683.6
758.4
760.6
774.0
788.7
806.9
816.6
830.7
834.6
842.8
848.7
849.7
850.7
862.6
872.7
898.7
Intensity...
m/z, Da
+NL (277.00): Period 8, 100 MCA scans from Sample 4 (std1) of T032916-JungyuXu-Maize1.wiff (Turbo Spray), Smoothed, Centroided
Max. 5.0e6 cps.
PI
828.5
842.5
843.6
850.6
852.7
853.6
856.6
857.6
858.7
859.6
870.5
871.7
874.8
878.6
881.3
884.7
885.7
886.7
887.7
Intensity...
m/z, Da

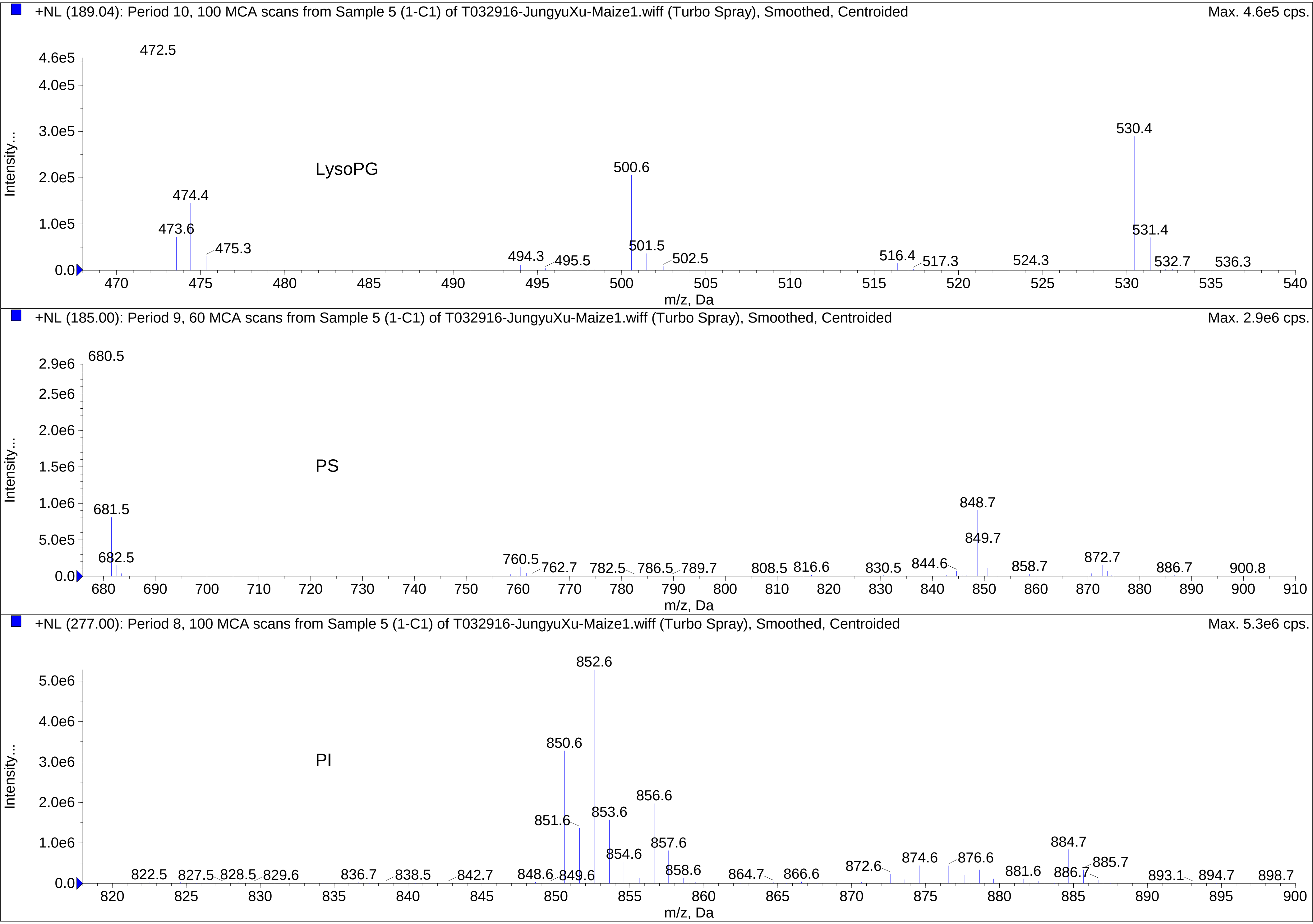

+NL (189.04): Period 10, 100 MCA scans from Sample 5 (1-C1) of T032916-JungyuXu-Maize1.wiff (Turbo Spray), Smoothed, Centroided
Max. 4.6e5 cps.
LysoPG
Intensity...
m/z, Da
472.5
473.6
474.4
475.3
494.3
495.5
500.6
501.5
502.5
516.4
517.3
524.3
530.4
531.4
532.7
536.3
+NL (185.00): Period 9, 60 MCA scans from Sample 5 (1-C1) of T032916-JungyuXu-Maize1.wiff (Turbo Spray), Smoothed, Centroided
Max. 2.9e6 cps.
PS
Intensity...
m/z, Da
680.5
681.5
682.5
760.5
762.7
782.5
786.5
789.7
808.5
816.6
830.5
844.6
848.7
849.7
858.7
872.7
886.7
900.8
+NL (277.00): Period 8, 100 MCA scans from Sample 5 (1-C1) of T032916-JungyuXu-Maize1.wiff (Turbo Spray), Smoothed, Centroided
Max. 5.3e6 cps.
PI
Intensity...
m/z, Da
852.6
850.6
851.6
853.6
856.6
857.6
854.6
858.6
822.5
827.5
828.5
829.6
836.7
838.5
842.7
848.6
849.6
864.7
866.6
872.6
874.6
876.6
881.6
884.7
885.7
886.7
893.1
894.7
898.7

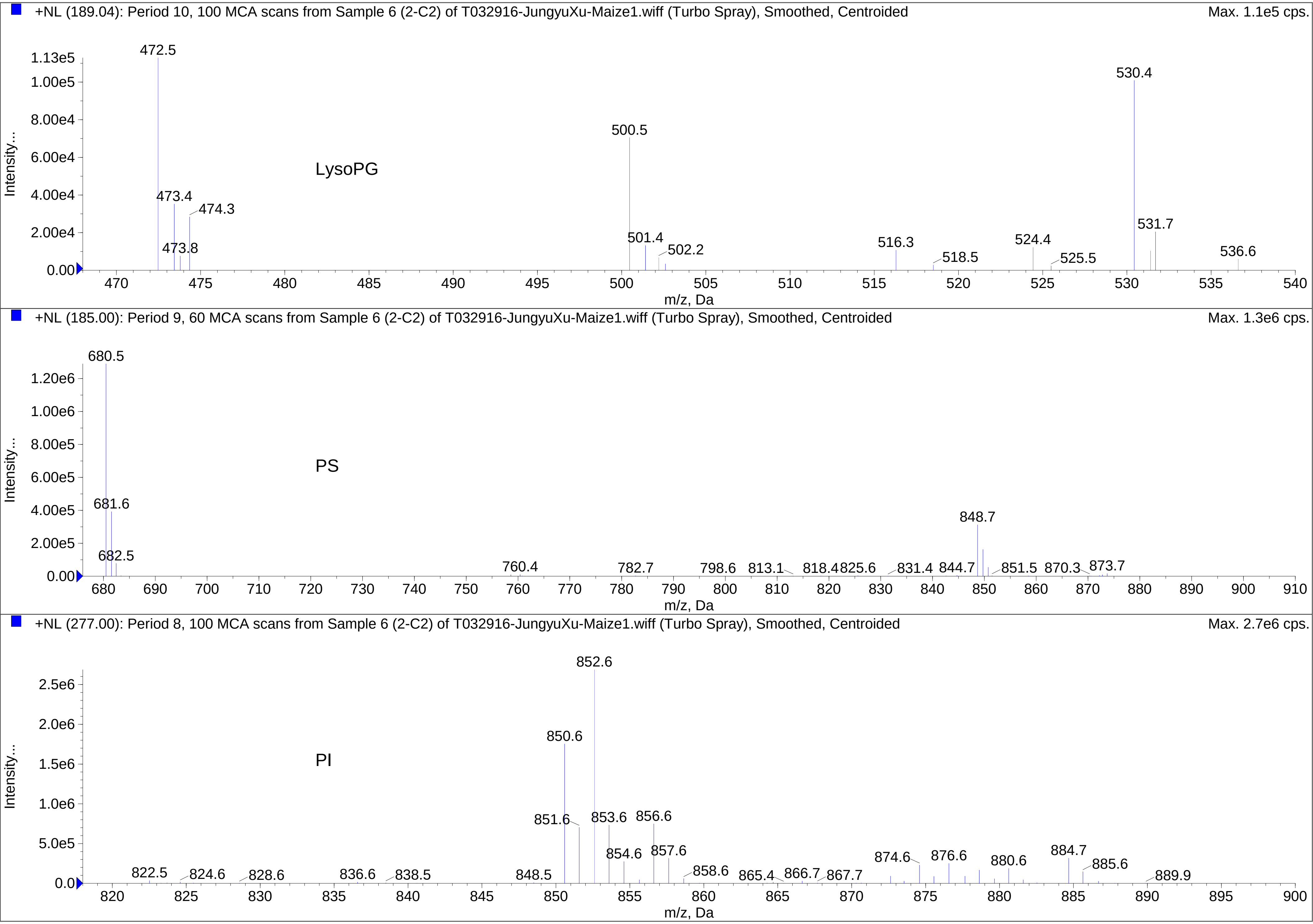
+NL (189.04): Period 10, 100 MCA scans from Sample 6 (2-C2) of T032916-JungyuXu-Maize1.wiff (Turbo Spray), Smoothed, Centroided
Max. 1.1e5 cps.
LysoPG
Intensity...
m/z, Da
472.5
473.4
474.3
473.8
500.5
501.4
502.2
516.3
518.5
524.4
525.5
530.4
531.7
536.6
+NL (185.00): Period 9, 60 MCA scans from Sample 6 (2-C2) of T032916-JungyuXu-Maize1.wiff (Turbo Spray), Smoothed, Centroided
Max. 1.3e6 cps.
PS
Intensity...
m/z, Da
680.5
681.6
682.5
760.4
782.7
798.6
813.1
818.4
825.6
831.4
844.7
848.7
851.5
870.3
873.7
+NL (277.00): Period 8, 100 MCA scans from Sample 6 (2-C2) of T032916-JungyuXu-Maize1.wiff (Turbo Spray), Smoothed, Centroided
Max. 2.7e6 cps.
PI
Intensity...
m/z, Da
852.6
850.6
851.6
853.6
856.6
854.6
857.6
858.6
822.5
824.6
828.6
836.6
838.5
848.5
865.4
866.7
867.7
874.6
876.6
880.6
884.7
885.6
889.9

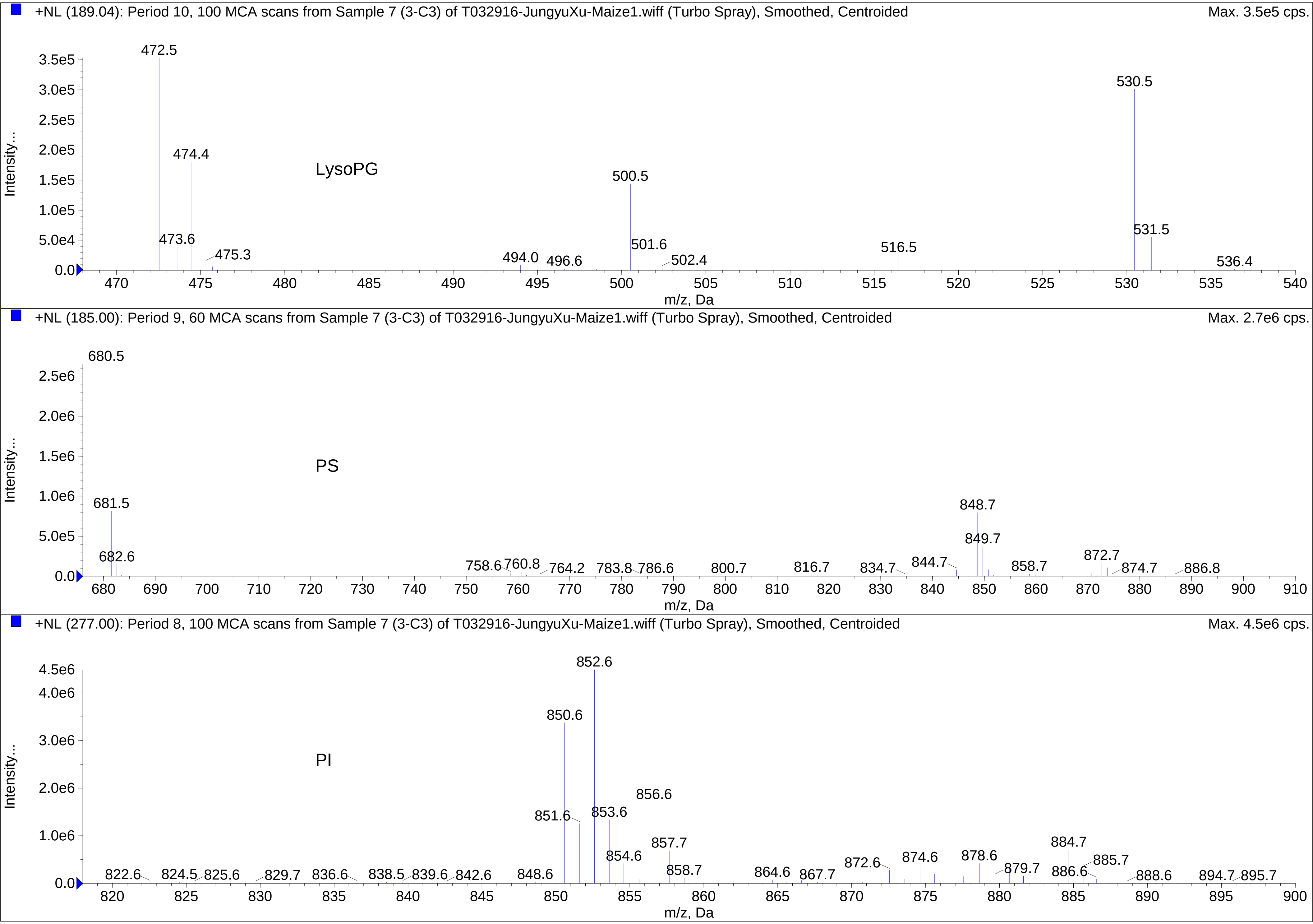
+NL (189.04): Period 10, 100 MCA scans from Sample 7 (3-C3) of T032916-JungyuXu-Maize1.wiff (Turbo Spray), Smoothed, Centroided
Max. 3.5e5 cps.
LysoPG
472.5
474.4
473.6
475.3
494.0
496.6
500.5
501.6
502.4
516.5
530.5
531.5
536.4
Intensity...
m/z, Da
+NL (185.00): Period 9, 60 MCA scans from Sample 7 (3-C3) of T032916-JungyuXu-Maize1.wiff (Turbo Spray), Smoothed, Centroided
Max. 2.7e6 cps.
PS
680.5
681.5
682.6
758.6
760.8
764.2
783.8
786.6
800.7
816.7
834.7
844.7
848.7
849.7
858.7
872.7
874.7
886.8
Intensity...
m/z, Da
+NL (277.00): Period 8, 100 MCA scans from Sample 7 (3-C3) of T032916-JungyuXu-Maize1.wiff (Turbo Spray), Smoothed, Centroided
Max. 4.5e6 cps.
PI
852.6
850.6
851.6
853.6
856.6
854.6
857.7
858.7
822.6
824.5
825.6
829.7
836.6
838.5
839.6
842.6
848.6
864.6
867.7
872.6
874.6
878.6
879.7
884.7
885.7
886.6
888.6
894.7
895.7
Intensity...
m/z, Da

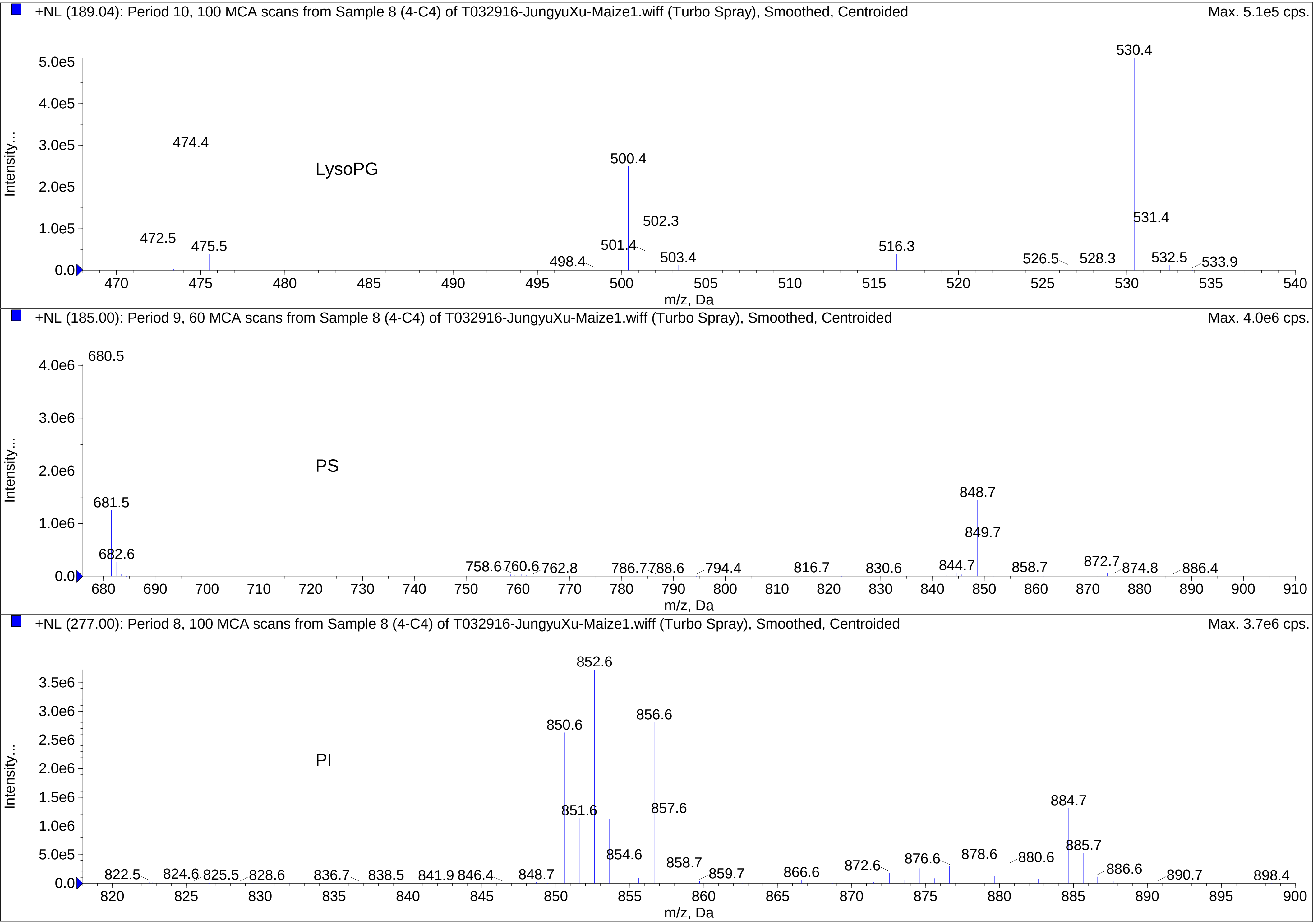

+NL (189.04): Period 10, 100 MCA scans from Sample 8 (4-C4) of T032916-JungyuXu-Maize1.wiff (Turbo Spray), Smoothed, Centroided
Max. 5.1e5 cps.
LysoPG
Intensity...
m/z, Da
530.4
474.4
500.4
502.3
531.4
472.5
475.5
501.4
516.3
498.4
503.4
526.5
528.3
532.5
533.9
+NL (185.00): Period 9, 60 MCA scans from Sample 8 (4-C4) of T032916-JungyuXu-Maize1.wiff (Turbo Spray), Smoothed, Centroided
Max. 4.0e6 cps.
PS
Intensity...
m/z, Da
680.5
681.5
682.6
848.7
849.7
758.6
760.6
762.8
786.7
788.6
794.4
816.7
830.6
844.7
858.7
872.7
874.8
886.4
+NL (277.00): Period 8, 100 MCA scans from Sample 8 (4-C4) of T032916-JungyuXu-Maize1.wiff (Turbo Spray), Smoothed, Centroided
Max. 3.7e6 cps.
PI
Intensity...
m/z, Da
852.6
850.6
856.6
851.6
857.6
884.7
885.7
854.6
858.7
822.5
824.6
825.5
828.6
836.7
838.5
841.9
846.4
848.7
859.7
866.6
872.6
876.6
878.6
880.6
886.6
890.7
898.4

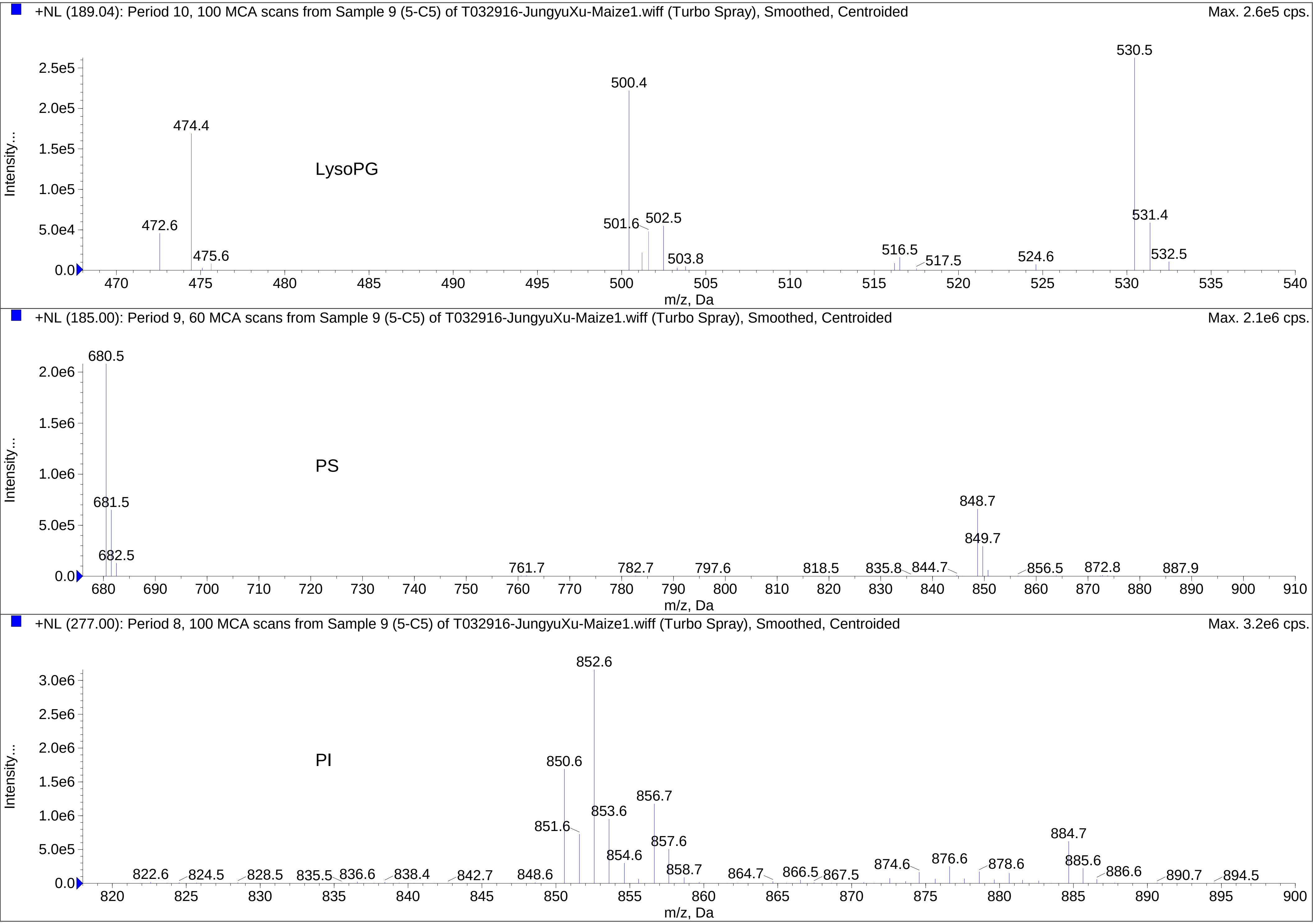
+NL (189.04): Period 10, 100 MCA scans from Sample 9 (5-C5) of T032916-JungyuXu-Maize1.wiff (Turbo Spray), Smoothed, Centroided
Max. 2.6e5 cps.
LysoPG
530.5
500.4
474.4
472.6
475.6
501.6
502.5
503.8
516.5
517.5
524.6
531.4
532.5
Intensity...
m/z, Da
+NL (185.00): Period 9, 60 MCA scans from Sample 9 (5-C5) of T032916-JungyuXu-Maize1.wiff (Turbo Spray), Smoothed, Centroided
Max. 2.1e6 cps.
PS
680.5
681.5
682.5
761.7
782.7
797.6
818.5
835.8
844.7
848.7
849.7
856.5
872.8
887.9
Intensity...
m/z, Da
+NL (277.00): Period 8, 100 MCA scans from Sample 9 (5-C5) of T032916-JungyuXu-Maize1.wiff (Turbo Spray), Smoothed, Centroided
Max. 3.2e6 cps.
PI
852.6
850.6
851.6
853.6
854.6
856.7
857.6
858.7
822.6
824.5
828.5
835.5
836.6
838.4
842.7
848.6
864.7
866.5
867.5
874.6
876.6
878.6
884.7
885.6
886.6
890.7
894.5
Intensity...
m/z, Da

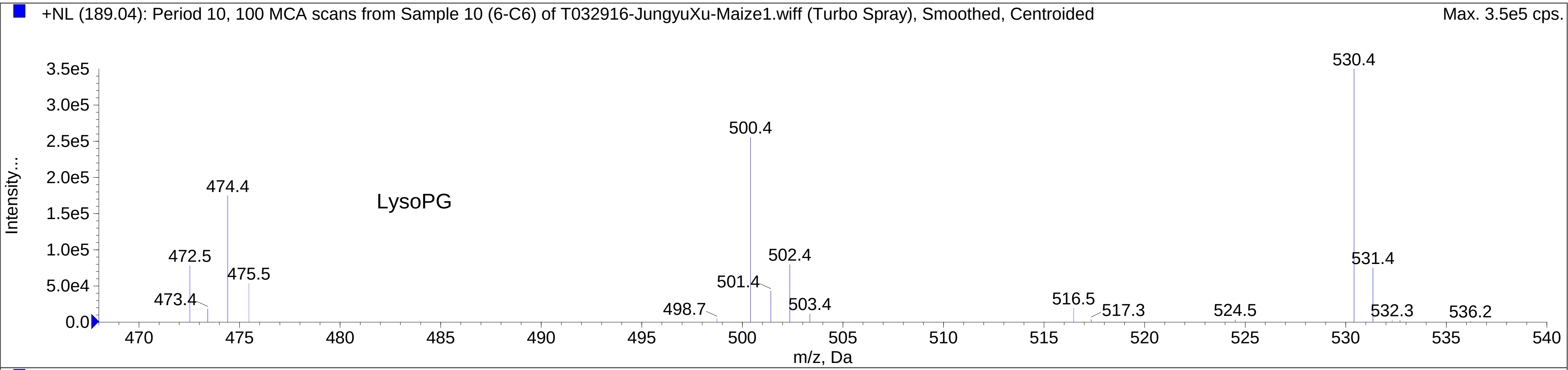
+NL (189.04): Period 10, 100 MCA scans from Sample 10 (6-C6) of T032916-JungyuXu-Maize1.wiff (Turbo Spray), Smoothed, Centroided
Max. 3.5e5 cps.
LysoPG
Intensity...
m/z, Da
530.4
500.4
474.4
472.5
475.5
473.4
502.4
501.4
503.4
498.7
516.5
517.3
524.5
531.4
532.3
536.2

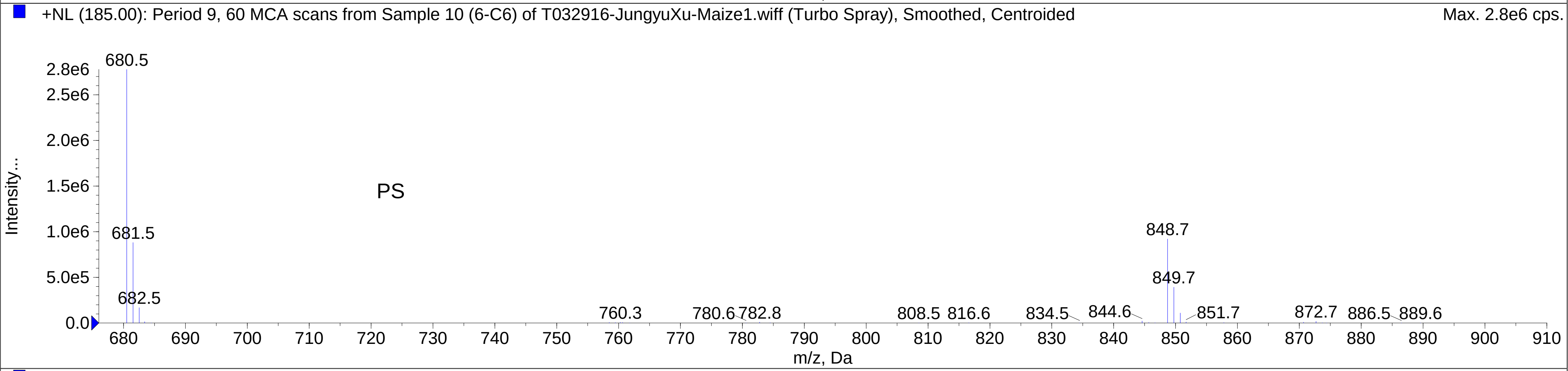
+NL (185.00): Period 9, 60 MCA scans from Sample 10 (6-C6) of T032916-JungyuXu-Maize1.wiff (Turbo Spray), Smoothed, Centroided
Max. 2.8e6 cps.
PS
Intensity...
m/z, Da
680.5
681.5
682.5
760.3
780.6
782.8
808.5
816.6
834.5
844.6
848.7
849.7
851.7
872.7
886.5
889.6

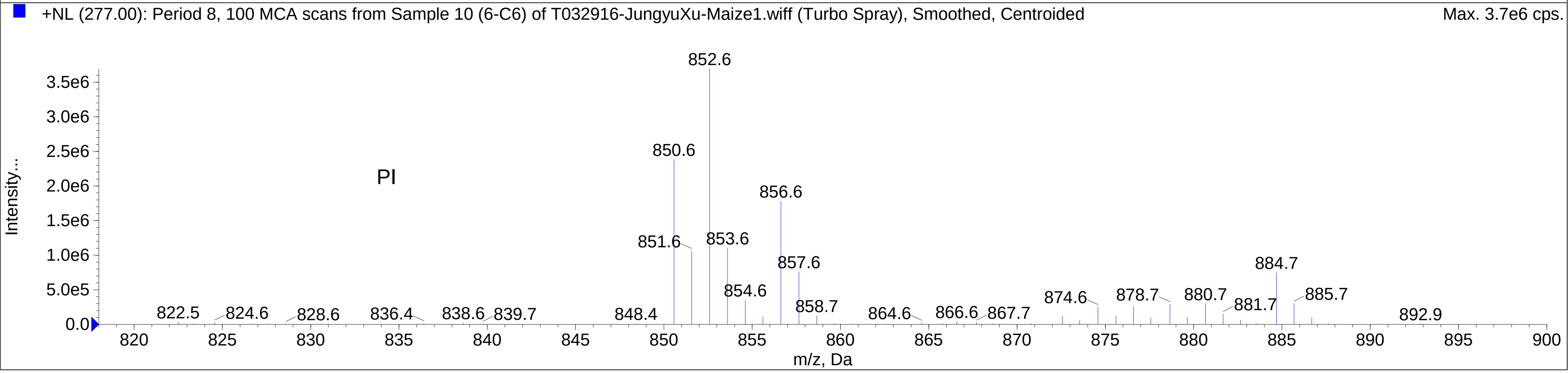
+NL (277.00): Period 8, 100 MCA scans from Sample 10 (6-C6) of T032916-JungyuXu-Maize1.wiff (Turbo Spray), Smoothed, Centroided
Max. 3.7e6 cps.
PI
Intensity...
m/z, Da
852.6
850.6
856.6
851.6
853.6
857.6
854.6
858.7
884.7
885.7
880.7
881.7
878.7
874.6
822.5
824.6
828.6
836.4
838.6
839.7
848.4
864.6
866.6
867.7
892.9

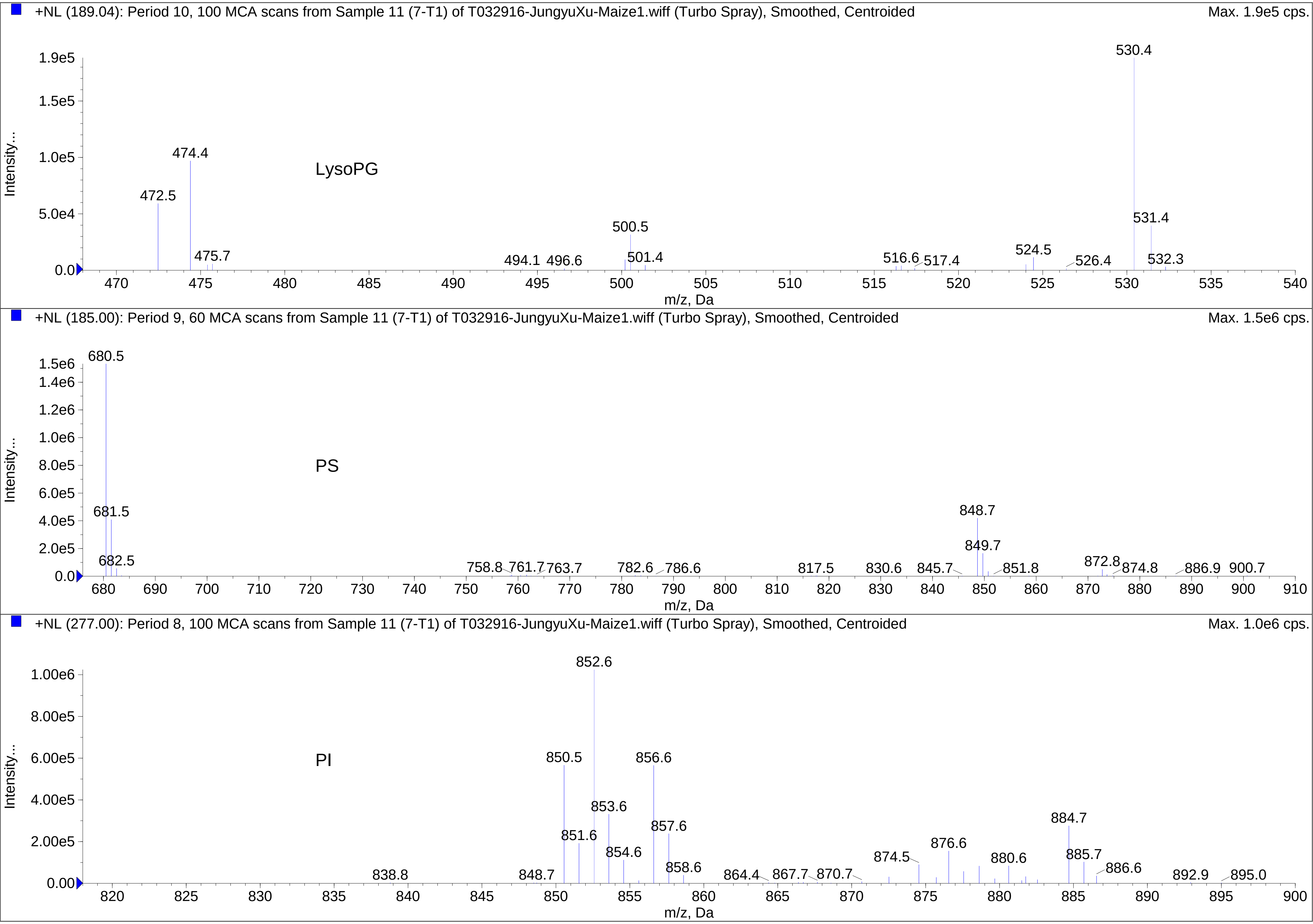

+NL (189.04): Period 10, 100 MCA scans from Sample 11 (7-T1) of T032916-JungyuXu-Maize1.wiff (Turbo Spray), Smoothed, Centroided
Max. 1.9e5 cps.
LysoPG
530.4
474.4
472.5
500.5
531.4
475.7
501.4
494.1
496.6
516.6
517.4
524.5
526.4
532.3
Intensity...
m/z, Da
+NL (185.00): Period 9, 60 MCA scans from Sample 11 (7-T1) of T032916-JungyuXu-Maize1.wiff (Turbo Spray), Smoothed, Centroided
Max. 1.5e6 cps.
PS
680.5
681.5
682.5
848.7
849.7
758.8
761.7
763.7
782.6
786.6
817.5
830.6
845.7
851.8
872.8
874.8
886.9
900.7
Intensity...
m/z, Da
+NL (277.00): Period 8, 100 MCA scans from Sample 11 (7-T1) of T032916-JungyuXu-Maize1.wiff (Turbo Spray), Smoothed, Centroided
Max. 1.0e6 cps.
PI
852.6
850.5
856.6
853.6
857.6
851.6
854.6
858.6
838.8
848.7
864.4
867.7
870.7
874.5
876.6
880.6
884.7
885.7
886.6
892.9
895.0
Intensity...
m/z, Da

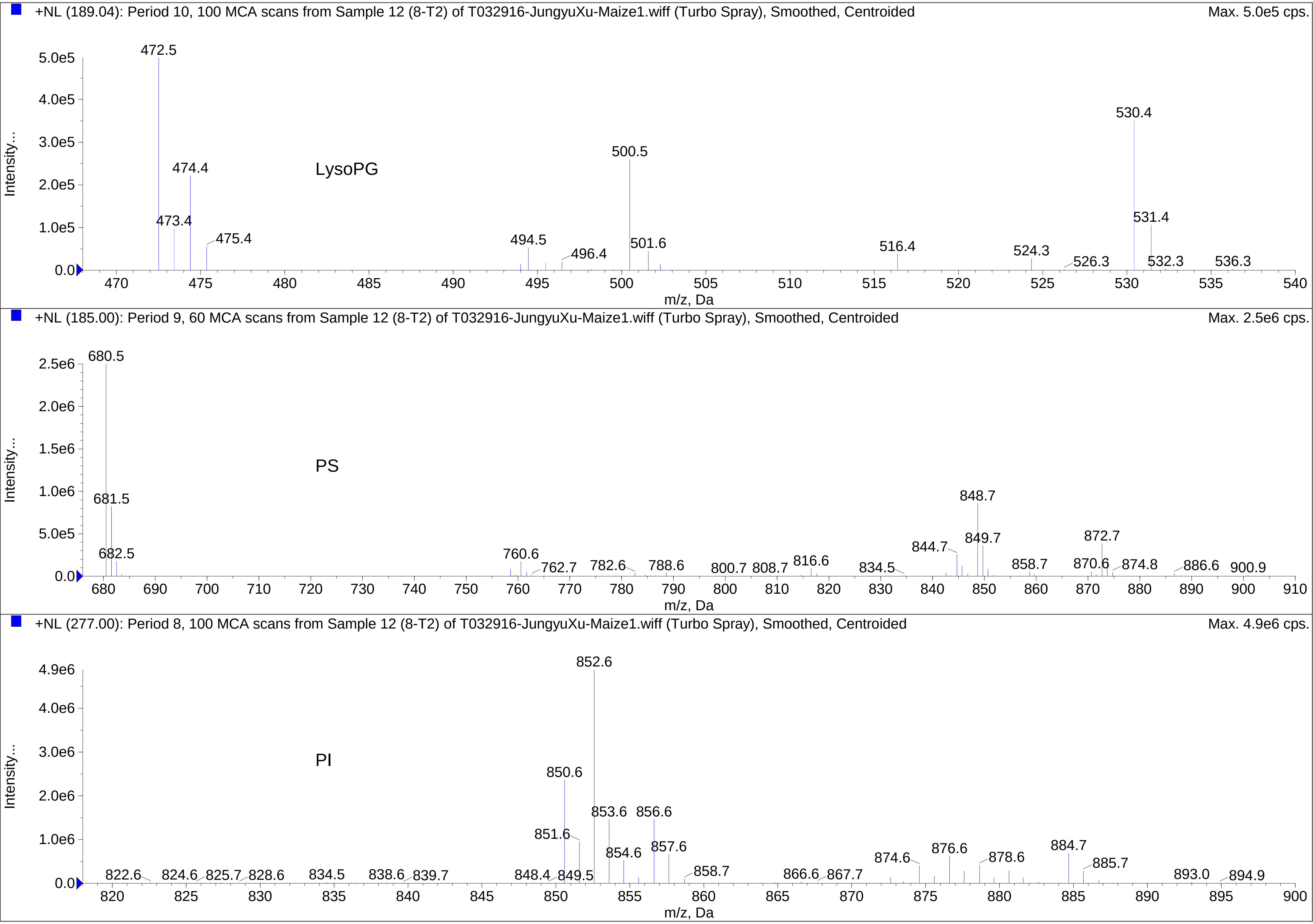
+NL (189.04): Period 10, 100 MCA scans from Sample 12 (8-T2) of T032916-JungyuXu-Maize1.wiff (Turbo Spray), Smoothed, Centroided
Max. 5.0e5 cps.
LysoPG
472.5
474.4
473.4
475.4
494.5
496.4
500.5
501.6
516.4
524.3
526.3
530.4
531.4
532.3
536.3
Intensity...
m/z, Da
+NL (185.00): Period 9, 60 MCA scans from Sample 12 (8-T2) of T032916-JungyuXu-Maize1.wiff (Turbo Spray), Smoothed, Centroided
Max. 2.5e6 cps.
PS
680.5
681.5
682.5
760.6
762.7
782.6
788.6
800.7
808.7
816.6
834.5
844.7
848.7
849.7
858.7
870.6
872.7
874.8
886.6
900.9
Intensity...
m/z, Da
+NL (277.00): Period 8, 100 MCA scans from Sample 12 (8-T2) of T032916-JungyuXu-Maize1.wiff (Turbo Spray), Smoothed, Centroided
Max. 4.9e6 cps.
PI
852.6
850.6
851.6
853.6
856.6
854.6
857.6
858.7
822.6
824.6
825.7
828.6
834.5
838.6
839.7
848.4
849.5
866.6
867.7
874.6
876.6
878.6
884.7
885.7
893.0
894.9
Intensity...
m/z, Da

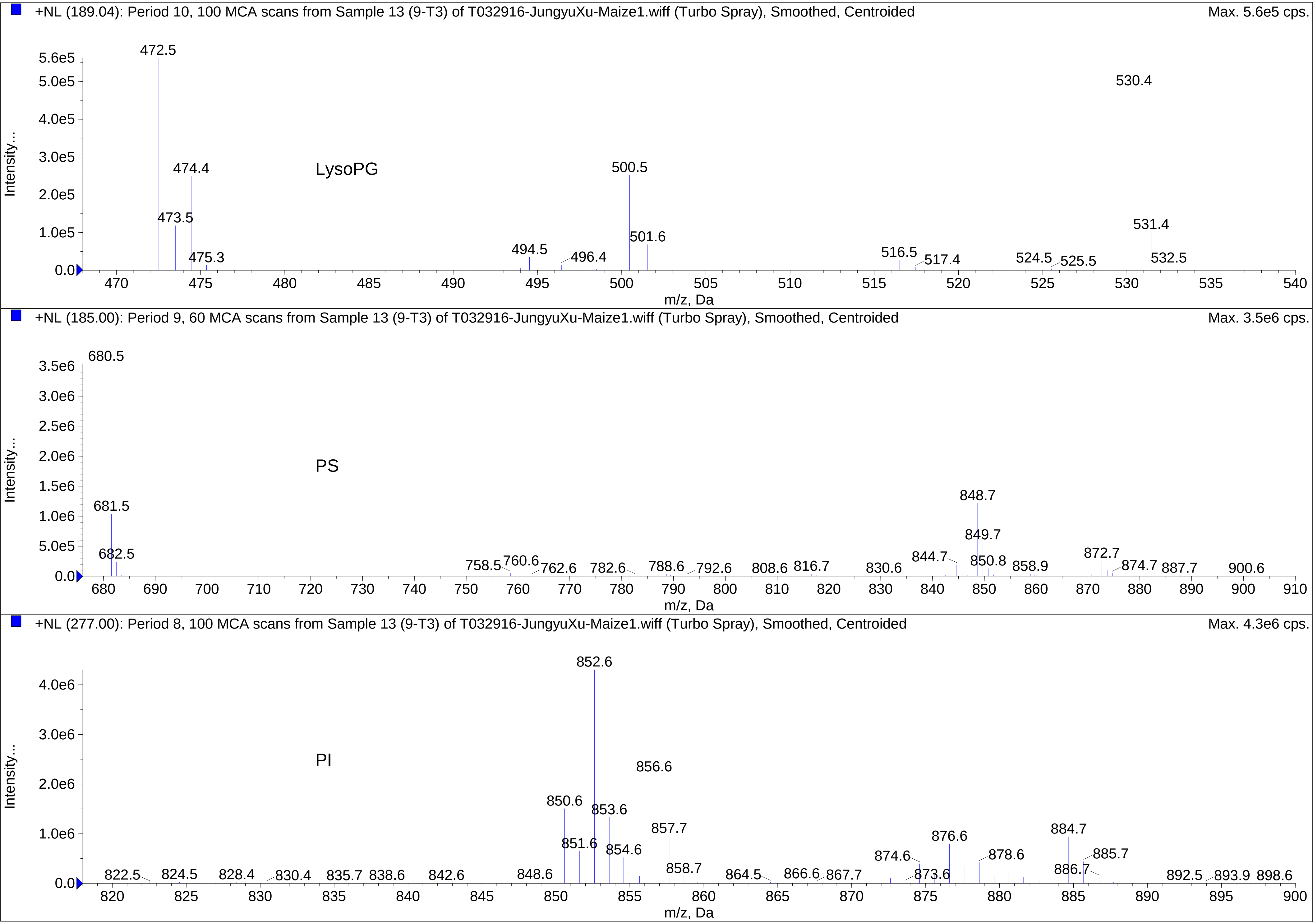

+NL (189.04): Period 10, 100 MCA scans from Sample 13 (9-T3) of T032916-JungyuXu-Maize1.wiff (Turbo Spray), Smoothed, Centroided
Max. 5.6e5 cps.
LysoPG
472.5
474.4
473.5
475.3
494.5
496.4
500.5
501.6
516.5
517.4
524.5
525.5
530.4
531.4
532.5
Intensity...
m/z, Da
+NL (185.00): Period 9, 60 MCA scans from Sample 13 (9-T3) of T032916-JungyuXu-Maize1.wiff (Turbo Spray), Smoothed, Centroided
Max. 3.5e6 cps.
PS
680.5
681.5
682.5
758.5
760.6
762.6
782.6
788.6
792.6
808.6
816.7
830.6
844.7
848.7
849.7
850.8
858.9
872.7
874.7
887.7
900.6
Intensity...
m/z, Da
+NL (277.00): Period 8, 100 MCA scans from Sample 13 (9-T3) of T032916-JungyuXu-Maize1.wiff (Turbo Spray), Smoothed, Centroided
Max. 4.3e6 cps.
PI
852.6
856.6
850.6
853.6
857.7
851.6
854.6
858.7
822.5
824.5
828.4
830.4
835.7
838.6
842.6
848.6
864.5
866.6
867.7
874.6
873.6
876.6
878.6
884.7
885.7
886.7
892.5
893.9
898.6
Intensity...
m/z, Da

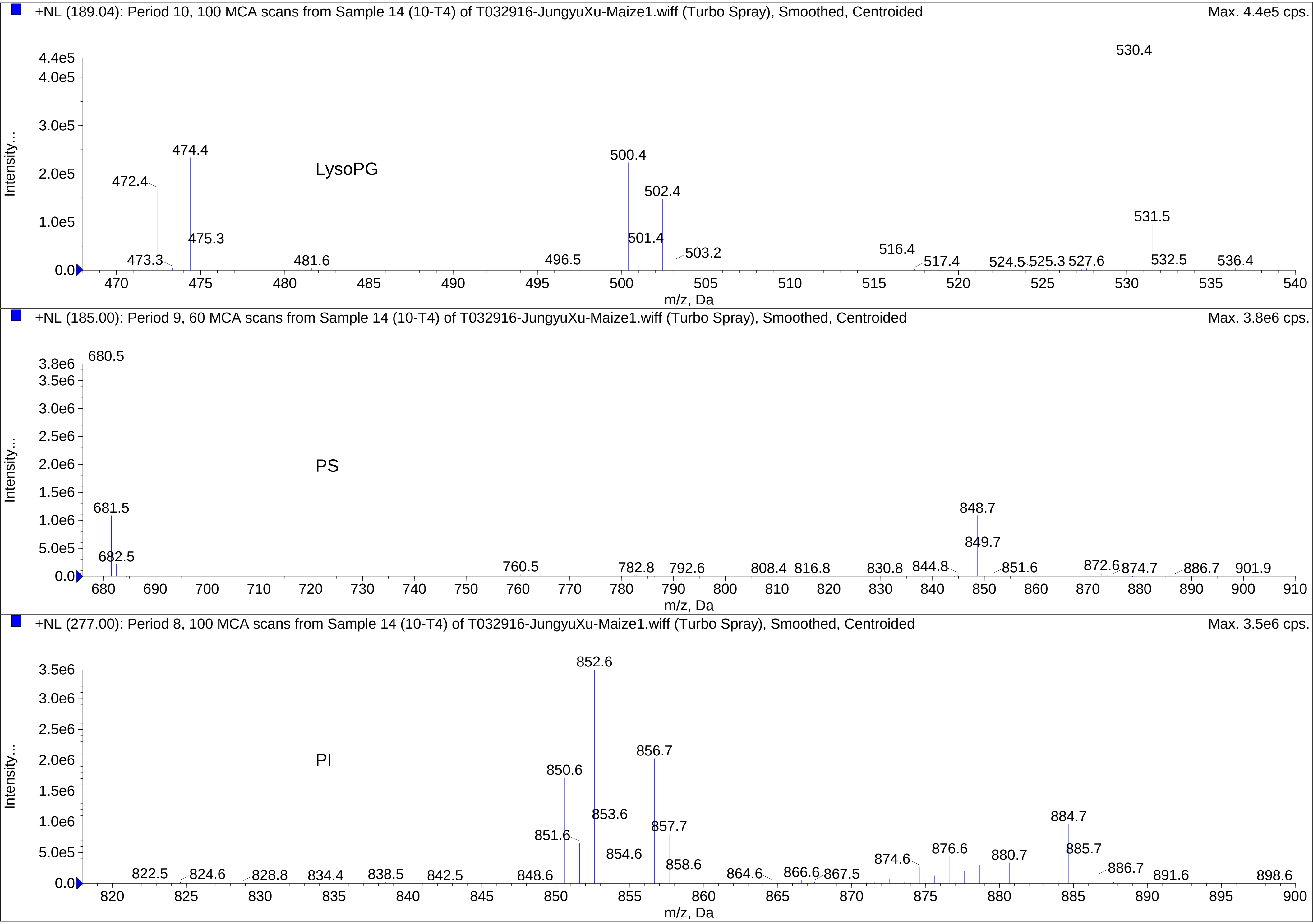
+NL (189.04): Period 10, 100 MCA scans from Sample 14 (10-T4) of T032916-JungyuXu-Maize1.wiff (Turbo Spray), Smoothed, Centroided
Max. 4.4e5 cps.
LysoPG
Intensity...
m/z, Da
530.4
474.4
500.4
472.4
502.4
531.5
475.3
501.4
503.2
516.4
473.3
481.6
496.5
517.4
524.5
525.3
527.6
532.5
536.4
+NL (185.00): Period 9, 60 MCA scans from Sample 14 (10-T4) of T032916-JungyuXu-Maize1.wiff (Turbo Spray), Smoothed, Centroided
Max. 3.8e6 cps.
PS
Intensity...
m/z, Da
680.5
681.5
682.5
848.7
849.7
760.5
782.8
792.6
808.4
816.8
830.8
844.8
851.6
872.6
874.7
886.7
901.9
+NL (277.00): Period 8, 100 MCA scans from Sample 14 (10-T4) of T032916-JungyuXu-Maize1.wiff (Turbo Spray), Smoothed, Centroided
Max. 3.5e6 cps.
PI
Intensity...
m/z, Da
852.6
856.7
850.6
853.6
857.7
851.6
854.6
858.6
884.7
885.7
886.7
876.6
880.7
874.6
822.5
824.6
828.8
834.4
838.5
842.5
848.6
864.6
866.6
867.5
891.6
898.6

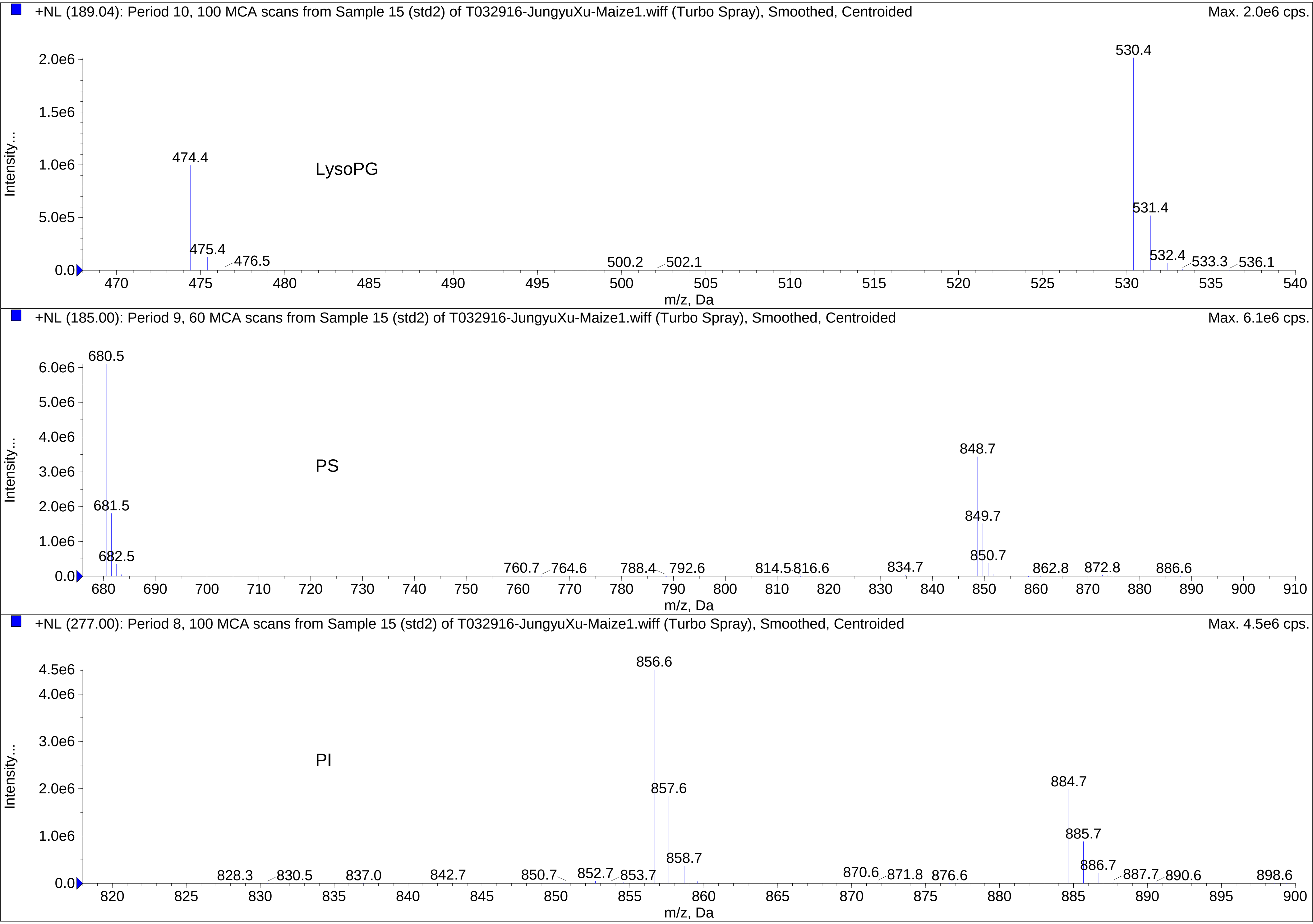

+NL (189.04): Period 10, 100 MCA scans from Sample 15 (std2) of T032916-JungyuXu-Maize1.wiff (Turbo Spray), Smoothed, Centroided
Max. 2.0e6 cps.
LysoPG
Intensity...
m/z, Da
530.4
474.4
531.4
475.4
476.5
500.2
502.1
532.4
533.3
536.1
+NL (185.00): Period 9, 60 MCA scans from Sample 15 (std2) of T032916-JungyuXu-Maize1.wiff (Turbo Spray), Smoothed, Centroided
Max. 6.1e6 cps.
PS
Intensity...
m/z, Da
680.5
681.5
682.5
760.7
764.6
788.4
792.6
814.5
816.6
834.7
848.7
849.7
850.7
862.8
872.8
886.6
+NL (277.00): Period 8, 100 MCA scans from Sample 15 (std2) of T032916-JungyuXu-Maize1.wiff (Turbo Spray), Smoothed, Centroided
Max. 4.5e6 cps.
PI
Intensity...
m/z, Da
856.6
857.6
858.7
828.3
830.5
837.0
842.7
850.7
852.7
853.7
870.6
871.8
876.6
884.7
885.7
886.7
887.7
890.6
898.6

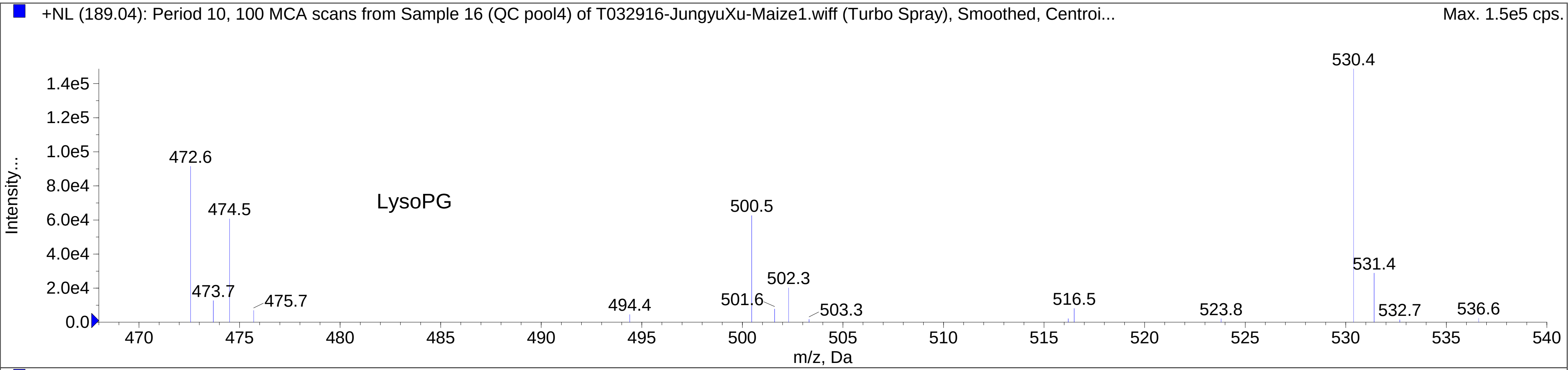
+NL (189.04): Period 10, 100 MCA scans from Sample 16 (QC pool4) of T032916-JungyuXu-Maize1.wiff (Turbo Spray), Smoothed, Centroi...
Max. 1.5e5 cps.
LysoPG
Intensity...
m/z, Da
472.6
473.7
474.5
475.7
494.4
500.5
501.6
502.3
503.3
516.5
523.8
530.4
531.4
532.7
536.6

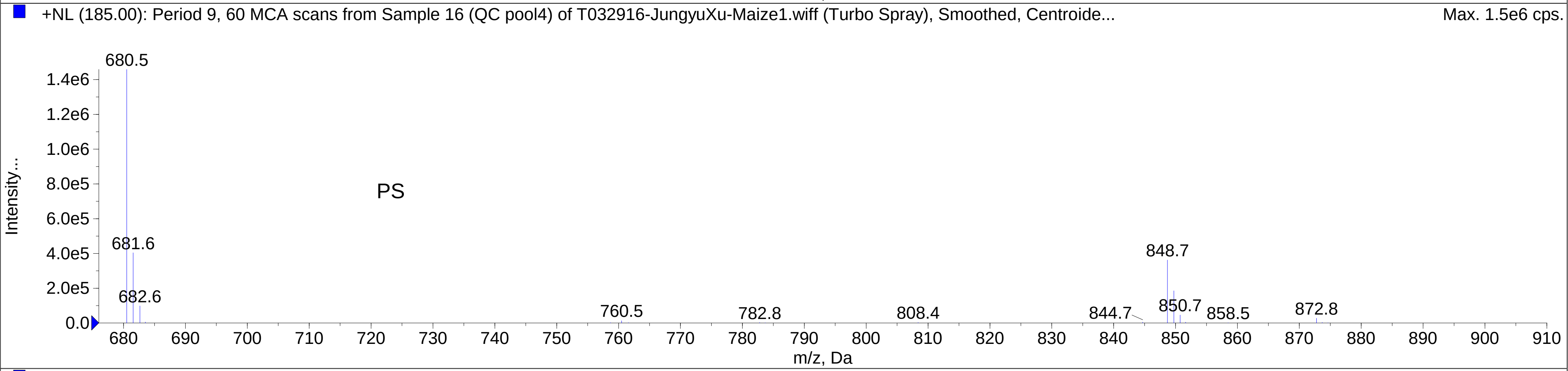
+NL (185.00): Period 9, 60 MCA scans from Sample 16 (QC pool4) of T032916-JungyuXu-Maize1.wiff (Turbo Spray), Smoothed, Centroide...
Max. 1.5e6 cps.
PS
Intensity...
m/z, Da
680.5
681.6
682.6
760.5
782.8
808.4
844.7
848.7
850.7
858.5
872.8

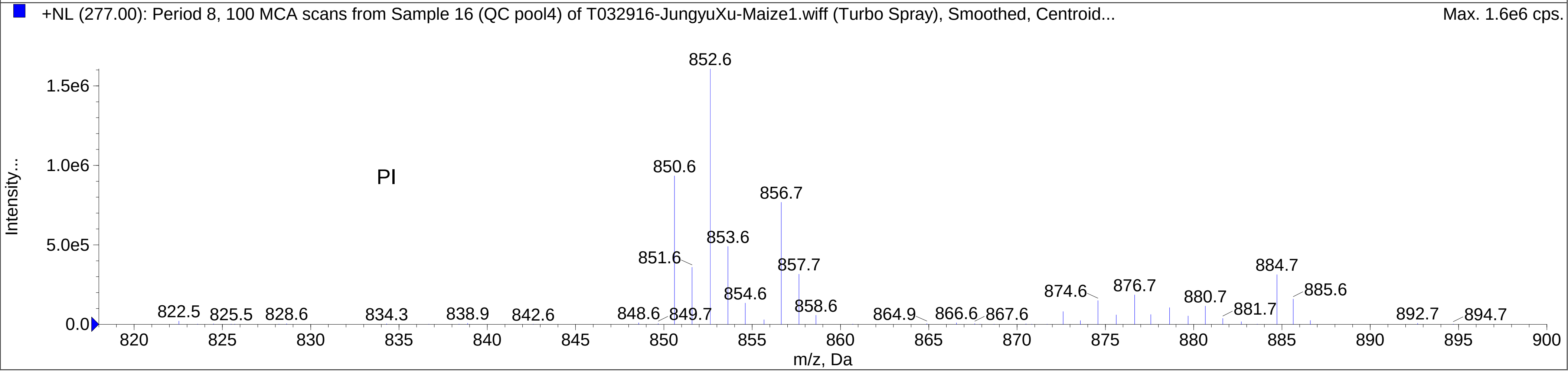
+NL (277.00): Period 8, 100 MCA scans from Sample 16 (QC pool4) of T032916-JungyuXu-Maize1.wiff (Turbo Spray), Smoothed, Centroid...
Max. 1.6e6 cps.
PI
Intensity...
m/z, Da
822.5
825.5
828.6
834.3
838.9
842.6
848.6
849.7
850.6
851.6
852.6
853.6
854.6
856.7
857.7
858.6
864.9
866.6
867.6
874.6
876.7
880.7
881.7
884.7
885.6
892.7
894.7

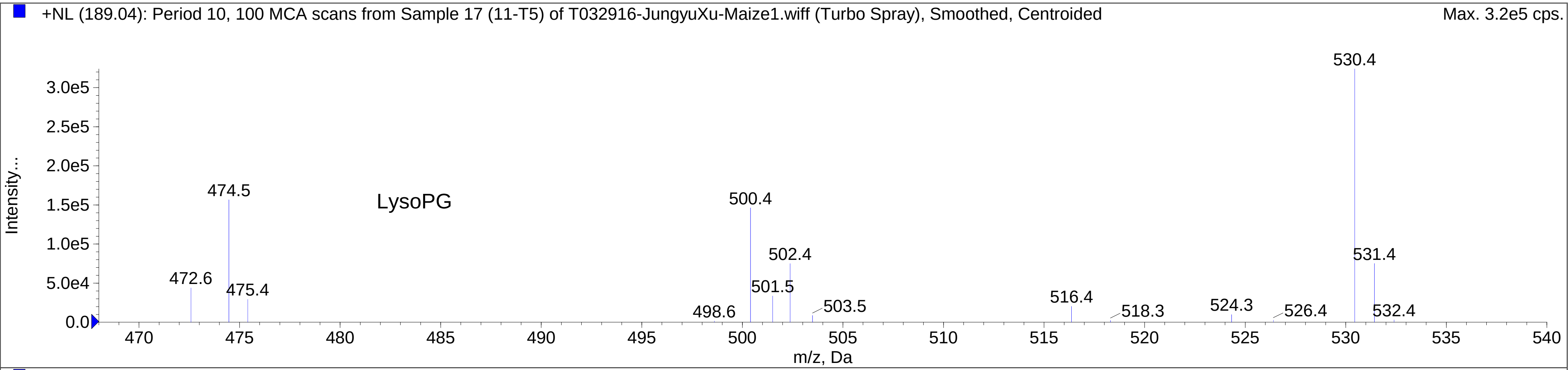
+NL (189.04): Period 10, 100 MCA scans from Sample 17 (11-T5) of T032916-JungyuXu-Maize1.wiff (Turbo Spray), Smoothed, Centroided
Max. 3.2e5 cps.
LysoPG
Intensity...
m/z, Da
530.4
474.5
500.4
502.4
531.4
472.6
475.4
501.5
503.5
516.4
518.3
524.3
526.4
532.4
498.6

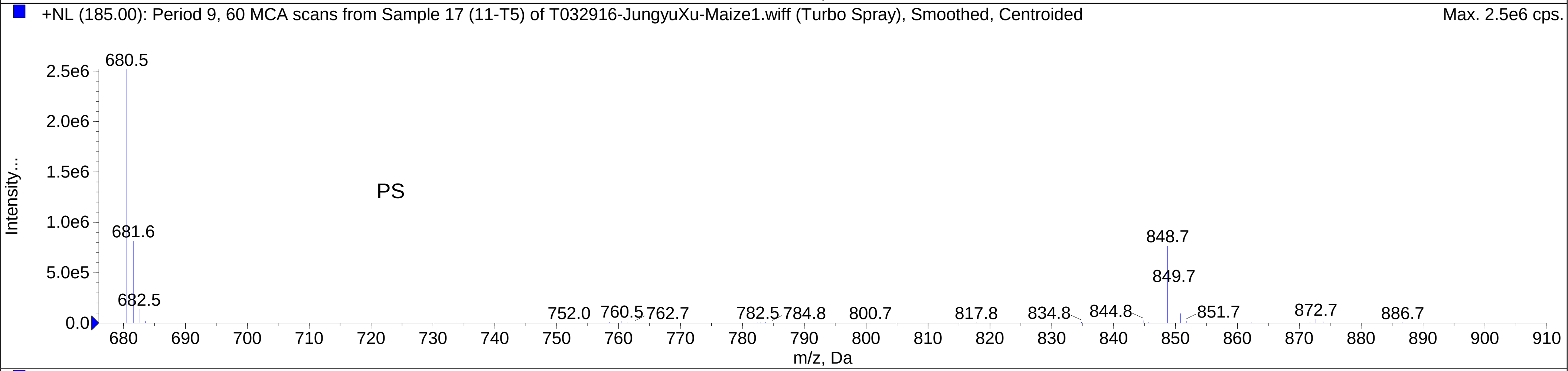
+NL (185.00): Period 9, 60 MCA scans from Sample 17 (11-T5) of T032916-JungyuXu-Maize1.wiff (Turbo Spray), Smoothed, Centroided
Max. 2.5e6 cps.
PS
Intensity...
m/z, Da
680.5
681.6
682.5
848.7
849.7
752.0
760.5
762.7
782.5
784.8
800.7
817.8
834.8
844.8
851.7
872.7
886.7

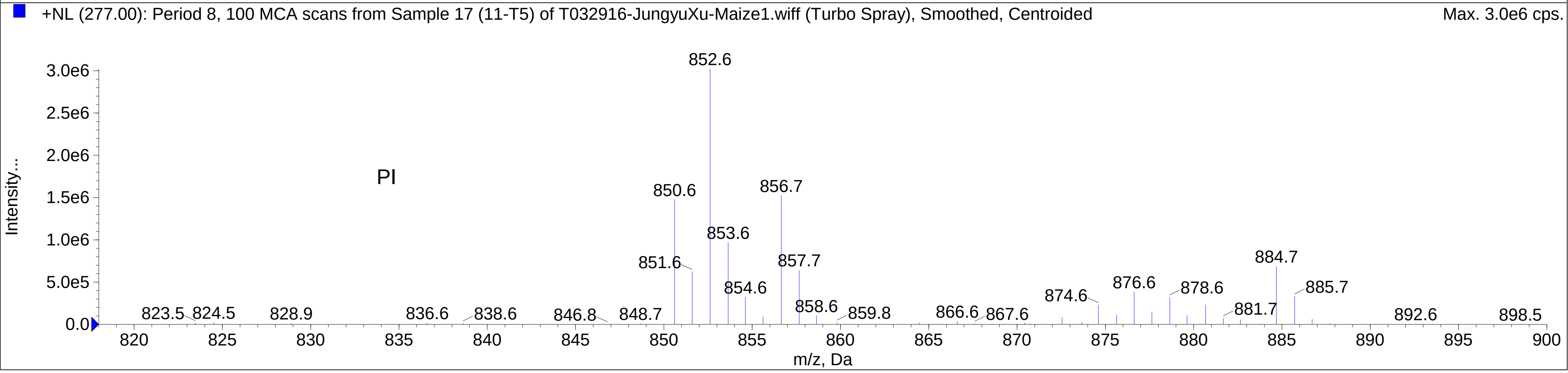
+NL (277.00): Period 8, 100 MCA scans from Sample 17 (11-T5) of T032916-JungyuXu-Maize1.wiff (Turbo Spray), Smoothed, Centroided
Max. 3.0e6 cps.
PI
Intensity...
m/z, Da
852.6
850.6
856.7
853.6
851.6
857.7
854.6
858.6
859.8
884.7
876.6
878.6
885.7
874.6
881.7
823.5
824.5
828.9
836.6
838.6
846.8
848.7
866.6
867.6
892.6
898.5

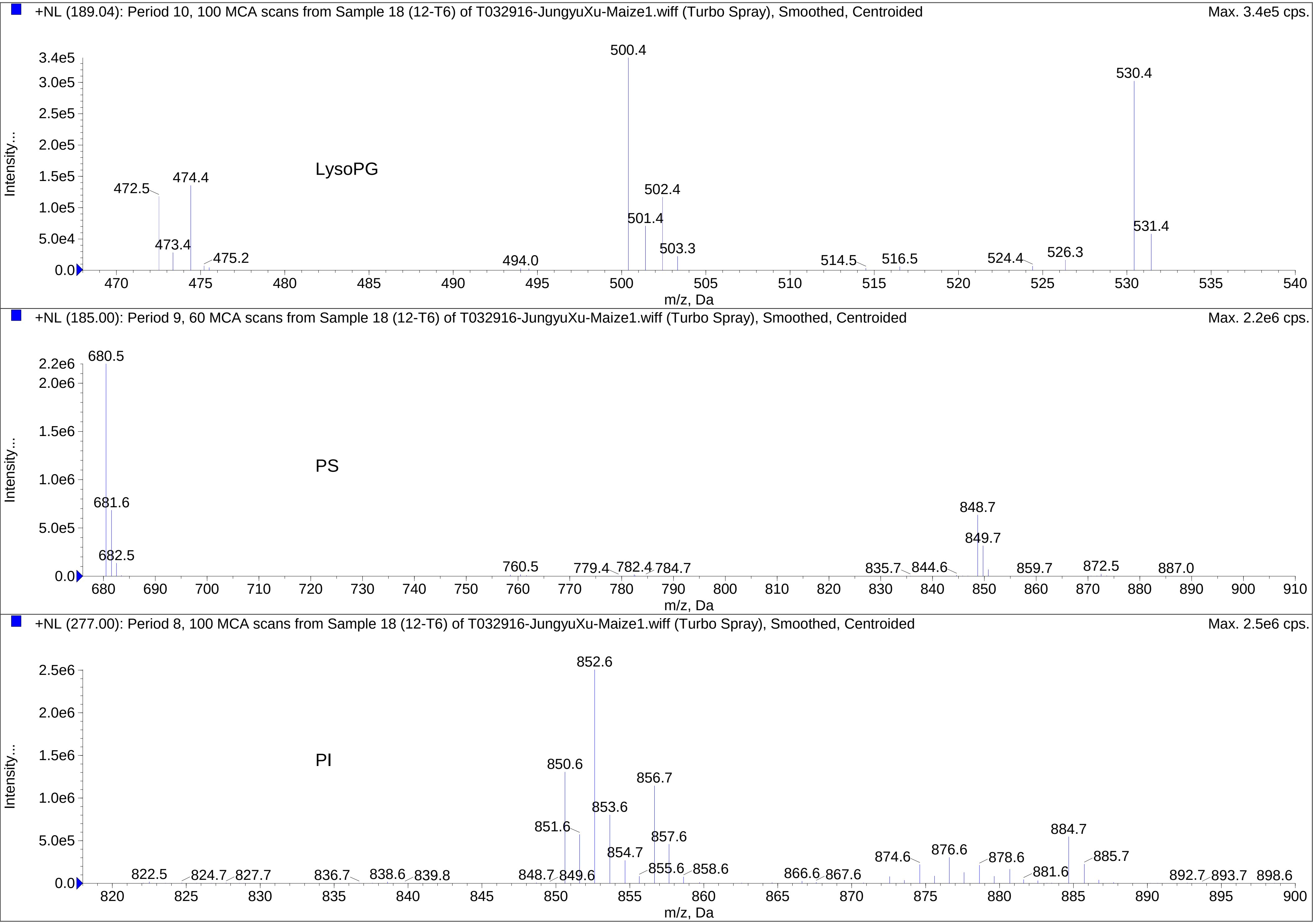
+NL (189.04): Period 10, 100 MCA scans from Sample 18 (12-T6) of T032916-JungyuXu-Maize1.wiff (Turbo Spray), Smoothed, Centroided
Max. 3.4e5 cps.
LysoPG
472.5
473.4
474.4
475.2
494.0
500.4
501.4
502.4
503.3
514.5
516.5
524.4
526.3
530.4
531.4
Intensity...
m/z, Da
+NL (185.00): Period 9, 60 MCA scans from Sample 18 (12-T6) of T032916-JungyuXu-Maize1.wiff (Turbo Spray), Smoothed, Centroided
Max. 2.2e6 cps.
PS
680.5
681.6
682.5
760.5
779.4
782.4
784.7
835.7
844.6
848.7
849.7
859.7
872.5
887.0
Intensity...
m/z, Da
+NL (277.00): Period 8, 100 MCA scans from Sample 18 (12-T6) of T032916-JungyuXu-Maize1.wiff (Turbo Spray), Smoothed, Centroided
Max. 2.5e6 cps.
PI
822.5
824.7
827.7
836.7
838.6
839.8
848.7
849.6
850.6
851.6
852.6
853.6
854.7
855.6
856.7
857.6
858.6
866.6
867.6
874.6
876.6
878.6
881.6
884.7
885.7
892.7
893.7
898.6
Intensity...
m/z, Da

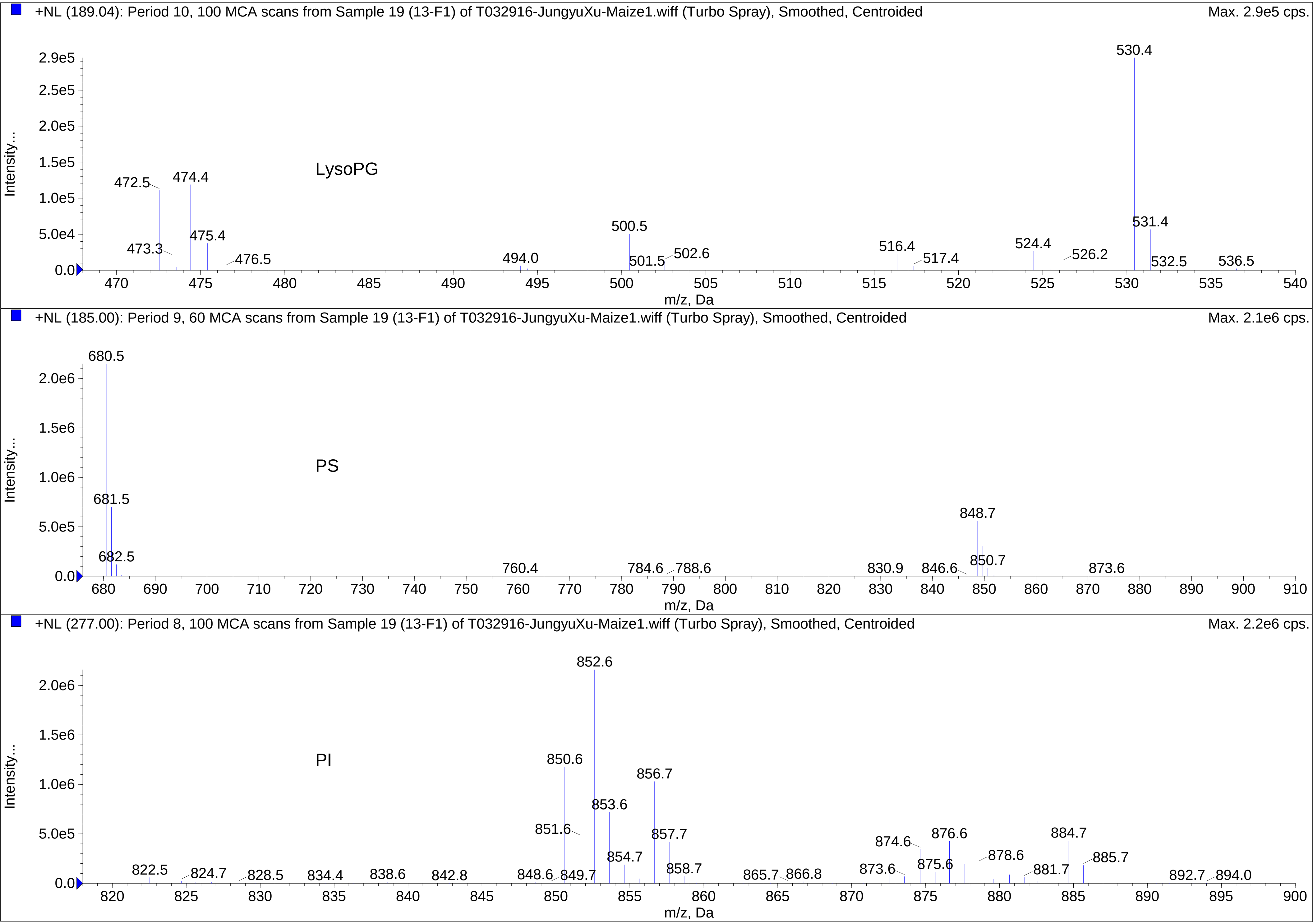

+NL (189.04): Period 10, 100 MCA scans from Sample 19 (13-F1) of T032916-JungyuXu-Maize1.wiff (Turbo Spray), Smoothed, Centroided
Max. 2.9e5 cps.
LysoPG
Intensity...
m/z, Da
530.4
472.5
474.4
473.3
475.4
476.5
494.0
500.5
501.5
502.6
516.4
517.4
524.4
526.2
531.4
532.5
536.5
+NL (185.00): Period 9, 60 MCA scans from Sample 19 (13-F1) of T032916-JungyuXu-Maize1.wiff (Turbo Spray), Smoothed, Centroided
Max. 2.1e6 cps.
PS
Intensity...
m/z, Da
680.5
681.5
682.5
760.4
784.6
788.6
830.9
846.6
848.7
850.7
873.6
+NL (277.00): Period 8, 100 MCA scans from Sample 19 (13-F1) of T032916-JungyuXu-Maize1.wiff (Turbo Spray), Smoothed, Centroided
Max. 2.2e6 cps.
PI
Intensity...
m/z, Da
852.6
850.6
856.7
853.6
851.6
857.7
854.7
858.7
822.5
824.7
828.5
834.4
838.6
842.8
848.6
849.7
865.7
866.8
873.6
874.6
875.6
876.6
878.6
881.7
884.7
885.7
892.7
894.0

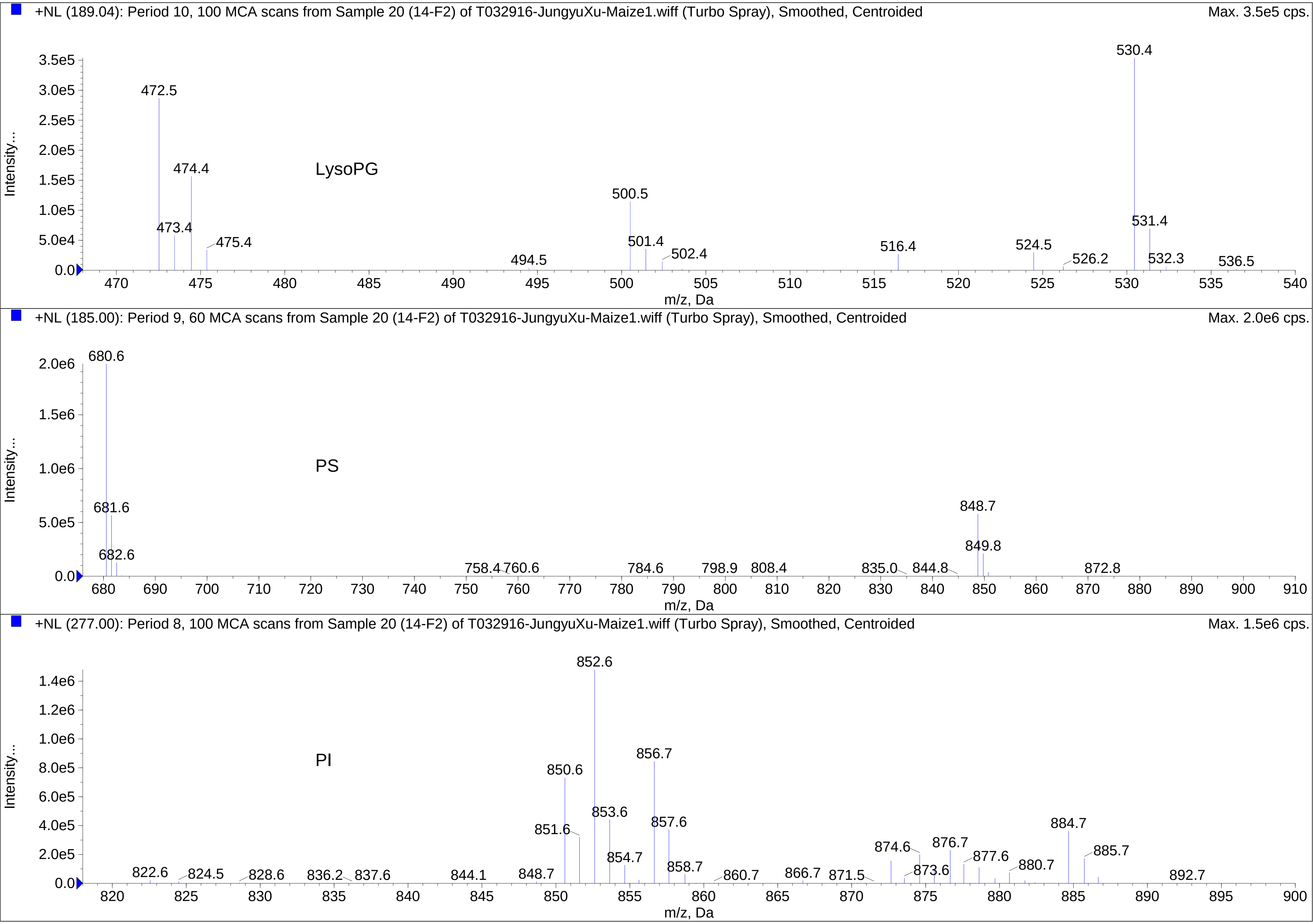

+NL (189.04): Period 10, 100 MCA scans from Sample 20 (14-F2) of T032916-JungyuXu-Maize1.wiff (Turbo Spray), Smoothed, Centroided
Max. 3.5e5 cps.
LysoPG
Intensity...
m/z, Da
472.5
473.4
474.4
475.4
494.5
500.5
501.4
502.4
516.4
524.5
526.2
530.4
531.4
532.3
536.5
+NL (185.00): Period 9, 60 MCA scans from Sample 20 (14-F2) of T032916-JungyuXu-Maize1.wiff (Turbo Spray), Smoothed, Centroided
Max. 2.0e6 cps.
PS
Intensity...
m/z, Da
680.6
681.6
682.6
758.4
760.6
784.6
798.9
808.4
835.0
844.8
848.7
849.8
872.8
+NL (277.00): Period 8, 100 MCA scans from Sample 20 (14-F2) of T032916-JungyuXu-Maize1.wiff (Turbo Spray), Smoothed, Centroided
Max. 1.5e6 cps.
PI
Intensity...
m/z, Da
822.6
824.5
828.6
836.2
837.6
844.1
848.7
850.6
851.6
852.6
853.6
854.7
856.7
857.6
858.7
860.7
866.7
871.5
873.6
874.6
876.7
877.6
880.7
884.7
885.7
892.7

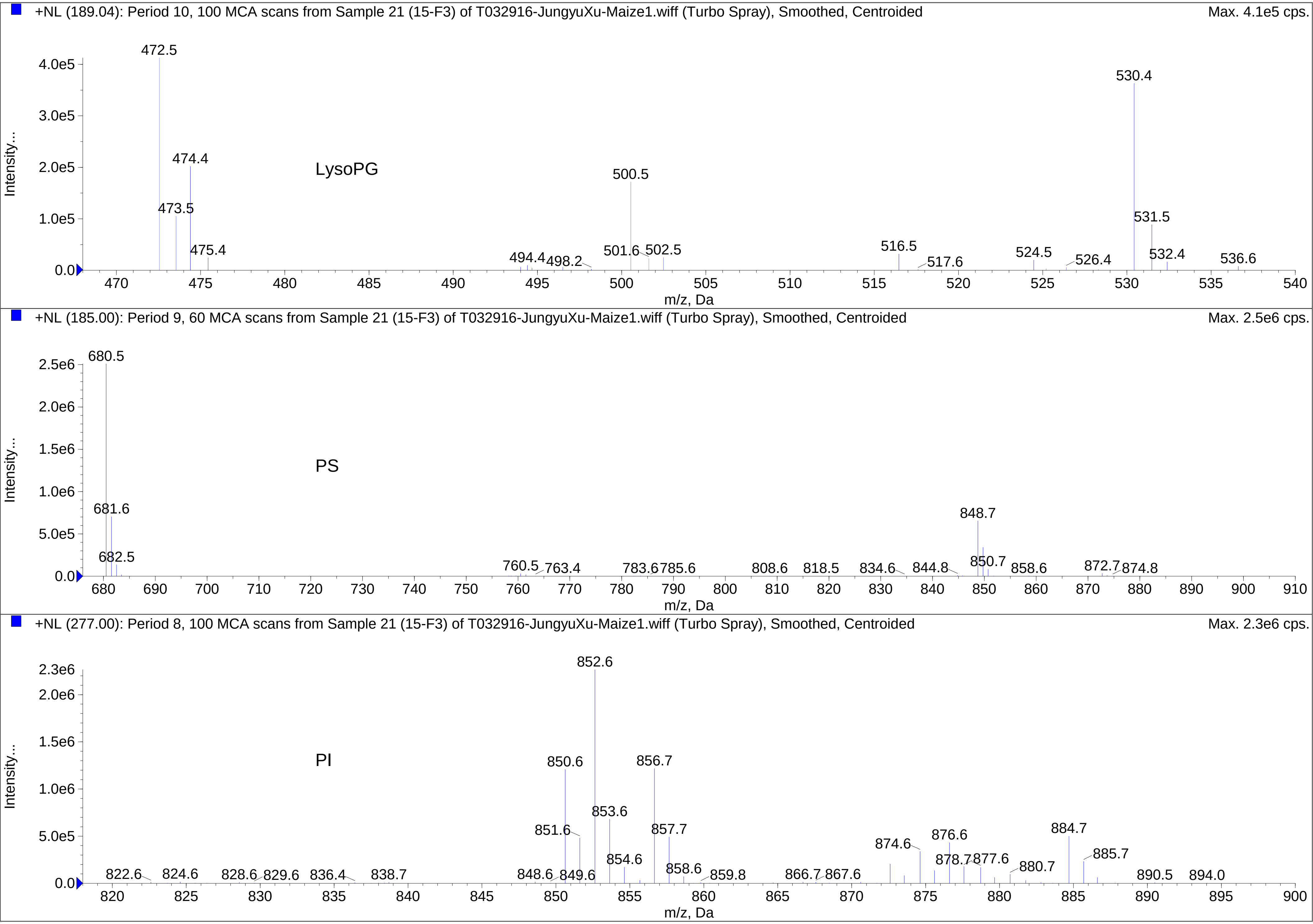

+NL (189.04): Period 10, 100 MCA scans from Sample 21 (15-F3) of T032916-JungyuXu-Maize1.wiff (Turbo Spray), Smoothed, Centroided
Max. 4.1e5 cps.
LysoPG
Intensity...
m/z, Da
472.5
474.4
473.5
475.4
494.4
498.2
500.5
501.6
502.5
516.5
517.6
524.5
526.4
530.4
531.5
532.4
536.6
+NL (185.00): Period 9, 60 MCA scans from Sample 21 (15-F3) of T032916-JungyuXu-Maize1.wiff (Turbo Spray), Smoothed, Centroided
Max. 2.5e6 cps.
PS
Intensity...
m/z, Da
680.5
681.6
682.5
760.5
763.4
783.6
785.6
808.6
818.5
834.6
844.8
848.7
850.7
858.6
872.7
874.8
+NL (277.00): Period 8, 100 MCA scans from Sample 21 (15-F3) of T032916-JungyuXu-Maize1.wiff (Turbo Spray), Smoothed, Centroided
Max. 2.3e6 cps.
PI
Intensity...
m/z, Da
852.6
850.6
856.7
853.6
851.6
857.7
854.6
858.6
859.8
822.6
824.6
828.6
829.6
836.4
838.7
848.6
849.6
866.7
867.6
874.6
876.6
878.7
877.6
880.7
884.7
885.7
890.5
894.0

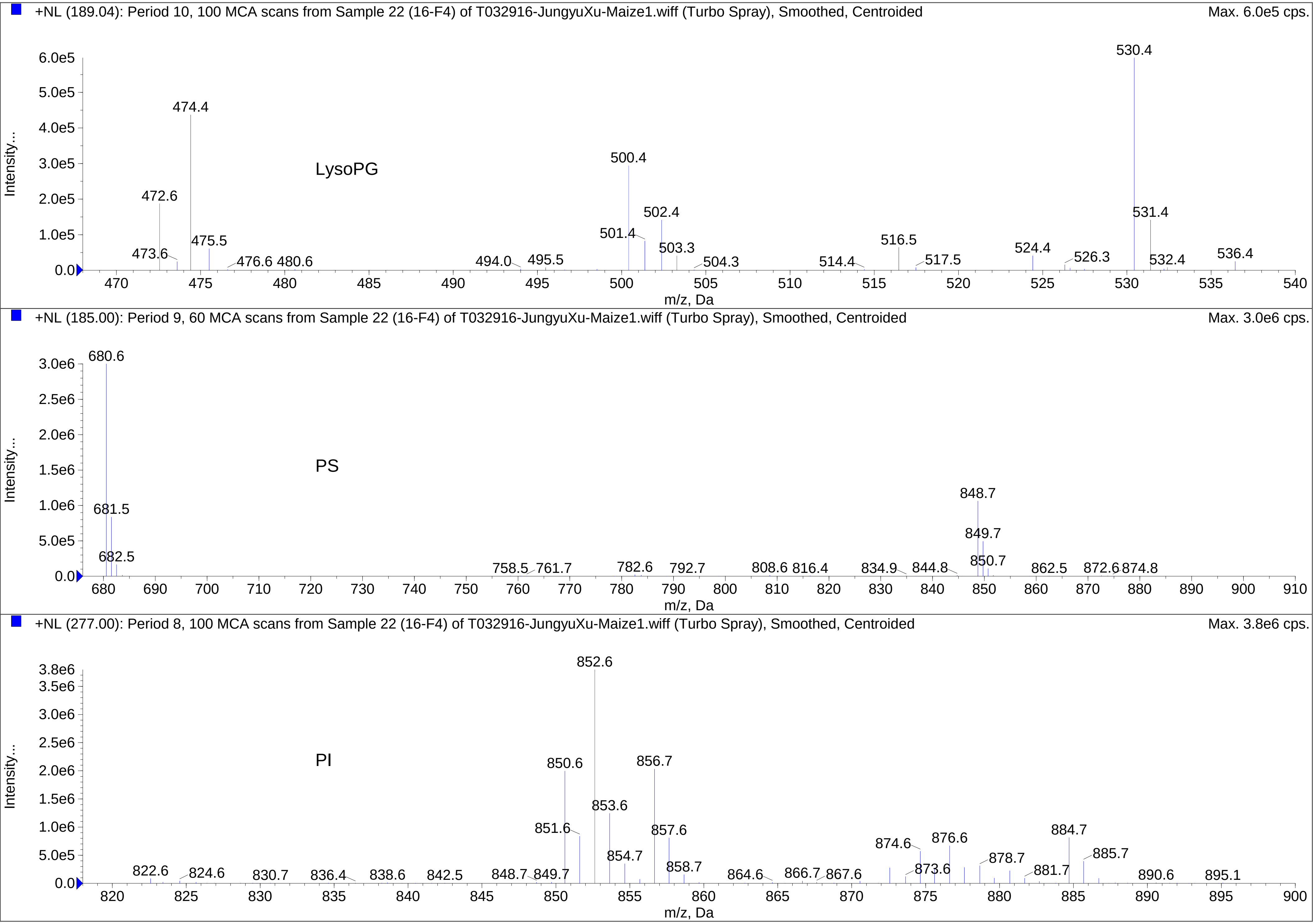

+NL (189.04): Period 10, 100 MCA scans from Sample 22 (16-F4) of T032916-JungyuXu-Maize1.wiff (Turbo Spray), Smoothed, Centroided
Max. 6.0e5 cps.
LysoPG
Intensity...
m/z, Da
530.4
474.4
500.4
472.6
502.4
531.4
501.4
475.5
516.5
473.6
503.3
524.4
536.4
526.3
476.6
480.6
494.0
495.5
504.3
514.4
517.5
532.4
+NL (185.00): Period 9, 60 MCA scans from Sample 22 (16-F4) of T032916-JungyuXu-Maize1.wiff (Turbo Spray), Smoothed, Centroided
Max. 3.0e6 cps.
PS
680.6
681.5
682.5
848.7
849.7
850.7
758.5
761.7
782.6
792.7
808.6
816.4
834.9
844.8
862.5
872.6
874.8
+NL (277.00): Period 8, 100 MCA scans from Sample 22 (16-F4) of T032916-JungyuXu-Maize1.wiff (Turbo Spray), Smoothed, Centroided
Max. 3.8e6 cps.
PI
852.6
850.6
856.7
853.6
851.6
857.6
874.6
876.6
884.7
854.7
878.7
885.7
858.7
873.6
881.7
822.6
824.6
830.7
836.4
838.6
842.5
848.7
849.7
864.6
866.7
867.6
890.6
895.1

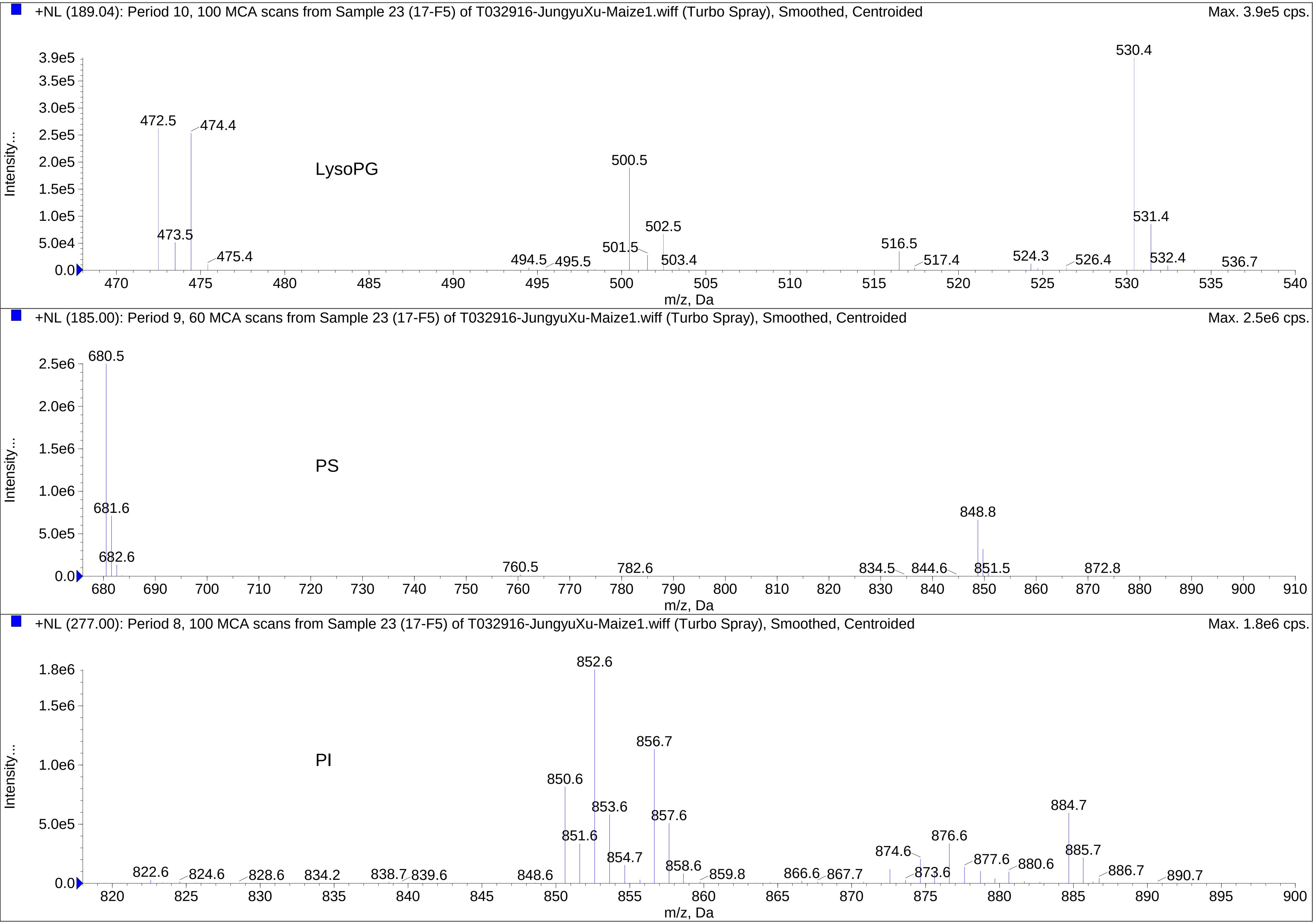

+NL (189.04): Period 10, 100 MCA scans from Sample 23 (17-F5) of T032916-JungyuXu-Maize1.wiff (Turbo Spray), Smoothed, Centroided
Max. 3.9e5 cps.
LysoPG
Intensity...
m/z, Da
472.5
473.5
474.4
475.4
494.5
495.5
500.5
501.5
502.5
503.4
516.5
517.4
524.3
526.4
530.4
531.4
532.4
536.7
+NL (185.00): Period 9, 60 MCA scans from Sample 23 (17-F5) of T032916-JungyuXu-Maize1.wiff (Turbo Spray), Smoothed, Centroided
Max. 2.5e6 cps.
PS
Intensity...
m/z, Da
680.5
681.6
682.6
760.5
782.6
834.5
844.6
848.8
851.5
872.8
+NL (277.00): Period 8, 100 MCA scans from Sample 23 (17-F5) of T032916-JungyuXu-Maize1.wiff (Turbo Spray), Smoothed, Centroided
Max. 1.8e6 cps.
PI
Intensity...
m/z, Da
822.6
824.6
828.6
834.2
838.7
839.6
848.6
850.6
851.6
852.6
853.6
854.7
856.7
857.6
858.6
859.8
866.6
867.7
873.6
874.6
876.6
877.6
880.6
884.7
885.7
886.7
890.7

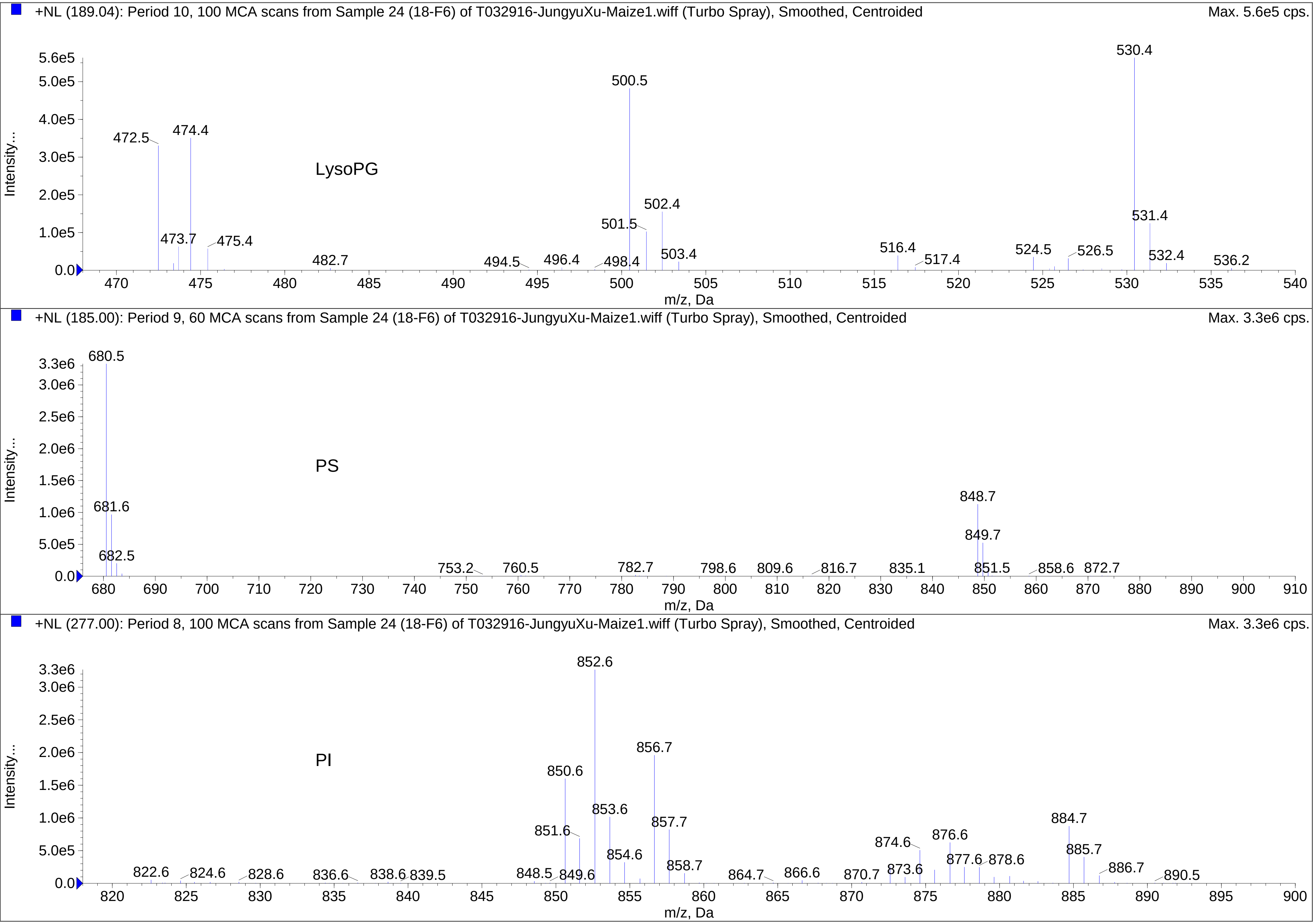
+NL (189.04): Period 10, 100 MCA scans from Sample 24 (18-F6) of T032916-JungyuXu-Maize1.wiff (Turbo Spray), Smoothed, Centroided
Max. 5.6e5 cps.
LysoPG
Intensity...
m/z, Da
530.4
500.5
474.4
472.5
502.4
501.5
531.4
473.7
475.4
516.4
524.5
526.5
532.4
503.4
482.7
494.5
496.4
498.4
517.4
536.2
+NL (185.00): Period 9, 60 MCA scans from Sample 24 (18-F6) of T032916-JungyuXu-Maize1.wiff (Turbo Spray), Smoothed, Centroided
Max. 3.3e6 cps.
PS
Intensity...
m/z, Da
680.5
681.6
682.5
848.7
849.7
851.5
753.2
760.5
782.7
798.6
809.6
816.7
835.1
858.6
872.7
+NL (277.00): Period 8, 100 MCA scans from Sample 24 (18-F6) of T032916-JungyuXu-Maize1.wiff (Turbo Spray), Smoothed, Centroided
Max. 3.3e6 cps.
PI
Intensity...
m/z, Da
852.6
856.7
850.6
853.6
857.7
851.6
854.6
858.7
884.7
885.7
876.6
874.6
877.6
878.6
873.6
886.7
890.5
822.6
824.6
828.6
836.6
838.6
839.5
848.5
849.6
864.7
866.6
870.7

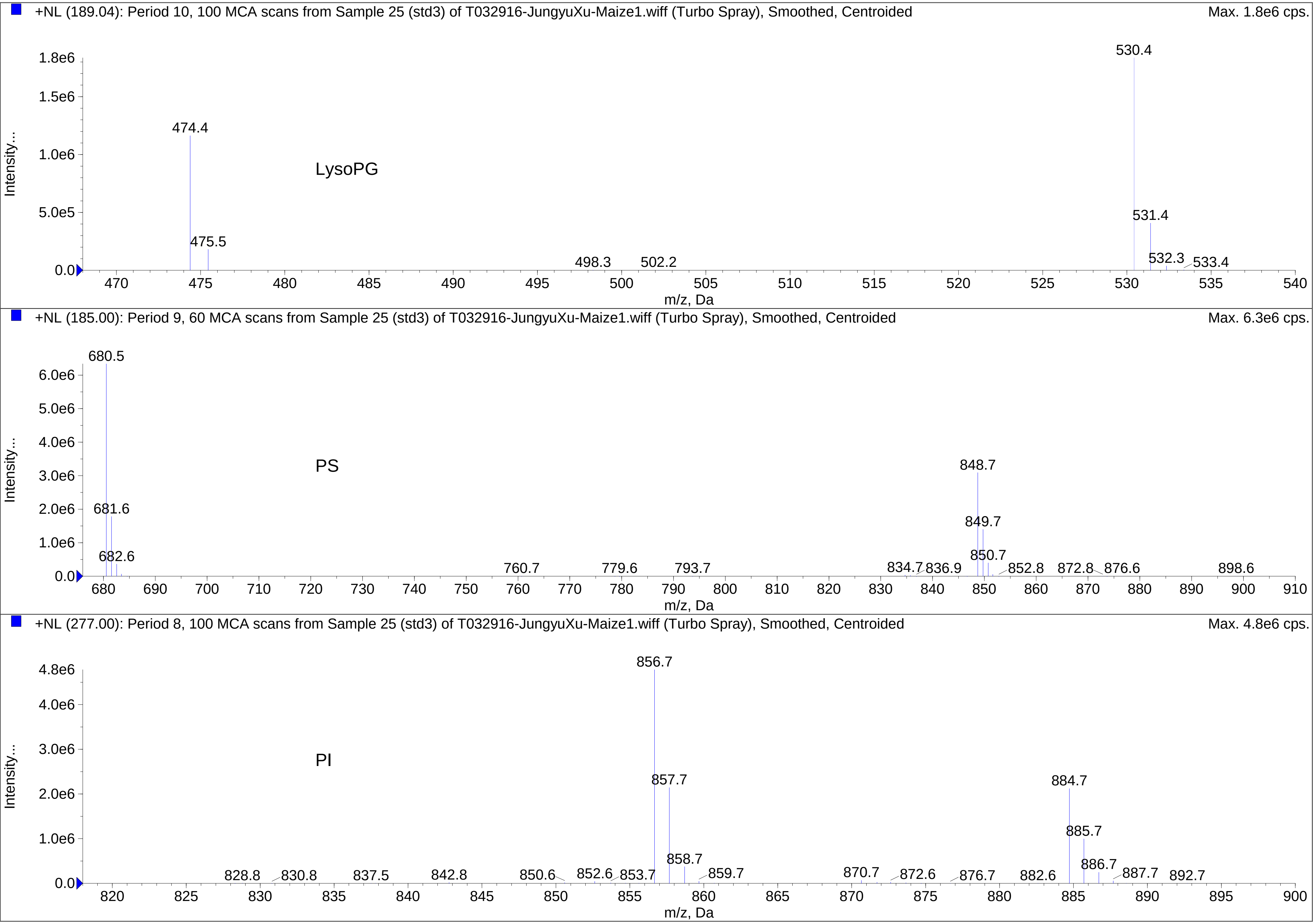

+NL (189.04): Period 10, 100 MCA scans from Sample 25 (std3) of T032916-JungyuXu-Maize1.wiff (Turbo Spray), Smoothed, Centroided
Max. 1.8e6 cps.
LysoPG
Intensity...
1.8e6
1.5e6
1.0e6
5.0e5
0.0
474.4
475.5
498.3
502.2
530.4
531.4
532.3
533.4
470
475
480
485
490
495
500
505
510
515
520
525
530
535
540
m/z, Da
+NL (185.00): Period 9, 60 MCA scans from Sample 25 (std3) of T032916-JungyuXu-Maize1.wiff (Turbo Spray), Smoothed, Centroided
Max. 6.3e6 cps.
PS
Intensity...
6.0e6
5.0e6
4.0e6
3.0e6
2.0e6
1.0e6
0.0
680.5
681.6
682.6
760.7
779.6
793.7
834.7
836.9
848.7
849.7
850.7
852.8
872.8
876.6
898.6
680
690
700
710
720
730
740
750
760
770
780
790
800
810
820
830
840
850
860
870
880
890
900
910
m/z, Da
+NL (277.00): Period 8, 100 MCA scans from Sample 25 (std3) of T032916-JungyuXu-Maize1.wiff (Turbo Spray), Smoothed, Centroided
Max. 4.8e6 cps.
PI
Intensity...
4.8e6
4.0e6
3.0e6
2.0e6
1.0e6
0.0
856.7
857.7
858.7
859.7
828.8
830.8
837.5
842.8
850.6
852.6
853.7
870.7
872.6
876.7
882.6
884.7
885.7
886.7
887.7
892.7
820
825
830
835
840
845
850
855
860
865
870
875
880
885
890
895
900
m/z, Da

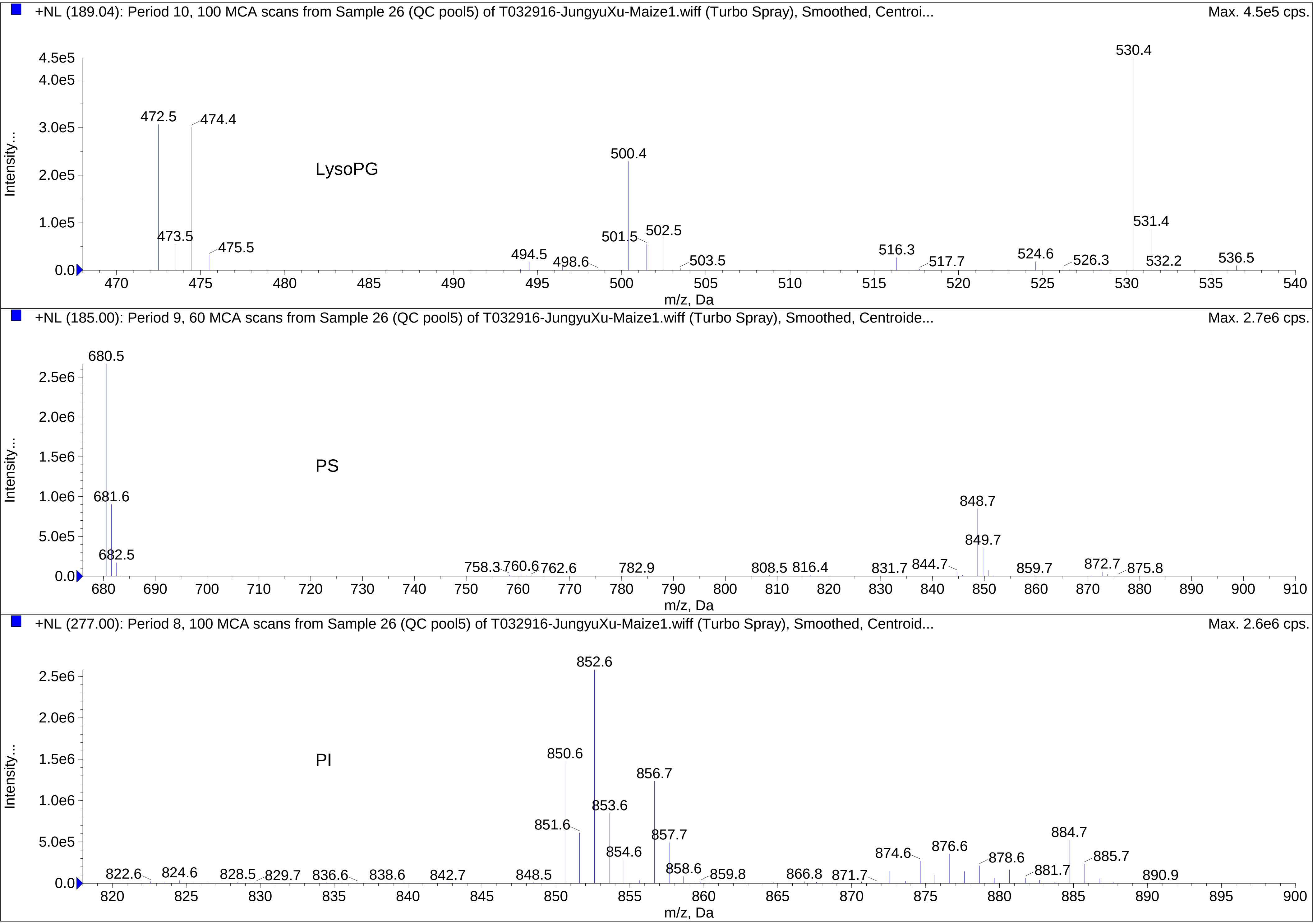

+NL (189.04): Period 10, 100 MCA scans from Sample 26 (QC pool5) of T032916-JungyuXu-Maize1.wiff (Turbo Spray), Smoothed, Centroi...
Max. 4.5e5 cps.
LysoPG
Intensity...
m/z, Da
472.5
473.5
474.4
475.5
494.5
498.6
500.4
501.5
502.5
503.5
516.3
517.7
524.6
526.3
530.4
531.4
532.2
536.5
+NL (185.00): Period 9, 60 MCA scans from Sample 26 (QC pool5) of T032916-JungyuXu-Maize1.wiff (Turbo Spray), Smoothed, Centroide...
Max. 2.7e6 cps.
PS
Intensity...
m/z, Da
680.5
681.6
682.5
758.3
760.6
762.6
782.9
808.5
816.4
831.7
844.7
848.7
849.7
859.7
872.7
875.8
+NL (277.00): Period 8, 100 MCA scans from Sample 26 (QC pool5) of T032916-JungyuXu-Maize1.wiff (Turbo Spray), Smoothed, Centroid...
Max. 2.6e6 cps.
PI
Intensity...
m/z, Da
822.6
824.6
828.5
829.7
836.6
838.6
842.7
848.5
850.6
851.6
852.6
853.6
854.6
856.7
857.7
858.6
859.8
866.8
871.7
874.6
876.6
878.6
881.7
884.7
885.7
890.9

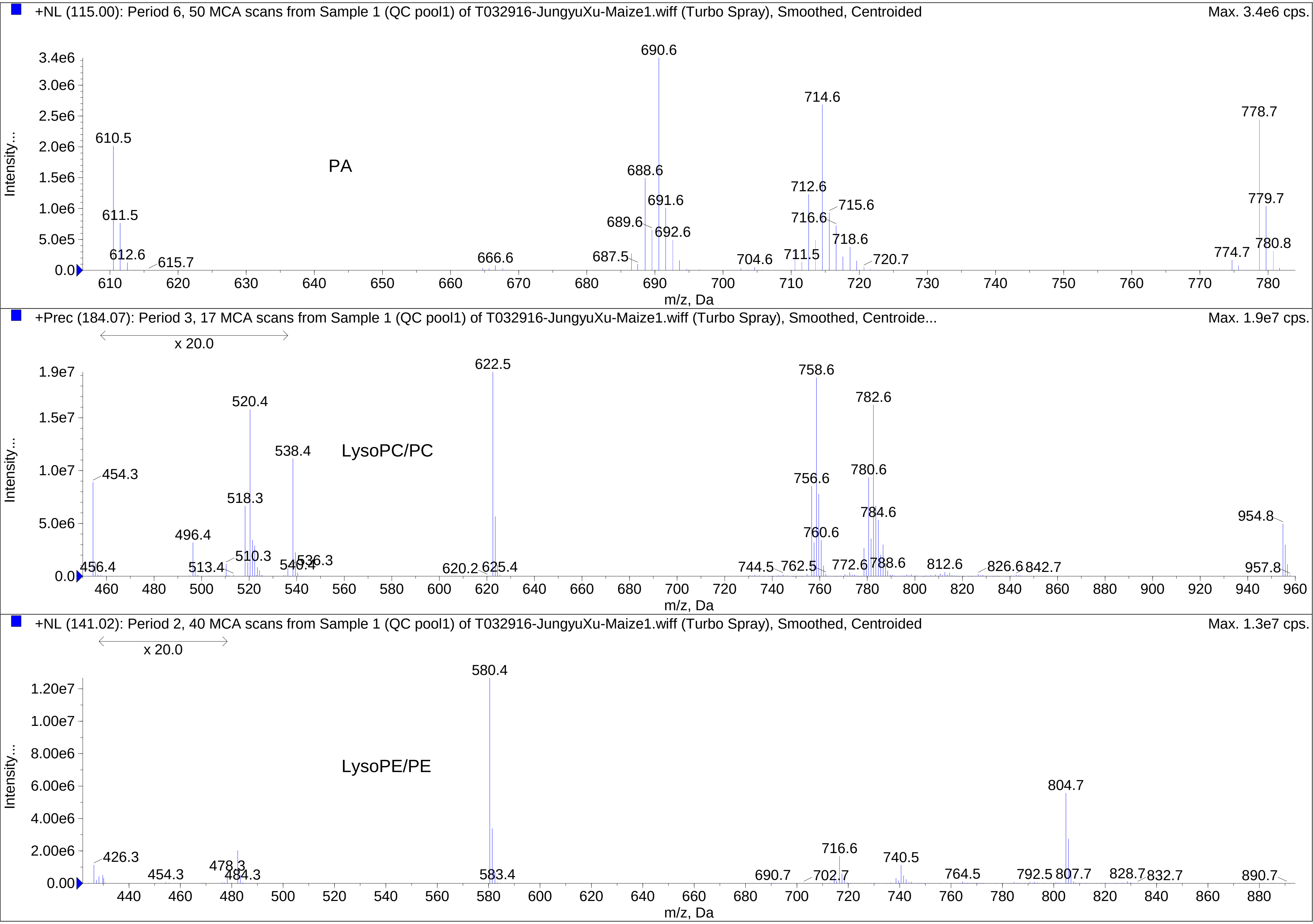
+NL (115.00): Period 6, 50 MCA scans from Sample 1 (QC pool1) of T032916-JungyuXu-Maize1.wiff (Turbo Spray), Smoothed, Centroided
Max. 3.4e6 cps.
PA
Intensity...
m/z, Da
+Prec (184.07): Period 3, 17 MCA scans from Sample 1 (QC pool1) of T032916-JungyuXu-Maize1.wiff (Turbo Spray), Smoothed, Centroide...
Max. 1.9e7 cps.
x 20.0
LysoPC/PC
Intensity...
m/z, Da
+NL (141.02): Period 2, 40 MCA scans from Sample 1 (QC pool1) of T032916-JungyuXu-Maize1.wiff (Turbo Spray), Smoothed, Centroided
Max. 1.3e7 cps.
x 20.0
LysoPE/PE
Intensity...
m/z, Da

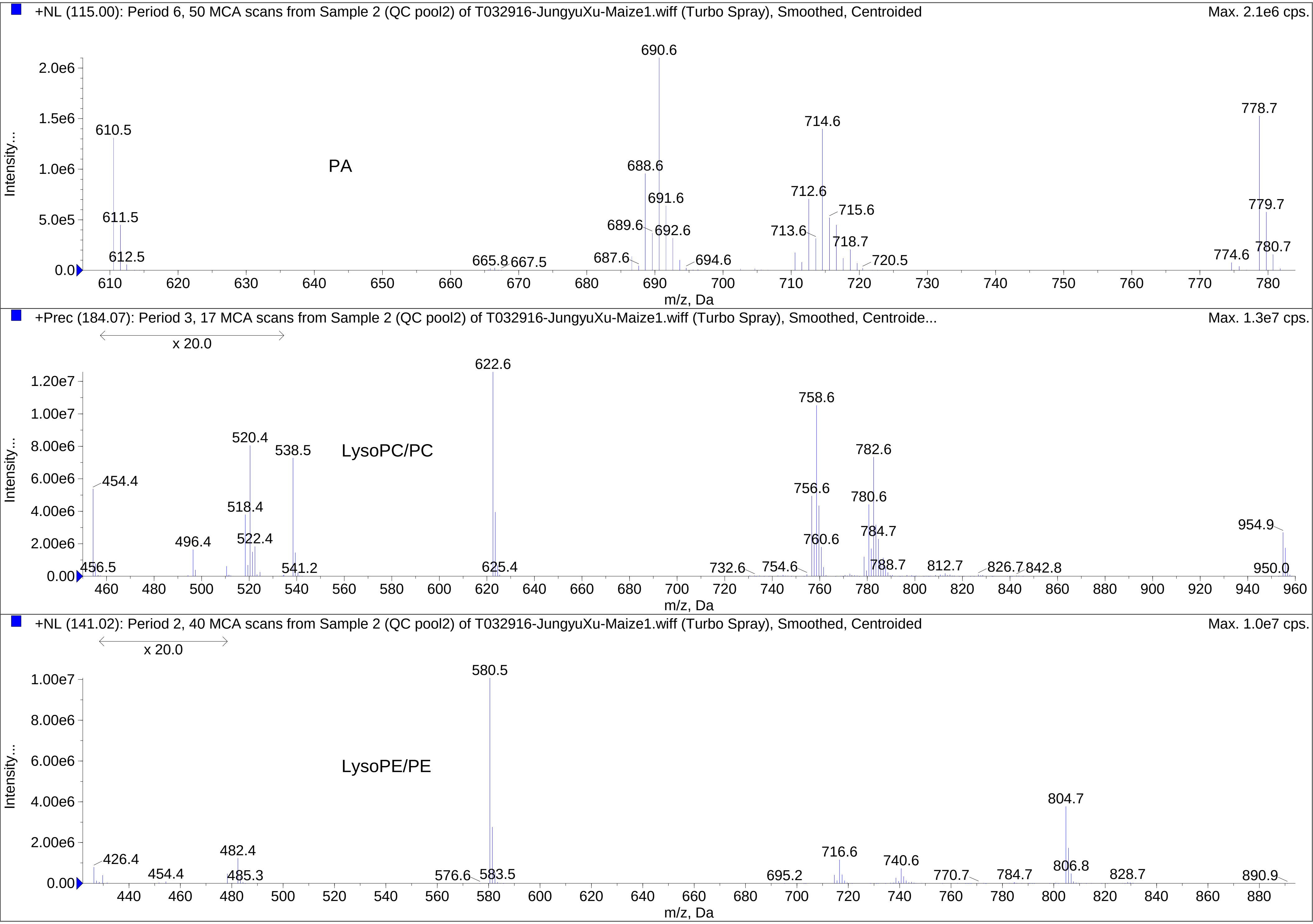
+NL (115.00): Period 6, 50 MCA scans from Sample 2 (QC pool2) of T032916-JungyuXu-Maize1.wiff (Turbo Spray), Smoothed, Centroided
Max. 2.1e6 cps.
PA
Intensity...
m/z, Da
610.5
611.5
612.5
665.8
667.5
687.6
688.6
689.6
690.6
691.6
692.6
694.6
712.6
713.6
714.6
715.6
718.7
720.5
774.6
778.7
779.7
780.7
+Prec (184.07): Period 3, 17 MCA scans from Sample 2 (QC pool2) of T032916-JungyuXu-Maize1.wiff (Turbo Spray), Smoothed, Centroide...
Max. 1.3e7 cps.
x 20.0
LysoPC/PC
Intensity...
m/z, Da
454.4
456.5
496.4
518.4
520.4
522.4
538.5
541.2
622.6
625.4
732.6
754.6
756.6
758.6
760.6
780.6
782.6
784.7
788.7
812.7
826.7
842.8
954.9
950.0
+NL (141.02): Period 2, 40 MCA scans from Sample 2 (QC pool2) of T032916-JungyuXu-Maize1.wiff (Turbo Spray), Smoothed, Centroided
Max. 1.0e7 cps.
x 20.0
LysoPE/PE
Intensity...
m/z, Da
426.4
454.4
482.4
485.3
576.6
580.5
583.5
695.2
716.6
740.6
770.7
784.7
804.7
806.8
828.7
890.9

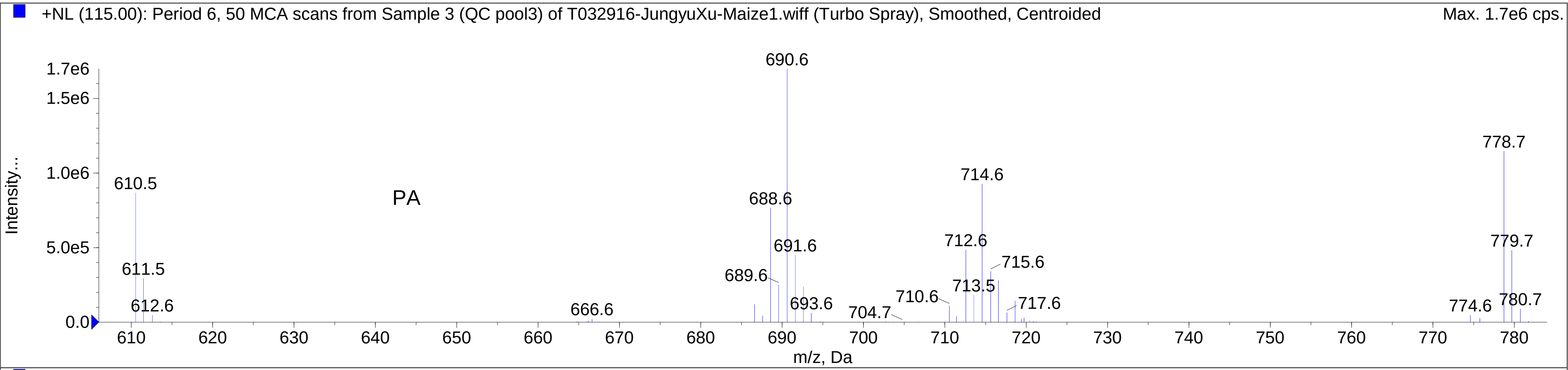
+NL (115.00): Period 6, 50 MCA scans from Sample 3 (QC pool3) of T032916-JungyuXu-Maize1.wiff (Turbo Spray), Smoothed, Centroided
Max. 1.7e6 cps.
PA
Intensity...
m/z, Da
690.6
778.7
714.6
610.5
688.6
691.6
712.6
779.7
715.6
611.5
689.6
713.5
710.6
717.6
612.6
666.6
693.6
704.7
774.6
780.7

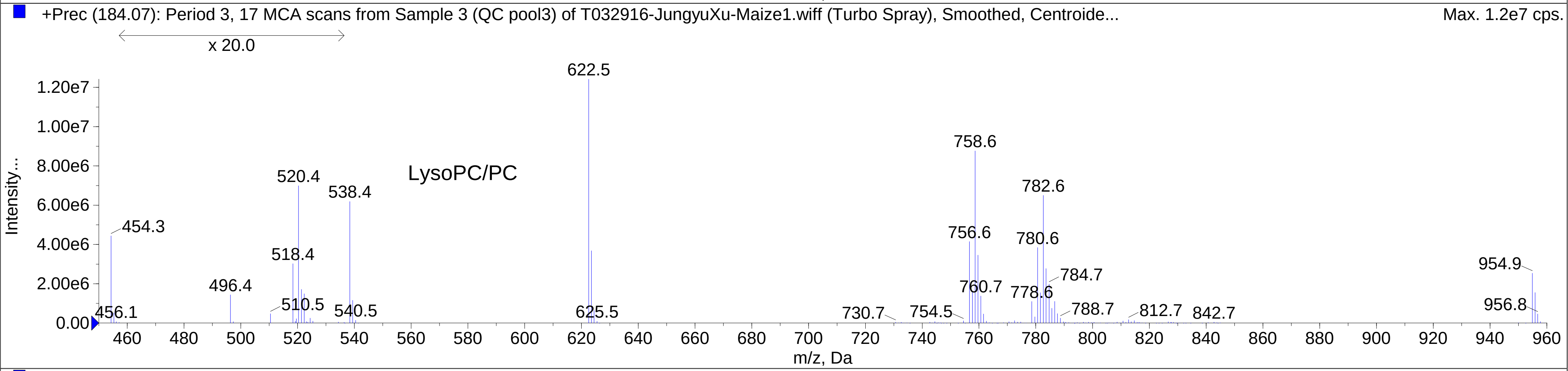
+Prec (184.07): Period 3, 17 MCA scans from Sample 3 (QC pool3) of T032916-JungyuXu-Maize1.wiff (Turbo Spray), Smoothed, Centroide...
Max. 1.2e7 cps.
x 20.0
LysoPC/PC
Intensity...
m/z, Da
622.5
758.6
520.4
538.4
782.6
454.3
756.6
780.6
518.4
784.7
496.4
760.7
954.9
778.6
510.5
540.5
456.1
625.5
730.7
754.5
788.7
812.7
842.7
956.8

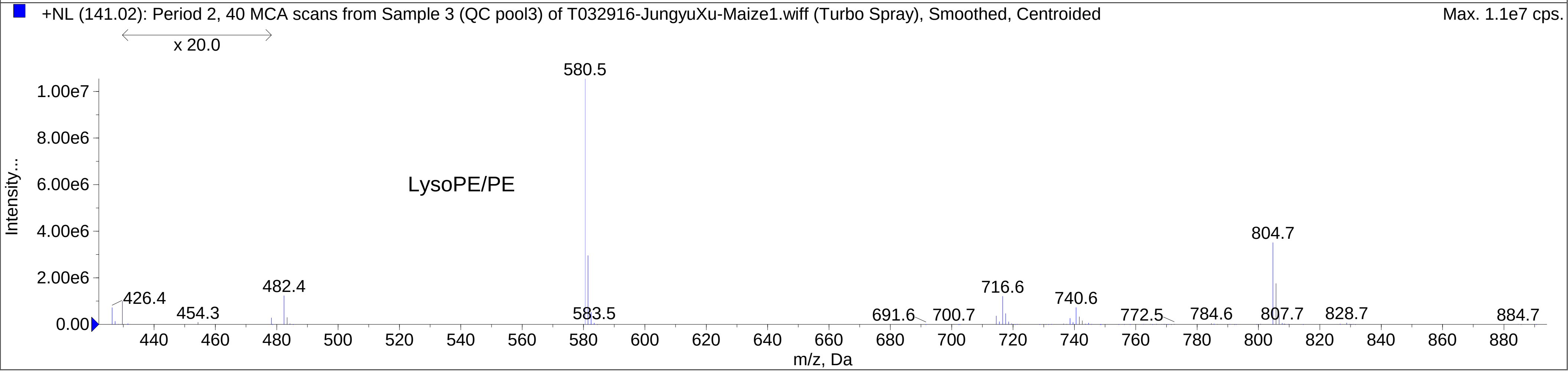
+NL (141.02): Period 2, 40 MCA scans from Sample 3 (QC pool3) of T032916-JungyuXu-Maize1.wiff (Turbo Spray), Smoothed, Centroided
Max. 1.1e7 cps.
x 20.0
LysoPE/PE
Intensity...
m/z, Da
580.5
804.7
426.4
482.4
716.6
740.6
454.3
583.5
691.6
700.7
772.5
784.6
807.7
828.7
884.7

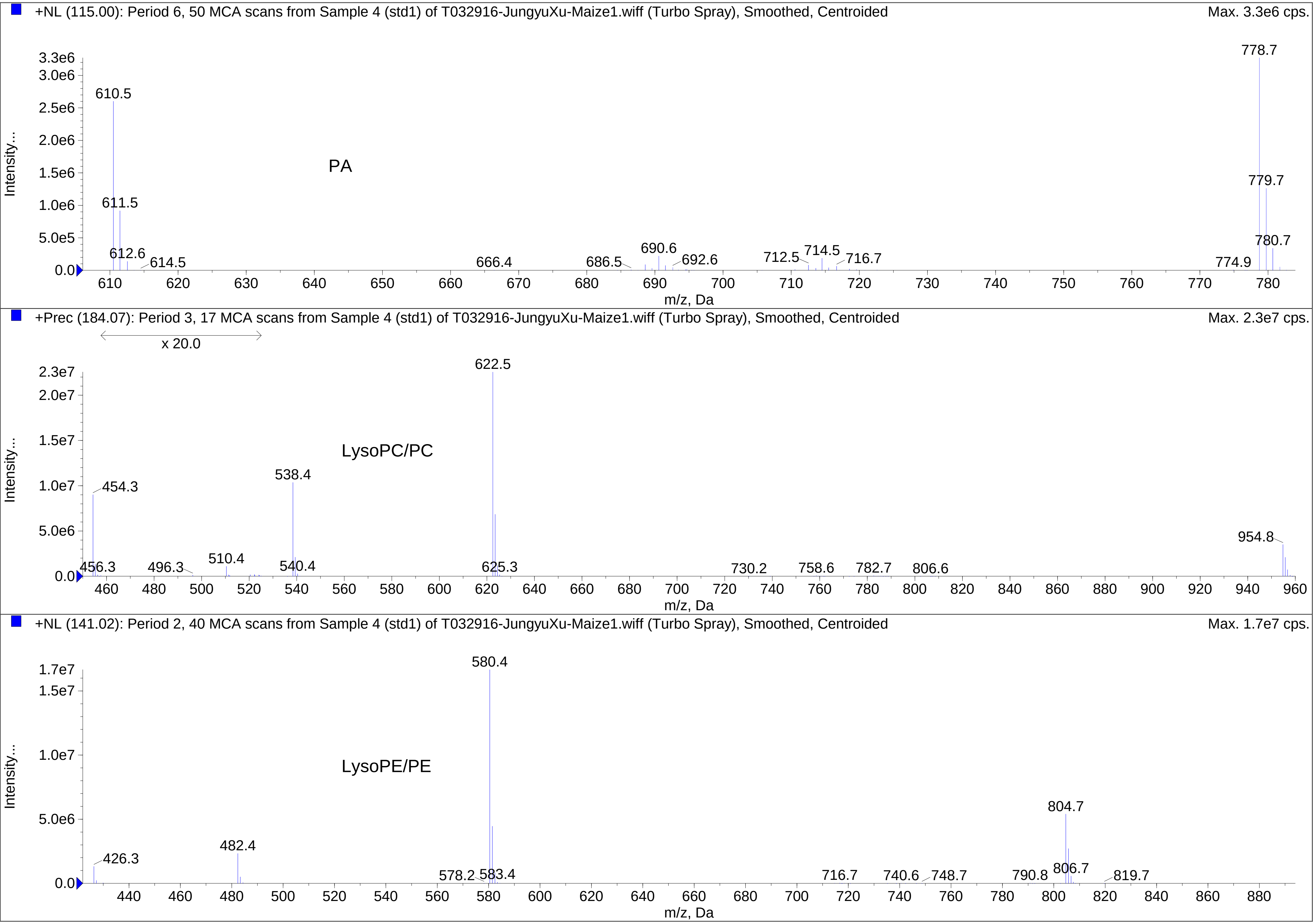
+NL (115.00): Period 6, 50 MCA scans from Sample 4 (std1) of T032916-JungyuXu-Maize1.wiff (Turbo Spray), Smoothed, Centroided
Max. 3.3e6 cps.
PA
Intensity...
m/z, Da
610.5
611.5
612.6
614.5
666.4
686.5
690.6
692.6
712.5
714.5
716.7
774.9
778.7
779.7
780.7
+Prec (184.07): Period 3, 17 MCA scans from Sample 4 (std1) of T032916-JungyuXu-Maize1.wiff (Turbo Spray), Smoothed, Centroided
Max. 2.3e7 cps.
x 20.0
LysoPC/PC
Intensity...
m/z, Da
454.3
456.3
496.3
510.4
538.4
540.4
622.5
625.3
730.2
758.6
782.7
806.6
954.8
+NL (141.02): Period 2, 40 MCA scans from Sample 4 (std1) of T032916-JungyuXu-Maize1.wiff (Turbo Spray), Smoothed, Centroided
Max. 1.7e7 cps.
LysoPE/PE
Intensity...
m/z, Da
426.3
482.4
578.2
580.4
583.4
716.7
740.6
748.7
790.8
804.7
806.7
819.7

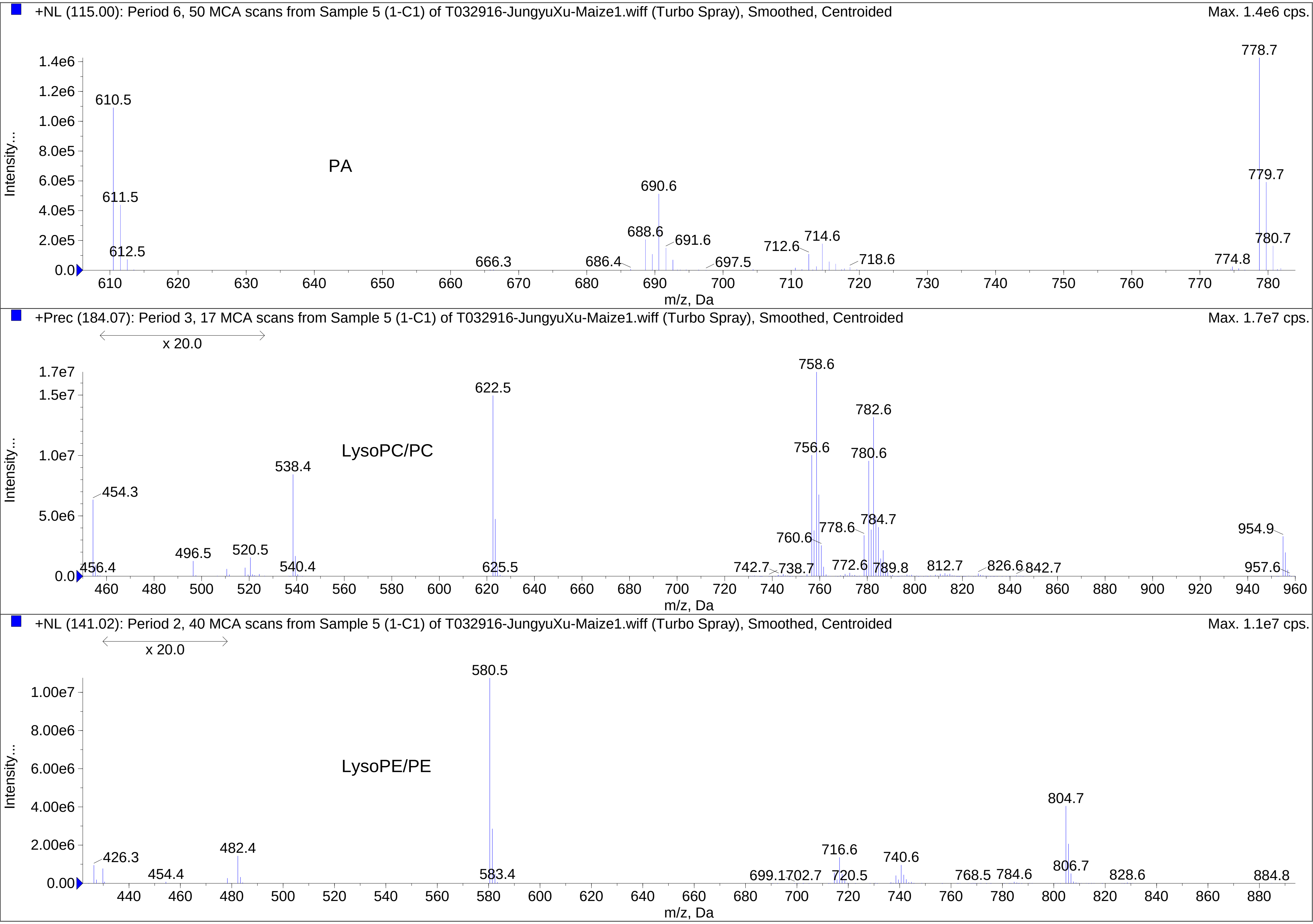

+NL (115.00): Period 6, 50 MCA scans from Sample 5 (1-C1) of T032916-JungyuXu-Maize1.wiff (Turbo Spray), Smoothed, Centroided
Max. 1.4e6 cps.
PA
778.7
610.5
690.6
779.7
611.5
688.6
691.6
714.6
712.6
780.7
612.5
666.3
686.4
697.5
718.6
774.8
Intensity...
m/z, Da
+Prec (184.07): Period 3, 17 MCA scans from Sample 5 (1-C1) of T032916-JungyuXu-Maize1.wiff (Turbo Spray), Smoothed, Centroided
Max. 1.7e7 cps.
x 20.0
LysoPC/PC
758.6
622.5
782.6
756.6
780.6
538.4
454.3
784.7
778.6
760.6
954.9
496.5
520.5
456.4
540.4
625.5
742.7
738.7
772.6
789.8
812.7
826.6
842.7
957.6
Intensity...
m/z, Da
+NL (141.02): Period 2, 40 MCA scans from Sample 5 (1-C1) of T032916-JungyuXu-Maize1.wiff (Turbo Spray), Smoothed, Centroided
Max. 1.1e7 cps.
x 20.0
LysoPE/PE
580.5
804.7
426.3
482.4
454.4
583.4
716.6
740.6
806.7
699.1
702.7
720.5
768.5
784.6
828.6
884.8
Intensity...
m/z, Da

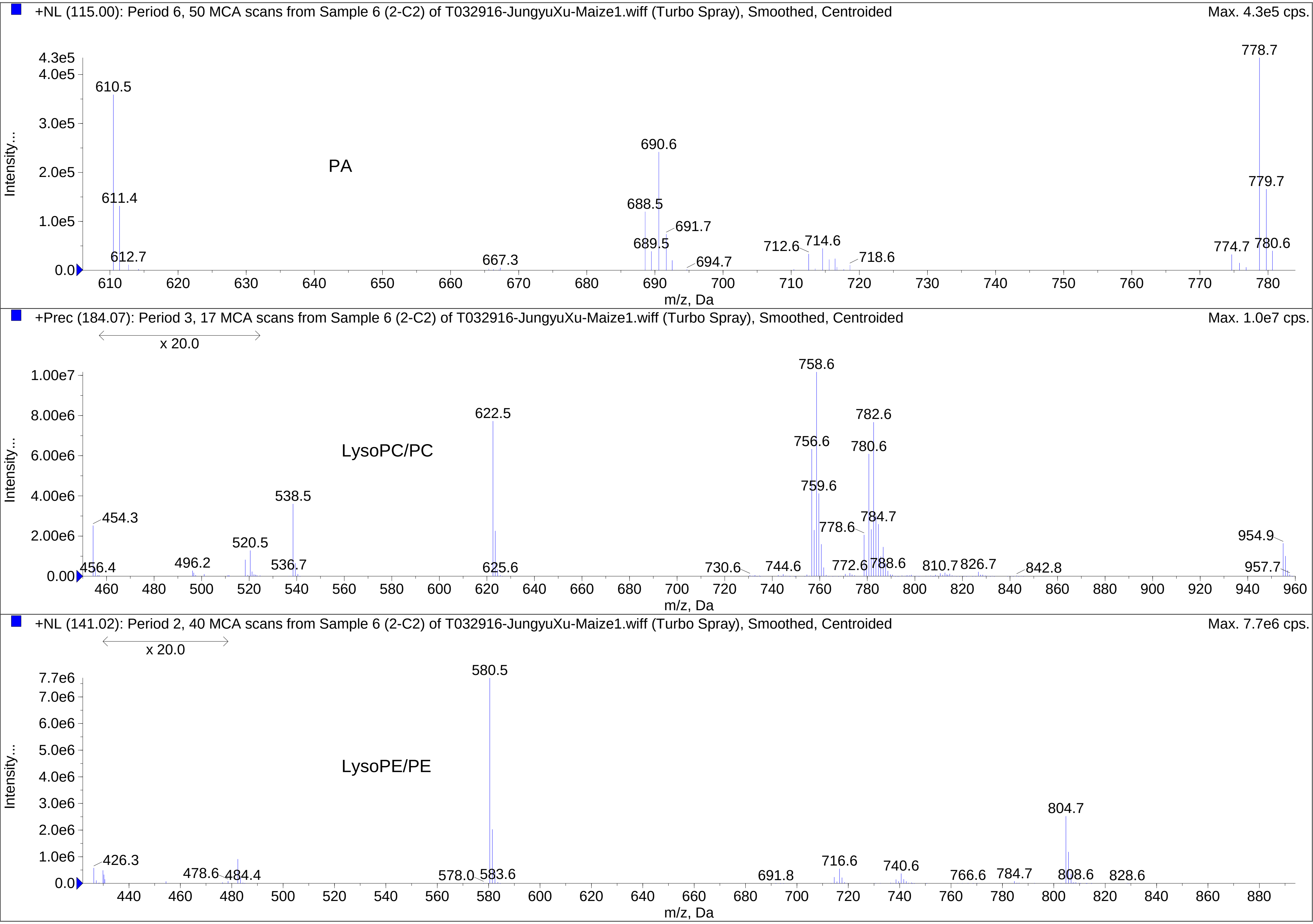

+NL (115.00): Period 6, 50 MCA scans from Sample 6 (2-C2) of T032916-JungyuXu-Maize1.wiff (Turbo Spray), Smoothed, Centroided
Max. 4.3e5 cps.
PA
Intensity...
m/z, Da
610.5
611.4
612.7
667.3
688.5
689.5
690.6
691.7
694.7
712.6
714.6
718.6
774.7
778.7
779.7
780.6
+Prec (184.07): Period 3, 17 MCA scans from Sample 6 (2-C2) of T032916-JungyuXu-Maize1.wiff (Turbo Spray), Smoothed, Centroided
Max. 1.0e7 cps.
x 20.0
LysoPC/PC
454.3
456.4
496.2
520.5
536.7
538.5
622.5
625.6
730.6
744.6
756.6
758.6
759.6
772.6
778.6
780.6
782.6
784.7
788.6
810.7
826.7
842.8
954.9
957.7
+NL (141.02): Period 2, 40 MCA scans from Sample 6 (2-C2) of T032916-JungyuXu-Maize1.wiff (Turbo Spray), Smoothed, Centroided
Max. 7.7e6 cps.
x 20.0
LysoPE/PE
426.3
478.6
484.4
578.0
580.5
583.6
691.8
716.6
740.6
766.6
784.7
804.7
808.6
828.6

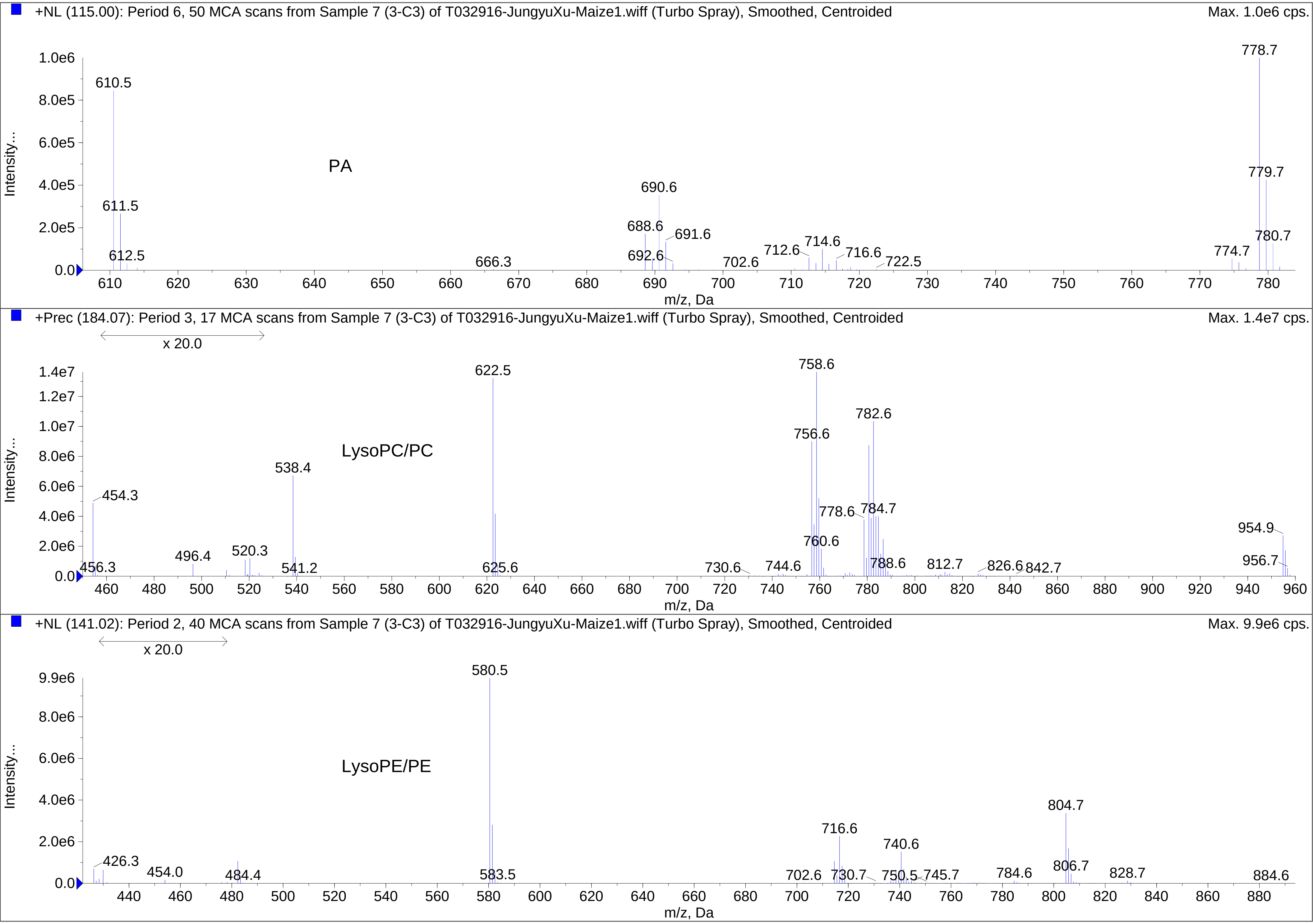
+NL (115.00): Period 6, 50 MCA scans from Sample 7 (3-C3) of T032916-JungyuXu-Maize1.wiff (Turbo Spray), Smoothed, Centroided
Max. 1.0e6 cps.
PA
Intensity...
m/z, Da
610.5
611.5
612.5
666.3
688.6
690.6
691.6
692.6
702.6
712.6
714.6
716.6
722.5
774.7
778.7
779.7
780.7
+Prec (184.07): Period 3, 17 MCA scans from Sample 7 (3-C3) of T032916-JungyuXu-Maize1.wiff (Turbo Spray), Smoothed, Centroided
Max. 1.4e7 cps.
x 20.0
LysoPC/PC
Intensity...
m/z, Da
454.3
456.3
496.4
520.3
538.4
541.2
622.5
625.6
730.6
744.6
756.6
758.6
760.6
778.6
782.6
784.7
788.6
812.7
826.6
842.7
954.9
956.7
+NL (141.02): Period 2, 40 MCA scans from Sample 7 (3-C3) of T032916-JungyuXu-Maize1.wiff (Turbo Spray), Smoothed, Centroided
Max. 9.9e6 cps.
x 20.0
LysoPE/PE
Intensity...
m/z, Da
426.3
454.0
484.4
580.5
583.5
702.6
716.6
730.7
740.6
750.5
745.7
784.6
804.7
806.7
828.7
884.6

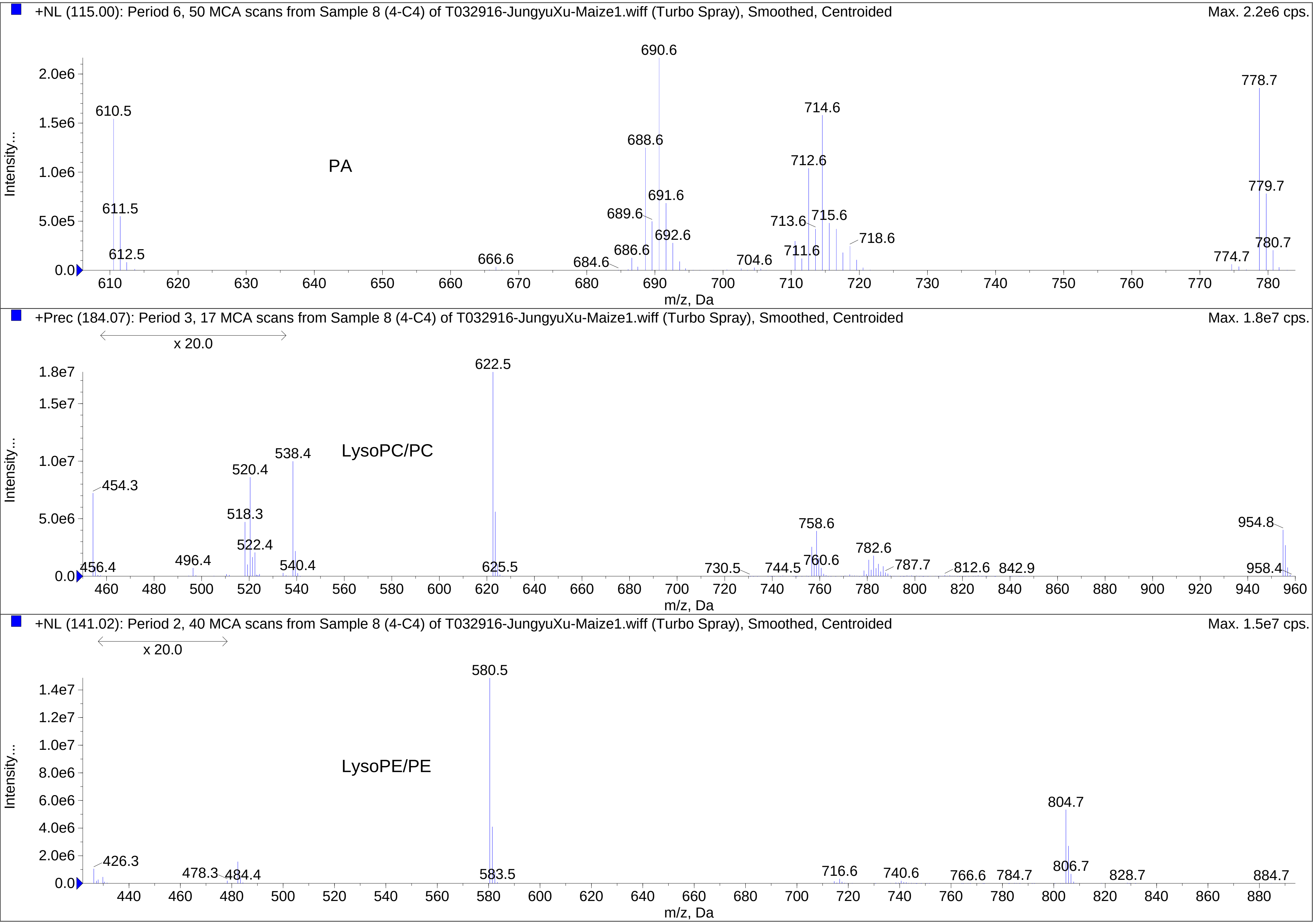

+NL (115.00): Period 6, 50 MCA scans from Sample 8 (4-C4) of T032916-JungyuXu-Maize1.wiff (Turbo Spray), Smoothed, Centroided
Max. 2.2e6 cps.
PA
Intensity...
m/z, Da
690.6
778.7
610.5
714.6
688.6
712.6
691.6
779.7
611.5
689.6
715.6
713.6
692.6
718.6
612.5
666.6
684.6
686.6
704.6
711.6
774.7
780.7
+Prec (184.07): Period 3, 17 MCA scans from Sample 8 (4-C4) of T032916-JungyuXu-Maize1.wiff (Turbo Spray), Smoothed, Centroided
Max. 1.8e7 cps.
x 20.0
LysoPC/PC
622.5
538.4
520.4
454.3
518.3
522.4
496.4
456.4
540.4
625.5
730.5
744.5
758.6
760.6
782.6
787.7
812.6
842.9
954.8
958.4
+NL (141.02): Period 2, 40 MCA scans from Sample 8 (4-C4) of T032916-JungyuXu-Maize1.wiff (Turbo Spray), Smoothed, Centroided
Max. 1.5e7 cps.
x 20.0
LysoPE/PE
580.5
804.7
426.3
478.3
484.4
583.5
716.6
740.6
766.6
784.7
806.7
828.7
884.7

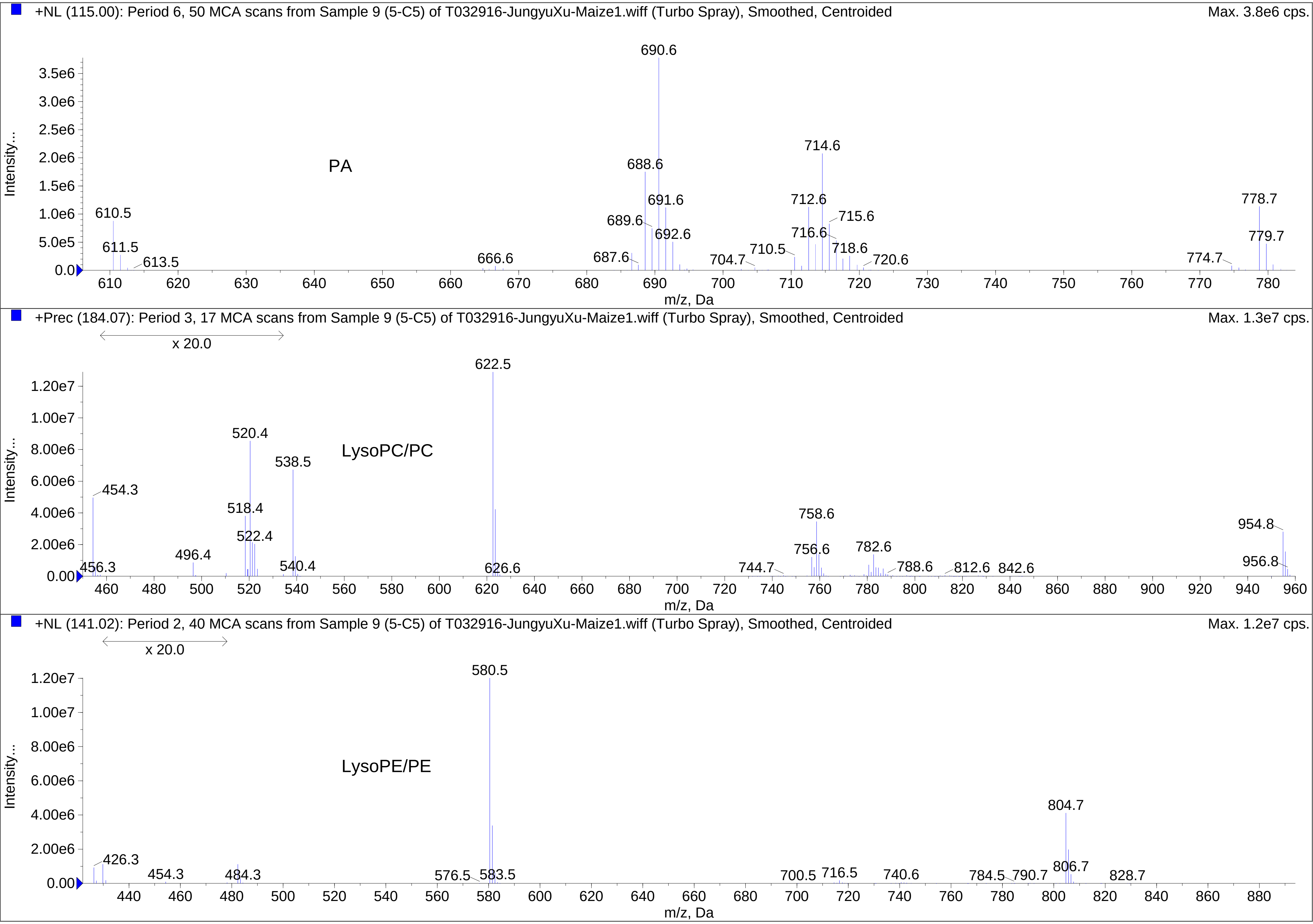
+NL (115.00): Period 6, 50 MCA scans from Sample 9 (5-C5) of T032916-JungyuXu-Maize1.wiff (Turbo Spray), Smoothed, Centroided
Max. 3.8e6 cps.
PA
690.6
714.6
688.6
691.6
712.6
778.7
610.5
689.6
715.6
716.6
692.6
779.7
611.5
718.6
710.5
613.5
666.6
687.6
704.7
720.6
774.7
Intensity...
m/z, Da
+Prec (184.07): Period 3, 17 MCA scans from Sample 9 (5-C5) of T032916-JungyuXu-Maize1.wiff (Turbo Spray), Smoothed, Centroided
Max. 1.3e7 cps.
x 20.0
622.5
520.4
LysoPC/PC
538.5
454.3
518.4
758.6
954.8
522.4
496.4
782.6
756.6
956.8
456.3
540.4
626.6
744.7
788.6
812.6
842.6
Intensity...
m/z, Da
+NL (141.02): Period 2, 40 MCA scans from Sample 9 (5-C5) of T032916-JungyuXu-Maize1.wiff (Turbo Spray), Smoothed, Centroided
Max. 1.2e7 cps.
x 20.0
580.5
LysoPE/PE
804.7
426.3
454.3
484.3
576.5
583.5
700.5
716.5
740.6
784.5
790.7
806.7
828.7
Intensity...
m/z, Da

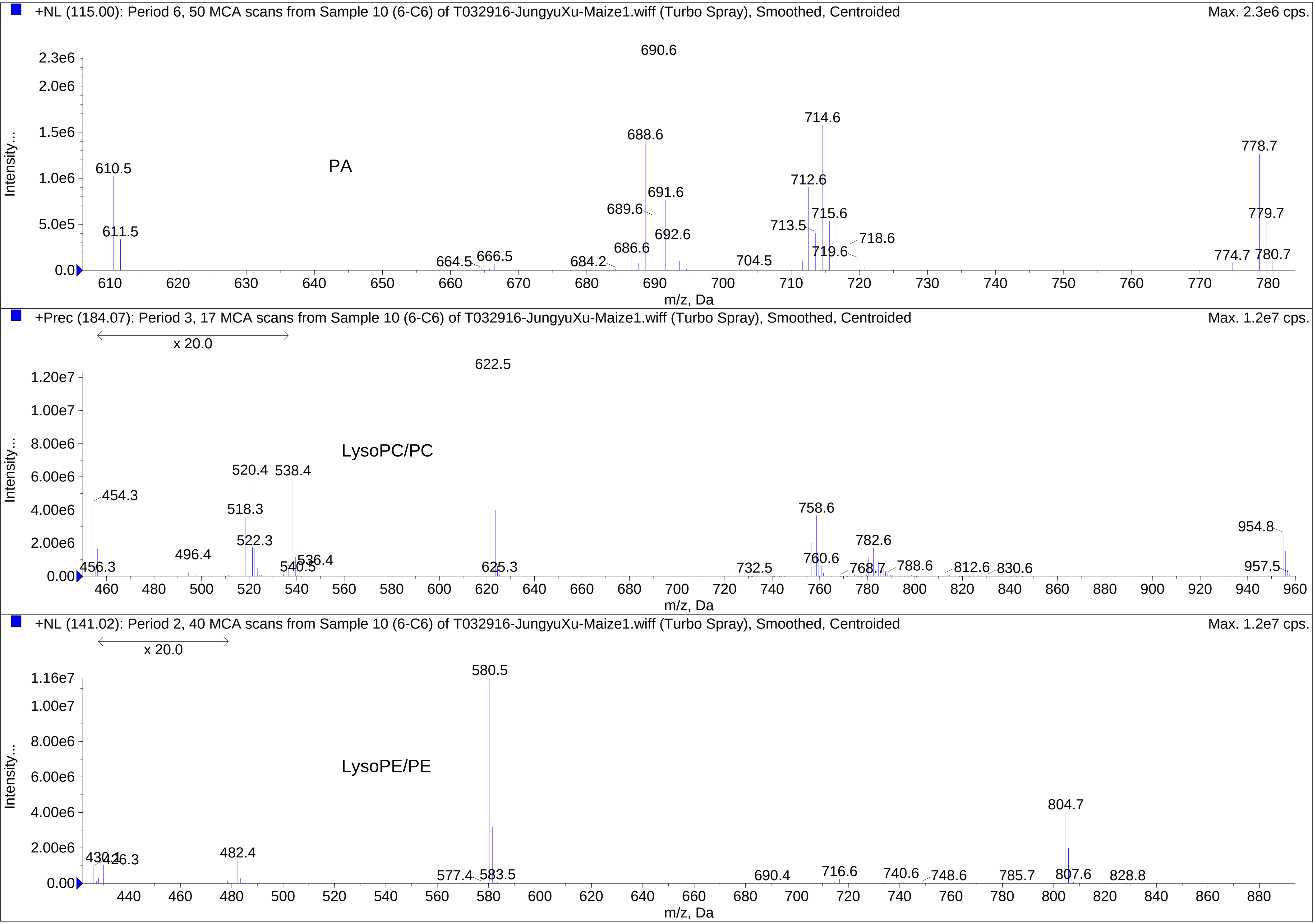
+NL (115.00): Period 6, 50 MCA scans from Sample 10 (6-C6) of T032916-JungyuXu-Maize1.wiff (Turbo Spray), Smoothed, Centroided
Max. 2.3e6 cps.
PA
Intensity...
m/z, Da
690.6
688.6
714.6
778.7
610.5
712.6
691.6
689.6
715.6
779.7
713.5
611.5
692.6
718.6
686.6
666.5
664.5
684.2
704.5
719.6
774.7
780.7
+Prec (184.07): Period 3, 17 MCA scans from Sample 10 (6-C6) of T032916-JungyuXu-Maize1.wiff (Turbo Spray), Smoothed, Centroided
Max. 1.2e7 cps.
x 20.0
LysoPC/PC
622.5
520.4
538.4
454.3
518.3
758.6
782.6
954.8
522.3
496.4
536.4
456.3
540.5
625.3
732.5
760.6
768.7
788.6
812.6
830.6
957.5
+NL (141.02): Period 2, 40 MCA scans from Sample 10 (6-C6) of T032916-JungyuXu-Maize1.wiff (Turbo Spray), Smoothed, Centroided
Max. 1.2e7 cps.
x 20.0
LysoPE/PE
580.5
804.7
482.4
430.1
426.3
577.4
583.5
690.4
716.6
740.6
748.6
785.7
807.6
828.8

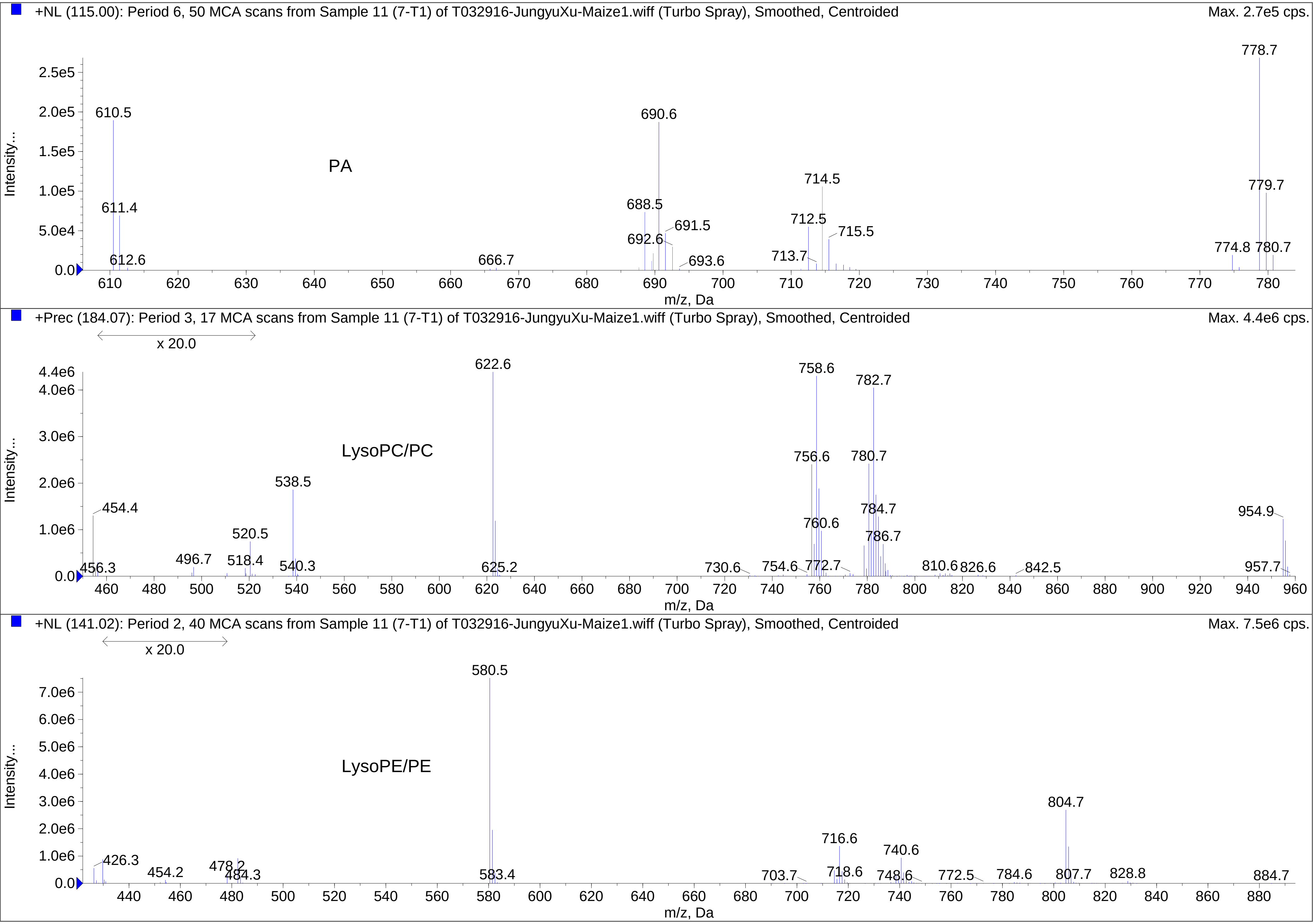
+NL (115.00): Period 6, 50 MCA scans from Sample 11 (7-T1) of T032916-JungyuXu-Maize1.wiff (Turbo Spray), Smoothed, Centroided
Max. 2.7e5 cps.
PA
Intensity...
m/z, Da
610.5
611.4
612.6
666.7
688.5
690.6
691.5
692.6
693.6
712.5
713.7
714.5
715.5
774.8
778.7
779.7
780.7
+Prec (184.07): Period 3, 17 MCA scans from Sample 11 (7-T1) of T032916-JungyuXu-Maize1.wiff (Turbo Spray), Smoothed, Centroided
Max. 4.4e6 cps.
x 20.0
LysoPC/PC
454.4
456.3
496.7
518.4
520.5
538.5
540.3
622.6
625.2
730.6
754.6
756.6
758.6
760.6
772.7
780.7
782.7
784.7
786.7
810.6
826.6
842.5
954.9
957.7
+NL (141.02): Period 2, 40 MCA scans from Sample 11 (7-T1) of T032916-JungyuXu-Maize1.wiff (Turbo Spray), Smoothed, Centroided
Max. 7.5e6 cps.
x 20.0
LysoPE/PE
426.3
454.2
478.2
484.3
580.5
583.4
703.7
716.6
718.6
740.6
748.6
772.5
784.6
804.7
807.7
828.8
884.7

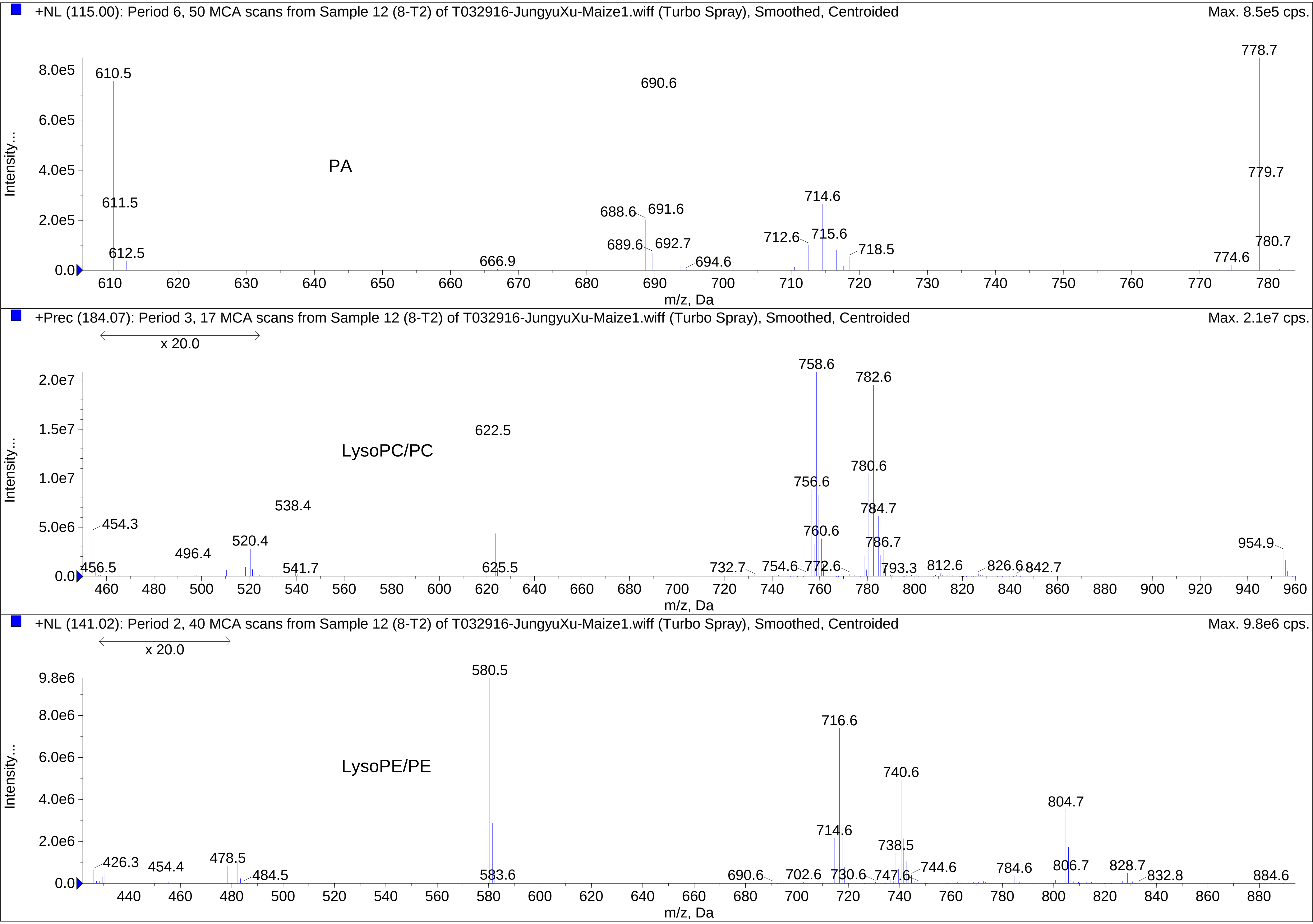
+NL (115.00): Period 6, 50 MCA scans from Sample 12 (8-T2) of T032916-JungyuXu-Maize1.wiff (Turbo Spray), Smoothed, Centroided
Max. 8.5e5 cps.
PA
Intensity...
m/z, Da
610.5
611.5
612.5
666.9
688.6
689.6
690.6
691.6
692.7
694.6
712.6
714.6
715.6
718.5
774.6
778.7
779.7
780.7
+Prec (184.07): Period 3, 17 MCA scans from Sample 12 (8-T2) of T032916-JungyuXu-Maize1.wiff (Turbo Spray), Smoothed, Centroided
Max. 2.1e7 cps.
x 20.0
LysoPC/PC
Intensity...
m/z, Da
454.3
456.5
496.4
520.4
538.4
541.7
622.5
625.5
732.7
754.6
756.6
758.6
760.6
772.6
780.6
782.6
784.7
786.7
793.3
812.6
826.6
842.7
954.9
+NL (141.02): Period 2, 40 MCA scans from Sample 12 (8-T2) of T032916-JungyuXu-Maize1.wiff (Turbo Spray), Smoothed, Centroided
Max. 9.8e6 cps.
x 20.0
LysoPE/PE
Intensity...
m/z, Da
426.3
454.4
478.5
484.5
580.5
583.6
690.6
702.6
714.6
716.6
730.6
738.5
740.6
744.6
747.6
784.6
804.7
806.7
828.7
832.8
884.6

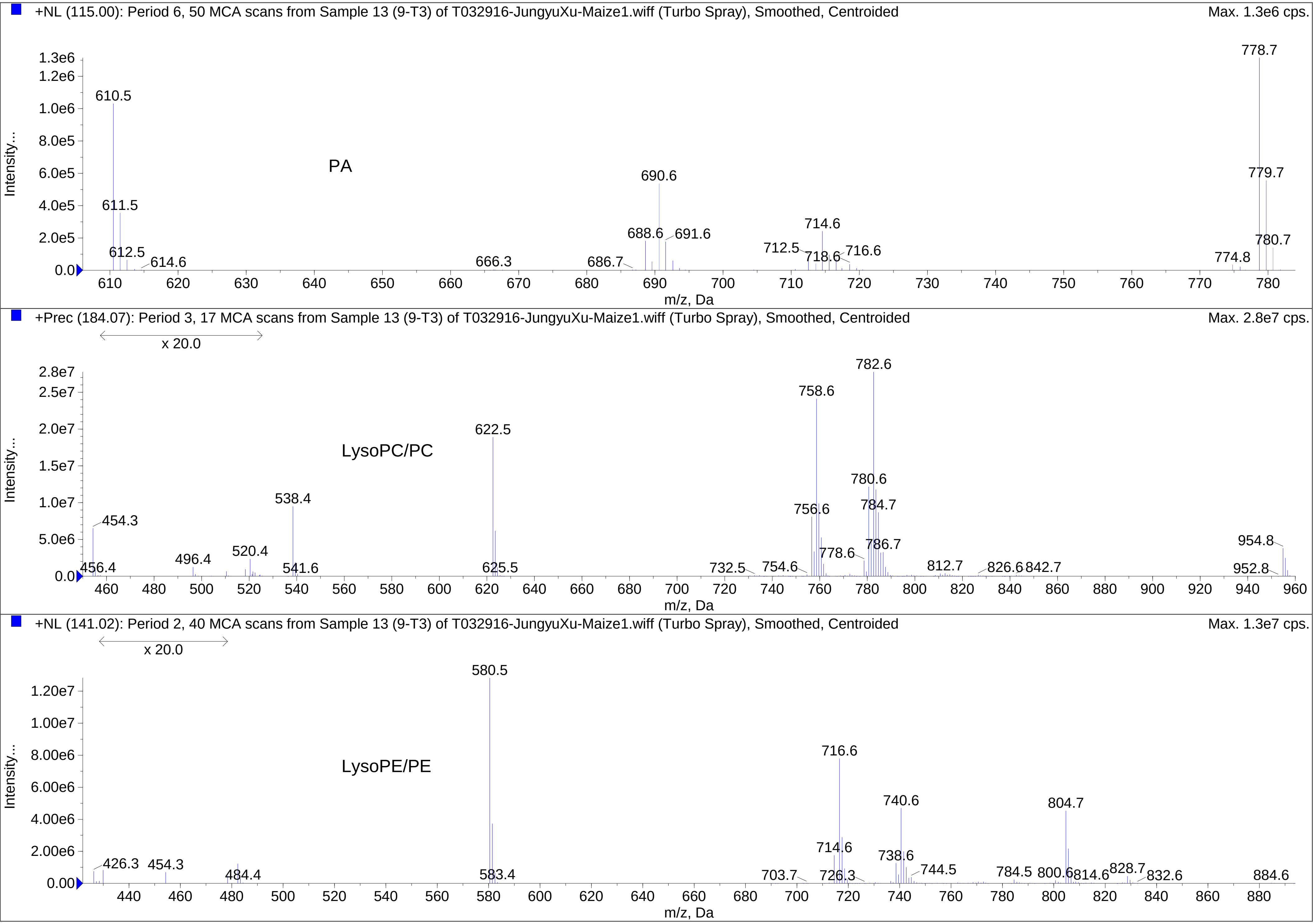
+NL (115.00): Period 6, 50 MCA scans from Sample 13 (9-T3) of T032916-JungyuXu-Maize1.wiff (Turbo Spray), Smoothed, Centroided
Max. 1.3e6 cps.
PA
Intensity...
m/z, Da
+Prec (184.07): Period 3, 17 MCA scans from Sample 13 (9-T3) of T032916-JungyuXu-Maize1.wiff (Turbo Spray), Smoothed, Centroided
Max. 2.8e7 cps.
x 20.0
LysoPC/PC
Intensity...
m/z, Da
+NL (141.02): Period 2, 40 MCA scans from Sample 13 (9-T3) of T032916-JungyuXu-Maize1.wiff (Turbo Spray), Smoothed, Centroided
Max. 1.3e7 cps.
x 20.0
LysoPE/PE
Intensity...
m/z, Da

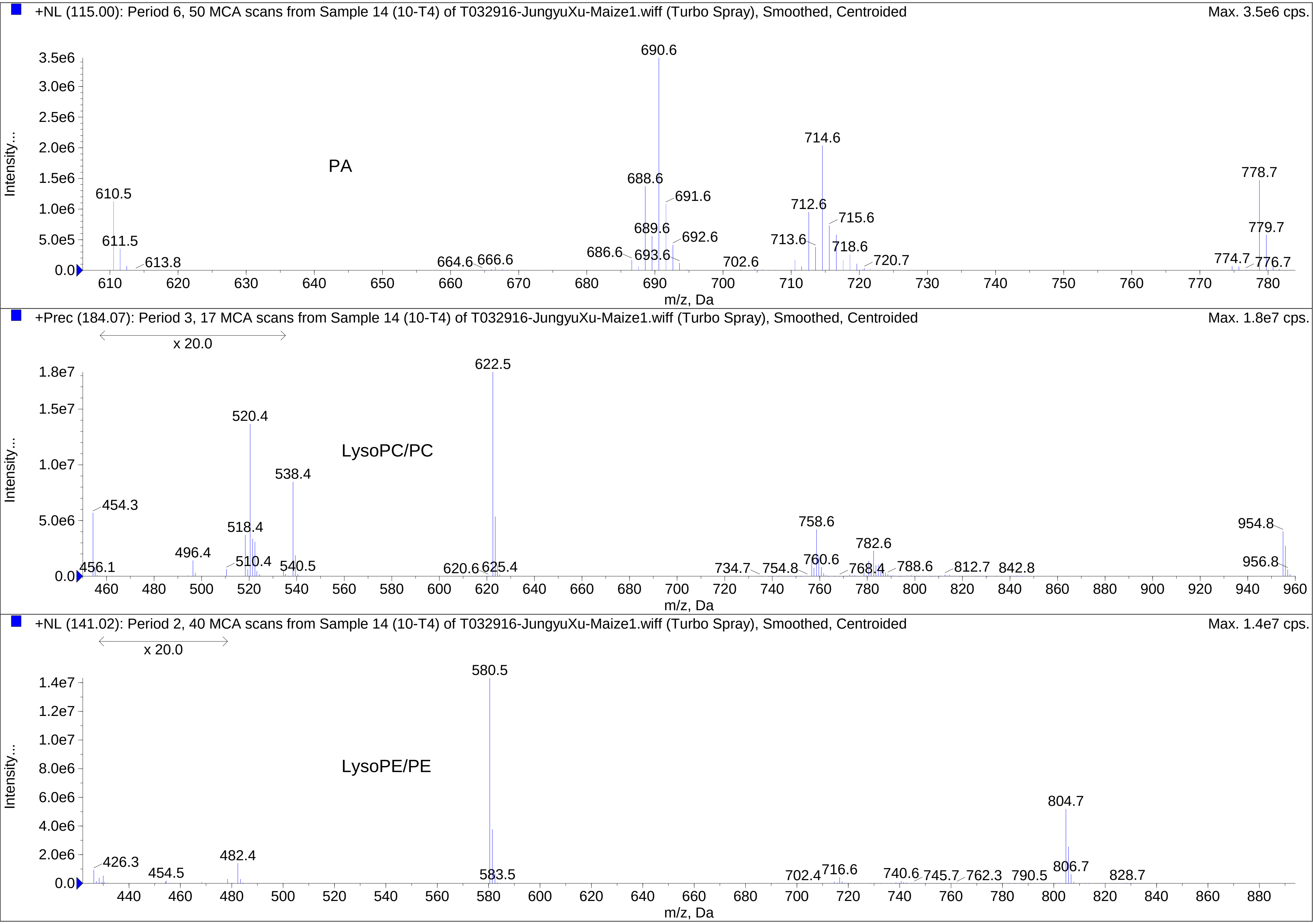
+NL (115.00): Period 6, 50 MCA scans from Sample 14 (10-T4) of T032916-JungyuXu-Maize1.wiff (Turbo Spray), Smoothed, Centroided
Max. 3.5e6 cps.
PA
690.6
714.6
778.7
688.6
691.6
610.5
712.6
715.6
689.6
692.6
779.7
611.5
713.6
718.6
686.6
693.6
774.7
776.7
613.8
664.6
666.6
702.6
720.7
Intensity...
m/z, Da
+Prec (184.07): Period 3, 17 MCA scans from Sample 14 (10-T4) of T032916-JungyuXu-Maize1.wiff (Turbo Spray), Smoothed, Centroided
Max. 1.8e7 cps.
x 20.0
LysoPC/PC
622.5
520.4
538.4
454.3
518.4
496.4
510.4
540.5
456.1
620.6
625.4
734.7
754.8
758.6
760.6
768.4
782.6
788.6
812.7
842.8
954.8
956.8
Intensity...
m/z, Da
+NL (141.02): Period 2, 40 MCA scans from Sample 14 (10-T4) of T032916-JungyuXu-Maize1.wiff (Turbo Spray), Smoothed, Centroided
Max. 1.4e7 cps.
x 20.0
LysoPE/PE
580.5
804.7
426.3
454.5
482.4
583.5
702.4
716.6
740.6
745.7
762.3
790.5
806.7
828.7
Intensity...
m/z, Da

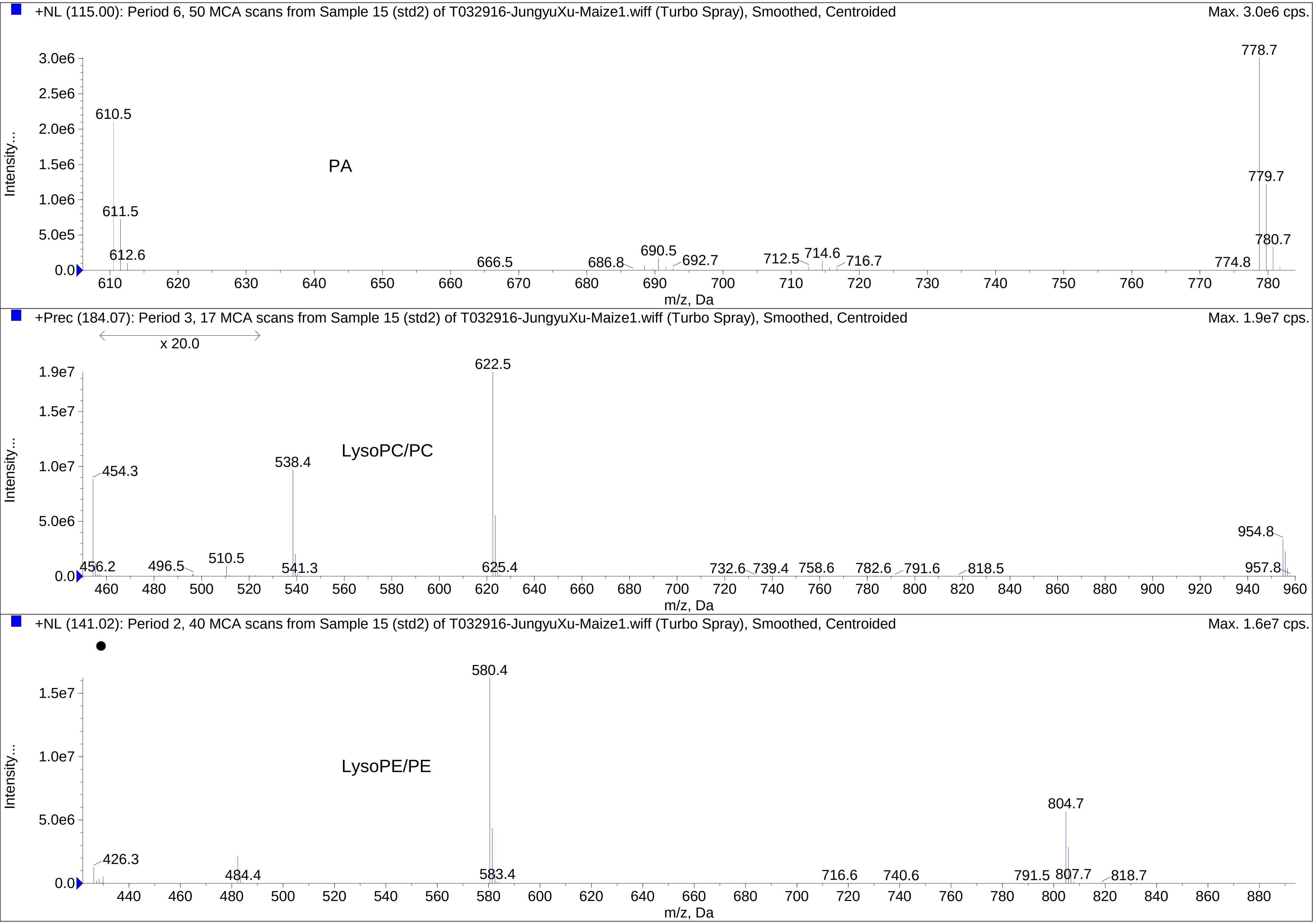

+NL (115.00): Period 6, 50 MCA scans from Sample 15 (std2) of T032916-JungyuXu-Maize1.wiff (Turbo Spray), Smoothed, Centroided
Max. 3.0e6 cps.
PA
610.5
611.5
612.6
666.5
686.8
690.5
692.7
712.5
714.6
716.7
774.8
778.7
779.7
780.7
Intensity...
m/z, Da
+Prec (184.07): Period 3, 17 MCA scans from Sample 15 (std2) of T032916-JungyuXu-Maize1.wiff (Turbo Spray), Smoothed, Centroided
Max. 1.9e7 cps.
x 20.0
LysoPC/PC
454.3
456.2
496.5
510.5
538.4
541.3
622.5
625.4
732.6
739.4
758.6
782.6
791.6
818.5
954.8
957.8
Intensity...
m/z, Da
+NL (141.02): Period 2, 40 MCA scans from Sample 15 (std2) of T032916-JungyuXu-Maize1.wiff (Turbo Spray), Smoothed, Centroided
Max. 1.6e7 cps.
LysoPE/PE
426.3
484.4
580.4
583.4
716.6
740.6
791.5
804.7
807.7
818.7
Intensity...
m/z, Da

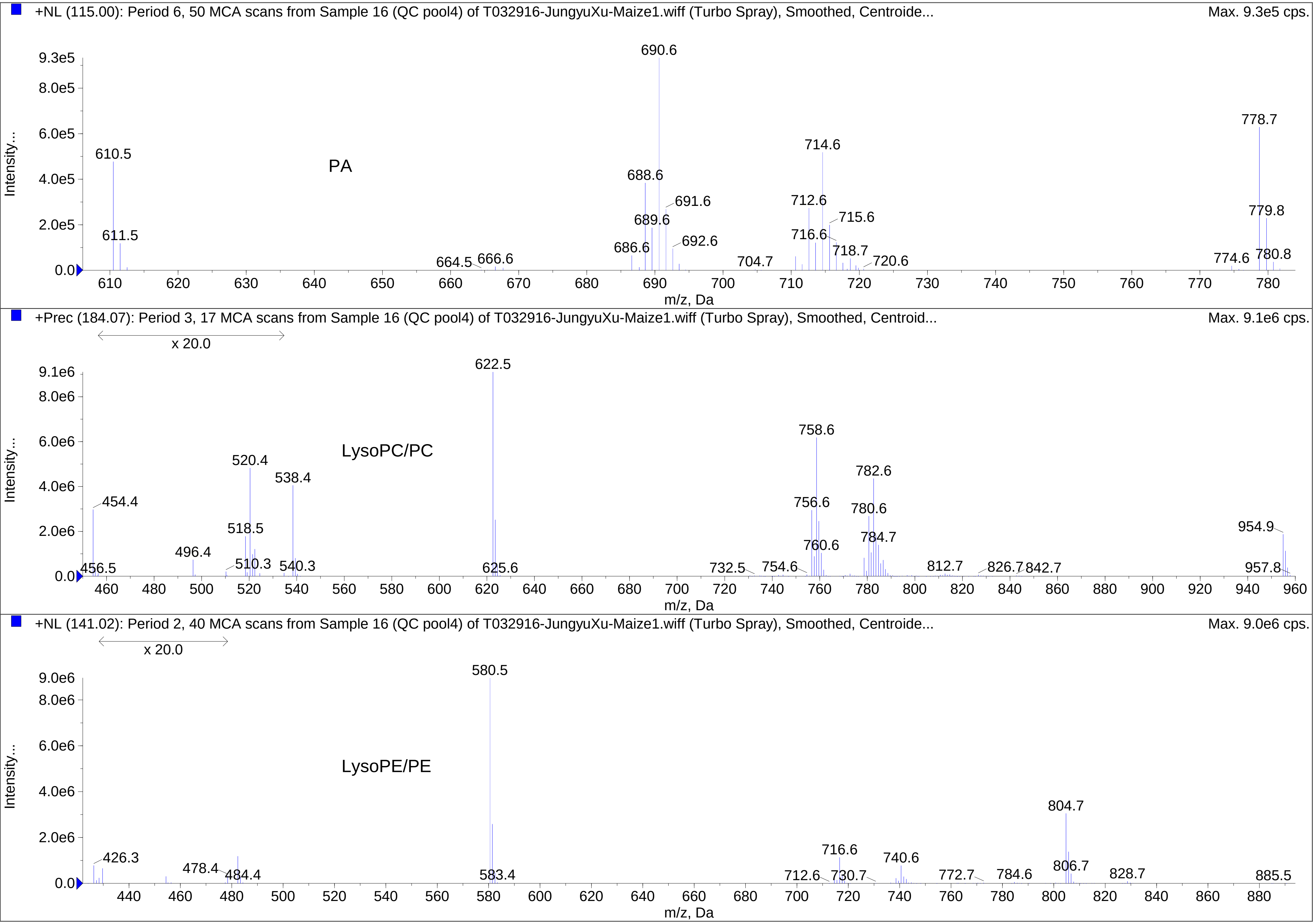

+NL (115.00): Period 6, 50 MCA scans from Sample 16 (QC pool4) of T032916-JungyuXu-Maize1.wiff (Turbo Spray), Smoothed, Centroide...
Max. 9.3e5 cps.
PA
Intensity...
m/z, Da
690.6
778.7
714.6
610.5
688.6
691.6
712.6
689.6
715.6
779.8
611.5
716.6
692.6
686.6
718.7
780.8
774.6
664.5
666.6
704.7
720.6
+Prec (184.07): Period 3, 17 MCA scans from Sample 16 (QC pool4) of T032916-JungyuXu-Maize1.wiff (Turbo Spray), Smoothed, Centroid...
Max. 9.1e6 cps.
x 20.0
LysoPC/PC
622.5
758.6
782.6
520.4
538.4
454.4
756.6
780.6
518.5
954.9
784.7
760.6
496.4
456.5
510.3
540.3
625.6
732.5
754.6
812.7
826.7
842.7
957.8
+NL (141.02): Period 2, 40 MCA scans from Sample 16 (QC pool4) of T032916-JungyuXu-Maize1.wiff (Turbo Spray), Smoothed, Centroide...
Max. 9.0e6 cps.
x 20.0
LysoPE/PE
580.5
804.7
716.6
740.6
426.3
806.7
478.4
484.4
583.4
712.6
730.7
772.7
784.6
828.7
885.5

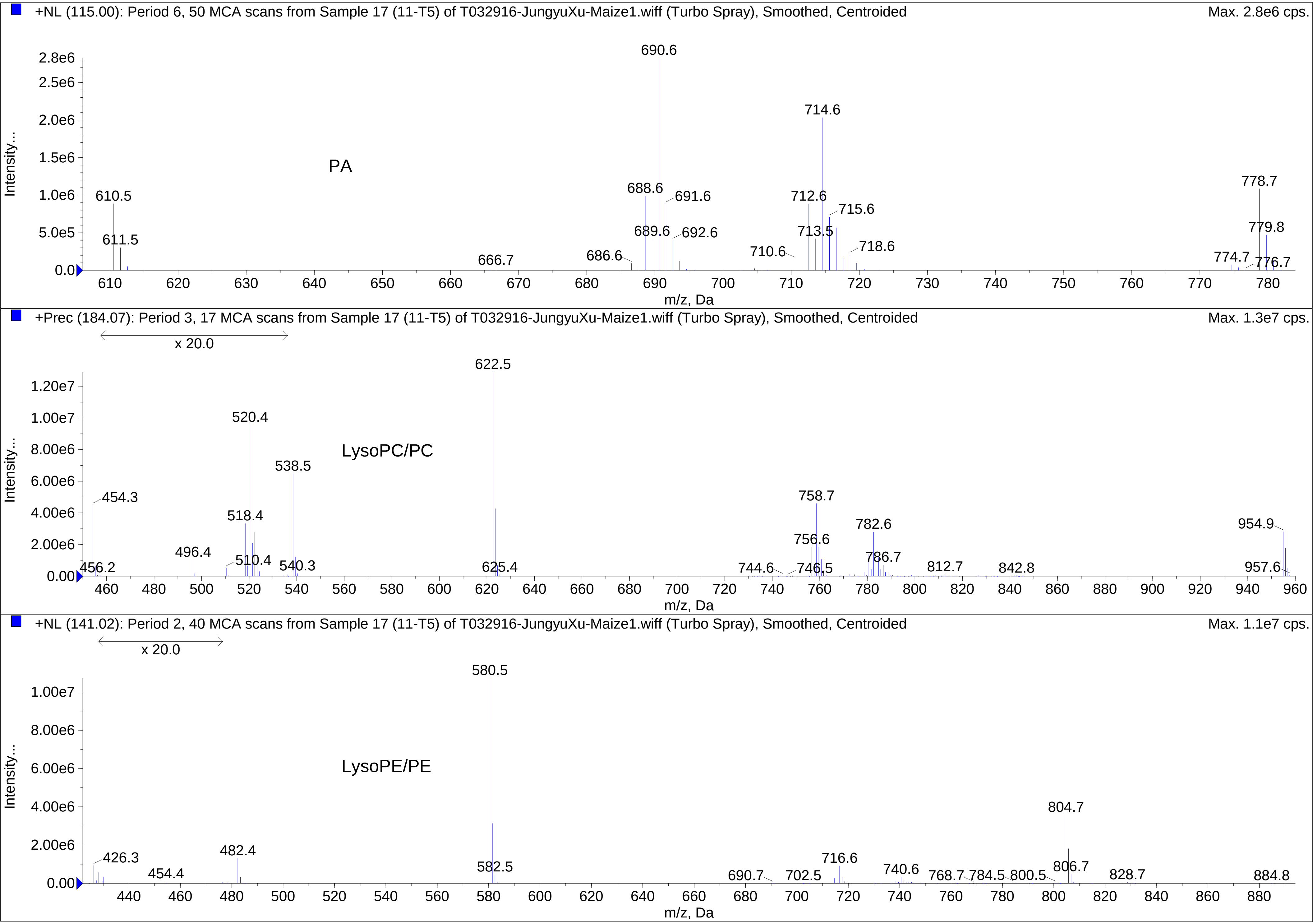
+NL (115.00): Period 6, 50 MCA scans from Sample 17 (11-T5) of T032916-JungyuXu-Maize1.wiff (Turbo Spray), Smoothed, Centroided
Max. 2.8e6 cps.
PA
Intensity...
m/z, Da
+Prec (184.07): Period 3, 17 MCA scans from Sample 17 (11-T5) of T032916-JungyuXu-Maize1.wiff (Turbo Spray), Smoothed, Centroided
Max. 1.3e7 cps.
x 20.0
LysoPC/PC
Intensity...
m/z, Da
+NL (141.02): Period 2, 40 MCA scans from Sample 17 (11-T5) of T032916-JungyuXu-Maize1.wiff (Turbo Spray), Smoothed, Centroided
Max. 1.1e7 cps.
x 20.0
LysoPE/PE
Intensity...
m/z, Da

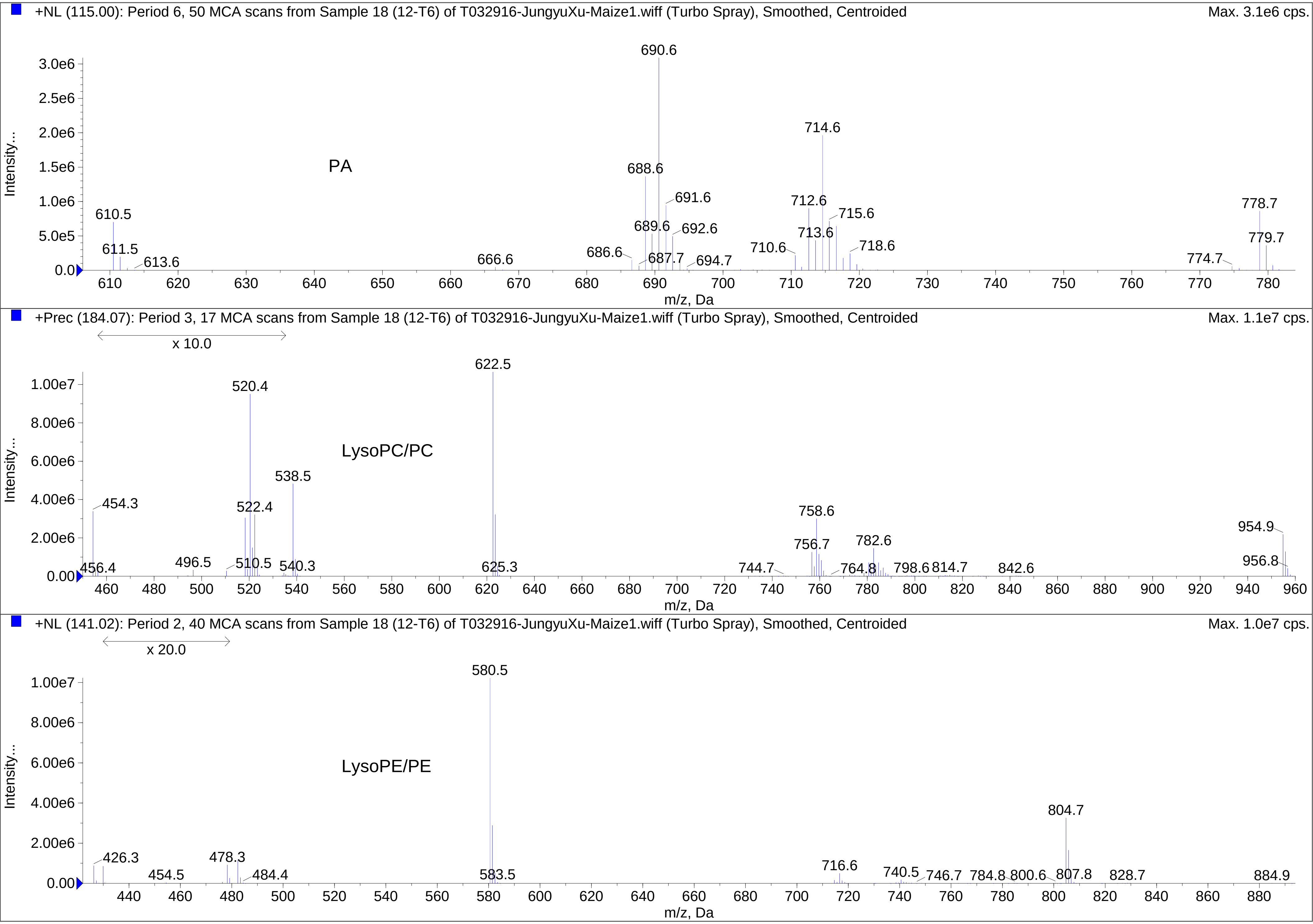

+NL (115.00): Period 6, 50 MCA scans from Sample 18 (12-T6) of T032916-JungyuXu-Maize1.wiff (Turbo Spray), Smoothed, Centroided
Max. 3.1e6 cps.
PA
690.6
714.6
688.6
691.6
712.6
715.6
610.5
689.6
692.6
713.6
611.5
613.6
666.6
686.6
687.7
694.7
710.6
718.6
778.7
779.7
774.7
Intensity...
m/z, Da
+Prec (184.07): Period 3, 17 MCA scans from Sample 18 (12-T6) of T032916-JungyuXu-Maize1.wiff (Turbo Spray), Smoothed, Centroided
Max. 1.1e7 cps.
x 10.0
LysoPC/PC
622.5
520.4
538.5
454.3
522.4
456.4
496.5
510.5
540.3
625.3
758.6
756.7
782.6
744.7
764.8
798.6
814.7
842.6
954.9
956.8
Intensity...
m/z, Da
+NL (141.02): Period 2, 40 MCA scans from Sample 18 (12-T6) of T032916-JungyuXu-Maize1.wiff (Turbo Spray), Smoothed, Centroided
Max. 1.0e7 cps.
x 20.0
LysoPE/PE
580.5
804.7
426.3
478.3
454.5
484.4
583.5
716.6
740.5
746.7
784.8
800.6
807.8
828.7
884.9
Intensity...
m/z, Da

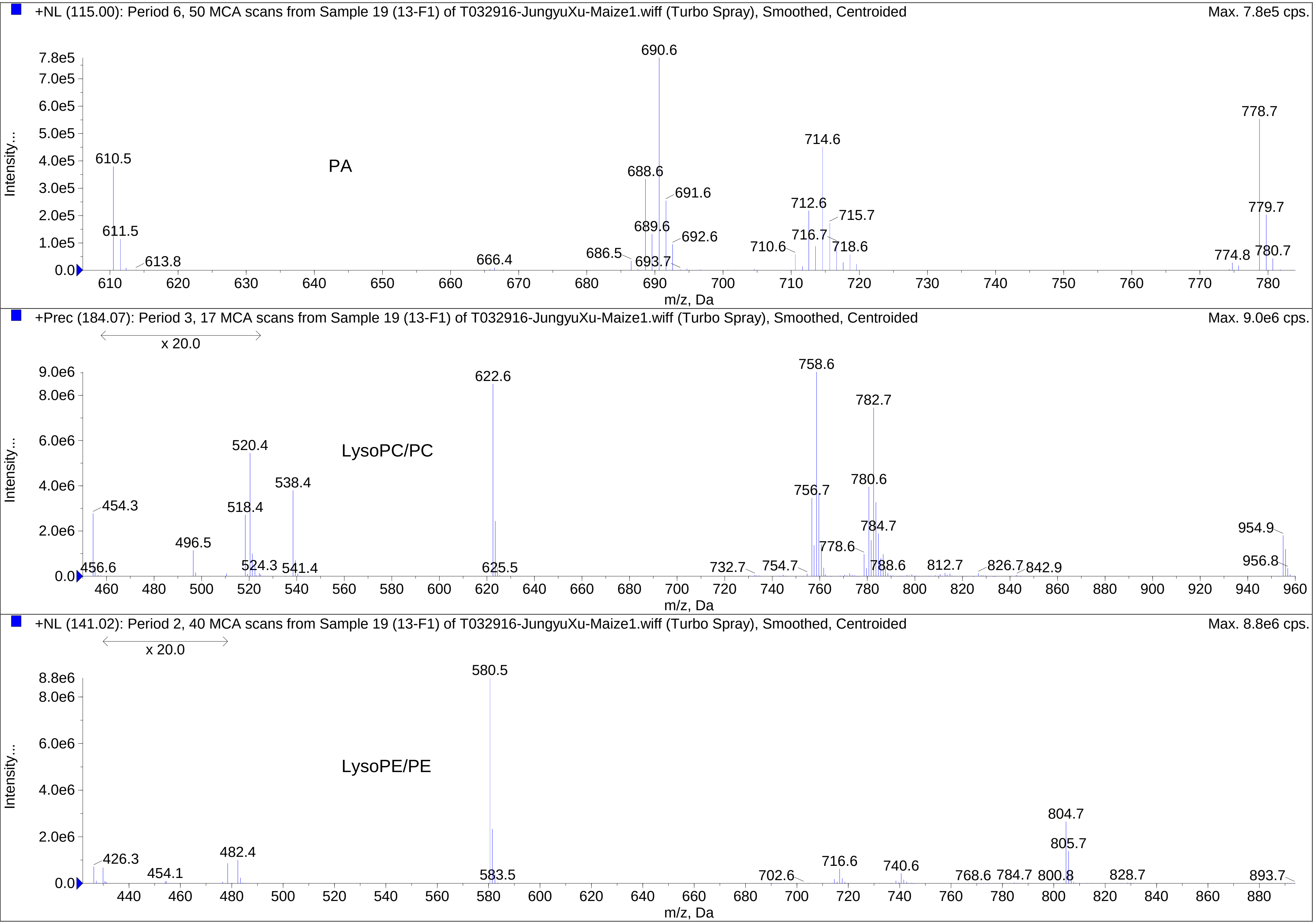

+NL (115.00): Period 6, 50 MCA scans from Sample 19 (13-F1) of T032916-JungyuXu-Maize1.wiff (Turbo Spray), Smoothed, Centroided
Max. 7.8e5 cps.
PA
Intensity...
m/z, Da
690.6
778.7
714.6
610.5
688.6
691.6
712.6
715.7
779.7
611.5
689.6
716.7
692.6
710.6
718.6
686.5
774.8
780.7
666.4
693.7
613.8
+Prec (184.07): Period 3, 17 MCA scans from Sample 19 (13-F1) of T032916-JungyuXu-Maize1.wiff (Turbo Spray), Smoothed, Centroided
Max. 9.0e6 cps.
x 20.0
LysoPC/PC
758.6
622.6
782.7
520.4
538.4
780.6
756.7
454.3
518.4
784.7
954.9
496.5
778.6
956.8
456.6
524.3
541.4
625.5
732.7
754.7
788.6
812.7
826.7
842.9
+NL (141.02): Period 2, 40 MCA scans from Sample 19 (13-F1) of T032916-JungyuXu-Maize1.wiff (Turbo Spray), Smoothed, Centroided
Max. 8.8e6 cps.
x 20.0
LysoPE/PE
580.5
804.7
805.7
426.3
482.4
454.1
583.5
702.6
716.6
740.6
768.6
784.7
800.8
828.7
893.7

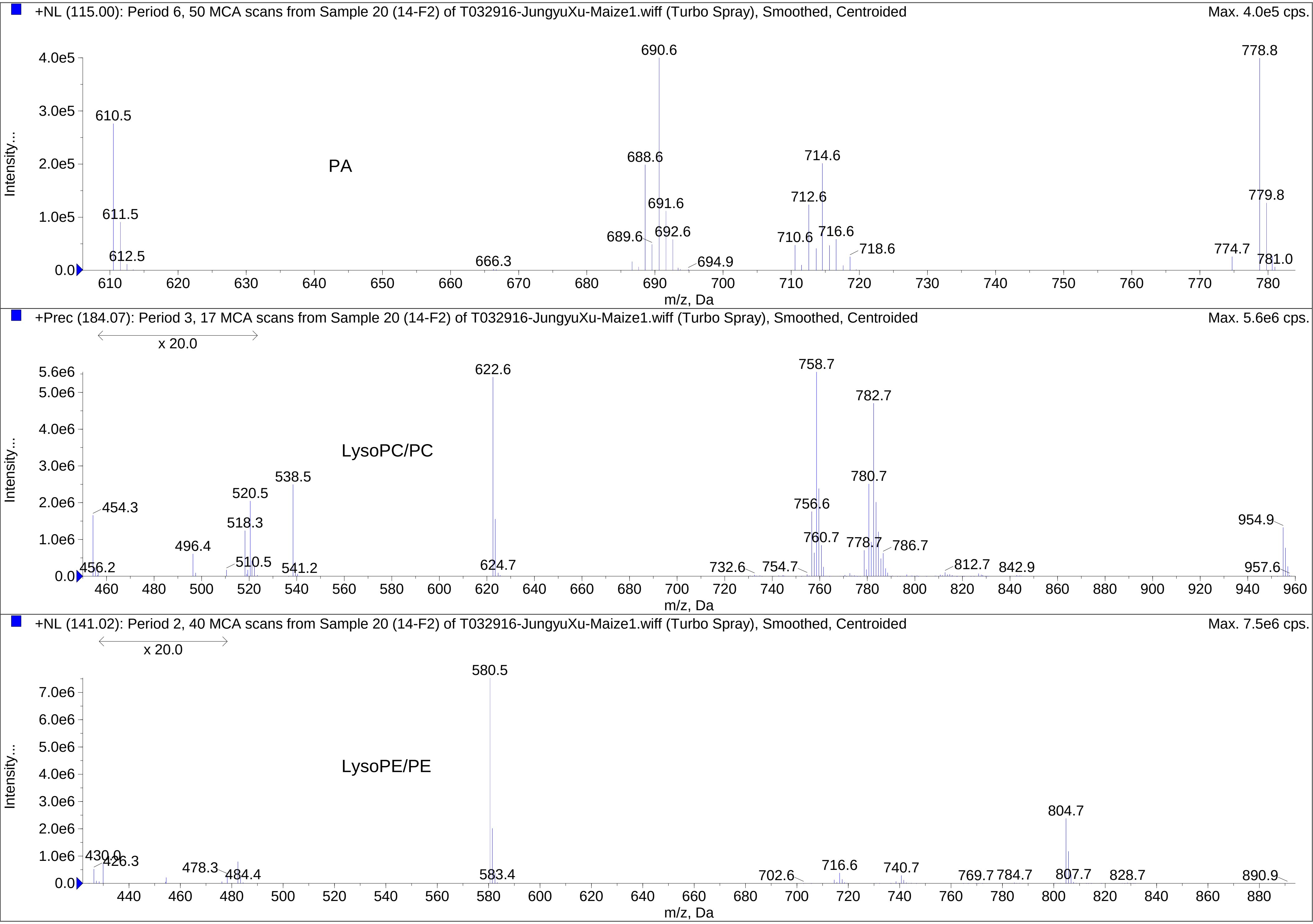
+NL (115.00): Period 6, 50 MCA scans from Sample 20 (14-F2) of T032916-JungyuXu-Maize1.wiff (Turbo Spray), Smoothed, Centroided
Max. 4.0e5 cps.
PA
Intensity...
m/z, Da
610.5
611.5
612.5
666.3
688.6
689.6
690.6
691.6
692.6
694.9
710.6
712.6
714.6
716.6
718.6
774.7
778.8
779.8
781.0
+Prec (184.07): Period 3, 17 MCA scans from Sample 20 (14-F2) of T032916-JungyuXu-Maize1.wiff (Turbo Spray), Smoothed, Centroided
Max. 5.6e6 cps.
x 20.0
LysoPC/PC
Intensity...
m/z, Da
454.3
456.2
496.4
510.5
518.3
520.5
538.5
541.2
622.6
624.7
732.6
754.7
756.6
758.7
760.7
778.7
780.7
782.7
786.7
812.7
842.9
954.9
957.6
+NL (141.02): Period 2, 40 MCA scans from Sample 20 (14-F2) of T032916-JungyuXu-Maize1.wiff (Turbo Spray), Smoothed, Centroided
Max. 7.5e6 cps.
x 20.0
LysoPE/PE
Intensity...
m/z, Da
430.0
426.3
478.3
484.4
580.5
583.4
702.6
716.6
740.7
769.7
784.7
804.7
807.7
828.7
890.9

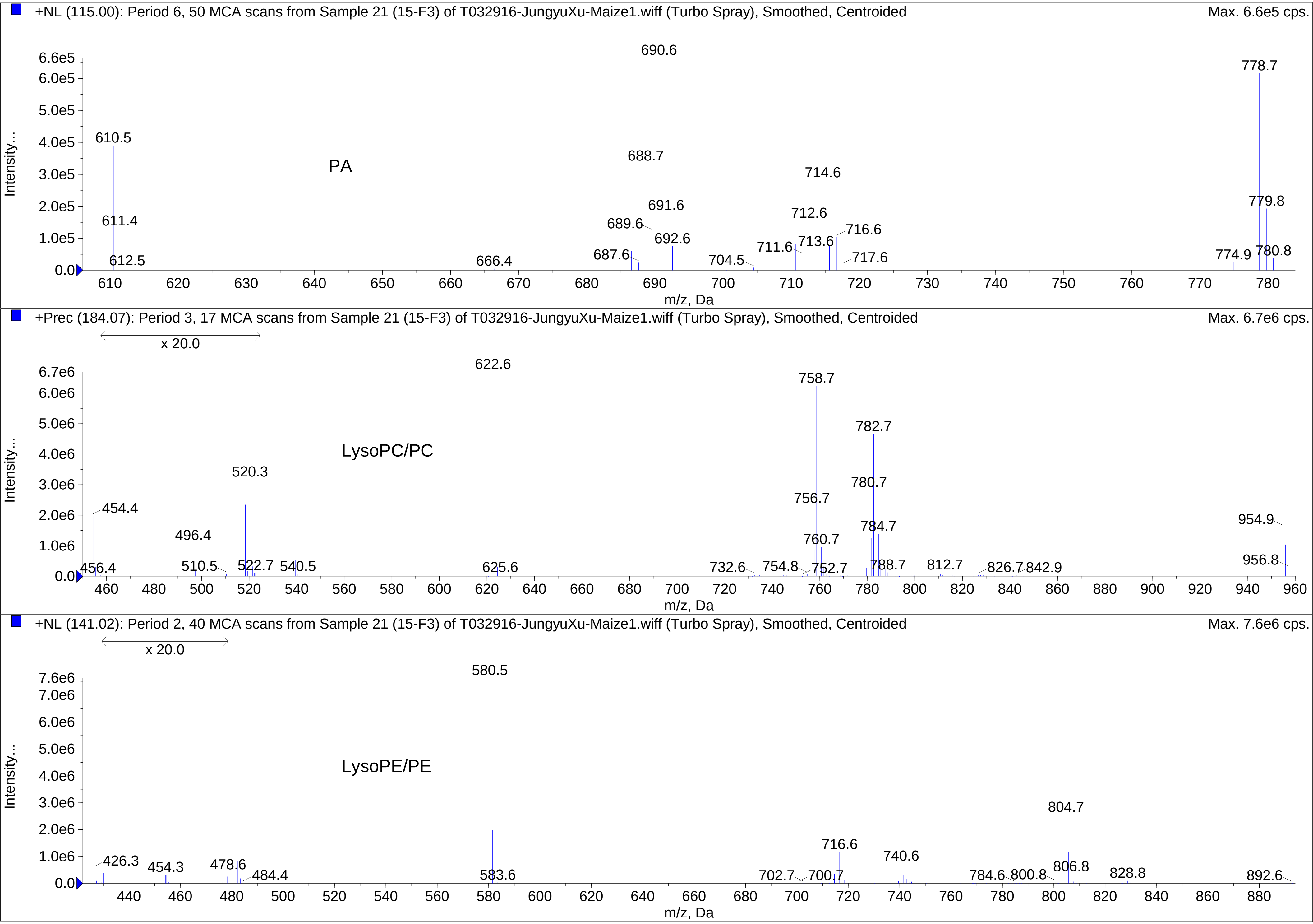

+NL (115.00): Period 6, 50 MCA scans from Sample 21 (15-F3) of T032916-JungyuXu-Maize1.wiff (Turbo Spray), Smoothed, Centroided
Max. 6.6e5 cps.
PA
Intensity...
m/z, Da
+Prec (184.07): Period 3, 17 MCA scans from Sample 21 (15-F3) of T032916-JungyuXu-Maize1.wiff (Turbo Spray), Smoothed, Centroided
Max. 6.7e6 cps.
x 20.0
LysoPC/PC
Intensity...
m/z, Da
+NL (141.02): Period 2, 40 MCA scans from Sample 21 (15-F3) of T032916-JungyuXu-Maize1.wiff (Turbo Spray), Smoothed, Centroided
Max. 7.6e6 cps.
x 20.0
LysoPE/PE
Intensity...
m/z, Da

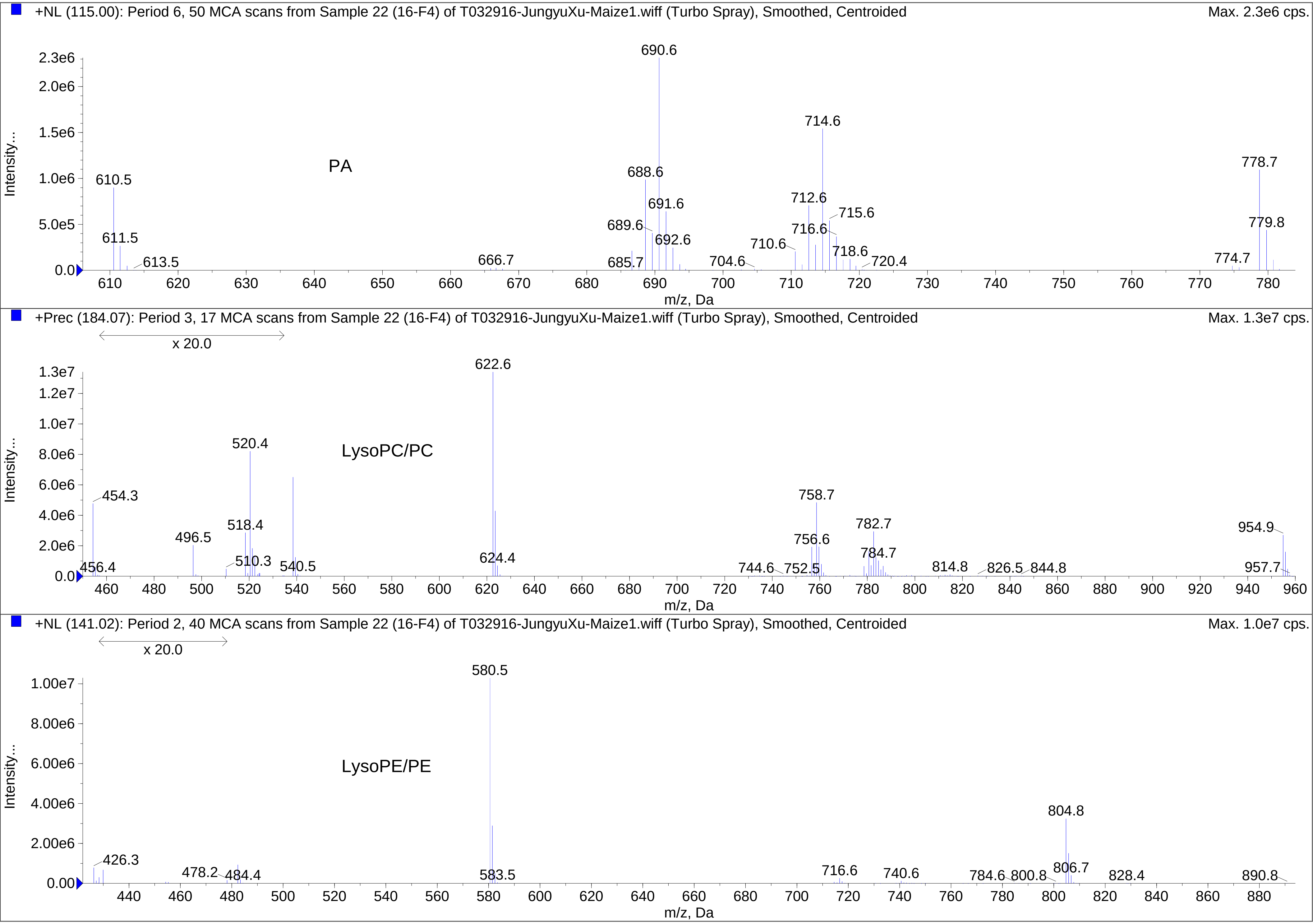
+NL (115.00): Period 6, 50 MCA scans from Sample 22 (16-F4) of T032916-JungyuXu-Maize1.wiff (Turbo Spray), Smoothed, Centroided
Max. 2.3e6 cps.
PA
Intensity...
m/z, Da
690.6
714.6
778.7
688.6
610.5
691.6
712.6
715.6
689.6
716.6
779.8
611.5
692.6
710.6
718.6
666.7
685.7
704.6
720.4
774.7
613.5
+Prec (184.07): Period 3, 17 MCA scans from Sample 22 (16-F4) of T032916-JungyuXu-Maize1.wiff (Turbo Spray), Smoothed, Centroided
Max. 1.3e7 cps.
x 20.0
LysoPC/PC
Intensity...
m/z, Da
622.6
520.4
454.3
758.7
518.4
782.7
496.5
756.6
954.9
510.3
784.7
624.4
456.4
540.5
744.6
752.5
814.8
826.5
844.8
957.7
+NL (141.02): Period 2, 40 MCA scans from Sample 22 (16-F4) of T032916-JungyuXu-Maize1.wiff (Turbo Spray), Smoothed, Centroided
Max. 1.0e7 cps.
x 20.0
LysoPE/PE
Intensity...
m/z, Da
580.5
804.8
426.3
478.2
484.4
583.5
716.6
740.6
784.6
800.8
806.7
828.4
890.8

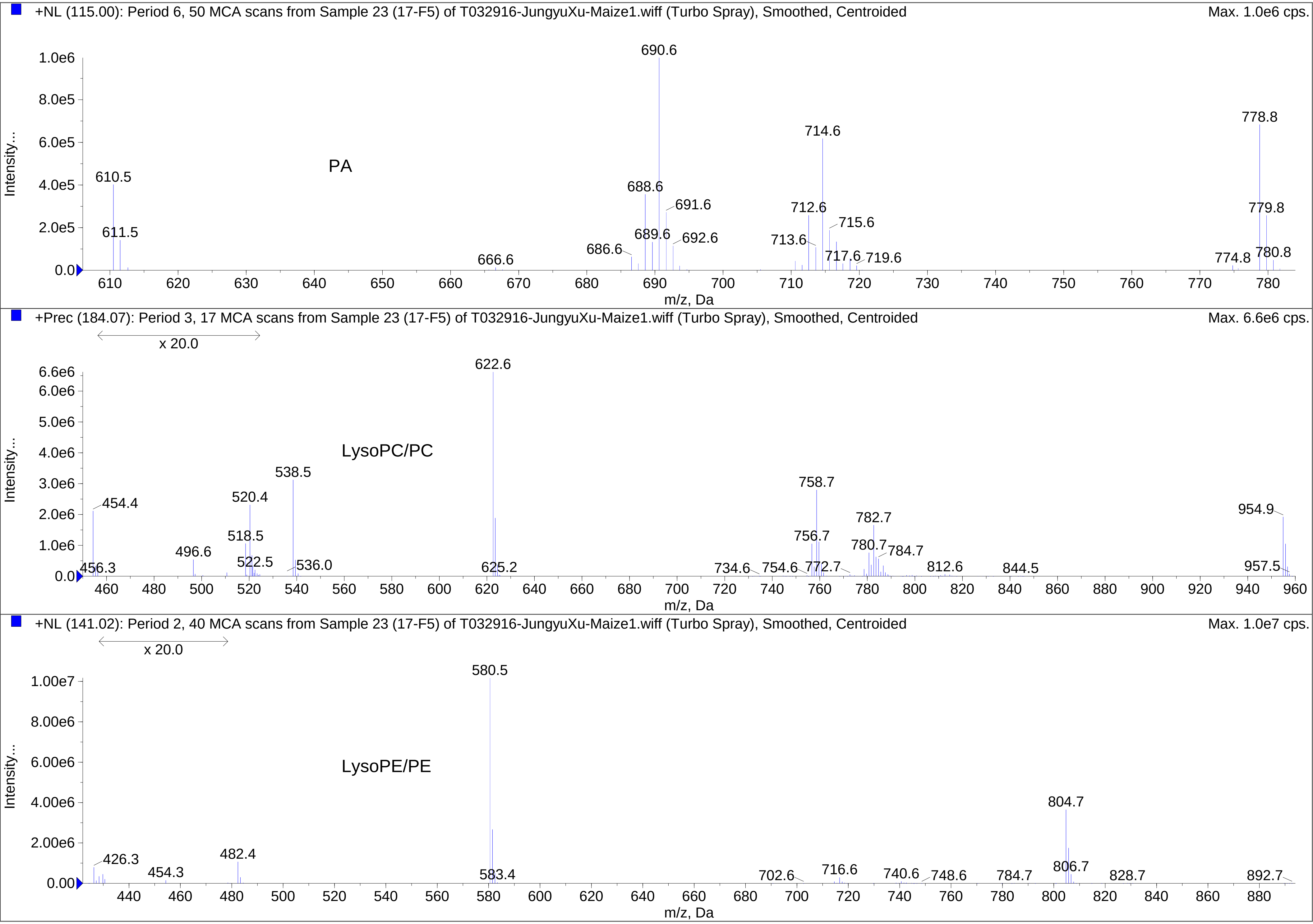

+NL (115.00): Period 6, 50 MCA scans from Sample 23 (17-F5) of T032916-JungyuXu-Maize1.wiff (Turbo Spray), Smoothed, Centroided
Max. 1.0e6 cps.
PA
Intensity...
m/z, Da
610.5
611.5
666.6
686.6
688.6
689.6
690.6
691.6
692.6
712.6
713.6
714.6
715.6
717.6
719.6
774.8
778.8
779.8
780.8
+Prec (184.07): Period 3, 17 MCA scans from Sample 23 (17-F5) of T032916-JungyuXu-Maize1.wiff (Turbo Spray), Smoothed, Centroided
Max. 6.6e6 cps.
x 20.0
LysoPC/PC
454.4
456.3
496.6
518.5
520.4
522.5
536.0
538.5
622.6
625.2
734.6
754.6
756.7
758.7
772.7
780.7
782.7
784.7
812.6
844.5
954.9
957.5
+NL (141.02): Period 2, 40 MCA scans from Sample 23 (17-F5) of T032916-JungyuXu-Maize1.wiff (Turbo Spray), Smoothed, Centroided
Max. 1.0e7 cps.
x 20.0
LysoPE/PE
426.3
454.3
482.4
580.5
583.4
702.6
716.6
740.6
748.6
784.7
804.7
806.7
828.7
892.7

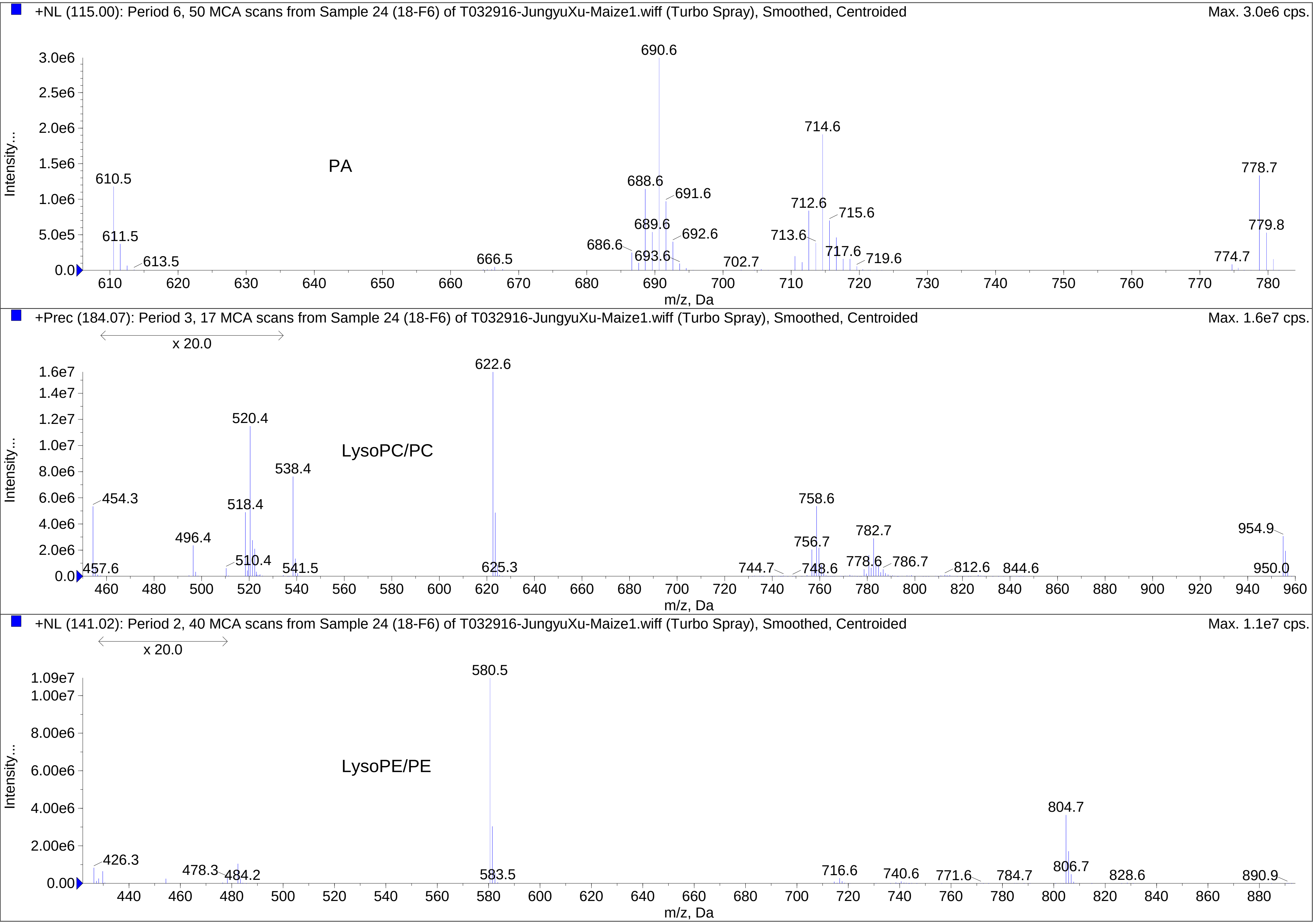

+NL (115.00): Period 6, 50 MCA scans from Sample 24 (18-F6) of T032916-JungyuXu-Maize1.wiff (Turbo Spray), Smoothed, Centroided
Max. 3.0e6 cps.
PA
Intensity...
m/z, Da
690.6
714.6
778.7
610.5
688.6
691.6
689.6
692.6
712.6
715.6
713.6
779.8
611.5
613.5
666.5
686.6
693.6
702.7
717.6
719.6
774.7
+Prec (184.07): Period 3, 17 MCA scans from Sample 24 (18-F6) of T032916-JungyuXu-Maize1.wiff (Turbo Spray), Smoothed, Centroided
Max. 1.6e7 cps.
x 20.0
LysoPC/PC
622.6
520.4
538.4
454.3
518.4
496.4
510.4
457.6
541.5
625.3
758.6
756.7
782.7
744.7
748.6
778.6
786.7
812.6
844.6
954.9
950.0
+NL (141.02): Period 2, 40 MCA scans from Sample 24 (18-F6) of T032916-JungyuXu-Maize1.wiff (Turbo Spray), Smoothed, Centroided
Max. 1.1e7 cps.
x 20.0
LysoPE/PE
580.5
583.5
426.3
478.3
484.2
716.6
740.6
771.6
784.7
804.7
806.7
828.6
890.9

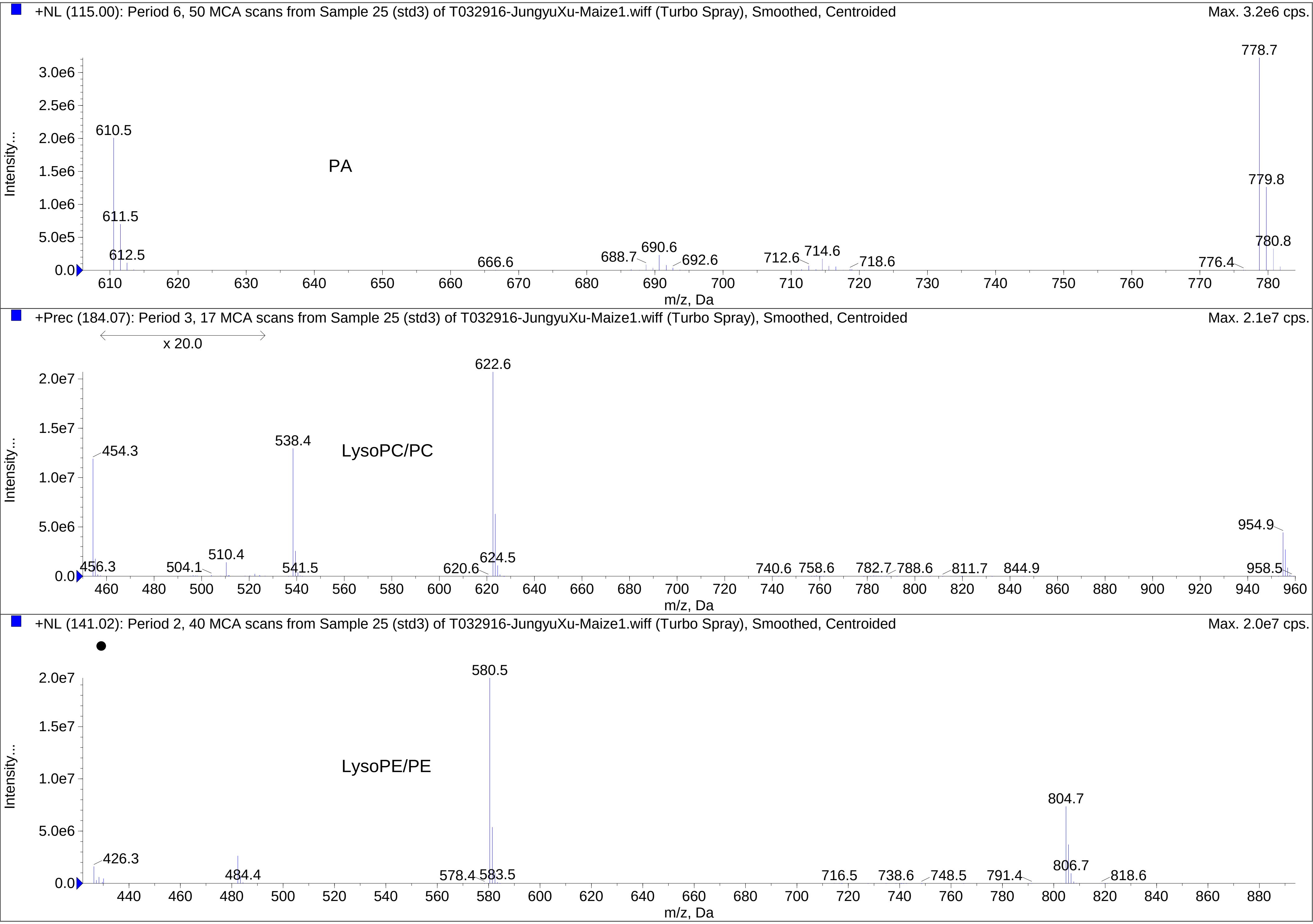

+NL (115.00): Period 6, 50 MCA scans from Sample 25 (std3) of T032916-JungyuXu-Maize1.wiff (Turbo Spray), Smoothed, Centroided
Max. 3.2e6 cps.
PA
610.5
611.5
612.5
666.6
688.7
690.6
692.6
712.6
714.6
718.6
776.4
778.7
779.8
780.8
Intensity...
m/z, Da
+Prec (184.07): Period 3, 17 MCA scans from Sample 25 (std3) of T032916-JungyuXu-Maize1.wiff (Turbo Spray), Smoothed, Centroided
Max. 2.1e7 cps.
x 20.0
LysoPC/PC
454.3
456.3
504.1
510.4
538.4
541.5
620.6
622.6
624.5
740.6
758.6
782.7
788.6
811.7
844.9
954.9
958.5
+NL (141.02): Period 2, 40 MCA scans from Sample 25 (std3) of T032916-JungyuXu-Maize1.wiff (Turbo Spray), Smoothed, Centroided
Max. 2.0e7 cps.
LysoPE/PE
426.3
484.4
578.4
580.5
583.5
716.5
738.6
748.5
791.4
804.7
806.7
818.6

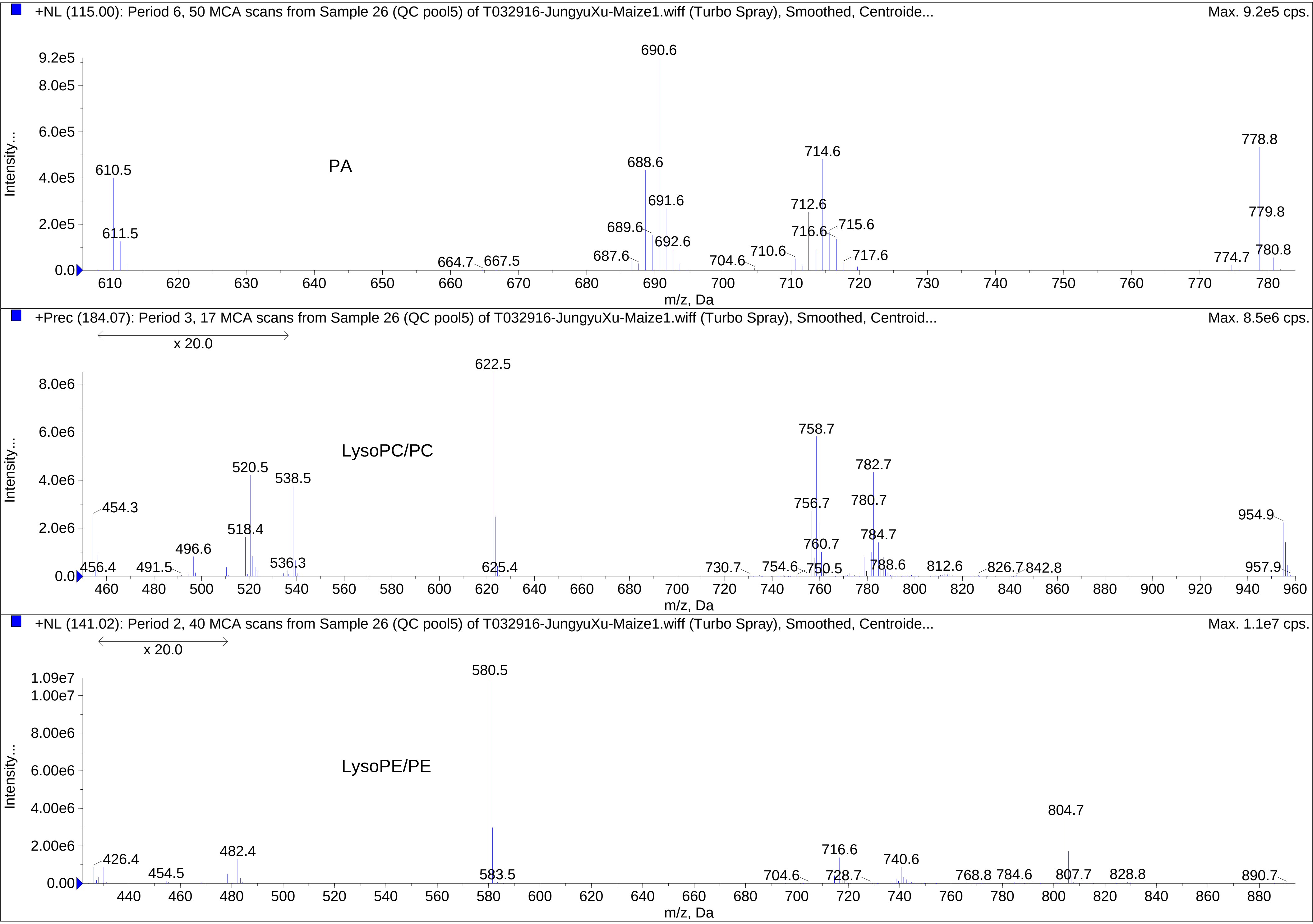
+NL (115.00): Period 6, 50 MCA scans from Sample 26 (QC pool5) of T032916-JungyuXu-Maize1.wiff (Turbo Spray), Smoothed, Centroide...
Max. 9.2e5 cps.
PA
Intensity...
m/z, Da
+Prec (184.07): Period 3, 17 MCA scans from Sample 26 (QC pool5) of T032916-JungyuXu-Maize1.wiff (Turbo Spray), Smoothed, Centroid...
Max. 8.5e6 cps.
x 20.0
LysoPC/PC
+NL (141.02): Period 2, 40 MCA scans from Sample 26 (QC pool5) of T032916-JungyuXu-Maize1.wiff (Turbo Spray), Smoothed, Centroide...
Max. 1.1e7 cps.
x 20.0
LysoPE/PE

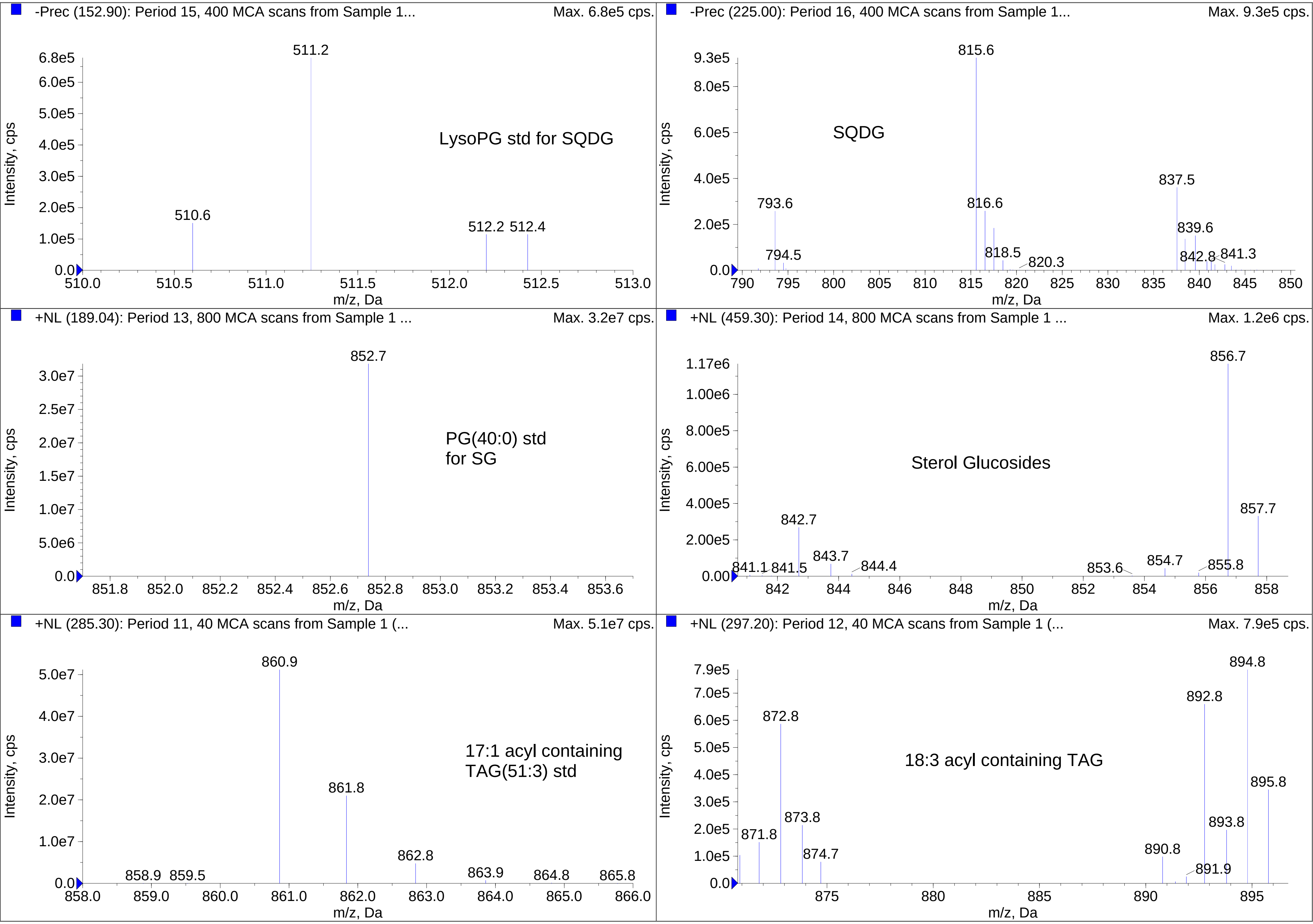

-Prec (152.90): Period 15, 400 MCA scans from Sample 1...
Max. 6.8e5 cps.
LysoPG std for SQDG
511.2
510.6
512.2
512.4
Intensity, cps
m/z, Da
-Prec (225.00): Period 16, 400 MCA scans from Sample 1...
Max. 9.3e5 cps.
SQDG
815.6
793.6
794.5
816.6
818.5
820.3
837.5
839.6
842.8
841.3
+NL (189.04): Period 13, 800 MCA scans from Sample 1 ...
Max. 3.2e7 cps.
PG(40:0) std
for SG
852.7
+NL (459.30): Period 14, 800 MCA scans from Sample 1 ...
Max. 1.2e6 cps.
Sterol Glucosides
856.7
857.7
842.7
843.7
844.4
841.1
841.5
853.6
854.7
855.8
+NL (285.30): Period 11, 40 MCA scans from Sample 1 (...
Max. 5.1e7 cps.
17:1 acyl containing
TAG(51:3) std
860.9
861.8
862.8
863.9
864.8
865.8
858.9
859.5
+NL (297.20): Period 12, 40 MCA scans from Sample 1 (...
Max. 7.9e5 cps.
18:3 acyl containing TAG
894.8
892.8
872.8
895.8
873.8
893.8
871.8
874.7
890.8
891.9

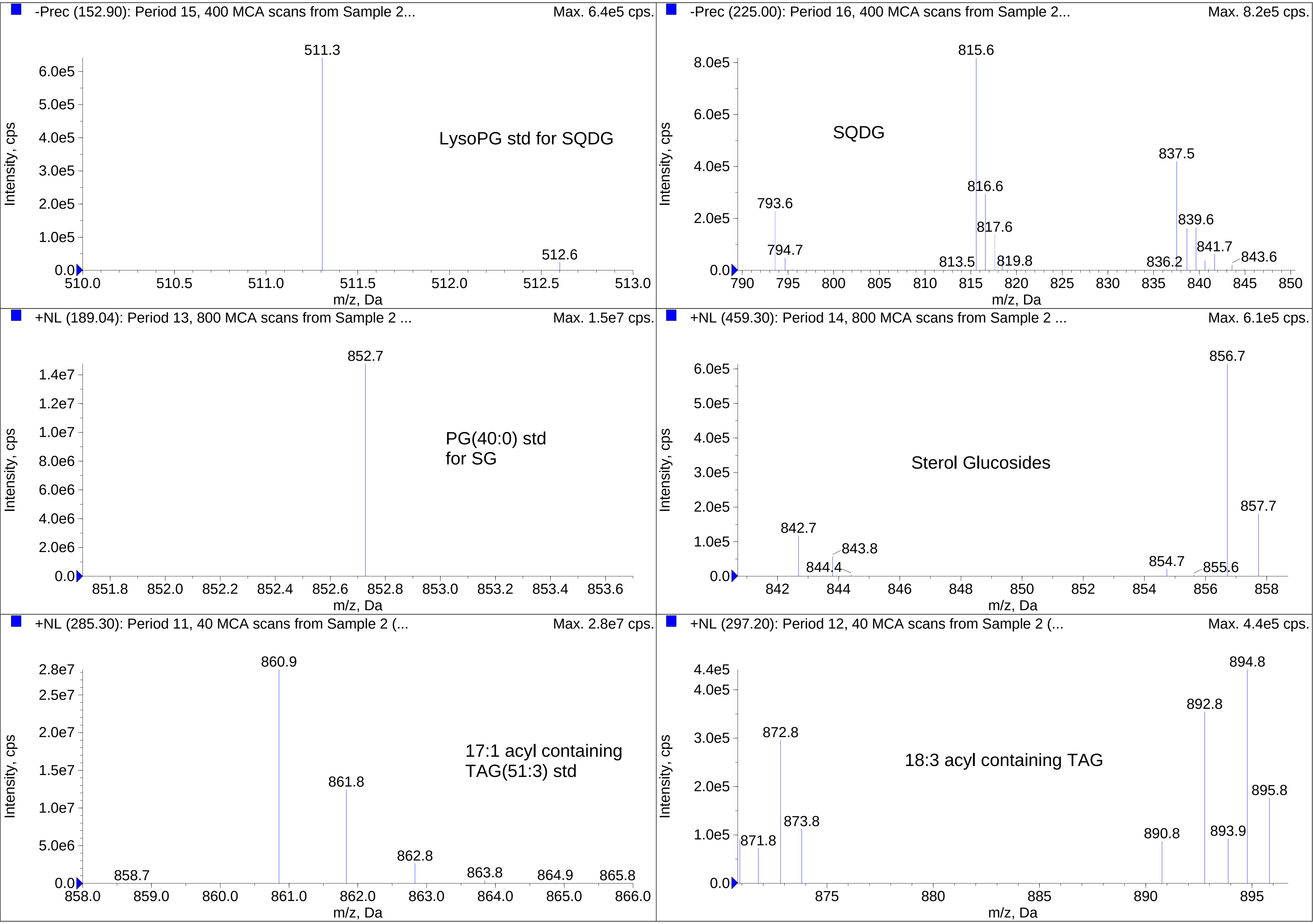
-Prec (152.90): Period 15, 400 MCA scans from Sample 2...
Max. 6.4e5 cps.
LysoPG std for SQDG
511.3
512.6
Intensity, cps
m/z, Da
-Prec (225.00): Period 16, 400 MCA scans from Sample 2...
Max. 8.2e5 cps.
SQDG
815.6
837.5
816.6
793.6
839.6
817.6
841.7
794.7
843.6
813.5
819.8
836.2
+NL (189.04): Period 13, 800 MCA scans from Sample 2 ...
Max. 1.5e7 cps.
PG(40:0) std
for SG
852.7
+NL (459.30): Period 14, 800 MCA scans from Sample 2 ...
Max. 6.1e5 cps.
Sterol Glucosides
856.7
857.7
842.7
843.8
844.4
854.7
855.6
+NL (285.30): Period 11, 40 MCA scans from Sample 2 (...
Max. 2.8e7 cps.
17:1 acyl containing
TAG(51:3) std
860.9
861.8
862.8
858.7
863.8
864.9
865.8
+NL (297.20): Period 12, 40 MCA scans from Sample 2 (...
Max. 4.4e5 cps.
18:3 acyl containing TAG
894.8
892.8
872.8
895.8
873.8
890.8
893.9
871.8

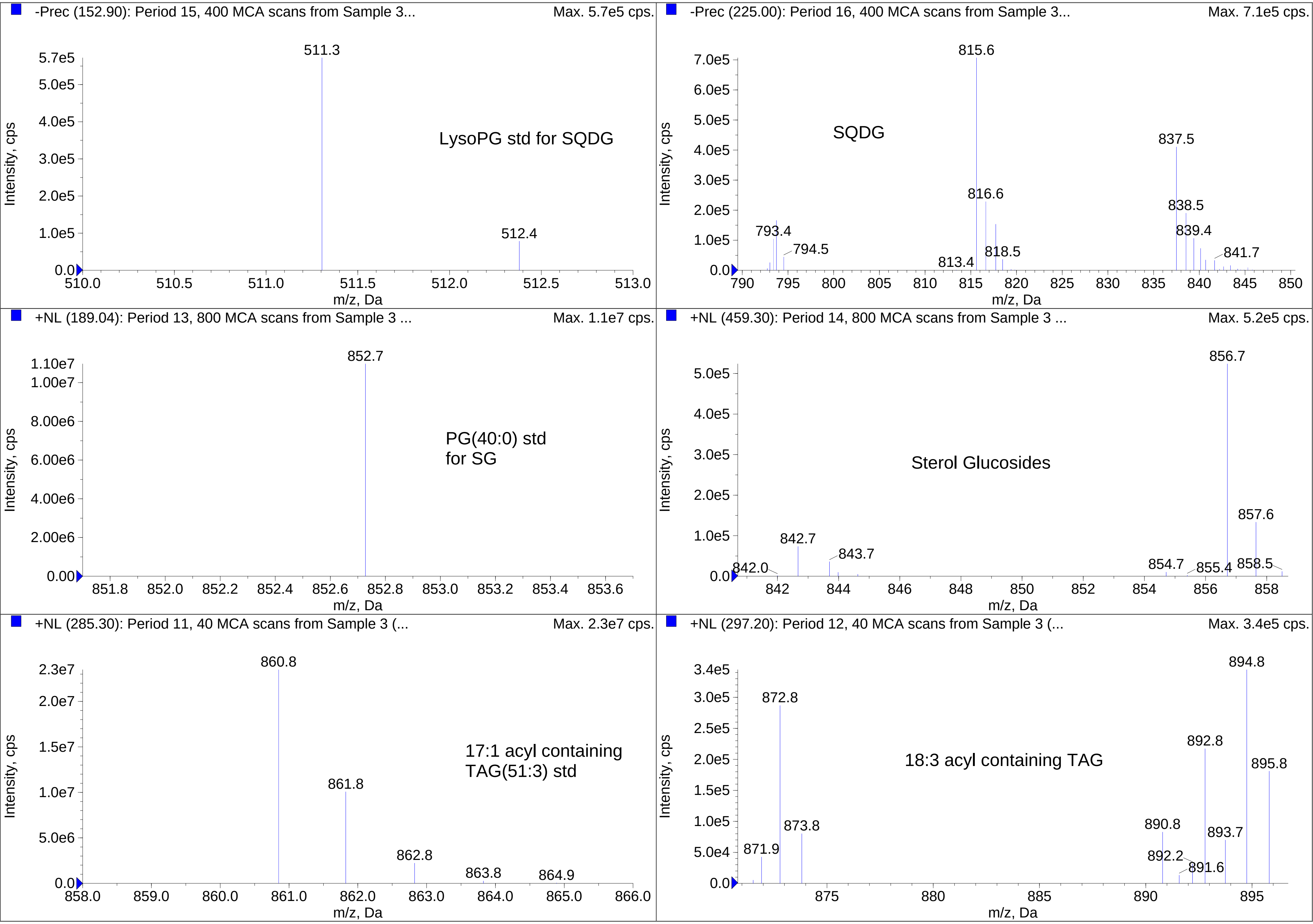

-Prec (152.90): Period 15, 400 MCA scans from Sample 3...
Max. 5.7e5 cps.
LysoPG std for SQDG
511.3
512.4
Intensity, cps
m/z, Da
-Prec (225.00): Period 16, 400 MCA scans from Sample 3...
Max. 7.1e5 cps.
SQDG
815.6
837.5
816.6
838.5
839.4
793.4
794.5
841.7
818.5
813.4
Intensity, cps
m/z, Da
+NL (189.04): Period 13, 800 MCA scans from Sample 3 ...
Max. 1.1e7 cps.
PG(40:0) std
for SG
852.7
Intensity, cps
m/z, Da
+NL (459.30): Period 14, 800 MCA scans from Sample 3 ...
Max. 5.2e5 cps.
Sterol Glucosides
856.7
857.6
842.7
843.7
842.0
854.7
855.4
858.5
Intensity, cps
m/z, Da
+NL (285.30): Period 11, 40 MCA scans from Sample 3 (...
Max. 2.3e7 cps.
17:1 acyl containing
TAG(51:3) std
860.8
861.8
862.8
863.8
864.9
Intensity, cps
m/z, Da
+NL (297.20): Period 12, 40 MCA scans from Sample 3 (...
Max. 3.4e5 cps.
18:3 acyl containing TAG
894.8
872.8
892.8
895.8
873.8
890.8
893.7
871.9
892.2
891.6
Intensity, cps
m/z, Da

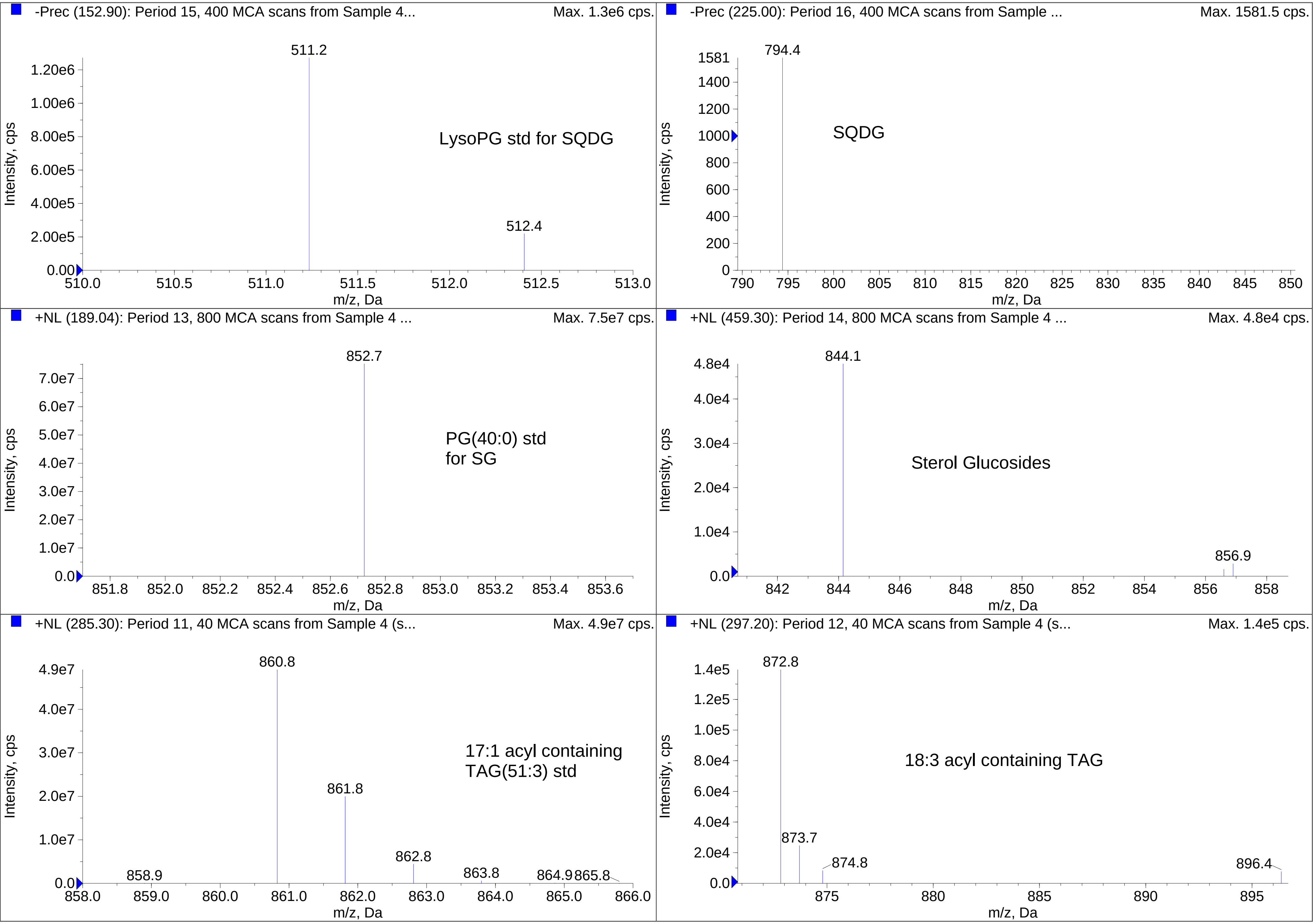
-Prec (152.90): Period 15, 400 MCA scans from Sample 4...
Max. 1.3e6 cps.
511.2
512.4
LysoPG std for SQDG
Intensity, cps
m/z, Da
-Prec (225.00): Period 16, 400 MCA scans from Sample ...
Max. 1581.5 cps.
794.4
SQDG
Intensity, cps
m/z, Da
+NL (189.04): Period 13, 800 MCA scans from Sample 4 ...
Max. 7.5e7 cps.
852.7
PG(40:0) std
for SG
Intensity, cps
m/z, Da
+NL (459.30): Period 14, 800 MCA scans from Sample 4 ...
Max. 4.8e4 cps.
844.1
856.9
Sterol Glucosides
Intensity, cps
m/z, Da
+NL (285.30): Period 11, 40 MCA scans from Sample 4 (s...
Max. 4.9e7 cps.
860.8
861.8
862.8
863.8
858.9
864.9
865.8
17:1 acyl containing
TAG(51:3) std
Intensity, cps
m/z, Da
+NL (297.20): Period 12, 40 MCA scans from Sample 4 (s...
Max. 1.4e5 cps.
872.8
873.7
874.8
896.4
18:3 acyl containing TAG
Intensity, cps
m/z, Da

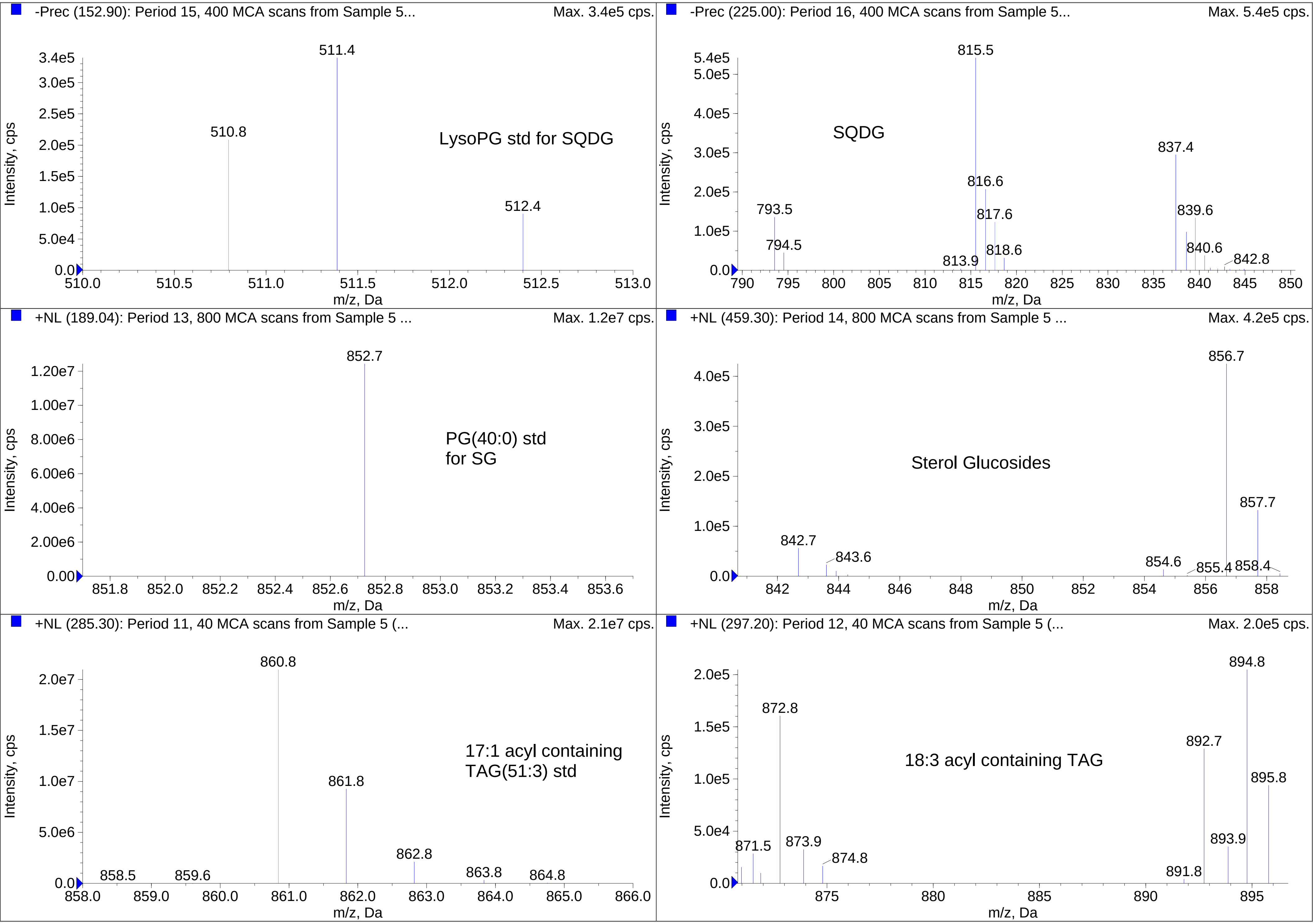
-Prec (152.90): Period 15, 400 MCA scans from Sample 5...
Max. 3.4e5 cps.
LysoPG std for SQDG
511.4
510.8
512.4
Intensity, cps
m/z, Da
-Prec (225.00): Period 16, 400 MCA scans from Sample 5...
Max. 5.4e5 cps.
SQDG
815.5
837.4
816.6
817.6
793.5
839.6
794.5
840.6
818.6
813.9
842.8
Intensity, cps
m/z, Da
+NL (189.04): Period 13, 800 MCA scans from Sample 5 ...
Max. 1.2e7 cps.
PG(40:0) std
for SG
852.7
Intensity, cps
m/z, Da
+NL (459.30): Period 14, 800 MCA scans from Sample 5 ...
Max. 4.2e5 cps.
Sterol Glucosides
856.7
857.7
842.7
843.6
854.6
855.4
858.4
Intensity, cps
m/z, Da
+NL (285.30): Period 11, 40 MCA scans from Sample 5 (...
Max. 2.1e7 cps.
17:1 acyl containing
TAG(51:3) std
860.8
861.8
862.8
863.8
864.8
858.5
859.6
Intensity, cps
m/z, Da
+NL (297.20): Period 12, 40 MCA scans from Sample 5 (...
Max. 2.0e5 cps.
18:3 acyl containing TAG
894.8
872.8
892.7
895.8
871.5
873.9
874.8
893.9
891.8
Intensity, cps
m/z, Da

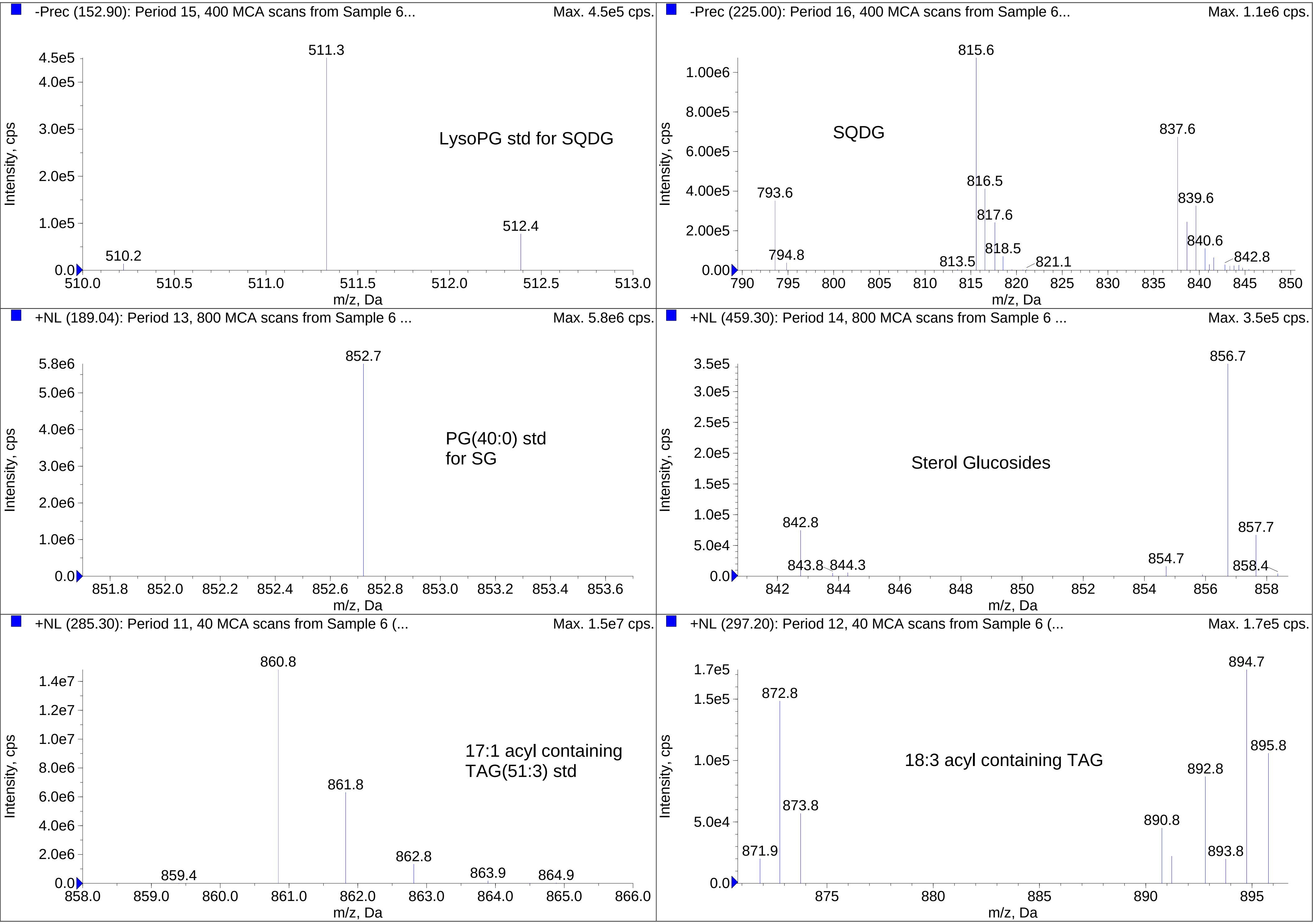

-Prec (152.90): Period 15, 400 MCA scans from Sample 6...
Max. 4.5e5 cps.
LysoPG std for SQDG
511.3
512.4
510.2
Intensity, cps
m/z, Da
-Prec (225.00): Period 16, 400 MCA scans from Sample 6...
Max. 1.1e6 cps.
SQDG
815.6
837.6
816.5
793.6
839.6
817.6
840.6
818.5
794.8
842.8
813.5
821.1
+NL (189.04): Period 13, 800 MCA scans from Sample 6 ...
Max. 5.8e6 cps.
PG(40:0) std
for SG
852.7
+NL (459.30): Period 14, 800 MCA scans from Sample 6 ...
Max. 3.5e5 cps.
Sterol Glucosides
856.7
842.8
857.7
854.7
843.8
844.3
858.4
+NL (285.30): Period 11, 40 MCA scans from Sample 6 (...
Max. 1.5e7 cps.
17:1 acyl containing
TAG(51:3) std
860.8
861.8
862.8
859.4
863.9
864.9
+NL (297.20): Period 12, 40 MCA scans from Sample 6 (...
Max. 1.7e5 cps.
18:3 acyl containing TAG
894.7
872.8
895.8
892.8
873.8
890.8
871.9
893.8

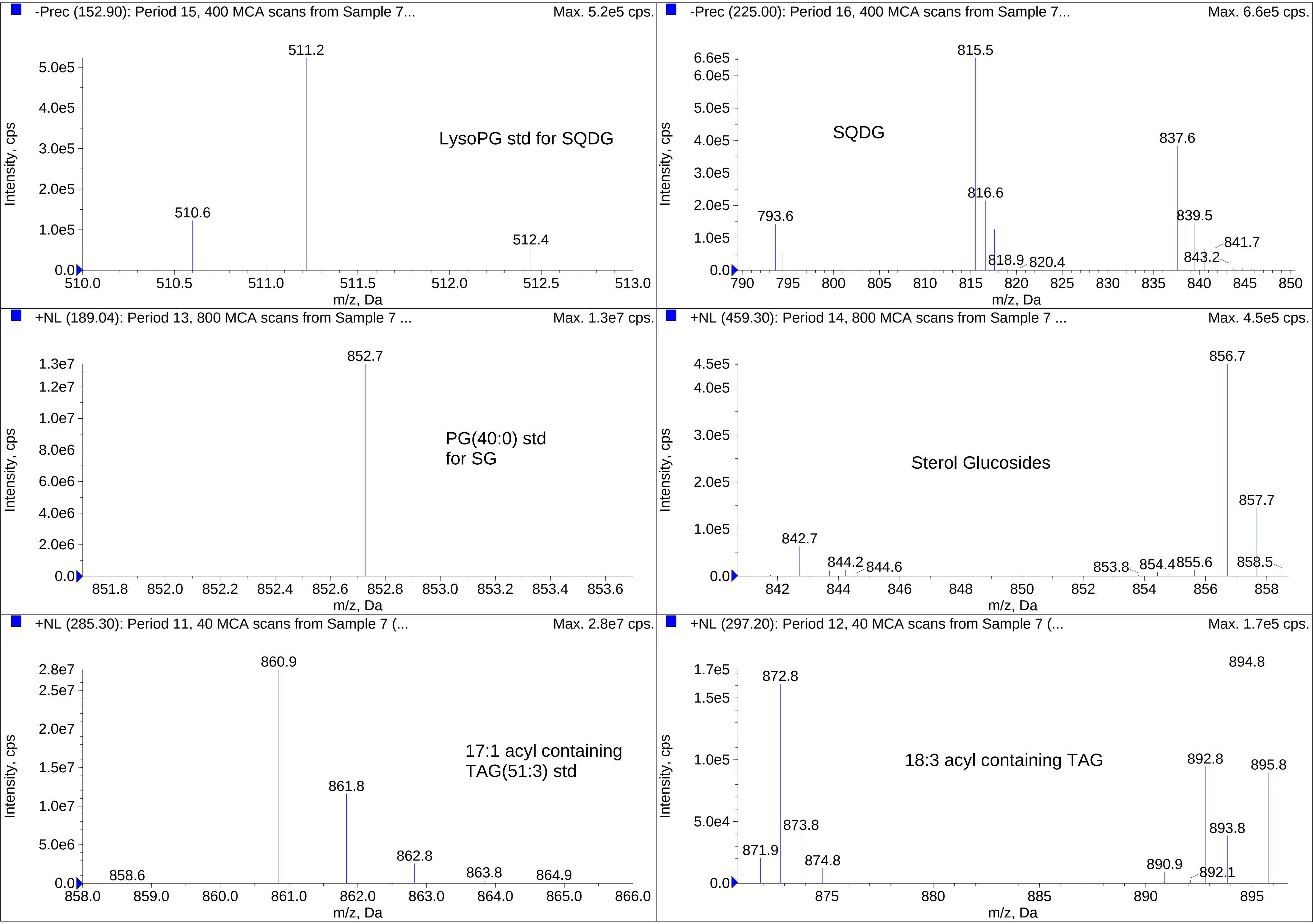
-Prec (152.90): Period 15, 400 MCA scans from Sample 7...
Max. 5.2e5 cps.
LysoPG std for SQDG
511.2
510.6
512.4
Intensity, cps
m/z, Da
-Prec (225.00): Period 16, 400 MCA scans from Sample 7...
Max. 6.6e5 cps.
SQDG
815.5
837.6
816.6
793.6
839.5
841.7
843.2
818.9
820.4
Intensity, cps
m/z, Da
+NL (189.04): Period 13, 800 MCA scans from Sample 7 ...
Max. 1.3e7 cps.
PG(40:0) std
for SG
852.7
Intensity, cps
m/z, Da
+NL (459.30): Period 14, 800 MCA scans from Sample 7 ...
Max. 4.5e5 cps.
Sterol Glucosides
856.7
857.7
842.7
844.2
844.6
853.8
854.4
855.6
858.5
Intensity, cps
m/z, Da
+NL (285.30): Period 11, 40 MCA scans from Sample 7 (...
Max. 2.8e7 cps.
17:1 acyl containing
TAG(51:3) std
860.9
861.8
862.8
863.8
864.9
858.6
Intensity, cps
m/z, Da
+NL (297.20): Period 12, 40 MCA scans from Sample 7 (...
Max. 1.7e5 cps.
18:3 acyl containing TAG
894.8
872.8
892.8
895.8
873.8
893.8
871.9
874.8
890.9
892.1
Intensity, cps
m/z, Da

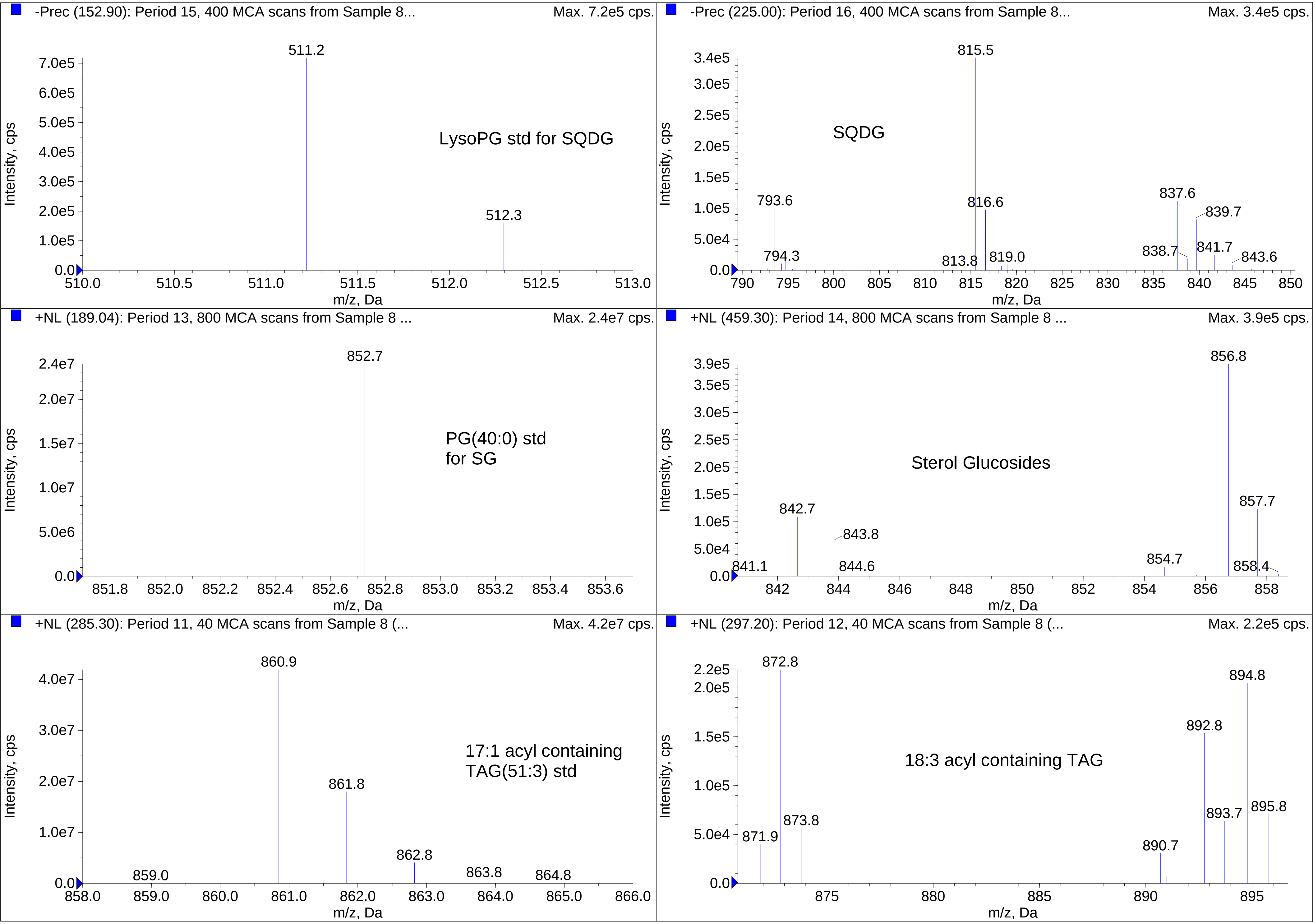
-Prec (152.90): Period 15, 400 MCA scans from Sample 8...
Max. 7.2e5 cps.
LysoPG std for SQDG
511.2
512.3
-Prec (225.00): Period 16, 400 MCA scans from Sample 8...
Max. 3.4e5 cps.
SQDG
815.5
793.6
794.3
813.8
816.6
819.0
837.6
838.7
839.7
841.7
843.6
+NL (189.04): Period 13, 800 MCA scans from Sample 8 ...
Max. 2.4e7 cps.
PG(40:0) std
for SG
852.7
+NL (459.30): Period 14, 800 MCA scans from Sample 8 ...
Max. 3.9e5 cps.
Sterol Glucosides
841.1
842.7
843.8
844.6
854.7
856.8
857.7
858.4
+NL (285.30): Period 11, 40 MCA scans from Sample 8 (...
Max. 4.2e7 cps.
17:1 acyl containing
TAG(51:3) std
859.0
860.9
861.8
862.8
863.8
864.8
+NL (297.20): Period 12, 40 MCA scans from Sample 8 (...
Max. 2.2e5 cps.
18:3 acyl containing TAG
871.9
872.8
873.8
890.7
892.8
893.7
894.8
895.8
Intensity, cps
m/z, Da

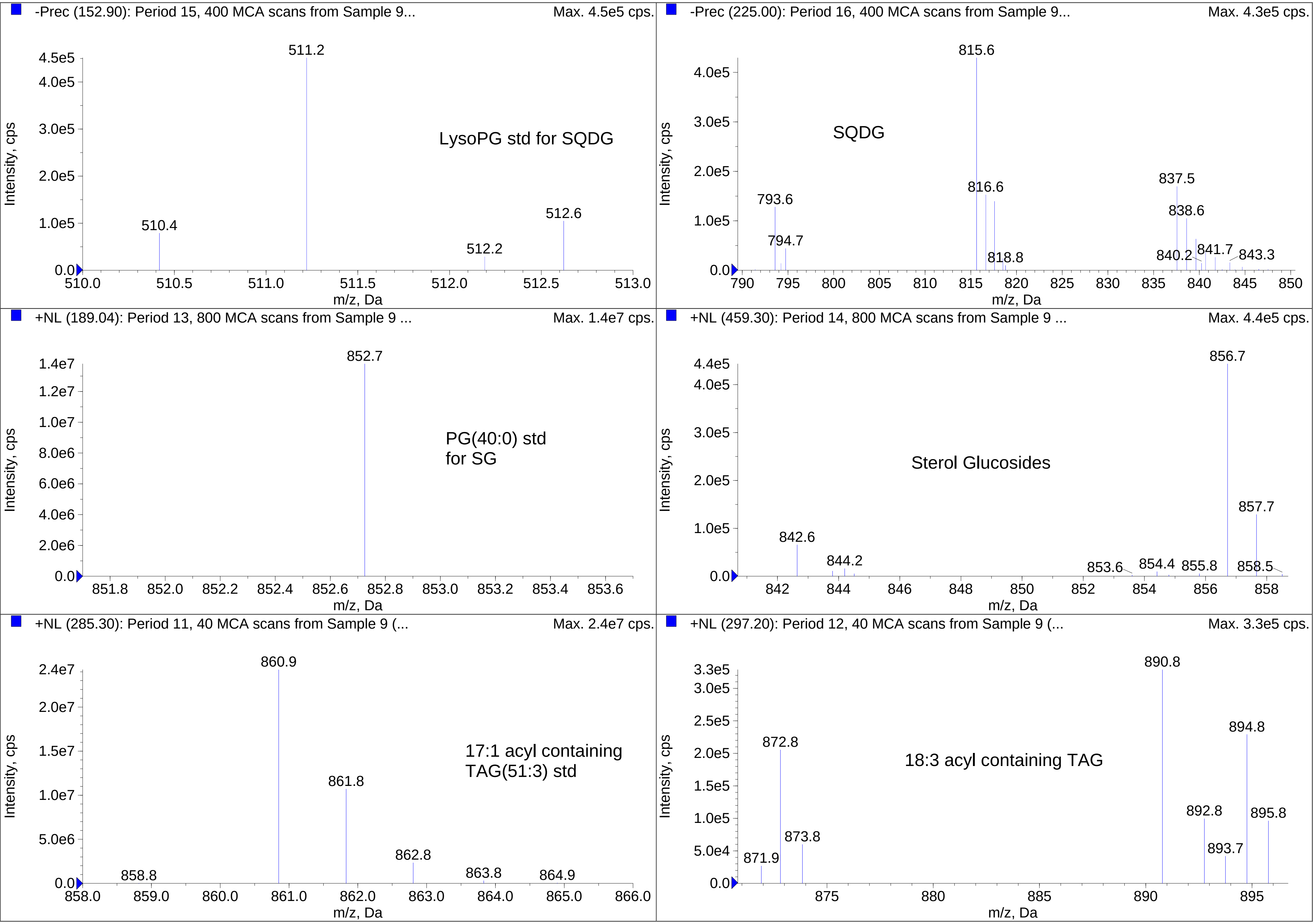
-Prec (152.90): Period 15, 400 MCA scans from Sample 9...
Max. 4.5e5 cps.
LysoPG std for SQDG
511.2
510.4
512.2
512.6
Intensity, cps
m/z, Da
-Prec (225.00): Period 16, 400 MCA scans from Sample 9...
Max. 4.3e5 cps.
SQDG
815.6
793.6
794.7
816.6
818.8
837.5
838.6
840.2
841.7
843.3
+NL (189.04): Period 13, 800 MCA scans from Sample 9 ...
Max. 1.4e7 cps.
PG(40:0) std
for SG
852.7
+NL (459.30): Period 14, 800 MCA scans from Sample 9 ...
Max. 4.4e5 cps.
Sterol Glucosides
856.7
857.7
842.6
844.2
853.6
854.4
855.8
858.5
+NL (285.30): Period 11, 40 MCA scans from Sample 9 (...
Max. 2.4e7 cps.
17:1 acyl containing
TAG(51:3) std
860.9
861.8
862.8
863.8
864.9
858.8
+NL (297.20): Period 12, 40 MCA scans from Sample 9 (...
Max. 3.3e5 cps.
18:3 acyl containing TAG
890.8
894.8
872.8
892.8
895.8
873.8
893.7
871.9

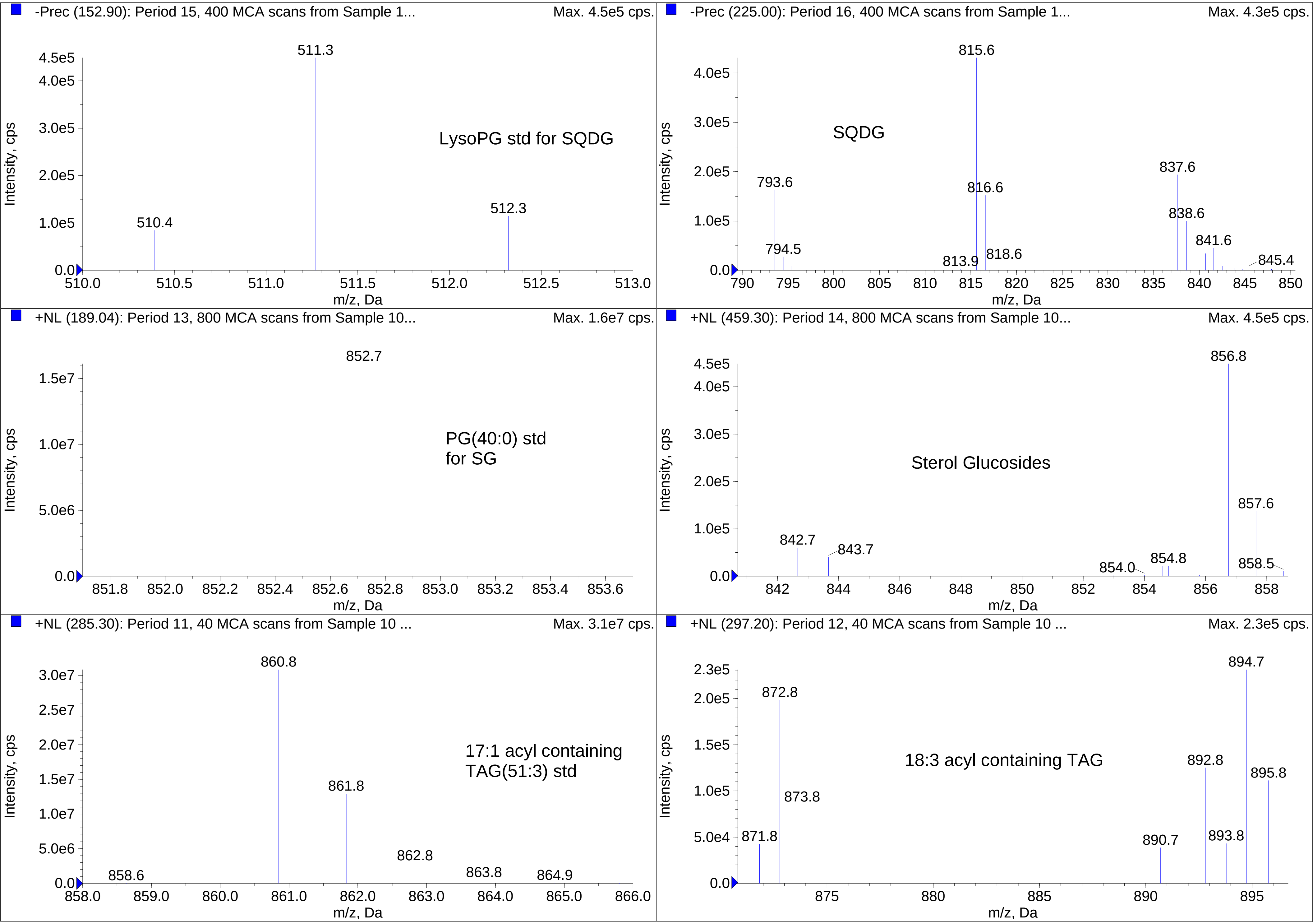
-Prec (152.90): Period 15, 400 MCA scans from Sample 1...
Max. 4.5e5 cps.
LysoPG std for SQDG
510.4
511.3
512.3
Intensity, cps
m/z, Da
-Prec (225.00): Period 16, 400 MCA scans from Sample 1...
Max. 4.3e5 cps.
SQDG
793.6
794.5
813.9
815.6
816.6
818.6
837.6
838.6
841.6
845.4
Intensity, cps
m/z, Da
+NL (189.04): Period 13, 800 MCA scans from Sample 10...
Max. 1.6e7 cps.
PG(40:0) std
for SG
852.7
Intensity, cps
m/z, Da
+NL (459.30): Period 14, 800 MCA scans from Sample 10...
Max. 4.5e5 cps.
Sterol Glucosides
842.7
843.7
854.0
854.8
856.8
857.6
858.5
Intensity, cps
m/z, Da
+NL (285.30): Period 11, 40 MCA scans from Sample 10 ...
Max. 3.1e7 cps.
17:1 acyl containing
TAG(51:3) std
858.6
860.8
861.8
862.8
863.8
864.9
Intensity, cps
m/z, Da
+NL (297.20): Period 12, 40 MCA scans from Sample 10 ...
Max. 2.3e5 cps.
18:3 acyl containing TAG
871.8
872.8
873.8
890.7
892.8
893.8
894.7
895.8
Intensity, cps
m/z, Da

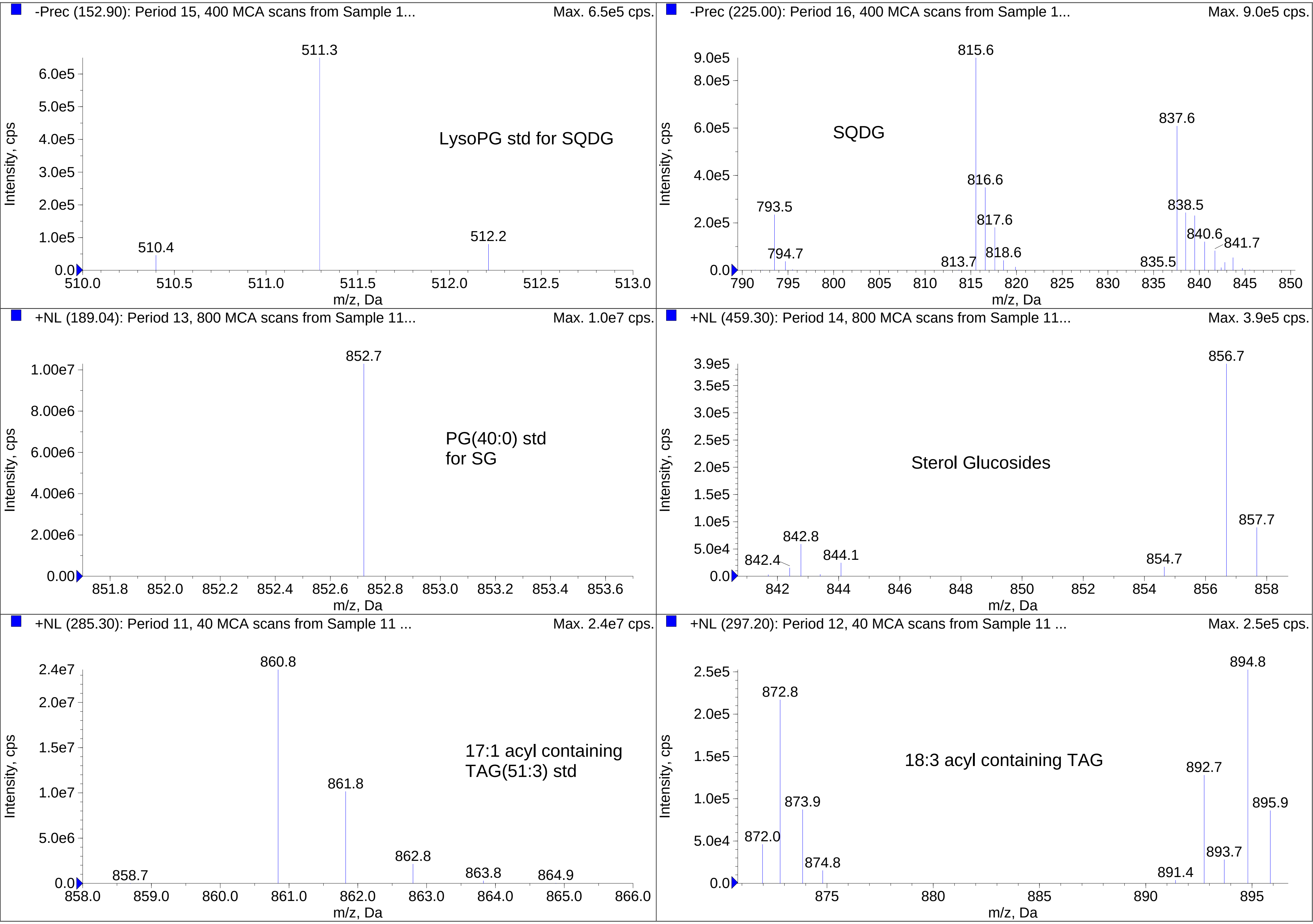

-Prec (152.90): Period 15, 400 MCA scans from Sample 1...
Max. 6.5e5 cps.
LysoPG std for SQDG
511.3
510.4
512.2
Intensity, cps
m/z, Da
-Prec (225.00): Period 16, 400 MCA scans from Sample 1...
Max. 9.0e5 cps.
SQDG
815.6
837.6
816.6
793.5
838.5
817.6
840.6
841.7
794.7
818.6
813.7
835.5
+NL (189.04): Period 13, 800 MCA scans from Sample 11...
Max. 1.0e7 cps.
PG(40:0) std
for SG
852.7
+NL (459.30): Period 14, 800 MCA scans from Sample 11...
Max. 3.9e5 cps.
Sterol Glucosides
856.7
857.7
842.8
842.4
844.1
854.7
+NL (285.30): Period 11, 40 MCA scans from Sample 11 ...
Max. 2.4e7 cps.
17:1 acyl containing
TAG(51:3) std
860.8
861.8
862.8
858.7
863.8
864.9
+NL (297.20): Period 12, 40 MCA scans from Sample 11 ...
Max. 2.5e5 cps.
18:3 acyl containing TAG
894.8
872.8
892.7
895.9
873.9
872.0
893.7
874.8
891.4

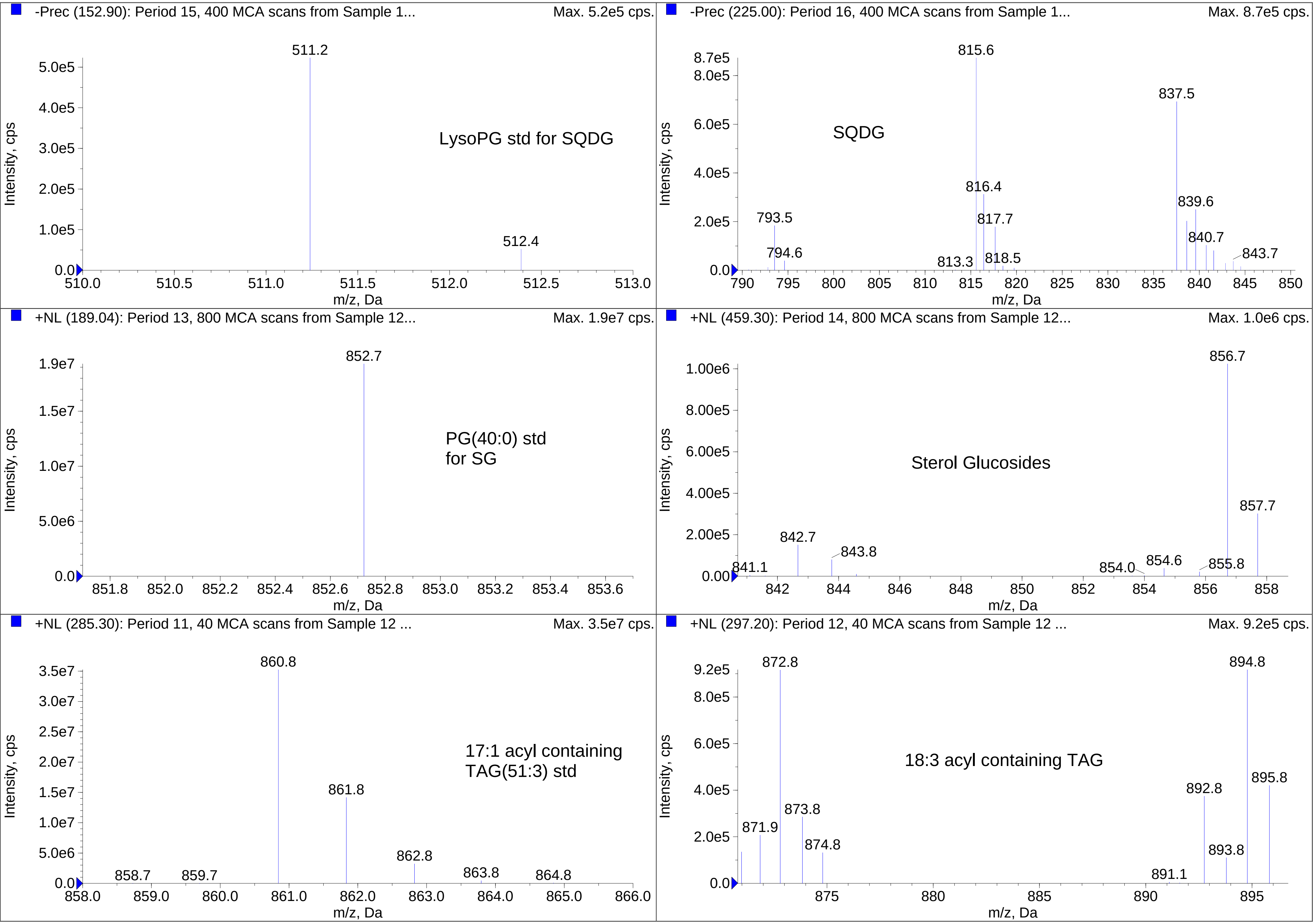
-Prec (152.90): Period 15, 400 MCA scans from Sample 1...
Max. 5.2e5 cps.
LysoPG std for SQDG
511.2
512.4
Intensity, cps
m/z, Da
-Prec (225.00): Period 16, 400 MCA scans from Sample 1...
Max. 8.7e5 cps.
SQDG
815.6
837.5
816.4
839.6
793.5
817.7
840.7
794.6
843.7
813.3
818.5
+NL (189.04): Period 13, 800 MCA scans from Sample 12...
Max. 1.9e7 cps.
PG(40:0) std
for SG
852.7
+NL (459.30): Period 14, 800 MCA scans from Sample 12...
Max. 1.0e6 cps.
Sterol Glucosides
856.7
857.7
842.7
843.8
841.1
854.0
854.6
855.8
+NL (285.30): Period 11, 40 MCA scans from Sample 12 ...
Max. 3.5e7 cps.
17:1 acyl containing
TAG(51:3) std
860.8
861.8
862.8
858.7
859.7
863.8
864.8
+NL (297.20): Period 12, 40 MCA scans from Sample 12 ...
Max. 9.2e5 cps.
18:3 acyl containing TAG
872.8
894.8
895.8
892.8
873.8
871.9
874.8
893.8
891.1

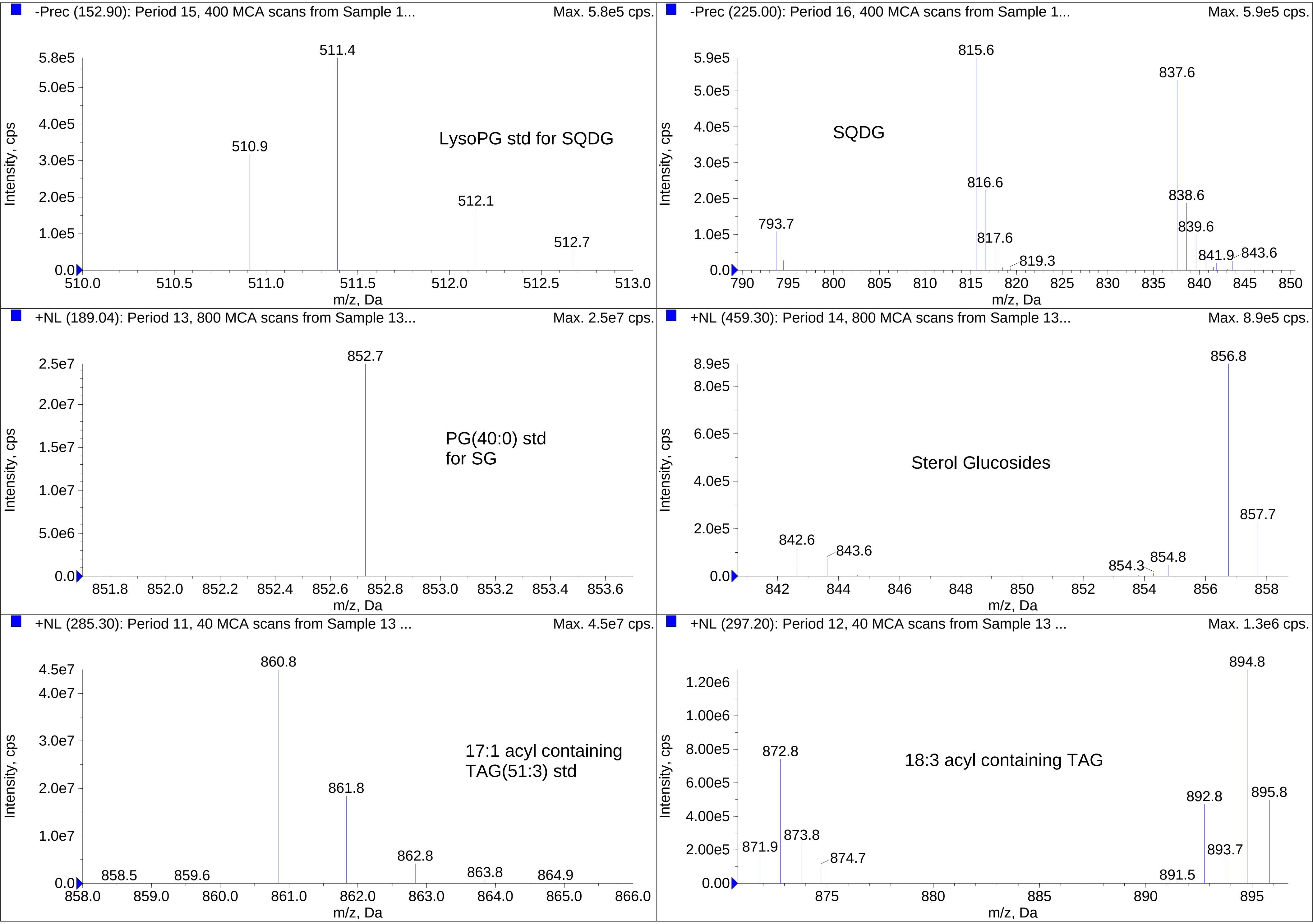
-Prec (152.90): Period 15, 400 MCA scans from Sample 1...
Max. 5.8e5 cps.
LysoPG std for SQDG
511.4
510.9
512.1
512.7
Intensity, cps
m/z, Da
-Prec (225.00): Period 16, 400 MCA scans from Sample 1...
Max. 5.9e5 cps.
SQDG
815.6
837.6
816.6
838.6
793.7
839.6
817.6
819.3
841.9
843.6
Intensity, cps
m/z, Da
+NL (189.04): Period 13, 800 MCA scans from Sample 13...
Max. 2.5e7 cps.
852.7
PG(40:0) std
for SG
Intensity, cps
m/z, Da
+NL (459.30): Period 14, 800 MCA scans from Sample 13...
Max. 8.9e5 cps.
856.8
Sterol Glucosides
857.7
842.6
843.6
854.3
854.8
Intensity, cps
m/z, Da
+NL (285.30): Period 11, 40 MCA scans from Sample 13 ...
Max. 4.5e7 cps.
860.8
17:1 acyl containing
TAG(51:3) std
861.8
862.8
858.5
859.6
863.8
864.9
Intensity, cps
m/z, Da
+NL (297.20): Period 12, 40 MCA scans from Sample 13 ...
Max. 1.3e6 cps.
894.8
872.8
18:3 acyl containing TAG
892.8
895.8
873.8
871.9
874.7
893.7
891.5
Intensity, cps
m/z, Da

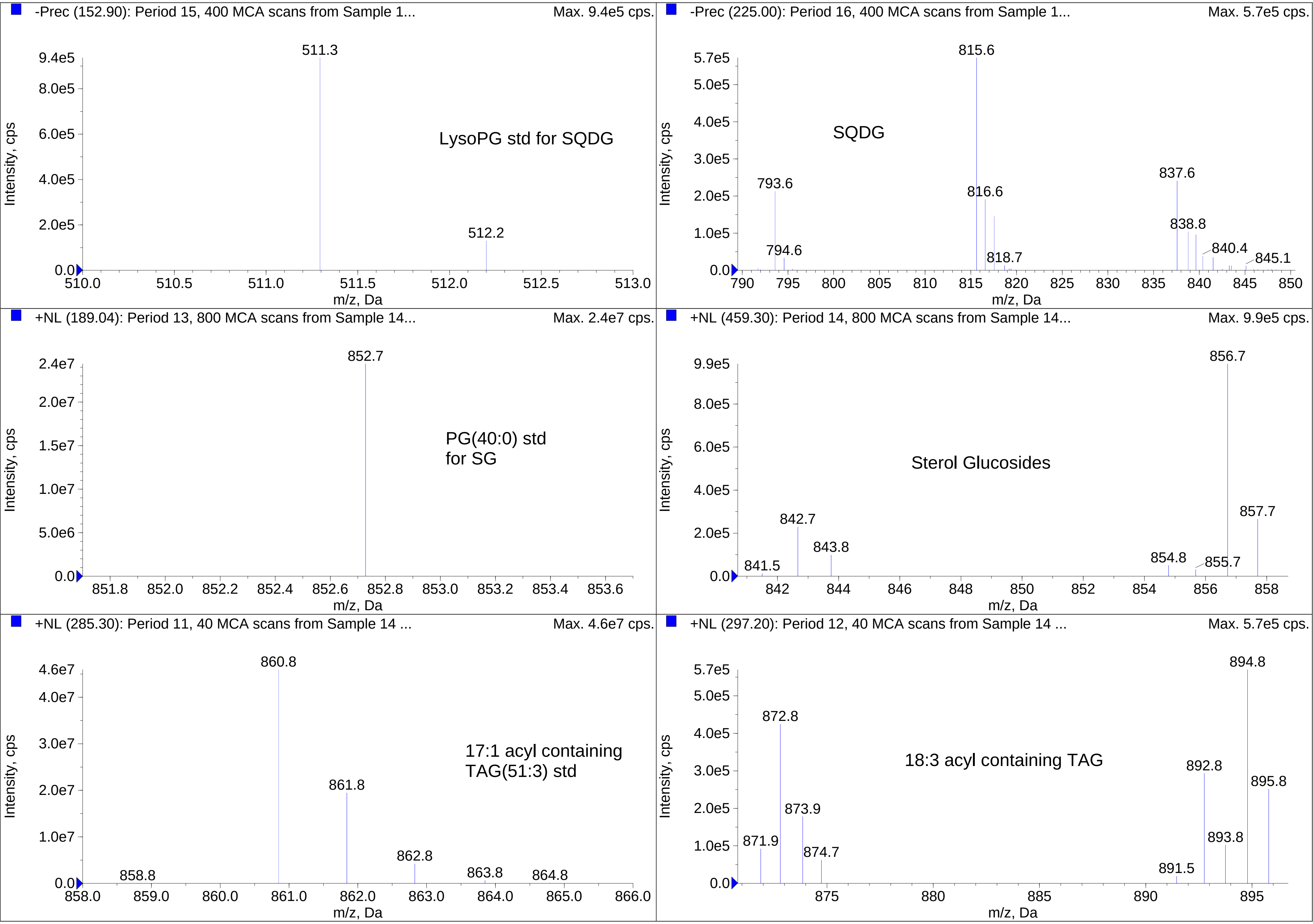
-Prec (152.90): Period 15, 400 MCA scans from Sample 1...
Max. 9.4e5 cps.
LysoPG std for SQDG
511.3
512.2
Intensity, cps
m/z, Da
-Prec (225.00): Period 16, 400 MCA scans from Sample 1...
Max. 5.7e5 cps.
SQDG
815.6
793.6
816.6
837.6
838.8
794.6
840.4
818.7
845.1
Intensity, cps
m/z, Da
+NL (189.04): Period 13, 800 MCA scans from Sample 14...
Max. 2.4e7 cps.
PG(40:0) std
for SG
852.7
Intensity, cps
m/z, Da
+NL (459.30): Period 14, 800 MCA scans from Sample 14...
Max. 9.9e5 cps.
Sterol Glucosides
856.7
857.7
842.7
843.8
854.8
841.5
855.7
Intensity, cps
m/z, Da
+NL (285.30): Period 11, 40 MCA scans from Sample 14 ...
Max. 4.6e7 cps.
17:1 acyl containing
TAG(51:3) std
860.8
861.8
862.8
858.8
863.8
864.8
Intensity, cps
m/z, Da
+NL (297.20): Period 12, 40 MCA scans from Sample 14 ...
Max. 5.7e5 cps.
18:3 acyl containing TAG
894.8
872.8
892.8
895.8
873.9
893.8
871.9
874.7
891.5
Intensity, cps
m/z, Da

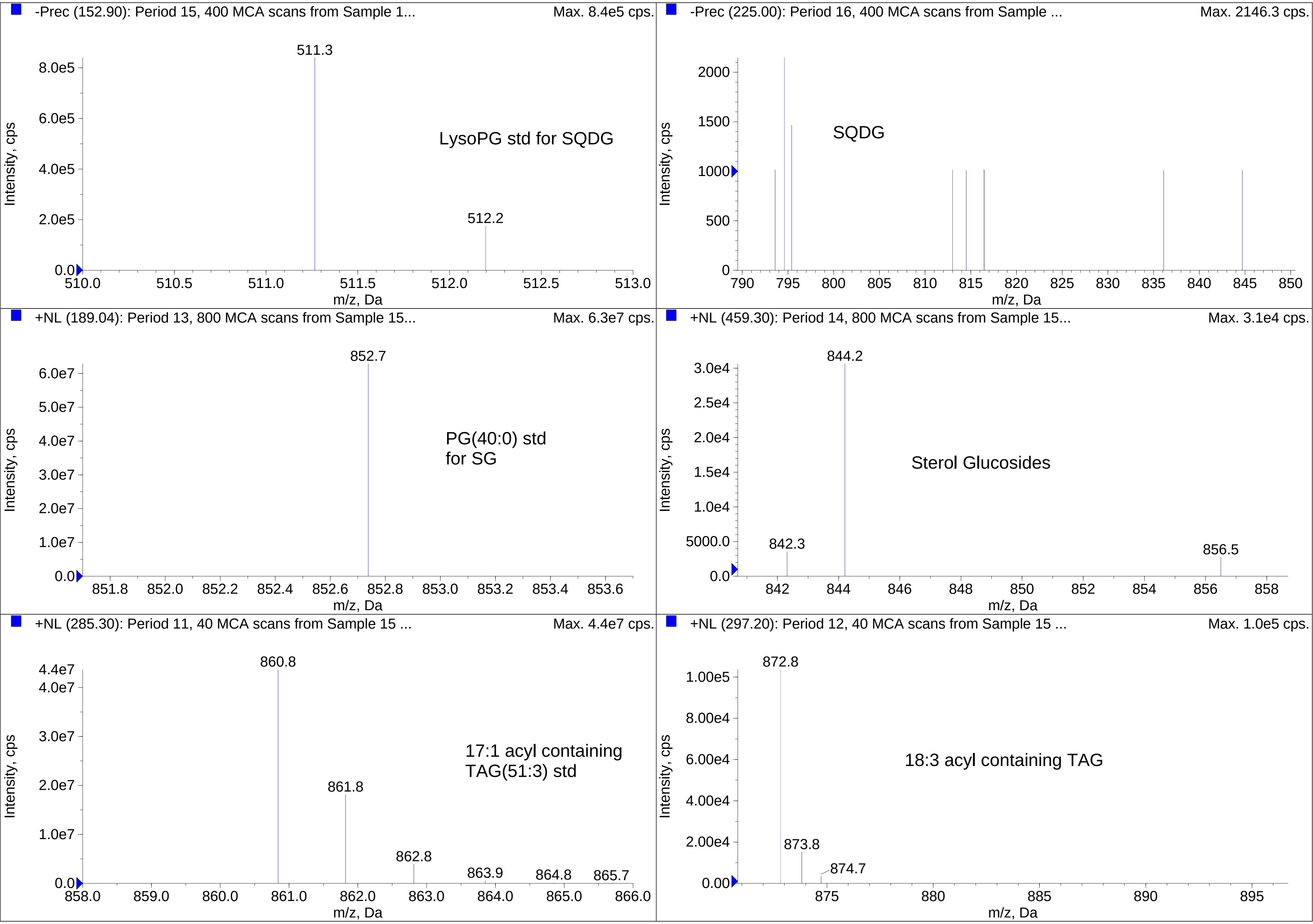

-Prec (152.90): Period 15, 400 MCA scans from Sample 1...
Max. 8.4e5 cps.
LysoPG std for SQDG
511.3
512.2
Intensity, cps
m/z, Da
-Prec (225.00): Period 16, 400 MCA scans from Sample ...
Max. 2146.3 cps.
SQDG
+NL (189.04): Period 13, 800 MCA scans from Sample 15...
Max. 6.3e7 cps.
PG(40:0) std
for SG
852.7
+NL (459.30): Period 14, 800 MCA scans from Sample 15...
Max. 3.1e4 cps.
Sterol Glucosides
844.2
842.3
856.5
+NL (285.30): Period 11, 40 MCA scans from Sample 15 ...
Max. 4.4e7 cps.
17:1 acyl containing
TAG(51:3) std
860.8
861.8
862.8
863.9
864.8
865.7
+NL (297.20): Period 12, 40 MCA scans from Sample 15 ...
Max. 1.0e5 cps.
18:3 acyl containing TAG
872.8
873.8
874.7

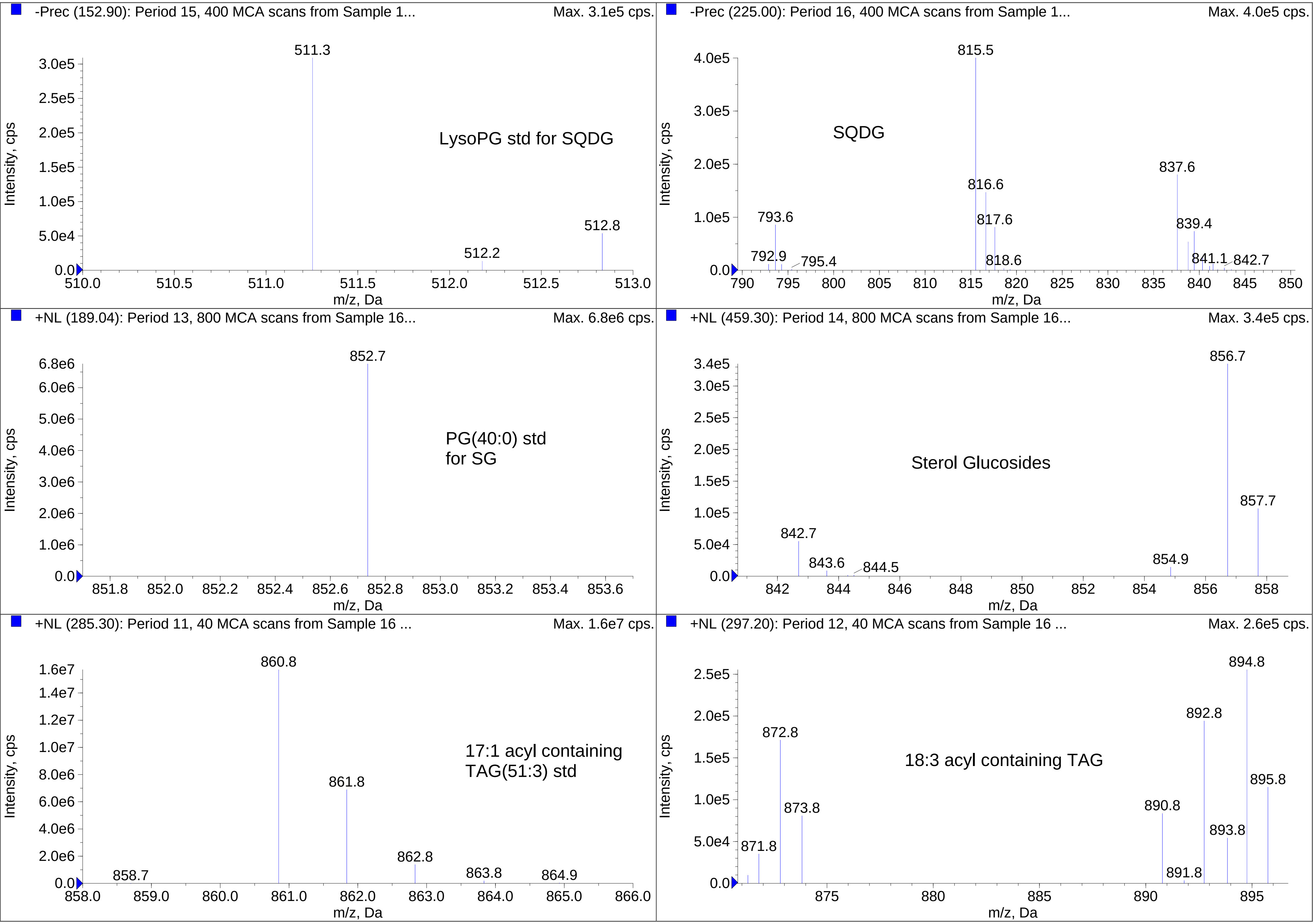
-Prec (152.90): Period 15, 400 MCA scans from Sample 1...
Max. 3.1e5 cps.
LysoPG std for SQDG
511.3
512.2
512.8
Intensity, cps
m/z, Da
-Prec (225.00): Period 16, 400 MCA scans from Sample 1...
Max. 4.0e5 cps.
SQDG
815.5
837.6
816.6
793.6
817.6
839.4
792.9
795.4
818.6
841.1
842.7
+NL (189.04): Period 13, 800 MCA scans from Sample 16...
Max. 6.8e6 cps.
PG(40:0) std
for SG
852.7
+NL (459.30): Period 14, 800 MCA scans from Sample 16...
Max. 3.4e5 cps.
Sterol Glucosides
856.7
857.7
842.7
843.6
844.5
854.9
+NL (285.30): Period 11, 40 MCA scans from Sample 16 ...
Max. 1.6e7 cps.
17:1 acyl containing
TAG(51:3) std
860.8
861.8
862.8
863.8
864.9
858.7
+NL (297.20): Period 12, 40 MCA scans from Sample 16 ...
Max. 2.6e5 cps.
18:3 acyl containing TAG
894.8
892.8
872.8
895.8
873.8
890.8
893.8
871.8
891.8

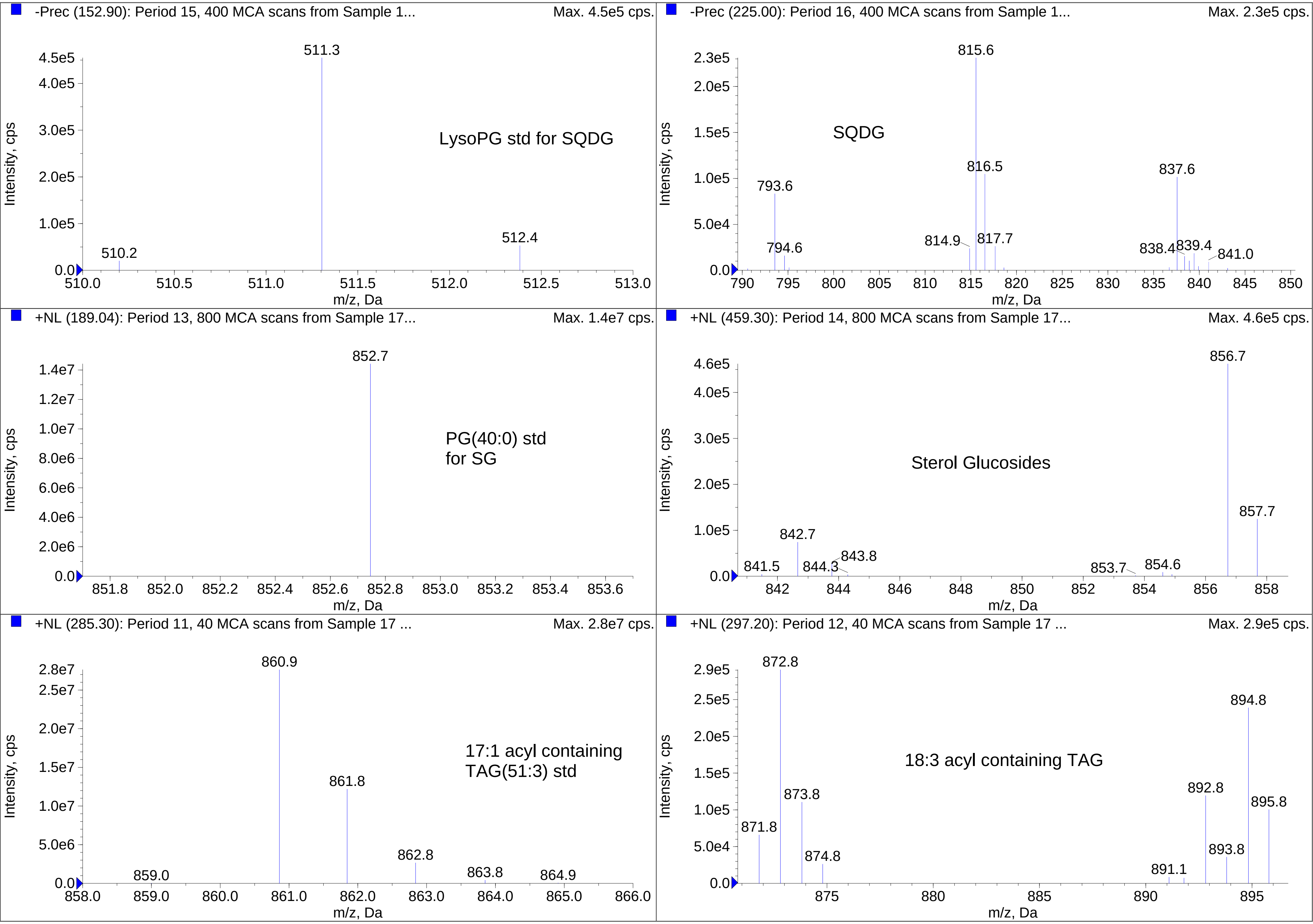
-Prec (152.90): Period 15, 400 MCA scans from Sample 1...
Max. 4.5e5 cps.
LysoPG std for SQDG
511.3
510.2
512.4
Intensity, cps
m/z, Da
-Prec (225.00): Period 16, 400 MCA scans from Sample 1...
Max. 2.3e5 cps.
SQDG
815.6
816.5
837.6
793.6
794.6
814.9
817.7
838.4
839.4
841.0
Intensity, cps
m/z, Da
+NL (189.04): Period 13, 800 MCA scans from Sample 17...
Max. 1.4e7 cps.
PG(40:0) std
for SG
852.7
Intensity, cps
m/z, Da
+NL (459.30): Period 14, 800 MCA scans from Sample 17...
Max. 4.6e5 cps.
Sterol Glucosides
856.7
857.7
842.7
841.5
844.3
843.8
853.7
854.6
Intensity, cps
m/z, Da
+NL (285.30): Period 11, 40 MCA scans from Sample 17 ...
Max. 2.8e7 cps.
17:1 acyl containing
TAG(51:3) std
860.9
861.8
862.8
859.0
863.8
864.9
Intensity, cps
m/z, Da
+NL (297.20): Period 12, 40 MCA scans from Sample 17 ...
Max. 2.9e5 cps.
18:3 acyl containing TAG
872.8
873.8
871.8
874.8
894.8
892.8
895.8
893.8
891.1
Intensity, cps
m/z, Da

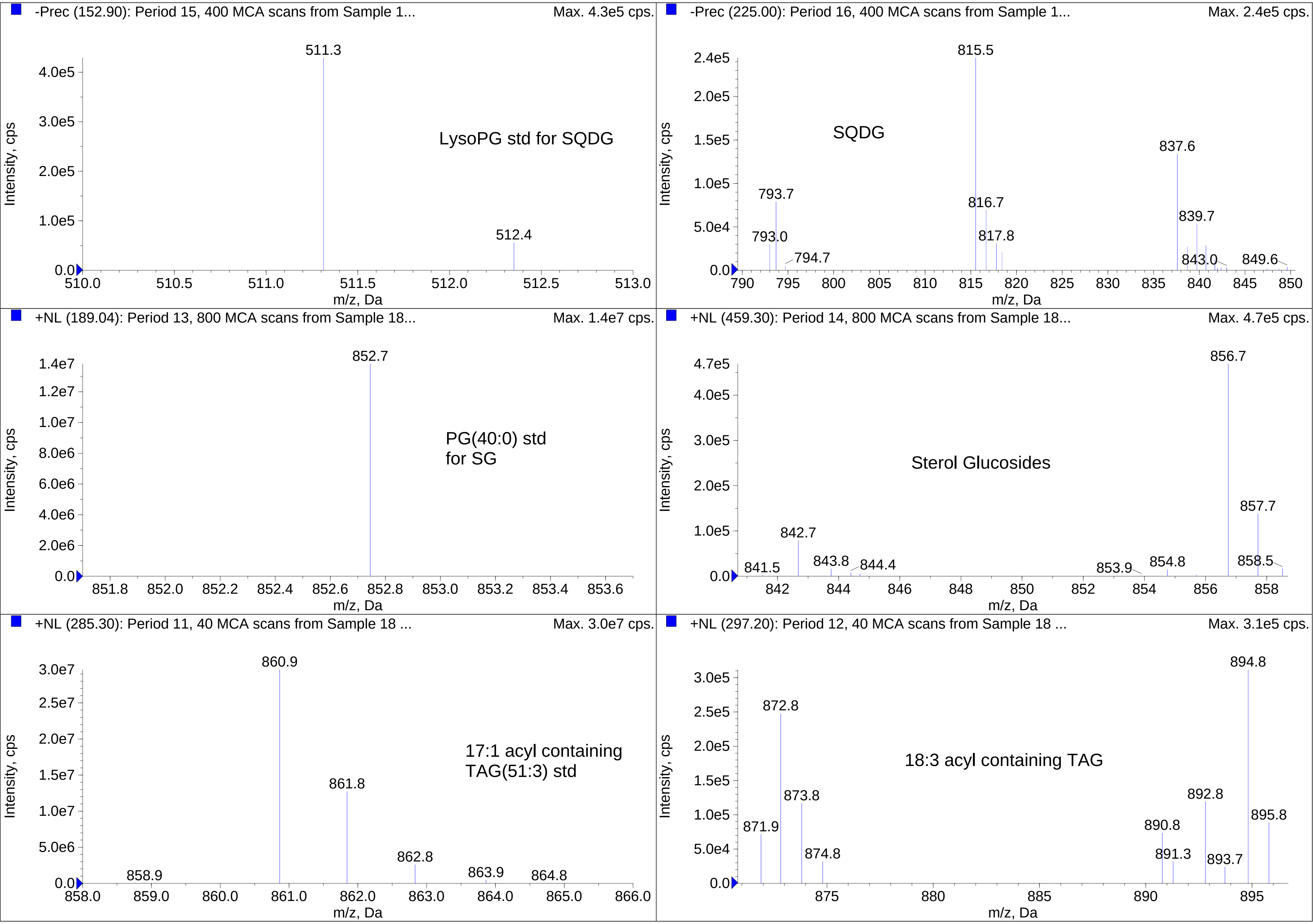
-Prec (152.90): Period 15, 400 MCA scans from Sample 1...
Max. 4.3e5 cps.
LysoPG std for SQDG
511.3
512.4
Intensity, cps
m/z, Da
-Prec (225.00): Period 16, 400 MCA scans from Sample 1...
Max. 2.4e5 cps.
SQDG
815.5
837.6
793.7
816.7
839.7
793.0
817.8
794.7
843.0
849.6
+NL (189.04): Period 13, 800 MCA scans from Sample 18...
Max. 1.4e7 cps.
PG(40:0) std
for SG
852.7
+NL (459.30): Period 14, 800 MCA scans from Sample 18...
Max. 4.7e5 cps.
Sterol Glucosides
856.7
857.7
842.7
841.5
843.8
844.4
853.9
854.8
858.5
+NL (285.30): Period 11, 40 MCA scans from Sample 18 ...
Max. 3.0e7 cps.
17:1 acyl containing
TAG(51:3) std
860.9
861.8
862.8
858.9
863.9
864.8
+NL (297.20): Period 12, 40 MCA scans from Sample 18 ...
Max. 3.1e5 cps.
18:3 acyl containing TAG
894.8
872.8
873.8
871.9
874.8
892.8
890.8
891.3
893.7
895.8

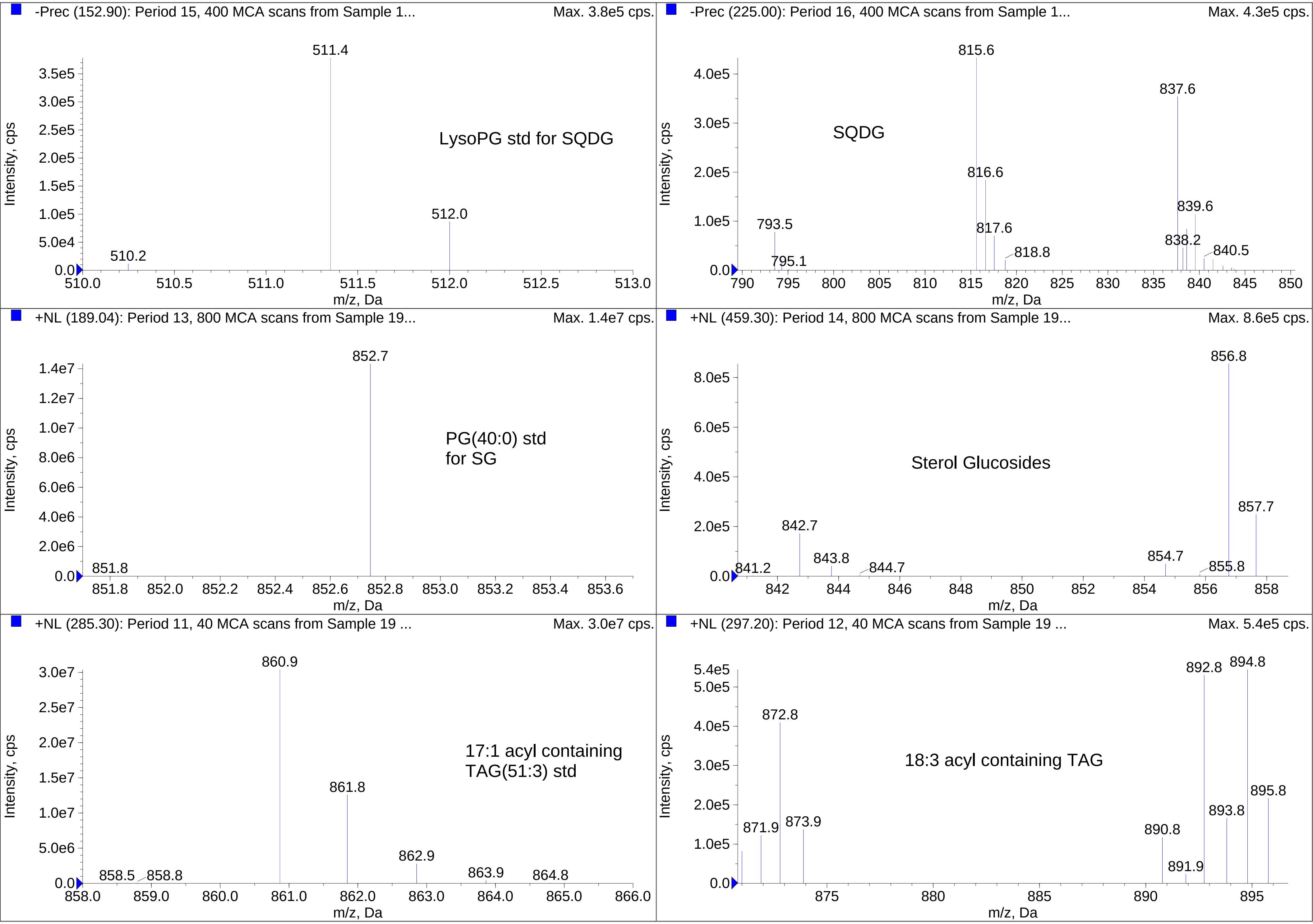

-Prec (152.90): Period 15, 400 MCA scans from Sample 1...
Max. 3.8e5 cps.
LysoPG std for SQDG
511.4
512.0
510.2
Intensity, cps
m/z, Da
-Prec (225.00): Period 16, 400 MCA scans from Sample 1...
Max. 4.3e5 cps.
SQDG
815.6
837.6
816.6
839.6
793.5
817.6
838.2
818.8
840.5
795.1
Intensity, cps
m/z, Da
+NL (189.04): Period 13, 800 MCA scans from Sample 19...
Max. 1.4e7 cps.
PG(40:0) std
for SG
852.7
851.8
Intensity, cps
m/z, Da
+NL (459.30): Period 14, 800 MCA scans from Sample 19...
Max. 8.6e5 cps.
Sterol Glucosides
856.8
857.7
842.7
843.8
844.7
841.2
854.7
855.8
Intensity, cps
m/z, Da
+NL (285.30): Period 11, 40 MCA scans from Sample 19 ...
Max. 3.0e7 cps.
17:1 acyl containing
TAG(51:3) std
860.9
861.8
862.9
863.9
864.8
858.5
858.8
Intensity, cps
m/z, Da
+NL (297.20): Period 12, 40 MCA scans from Sample 19 ...
Max. 5.4e5 cps.
18:3 acyl containing TAG
892.8
894.8
872.8
895.8
893.8
871.9
873.9
890.8
891.9
Intensity, cps
m/z, Da

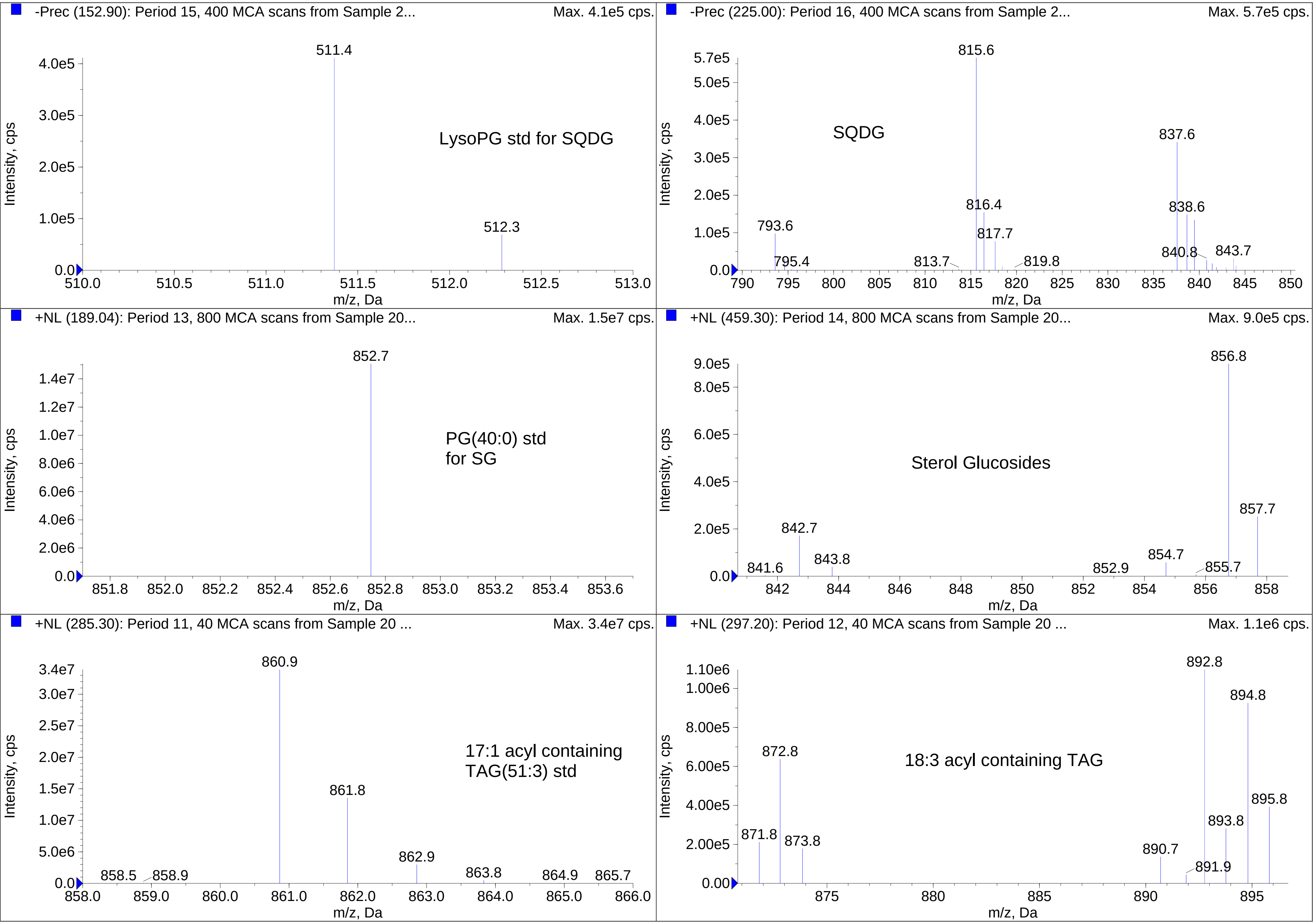
-Prec (152.90): Period 15, 400 MCA scans from Sample 2...
Max. 4.1e5 cps.
LysoPG std for SQDG
511.4
512.3
Intensity, cps
m/z, Da
-Prec (225.00): Period 16, 400 MCA scans from Sample 2...
Max. 5.7e5 cps.
SQDG
815.6
837.6
816.4
838.6
793.6
817.7
840.8
843.7
795.4
813.7
819.8
Intensity, cps
m/z, Da
+NL (189.04): Period 13, 800 MCA scans from Sample 20...
Max. 1.5e7 cps.
PG(40:0) std
for SG
852.7
Intensity, cps
m/z, Da
+NL (459.30): Period 14, 800 MCA scans from Sample 20...
Max. 9.0e5 cps.
Sterol Glucosides
856.8
857.7
842.7
843.8
841.6
852.9
854.7
855.7
Intensity, cps
m/z, Da
+NL (285.30): Period 11, 40 MCA scans from Sample 20 ...
Max. 3.4e7 cps.
17:1 acyl containing
TAG(51:3) std
860.9
861.8
862.9
858.5
858.9
863.8
864.9
865.7
Intensity, cps
m/z, Da
+NL (297.20): Period 12, 40 MCA scans from Sample 20 ...
Max. 1.1e6 cps.
18:3 acyl containing TAG
892.8
894.8
872.8
895.8
893.8
871.8
873.8
890.7
891.9
Intensity, cps
m/z, Da

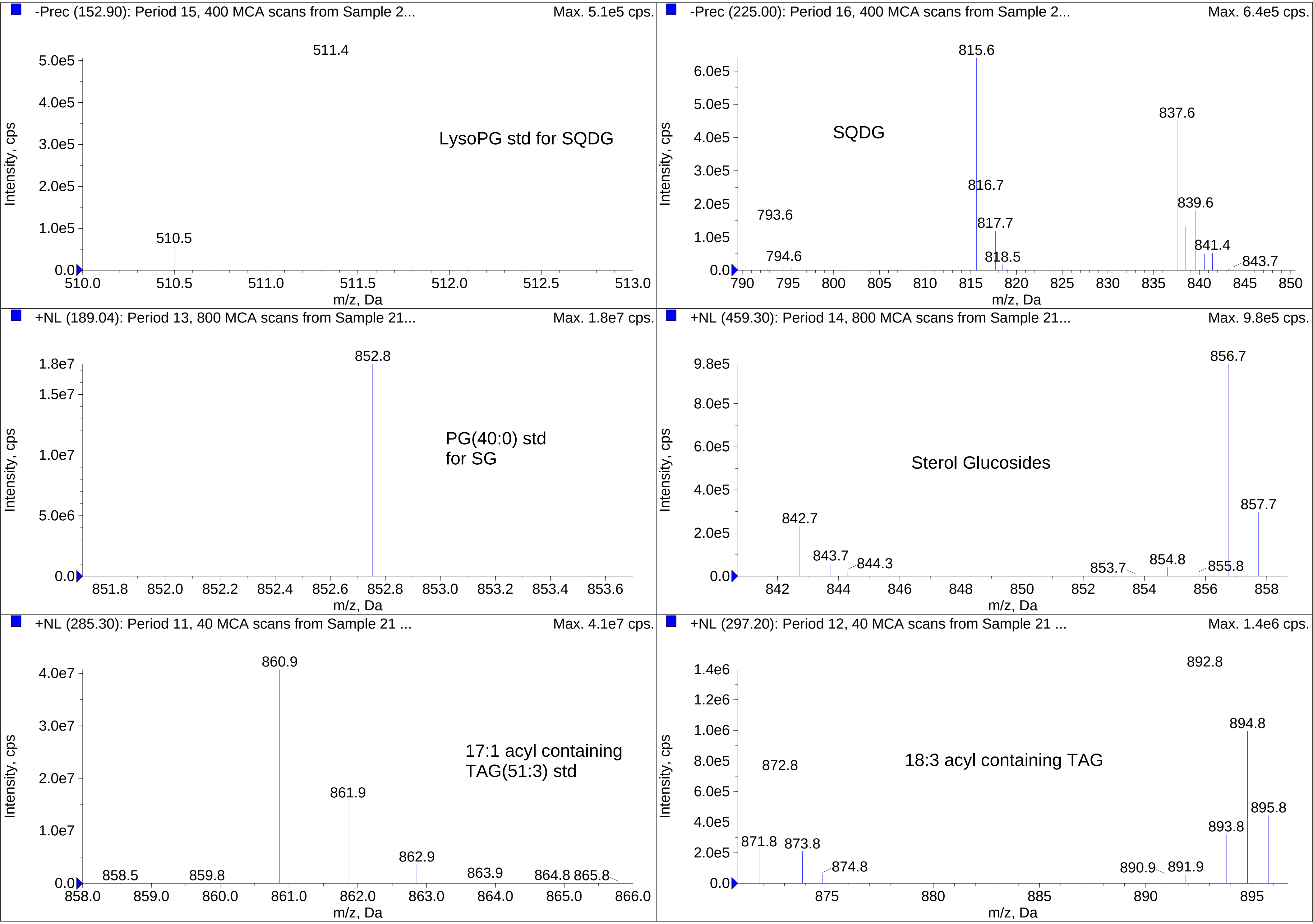
-Prec (152.90): Period 15, 400 MCA scans from Sample 2...
Max. 5.1e5 cps.
LysoPG std for SQDG
511.4
510.5
Intensity, cps
m/z, Da
-Prec (225.00): Period 16, 400 MCA scans from Sample 2...
Max. 6.4e5 cps.
SQDG
815.6
837.6
816.7
839.6
793.6
817.7
841.4
794.6
818.5
843.7
Intensity, cps
m/z, Da
+NL (189.04): Period 13, 800 MCA scans from Sample 21...
Max. 1.8e7 cps.
PG(40:0) std
for SG
852.8
Intensity, cps
m/z, Da
+NL (459.30): Period 14, 800 MCA scans from Sample 21...
Max. 9.8e5 cps.
Sterol Glucosides
856.7
857.7
842.7
843.7
844.3
853.7
854.8
855.8
Intensity, cps
m/z, Da
+NL (285.30): Period 11, 40 MCA scans from Sample 21 ...
Max. 4.1e7 cps.
17:1 acyl containing
TAG(51:3) std
860.9
861.9
862.9
858.5
859.8
863.9
864.8 865.8
Intensity, cps
m/z, Da
+NL (297.20): Period 12, 40 MCA scans from Sample 21 ...
Max. 1.4e6 cps.
18:3 acyl containing TAG
892.8
894.8
872.8
895.8
893.8
871.8
873.8
874.8
890.9
891.9
Intensity, cps
m/z, Da

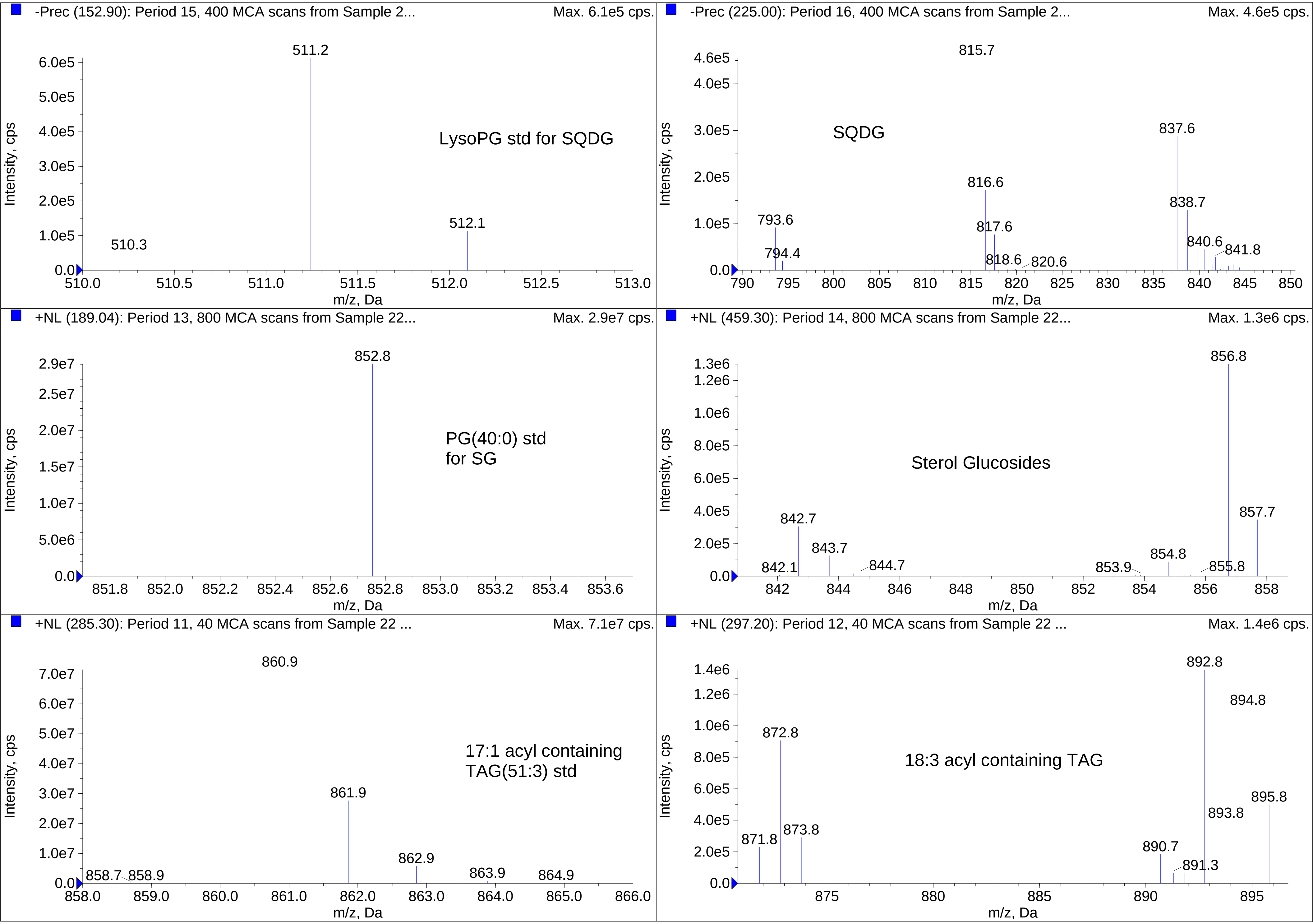

-Prec (152.90): Period 15, 400 MCA scans from Sample 2...
Max. 6.1e5 cps.
LysoPG std for SQDG
510.3
511.2
512.1
Intensity, cps
m/z, Da
-Prec (225.00): Period 16, 400 MCA scans from Sample 2...
Max. 4.6e5 cps.
SQDG
793.6
794.4
815.7
816.6
817.6
818.6
820.6
837.6
838.7
840.6
841.8
Intensity, cps
m/z, Da
+NL (189.04): Period 13, 800 MCA scans from Sample 22...
Max. 2.9e7 cps.
PG(40:0) std
for SG
852.8
Intensity, cps
m/z, Da
+NL (459.30): Period 14, 800 MCA scans from Sample 22...
Max. 1.3e6 cps.
Sterol Glucosides
842.1
842.7
843.7
844.7
853.9
854.8
855.8
856.8
857.7
Intensity, cps
m/z, Da
+NL (285.30): Period 11, 40 MCA scans from Sample 22 ...
Max. 7.1e7 cps.
17:1 acyl containing
TAG(51:3) std
858.7
858.9
860.9
861.9
862.9
863.9
864.9
Intensity, cps
m/z, Da
+NL (297.20): Period 12, 40 MCA scans from Sample 22 ...
Max. 1.4e6 cps.
18:3 acyl containing TAG
871.8
872.8
873.8
890.7
891.3
892.8
893.8
894.8
895.8
Intensity, cps
m/z, Da

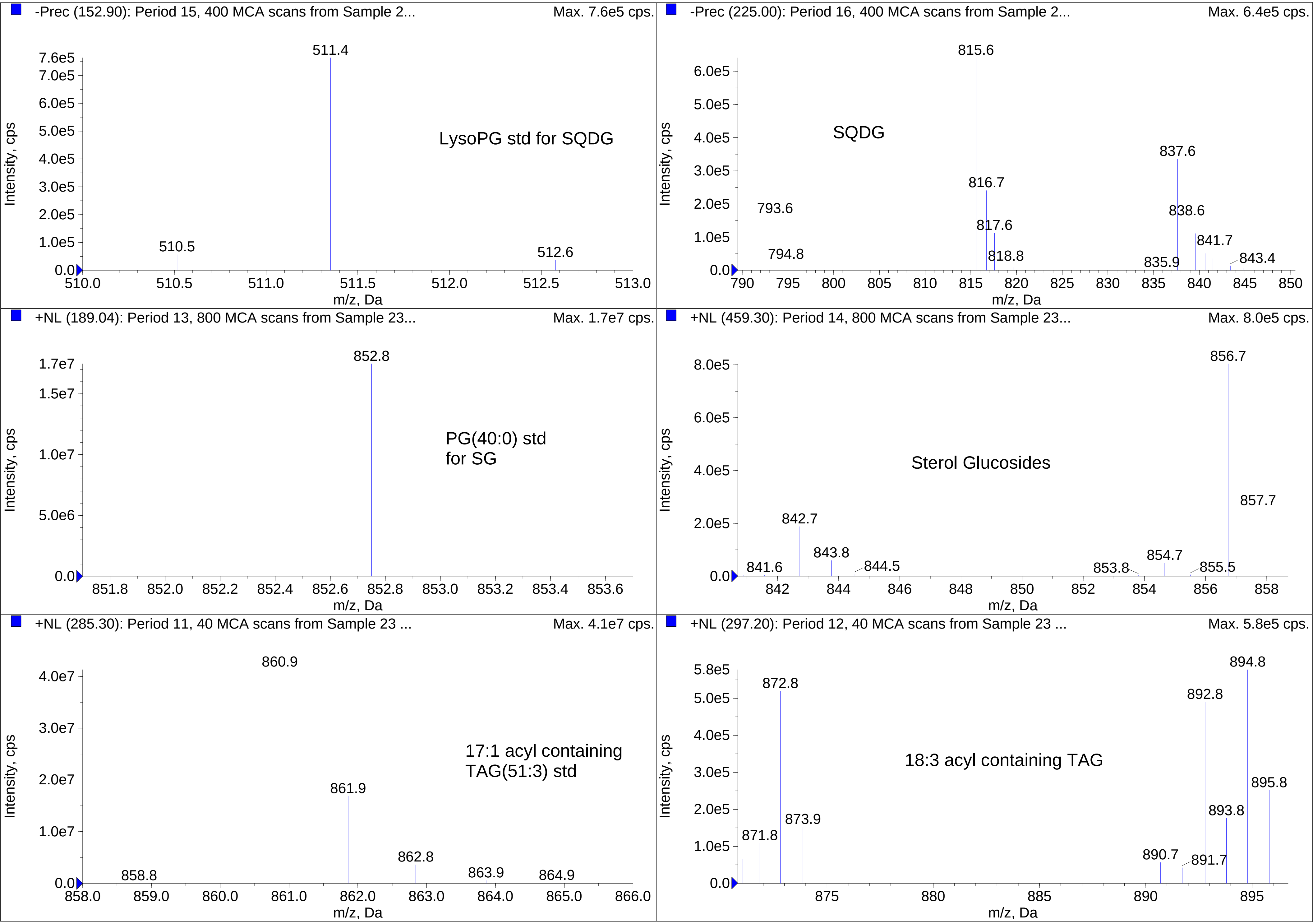

-Prec (152.90): Period 15, 400 MCA scans from Sample 2...
Max. 7.6e5 cps.
LysoPG std for SQDG
511.4
510.5
512.6
Intensity, cps
m/z, Da
-Prec (225.00): Period 16, 400 MCA scans from Sample 2...
Max. 6.4e5 cps.
SQDG
815.6
837.6
816.7
793.6
838.6
817.6
841.7
794.8
818.8
843.4
835.9
Intensity, cps
m/z, Da
+NL (189.04): Period 13, 800 MCA scans from Sample 23...
Max. 1.7e7 cps.
852.8
PG(40:0) std
for SG
Intensity, cps
m/z, Da
+NL (459.30): Period 14, 800 MCA scans from Sample 23...
Max. 8.0e5 cps.
856.7
Sterol Glucosides
857.7
842.7
843.8
854.7
841.6
844.5
853.8
855.5
Intensity, cps
m/z, Da
+NL (285.30): Period 11, 40 MCA scans from Sample 23 ...
Max. 4.1e7 cps.
860.9
17:1 acyl containing
TAG(51:3) std
861.9
862.8
858.8
863.9
864.9
Intensity, cps
m/z, Da
+NL (297.20): Period 12, 40 MCA scans from Sample 23 ...
Max. 5.8e5 cps.
894.8
872.8
892.8
18:3 acyl containing TAG
895.8
893.8
873.9
871.8
890.7
891.7
Intensity, cps
m/z, Da

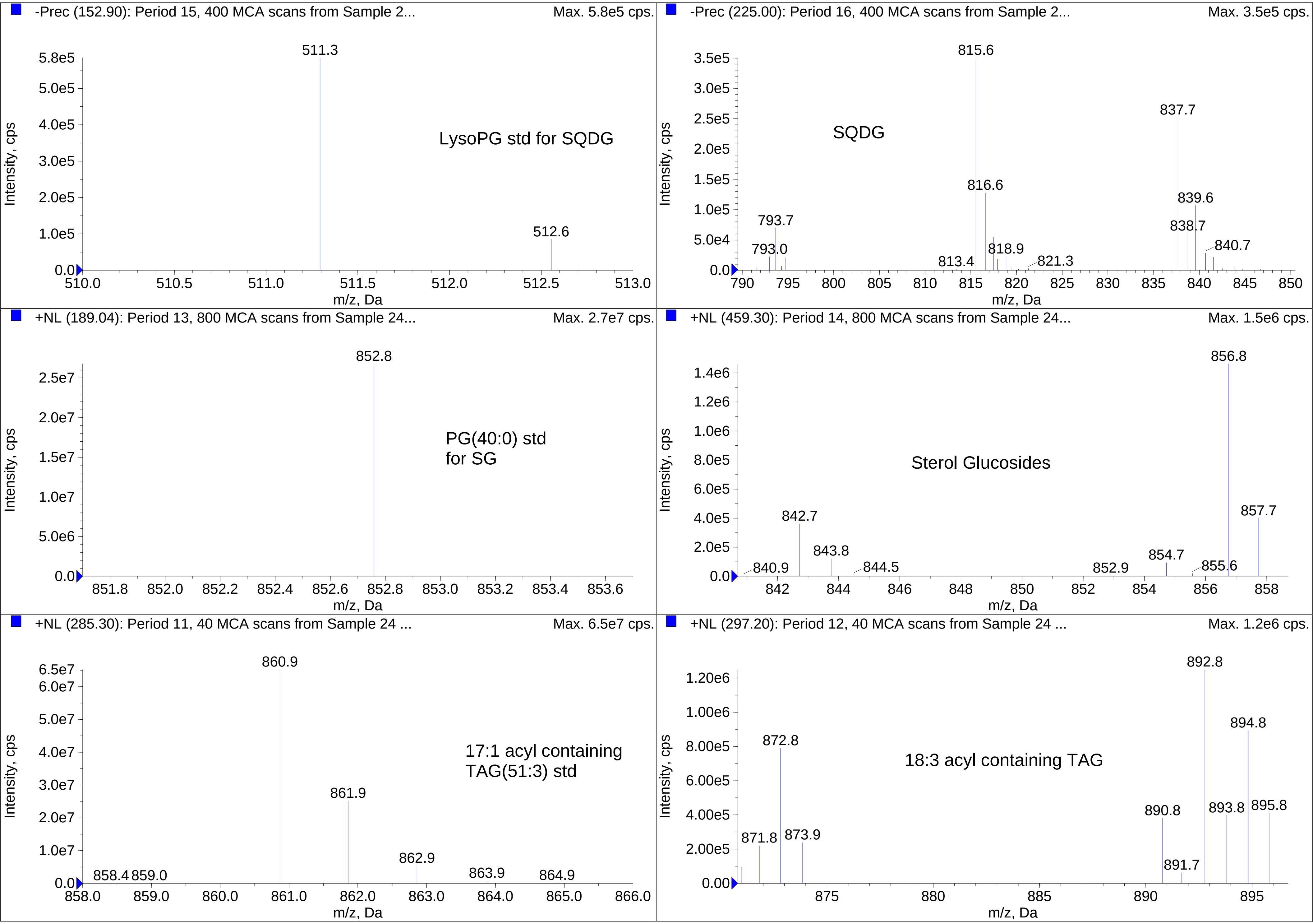
-Prec (152.90): Period 15, 400 MCA scans from Sample 2... Max. 5.8e5 cps.
LysoPG std for SQDG
-Prec (225.00): Period 16, 400 MCA scans from Sample 2... Max. 3.5e5 cps.
SQDG
+NL (189.04): Period 13, 800 MCA scans from Sample 24... Max. 2.7e7 cps.
PG(40:0) std
for SG
+NL (459.30): Period 14, 800 MCA scans from Sample 24... Max. 1.5e6 cps.
Sterol Glucosides
+NL (285.30): Period 11, 40 MCA scans from Sample 24 ... Max. 6.5e7 cps.
17:1 acyl containing
TAG(51:3) std
+NL (297.20): Period 12, 40 MCA scans from Sample 24 ... Max. 1.2e6 cps.
18:3 acyl containing TAG
Intensity, cps
m/z, Da

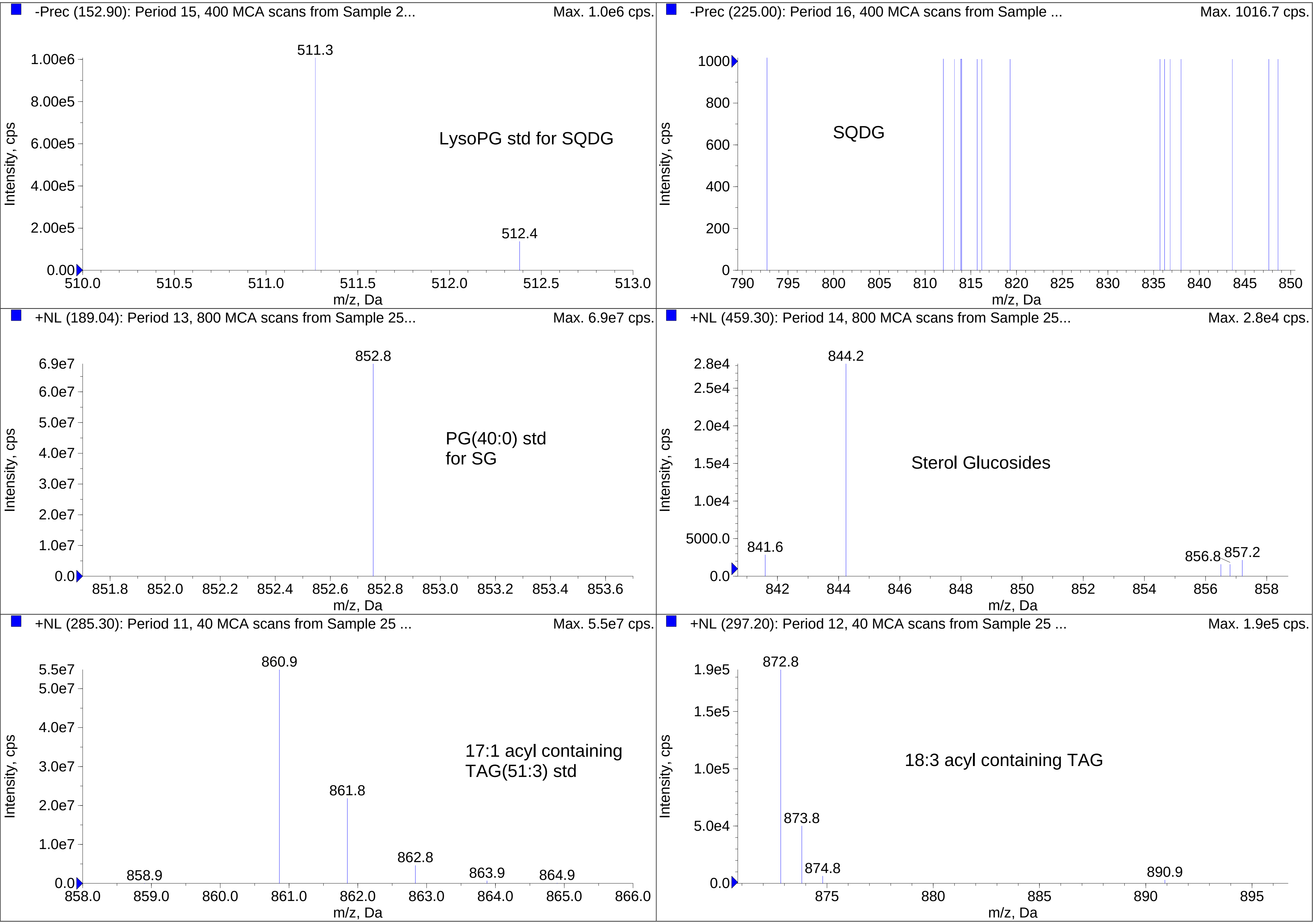
-Prec (152.90): Period 15, 400 MCA scans from Sample 2...
Max. 1.0e6 cps.
LysoPG std for SQDG
511.3
512.4
Intensity, cps
m/z, Da
-Prec (225.00): Period 16, 400 MCA scans from Sample ...
Max. 1016.7 cps.
SQDG
+NL (189.04): Period 13, 800 MCA scans from Sample 25...
Max. 6.9e7 cps.
852.8
PG(40:0) std
for SG
+NL (459.30): Period 14, 800 MCA scans from Sample 25...
Max. 2.8e4 cps.
844.2
841.6
856.8
857.2
Sterol Glucosides
+NL (285.30): Period 11, 40 MCA scans from Sample 25 ...
Max. 5.5e7 cps.
860.9
861.8
862.8
863.9
864.9
858.9
17:1 acyl containing
TAG(51:3) std
+NL (297.20): Period 12, 40 MCA scans from Sample 25 ...
Max. 1.9e5 cps.
872.8
873.8
874.8
890.9
18:3 acyl containing TAG

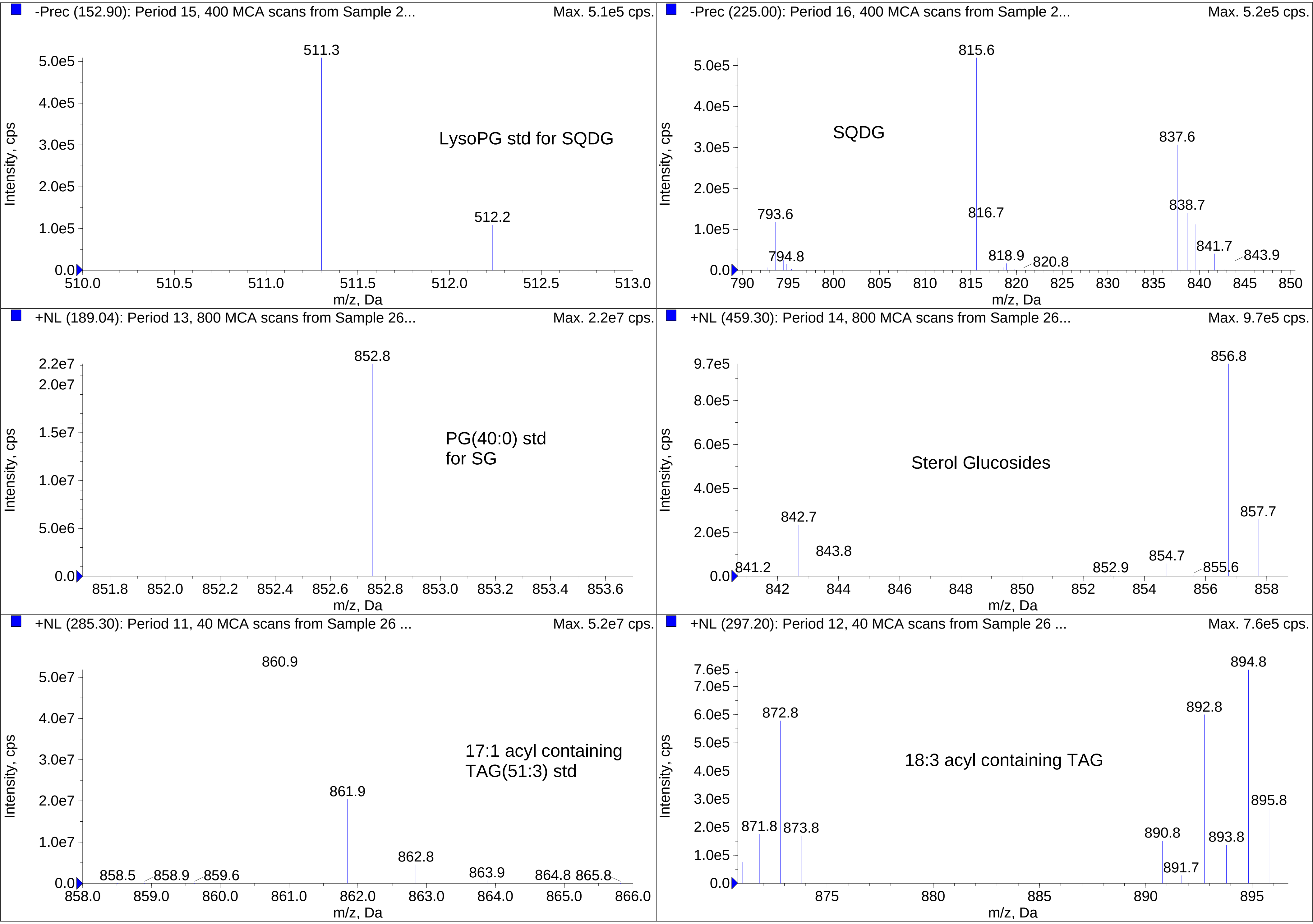


Supplementary Figure 4. Spectral specific scan of the identified lipid species
